# Shedding light on the use of graphene oxide-thiosemicarbazone hybrids towards the rapid immobilisation of methylene blue and functional coumarins†

**DOI:** 10.1039/d3na01042b

**Published:** 2024-01-10

**Authors:** Danielle Bradley, Sophia Sarpaki, Vincenzo Mirabello, Simone Giuseppe Giuffrida, Gabriele I. Kociok-Köhn, David G. Calatayud, Sofia I. Pascu

**Affiliations:** a Department of Chemistry, University of Bath Claverton Down Rd. BA2 7AY Bath UK S.Pascu@bath.ac.uk; b Department of Inorganic Chemistry, Faculty of Sciences, Universidad Autónoma de Madrid Campus de Cantoblanco, Francisco Tomas y Valiente 7, Madrid 28049 Spain david.gcalatayud@uam.es

## Abstract

Coumarins, methylene blue derivatives, as well as related functional organic dyes have become prevalent tools in life sciences and biomedicine. Their intense blue fluorescence emission makes them ideal agents for a range of applications, yet an unwanted facet of the interesting biological properties of such probes presents a simultaneous environmental threat due to inherent toxicity and persistence in aqueous media. As such, significant research efforts now ought to focus on their removal from the environment, and the sustainable trapping onto widely available, water dispersible and processable adsorbent structures such as graphene oxides could be advantageous. Additionally, flat and aromatic bis(thiosemicarbazones) (BTSCs) have shown biocompatibility and chemotherapeutic potential, as well as intrinsic fluorescence, hence traceability in the environment and in living systems. A new palette of graphene oxide-based hierarchical supramolecular materials incorporating BTSCs were prepared, characterised, and reported hereby. We report on the supramolecular entrapping of several flat, aromatic fluorogenic molecules onto graphene oxide on basis of non-covalent interactions, by virtue of their structural features with potential to form aromatic stacks and H-bonds. The evaluations of the binding interactions in solution by between organic dyes (methylene blue and functional coumarins) and new graphene oxide-anchored Zn(ii) derivatised bis(thiosemicarbazones) nanohybrids were carried out by UV-Vis and fluorescence spectroscopies.

## Abstract 2

Methylene blue (MB) and other functional organic dyes such as coumarins are known pollutants, which can be immobilised by adsorbtion on graphene oxide nanohybrids and/or photo-catalytically degraded.

## Graphical abstract

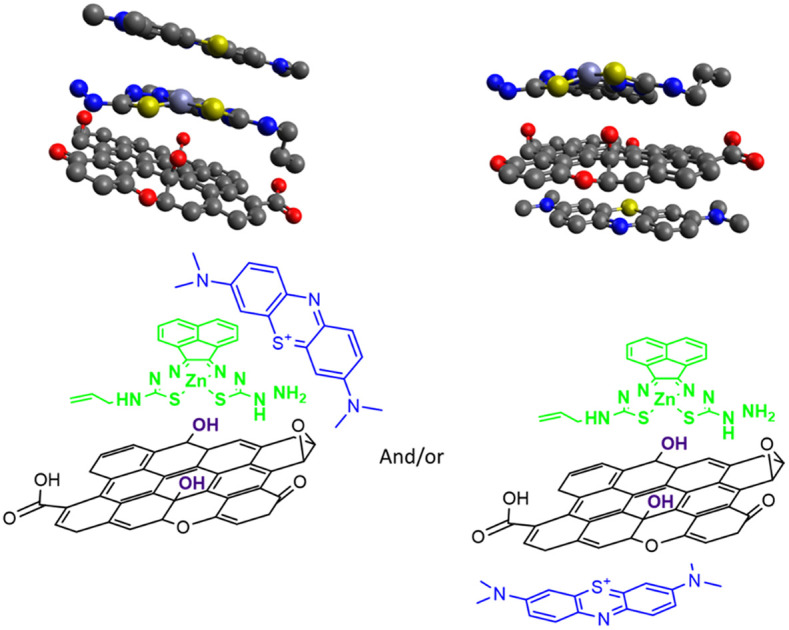

## Introduction

The vast synthetic potential of graphene oxide (GO) remains largely unexplored due to the intricate and heterogeneous nature of its layered structure, posing substantial challenges in chemical exploration and precise functionalisation. This investigation addresses the intricate landscape of graphene-based functional nanohybrids through their potential for the supramolecular entrapment of flat, aromatic fluorogenic molecules onto the aromatic surfaces. We aim to introduce a new repertoire of graphene-based hierarchical supramolecular materials designed for the sustainable trapping of organic contaminants, which are often fluorogenic molecules.

Graphene was first isolated in the early 21^st^ century by Novoselov and Geim earning them the Nobel Prize in Physics. This new material consists of carbons bound in a honeycomb lattice constructing a 2D planar surface enriched in sp^2^ layer orbitals. Two main functional graphene-based derivatives have been reported and studied widely: graphene oxide (GO) and reduced graphene oxide (rGO).^1–6^ The graphene oxide surface contains uncharged epoxides (–O–) allowing the binding with organic molecules such as dyes, chemotherapeutic drugs *etc.* through aromatic stacking, hydrogen bonding or other noncovalent interactions. GO nanoflakes are additionally decorated with hydroxyl (–OH) groups on the peripheral surface which increases the hydrophilicity of corresponding nanoflakes^1–6^ which render this materials suitable for applications such as drug delivery, biosensing and environmental remediation. Graphene and its functional congeners such as graphene oxide (GO) and reduced graphene oxide (rGO) have presented ample opportunities for the development of a broad range of biomedical applications. Due to graphene's tuneable surface, its lower toxicity and to its superior biocompatibility in comparison with other carbon-based nanomaterials and metallic nanoparticles, its use has been intensively explored as a nanocarrier for drug/gene delivery^7–10^ or as synthetic platform for biosensors^1,10–13^ and theranostics.^14,15^ Recent developments have shown that a variety of anticancer molecules (doxorubicin, camptothecin, nucleic acids *etc.*) can be incorporated onto the GO's surface.^16–18^ The main challenge of GOs nanocomposites, similarly to the case of most nanoparticles, is to mediate selective drug delivery: tumour-targeted GO drug carriers have been reported and the GO's potential to reduce reactive oxygen species, of relevance to tumour microenvironment targeting, has also been proposed.^19^ The GOs toxicity may be affected by a range of parameters such as the number of layers, the size, the surface, the time of exposure or the cell type, and its heterogeneity pose a real challenge for any level of quantification for such interactions with biological environment.^1,10,20^ Its biocompatibility has been achieved by further functionalisation of the GO surface with folic acid, polyethylene glycol (PEG), or other biomolecules such as monoclonal antibodies that target a specific cancer type.^9,21^ State-of-the-art showed that engineered GO nanocomposites have been explored for their potential to act as nano-platforms for a variety of imaging techniques, such as MRI,^1,22^ as fluorescence markers allowing intracellular imaging studies^1,22–25^ or nuclear imaging techniques for pre-clinical *in vivo* applications^1,26,27^ They were shown to be effective in the immobilisation of radioactive metal ions of biomedical importance in theranostics, and in acting as synthetic platforms for the combination of different imaging modalities within the same nanocomposite.^1,12,28,29^

The role of graphene as a synthetic scaffold for supramolecular chemistry was instrumental in the development of functional materials for a variety of applications ranging from materials sciences, to biomedicine and sustainable catalysis including photocatalysis.^30,31^ The high electron mobility and high thermal conductivity of graphene can be explained by the 2D structural array of sp^2^ hybridized carbon atoms, which allows conduction of electrons.^32^ Graphene has the potential to be used in solar cells to enhance the efficacy as a component to a multilayer thin film systems,^33^ and such applications may be extended to graphene oxides and related nanohybrids. Carbon nanomaterials (including carbon nanotubes, additionally to graphene oxides) have been used to immobilize molecules in supramolecular assemblies (which often exhibit π–π stacking interactions between the extended aromatic layers)^34^ and their surface can be deliberately modified to assist the extraction of heavy metals in water remediation and to act as suitable adsorbent structures for organic molecules. For example, the binding of 8-hydroxyquinoline to carbon nanotubes has been shown to improve the selectivity of heavy metal removal *via* chelation.^35^ A straightforward technique to filter pollutants from water effectively has been to use a separation column equipped with functionalized carbon nanotube (CNT).^36^ However, using CNTs as proposed devices for the removal of water pollutants, may not represent sustainable solutions, for all types of environmental remediations considering the scale-up challenges involved and the inherent toxicity of air-borne carbon nanotubes. Instead, functional bulk graphene-based substrates such as graphene oxides which already incorporate in hydrophilic groups, can be a more societally and industrially acceptable solution to environmental remediation. Several advantages and disadvantages exist for all methods of synthesis of functional graphene, which require consideration for large scale applications. For example, Chemical Vapour Deposition (CVD) generates bulk graphene but often requires high temperatures to generate gaseous precursors. This technique provides high quality graphene with minimal defects yet further functionalities may have to be introduced for environmental compatibility.^37^ Due to advantages such as reduced toxicity of reagents, batch-to-batch reproducibility, scalability and yield of reactions, functionalised GO emerged as a versatile synthetic building block for suitable adsorbent nanomaterials. The conversion of graphite to GO (*via* graphite oxide which is then exfoliated) is often carried out using synthetic adaptations of the Hummer's method as a major route to produce GO.^38^

The technique involves the oxidation of graphite flakes using sodium nitrate, potassium permanganate and sulphuric acid, and has more recently been adapted to achieve a ‘greener’ synthesis which avoids the harmful production of nitric oxide by eliminating sodium nitrate, whilst maintaining a similar yield.^39^

The functionalisation of graphene to give graphene oxide also gave rise to a new realm of materials suitable for sustainable technologies applications due to the abundance of oxygen-containing functional groups (–OH, –C=O, –COOH and epoxides) on the surface, ability to engage in H-bonding, aromatic stacking and electrostatic interactions, and, if the binding substrate contains a metal ion, participate in coordination chemistry too. The covalent or non-covalent binding of organic molecules to the oxygen functionalities of GO can help tune the physico-chemical characteristics and spectroscopic properties of emerging GO-based nanohybrids. It has been demonstrated that GO species can interact with dyes such as free base or metalloporphyrins incorporating Zn(ii), Cu(ii) and Sn(iv) ions. The nature of their interactions in solution has been studied by fluorescence spectroscopy, whereby an energy transfer process has been described, with energy transfer occurring from the excited porphyrin molecules to the GO nanoflakes.^40^ The large surface area of GO also provides scope for the adsorption of target molecules on the surface. Combined with the low cost and ease of scalability these render GO as an ideal candidate for sustainable technologies.

The removal of heavy metals and toxic dyes in water is a significant issue environmentally. Through a combination of aromatic stacking, H-bonding interaction and interlayer hydrophobic interactions GO has the potential to facilitate the surface adsorption of water pollutants and to enable the recovering followed by disposal of toxic organic dyes and/or metallic contaminant species. GO has a demonstrated ability to adsorb heavy divalent metal ions such as Pb^2+^ and Cd^2+^ which then precipitate out of solution providing a means to ultimately remove the heavy metal ions.^41^ Other studies employed GO in water remediation to remove heavy metal ions, with the main problems encountered including, the sensitivity to pH changes and challenges posed by the adsorption phenomena for such ions in low concentrations.^42^ Water-soluble Fe_3_O_4_ nanoparticles were shown to interact with GO and the resulting suspension has subsequently been used to ‘encapsulate’ dyes such as methylene blue (MB) through bonding modes such as hydrogen bonding to the alcohol groups on polyol or GO, π–π stacking of aromatic rings, and/or electrostatic interactions from the charged dye.^43^

**Fig. 1:**
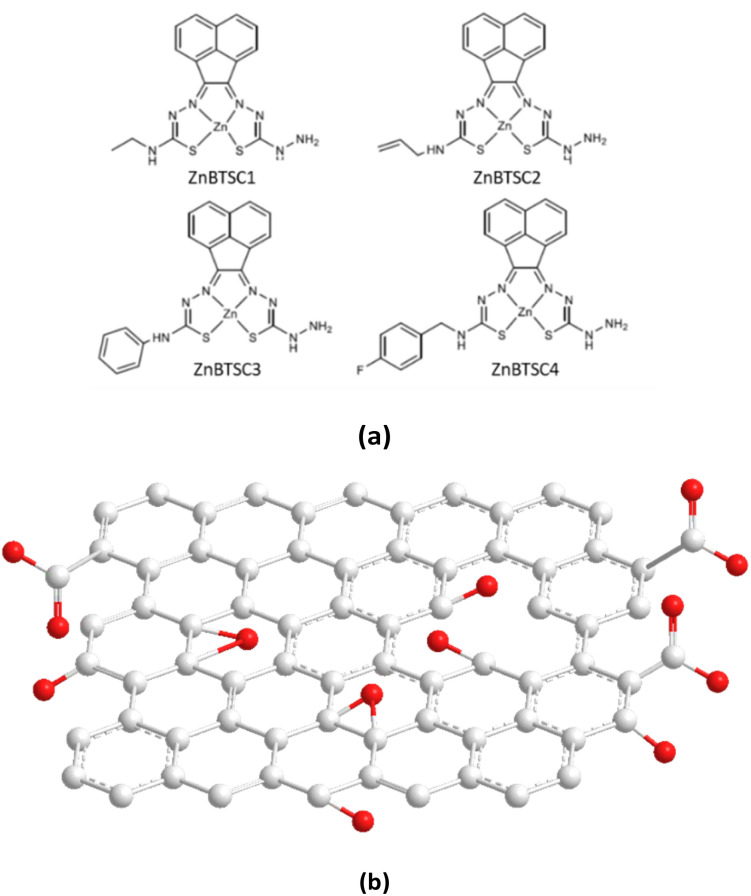
Overview of: (a) the asymmetrically substituted BTSCs as nanohybrid components of interest for this work; and (b) the structural representation of the typical MM+ minimised GO nanoflake as a generalised fragment, represented schematically in agreement with previous reports.^6^

Photocatalytic activation with UV light can be used to facilitate the destruction of the immobilised dye as a complementary possible avenue towards the total elimination of such organic dyes as water pollutants. It has been reported with the photocatalytic oxidation of methylene blue using a graphitic carbon nitride (C_3_N_4_) catalyst, which initiates decomposition of the dye.^44^ Recent studies have focused on producing nanocomposites of GO to provide an alternate route to achieving successful water remediation *via* photocatalytic approaches.^45^ Here we report upon our recent investigations into novel hybrids emerging from the self-assembly of flat, aromatic metal Zn(ii) bis(thiosemicarbazonato) complexes (ZnBTSC) onto GO nanoflakes. These complexes may provide unique and advantageous properties to the GO, and are explored in the context of the role of such supramolecular assemblies, (denoted ZnBTSC@GO) towards organic dyes adsorbtion and removal from aqueous environments. Building blocks applied to immobilise methylene blue and functional coumarins in this work are depicted in Fig. 1.

This article reports upon our recent investigations into:

(a) Synthesis of new asymmetric Zn(ii) bis(thiosemicarbazonato) complexes as building blocks for new nanohybrids incorporating GO;

(b) Spectroscopic insights into the nature of interaction between the Zn(ii) BTSCs and graphene oxide within the hybrids denoted generally ZnBTSC@GO which complement the microscopy studies;

(c) Assessment of the ability of these nanohybrids to trap rapidly methylene blue dye, with, and without photocatalytic activation using violet light irradiation.

(d) An evaluation of the ability of water-soluble coumarin dyes to interact with a ZnBTSC@GO nanohybrid and give rise to new fluorogenic materials.

## Results and discussion

### Synthesis and characterisation of new asymmetric ZnBTSC complexes and their GO hybrids ZnBTSC@GO

Building blocks applied to immobilise methylene blue and functional coumarins in this work are depicted in Fig. 1. Unsymmetrical zinc-substituted bis(thiosemicarbazone) complexes (ZnBTSC) have shown potential to aggregate and to form donor–acceptor interactions with other substrates through H-bonds, aromatic π–π stacking.^46^ We synthesised a series of simple BTSC complexes with asymmetric frameworks which present a free NH_2_ group available to bind either covalently or non-covalently (through H bonds) to the oxygen-reach functionalities of the GO surface. Maintaining a free hydrazine linker on one arm of the TSC moiety as a molecular design feature was deemed important to facilitate the occurrence of non-covalent interactions. Several functional groups can be attached on one ‘arm’ of the thiosemicarbazone (TSC) ligand, giving rise to asymmetric, flat, aromatic system shown in Fig. 1.

Here, a new palette of unsymmetrical ZnBTSC complexes featuring –NH_2_ groups and also four different substituents were selected for investigation, where R = ethyl (ZnBTSC1), allyl (ZnBTSC2), phenyl (ZnBTSC3), methyl-(4-fluorobenzyl) (ZnBTSC4).

An optimised microwave reaction was used to synthesise all mono substituted TSCs in relatively high yield (60–80%) over short reaction times (less than 90 min). To afford the desired [ZnBTSC] complexes, the known^47^ corresponding mono-substituted thiosemicarbazone were treated with 5 equivalents of zinc acetate and then heated for 30 hours with thiocarbohydrazide at 120 °C, giving rise to the desired asymmetric thiosemicarbazones *via* established protocols.^48^ These compounds were synthesised in high purity and analysed using a range of standard spectroscopic methods (see Experimental section).

The comparison of the ^1^H NMR spectra of compound ZnBTSC3 and the mono-substituted ligand (ESI†) reflects the structural change from reaction of mono-substituted ligand to the asymmetric Zn(ii) BTSC. Mass spectrometry provided further support of the final structures in addition to the individual ^1^H and ^13^C{^1^H}NMR assignments of the mono-substituted TSC precursors for all complexes and resulting asymmetric ZnBTSCs. The fluorescence spectra of the ZnBTSC1–4 described here generally observe broad emission peaks at around 400–700 nm when excited at *λ*_ex_ = 320–350 nm (ESI†). The interactions of these ZnBTSCs with GO are postulated to be driven by π–π stacking between aromatic rings, hydrogen bonding from heteroatom electron lone pairs, as well as by the Zn⋯OH or Zn⋯HO(O)C– bonds formation (involving the 5th possible coordination site of Zn(ii)complex) based on the deliberate design elements included. Here we hypothesise that these properties can facilitate their binding to complementary organic species compounds, by donor–acceptor interactions, as well as to graphene oxide nanoflakes, however self-aggregation of ZnBTSC through Zn⋯NH_2_ extended interactions in organic solvents and/or in solid state cannot be discounted, analogous to the related and well-studied Zn(ATSM)/A complex.^49,50^ We observed spectroscopically the change in optical properties in presence or absence of graphene oxides to explored subtle substituents effects due to the functional groups attached at the exocyclic N's. The nature of the interaction between the Zn(ii) complexes, ZnBTSCs (shown in Fig. 1a and b) with GO nanoflakes may govern the properties displayed by their corresponding ZnBTSC@GO nanohybrids.

First, a reliable process was employed to give rise to graphene oxide scaffolds, which were prepared using an adapted Hummer's method from graphite flakes,^1^ a synthetic method widely used to produce graphene oxide from graphene flakes with well-established batch-to-batch reproducibility.^6^ The graphite oxide intermediate was exfoliated before use (by sonnication, as described in ESI†) and characterised by Raman spectroscopy and TEM in a range of organic solvents, and showed a high batch-to-batch structural consistency (ESI†).

**Fig. 2:**
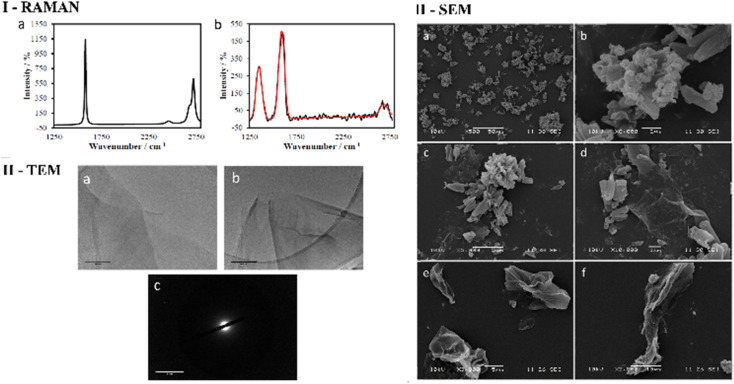
(I) Raman spectra of: (a) graphite in THF (1 mg/1 mL) displays a G (1600 cm^−1^) band and G* (2700 cm^−1^) band, (b) GO in THF (1 mg/1 mL) displays a D band (1350 cm^−1^), G (1600 cm^−1^) band and G* (2750 cm^−1^) band (red line: curve of best fit). (II) TEM images of (a) and (b) GO flakes in ethanol (0.25 mg/1 mL) at 200 nm 500 nm scale, (c) SAED at 2 nm scale on lacey carbon grids. (III) SEM analysis of (a) compound ZnBTSC3 solid at 50 μm scale. (b) Compound ZnBTSC3 solid at 2 μm scale. (c) Compound ZnBTSC3 with GO at 5 μm scale. (d) Compound ZnBTSC3 with GO at 1 μm scale. (e) Free GO at 5 μm scale. (f) Free GO at 10 μm scale.

**Fig. 3:**
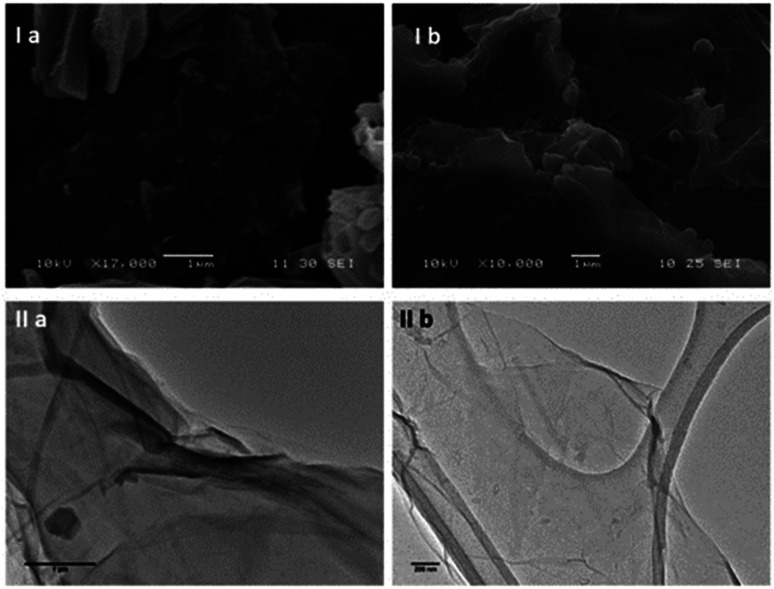
(I) FESEM images of (a) compound ZnBTSC3 and (b) compound ZnBTSC4, whereby solid samples were deposited on HOPG as the carbon support and gold coated. (II) TEM micrographs recorded on lacey carbon support: (a) ZnBTSC3@GO hybrid (scale bar: 1 μm). (b) ZnBTSC4@GO nanohybrid (scale bar: 500 μm).

As expected, the Raman spectroscopy of graphene oxide produced three peaks, that correspond to D, G and G* (also referred to as 2D) bands respectively (Fig. 2 and ESI†), which are in accordance with spectra reported in other studies^51,52^ The main structural differences between graphite starting material and GO reside in the nature of the interactions between the graphene sheets due to stacking and the oxidation functionalities present on the surface. It is therefore reasonable to assume that the change in spectra is a consequence of the interaction between layers of GO since the D band arises upon oxidation. It is well known that for functional graphene substrates a shift of the 2D band from approximately 2700 cm^−1^ to 2750 cm^−1^ may be observed due to the nature of the organic solvent involved (*e.g.* DMSO, EtOH or THF). Transmission electron microscopy (TEM) was used to investigate the morphology of GO dispersed in ethanol. Fig. 2 and 3 display the images recorded at 1 μm and 500 nm scale of graphene flakes, which possess various folding formations evidential of the disruptive defects and the introduction of sp^3^ hybridised carbons. Without these topographical features a sheet-like appearance might be expected, suggesting the occurrence of stacks of graphene layers and a planar assembly. Thus, based on these prominent features with respect to the Raman spectra and infrared spectroscopy it can be assumed that oxidation to GO was successfully achieved. Selected area electron diffraction (SAED) of GO suggests that the flakes are amorphous, which is likely a consequence of the disruptive nature of bonding caused by the sp^2^ oxygen groups or structural defects. It could be possible that certain areas of the GO flakes are amorphous and other areas possess a degree of crystallinity which could be found by probing several areas across the sample with SAED coupled with TEM (Fig. 2 and 3).

Then, the Zn(ii)-substituted BTSC samples were solubilized in DMSO and sonicated before being combined with GO (2 mg mL^−1^) to create the corresponding dispersions. In parallel, titrations of ZnBTSCs with 2 mg mL^−1^ GO dispersions were performed. The solid-state morphologies of the microcrystalline complexes ZnBTSC in the presence, and absence of GO nanoflakes were investigated by SEM and TEM, and, for the case of ZnBTSC3@GO in the thin film by two-photon fluorescence lifetime microscopy (2P FLIM, ESI†). The nature of the interactions between the GO and ZnBTSCs was investigated in the dispersed phase using spectroscopic methods which were previously used to compare the behaviour of Zn-functionalised porphyrins complexes with flat aromatic surfaces such as GO, and/or carbon nanotubes. As such, UV-Vis spectroscopy as well as two-photon fluorescence lifetime spectroscopies were applied to probe the effect of adding increasing amounts of GO to the ZnBTSC solutions (ESI†).

The changes in UV/Vis spectra were informative as these helped identify the occurrence of charge transfer processes in the dispersed phase. Whilst the corresponding quantum yields were low (as expected for this class of compounds, Table 1),^47,53^ it was observed that addition of GO quenched the fluorescence emission and diminished the lifetime emission of the ZnBTSC, ESI.† This indicated that the formation of GO-Zn(ii) nano-hybrids could be characterised by a charge transfer process, and/or other non-radiative decay pathways (caused by H-bonding, aromatic stacking, Zn–OH, Zn–NH, Zn–DMSO coordination *etc.*). Such possible charge transfer processes were indicated by the occurrence of new peaks in the UV/Vis spectrum of the Zn(ii)@GO hybrids with respect to those of the free complexes. The UV/Vis titration of the asymmetric ZnBTSC complexes with GO were conducted to observe the effect of increasing GO content in solution on the absorption bands characteristic to ZnBTSCs (*λ*_max_ values), and the corresponding change in the GO characteristic absorption band. The experiment required a stock solution of ZnBTSC where 0.2 mL was removed and replaced with 0.2 mL of a stock solution (to maintain constant host concentration). Experiments were carried out in duplicate, from a 2 mg mL^−1^ stock of GO dispersion in DMSO. It is well established that dispersed GO nanoflakes absorb at *ca.* 230 nm and this is dependent on the solvent of choice. Additionally, bands in this region were shown to increase in absorbance with increased concentration of GO.^54,55^ Interestingly new absorption peaks occurred in the range *λ*_max_ 300–400 nm which we assigned to the incorporation of the ZnBTSC chromophores in the GO framework (ESI†). A blue (hypsochromic) shift was observed throughout the titration process, with an increased absorbance which is expected upon increasing the concentration of GO (Fig. 5 and ESI†). The blue shift was combined with a decrease in the *λ*_max_ absorbance value, which corresponds to a higher energy transition: these changes in *λ*_max_ peak could be a result of an increasing level of interaction/binding between the GO and the metal complex which begins level out upon saturation with GO in the final stages of the titration. In addition, the shoulder at *ca.* 300 nm which is observed in the absence of GO reduces, and noticeably broadens, the peak with increased GO concentration. The disappearance of the shoulder seen in UV/Vis spectra has already been documented and assigned to the progressive increase in the level of oxidation of graphene to GO, however this characteristic shoulder also corresponds to the level of GO aggregation and the level of interaction/conjugation between the oxygen-functionalised flakes.^55^

**Table 1:** UV/Vis spectroscopy of ZnBTSCs showing maximum absorbance *λ*_ex_ wavelengths, calculated molar extinction coefficient (*ε*) and quantum yield of fluorescence *φf*

Compound	*λ*_exc_ (nm)	*λ*_em-max_ (nm)	*ε* (L mol^−1^ cm^−1^)	*φf*
ZnBTSC1 (R = Et)	353	527	12 133	0.024
ZnBTSC2 (R = allyl)	335	559	42 657	0.090
ZnBTSC3 (R = Ph)	360	450	13 214	0.028
ZnBTSC4 (R = –CH_2_–o–F–C_6_H_4_)	352 (454)	537 (523)	14 426 (4622)	0.107 (0.045)

UV/Vis titrations involved the aliquots additions from a stock solution (1.15 mM compound ZnBTSC3 in DMSO) with increased absorbance with consecutive titrations (in duplicate) of GO stock solutions, which varied as indicated by the absorbance spectra, as shown in Fig. 5, 7, 8 and ESI†. It was observed that the absorbance at *λ*_max_ with each 0.2 mL aliquot increased linearly with GO content (suggested by the absorbance at *λ* = 258 nm, local maximum for GO). All other ZnBTSC compounds displayed the same trends in UV/Vis spectra however, the shoulder was more prominent in ZnBTSC3 and ZnBTSC4 which may be a consequence of the phenyl rings contained within their structures. The presence of aromatic substituents may also influence the orientation of these complexes onto the GO through the π–π stacking and influence the magnitude of the charge transfer processes between the complex and GO, however a quantitative evaluation was not possible due to the heterogeneous nature of each GO nanoflake. No new absorbance bands were observed in the 500–600 nm range upon increasing the GO content in solution, which may have been observed in case the covalent (additionally to the expected non-covalent) functionalisation of the GO surface occurred.^56^

**Fig. 4:**
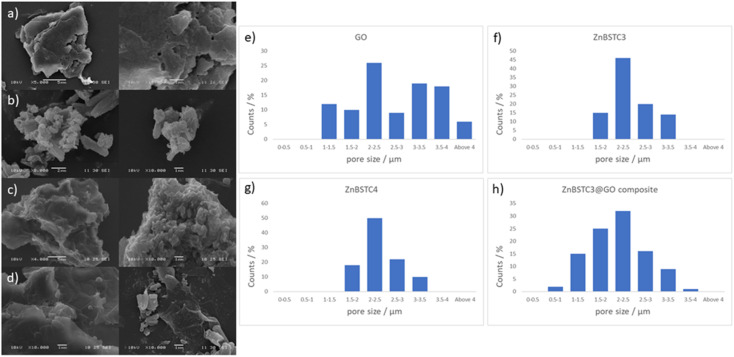
Extensive FESEM imaging whereby solid samples were deposited on HOPG as the carbon support and gold coated, for various batches of (a) GO, (b) ZnBTSC3, (c) ZnBTSC4 and (d) ZnBTSC3@GO with corresponding estimated pore sizes distributions for (e) GO, (f) compound ZnBTSC3 and (g) compound ZnBTSC4 (h) ZnBTSC3@GO hybrid, showing a general trend for the diminishing of pore sizes of GO upon ZnBTSC3 incorporation.

Transmission Electron Microscopy (TEM) and High-Resolution Transmission Electron Microscopy (HR-TEM) were used to obtain direct morphological information of the GO starting material, and TEM combined with EDX characterised the corresponding functionalised GO hybrids. By TEM, small aggregates were identified on the GO flakes, which show differing morphology to the GO (Fig. 2–4, also ESI†). These aggregates are assumed to be due to the ZnBTSC species present either on the surface of aggregated GO or in between GO layers. This could be indicative of some degree of interaction between the two species that coexist in various positions on the GO surface. Contrastingly, the TEM images of compound ZnBTSC4 suggest little to no aggregation of the Zn complexes on the GO flakes. Many nano-dimensional aggregates were observed throughout the lacey carbon grid, however only a few were seen isolated on the GO flakes (Fig. 3 and ESI†).

**Fig. 5:**
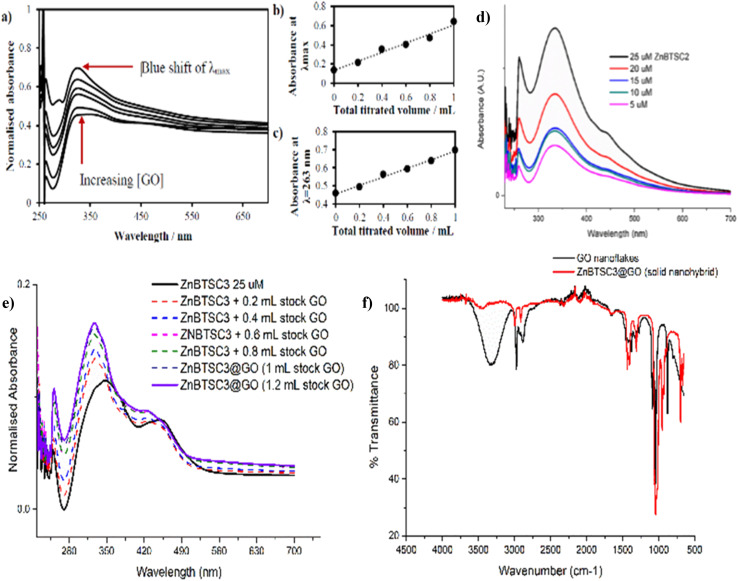
(a) UV/Vis spectroscopy for the titration experiment of ZnBTSC1 with GO. The absorbance of a stock solution (1.15 mM compound ZnBTSC1 in DMSO, solid line) showed increased absorbance with consecutive titrations with a dispersion of GO (dashed line, 2 mg mL^−1^). (b) Absorbance at *λ*_max_ with each 0.2 mL titration aliquot increases linearly with GO content. (c) Absorbance at *λ* = 258 nm (local maxima for GO) with each 0.2 mL titre increases linearly with GO content. Additional titrations with ZnBTSC1–4 and GO are given in ESI.† (d) Self-aggregation behaviour of ZnBTSC2 in DMSO : H_2_O 1 : 1 mixtures. Non-linear behaviour of ZnBTSC2 absorbance. Supramolecular polymerisation may occur, likely driven by (ZnBTSCNH-NH_2_)⋯Zn(BTSC) supramolecular and coordination polymers formation, extended H-bonding, solvent-Zn coordination at 5th coordination site, DMSO-Zn(BTSC) and H_2_O complex formation, and aromatic stacking Zn(BTSC)⋯Zn(BTSC). Hydrophobic interactions in the aqueous DMSO phase cannot be discounted. (e) UV/Vis spectroscopy for the titration experiment of ZnBTSC3 with GO; (f) IR spectroscopy of ZnBTSC3@GO hybrid, compared to GO nanoflakes. Further IR spectra for ZnBTSC complexes (solid state) are given in ESI.†

Scanning electron microscopy (SEM) provided further morphological information on the nanohybrids and when coupled with energy dispersive X-ray spectroscopy (EDX) to provide a means of confirming that such aggregates occur on GO. The SEM images of the ZnBTSC3@GO nanohybrid display differences in morphology to that of GO on its own, and indeed with respect to the morphology, and general pore sizes distribution of free ZnBTSC3, as estimated from SEM using ImageJ software. SEM analysis indicated the occurrence of aggregates of the complexes on the GO flakes, and energy dispersive X-ray spectroscopy confirmed the presence of heavy elements (Zn, S) on the surface of GO, as expected. The addition of GO to ZnBTSCs leads to the formation of aggregates on the surface of the GO flakes, which could also be found in between the layers of GO. In comparison, the features display by GO nanoflakes on its own showed a smoother appearance on the nanoscale and suggestive of layers of GO with folding appearances. SEM and TEMs indicated that different nano-hybrid composites present similar morphology on the nanoscale and therefore is suggested that in each case the ZnBTSC1–4 complexes are incorporated within the GO networks in very similar ways resulting in morphologically, and structurally similar hybrids denoted ZnBTSC1–4@GO.

From a large selection of micrographs corresponding to different batches of GO, ZnBTSC and ZnBTSC@GO complexes including several previously published batches,^57–59^ the pore size of such hybrids was measured directly from SEM images using ImageJ 1.52.^60^

Porosity and morphology investigations of representative batches of ZnBTSC3@GO hybrid were carried out by SEM measurements and compared to the corresponding measurements on free ZnBTSC3 and ZnBTSC4 complexes deposited on highly oriented pyrolytic graphite (HOPG). This was employed as a supporting substrate for these complexes to facilitate the imaging of morphological features of ZnBTSC3 and ZnBTSC4 on flat extended aromatic carbon surfaces. This would minimise errors at the estimation of the pore size for these molecular compounds and facilitate a comparison with GO and rGO pore estimations by SEM. The measuring areas on SEM images were randomly chosen and the pore sizes histograms of the measured results (number of measurements *N* > 100) as presented in Fig. 4.

Fig. 4 suggests that the pore size of pristine GO varies in the 1–4 μm range which did not fit to a normal distribution such as Gaussian, Gauss or Laplace-Gauss. They are in the same order of magnitude with the pore sizes of the thermally reduced GO and porphyrin-rGO hybrids reported previously^57,61^ and which were obtained by BET measurements. Interestingly here the pore size estimations showed a nearly Gaussian distribution with most pores sizes being found below 3 μm. This suggests that the presence of ZnBTSC3 complex in the dispersed phase mixture represents the major process triggering the formation of smooth surfaces (as seen by SEM) and causes the occurrence of small pores with a relatively narrow distribution range size in resulting nano-hybrid. The pore sizes estimated here are a very good match with previously reported values from BET measurements for GO, rGO and porphyrin-rGO systems.^57,58^

Previous studies reporting solvothermal/hydrothermal fabricated graphene 3D structures generally led to a relatively large pore sizes of around 5 μm or above.^62–66^

**Fig. 6:**
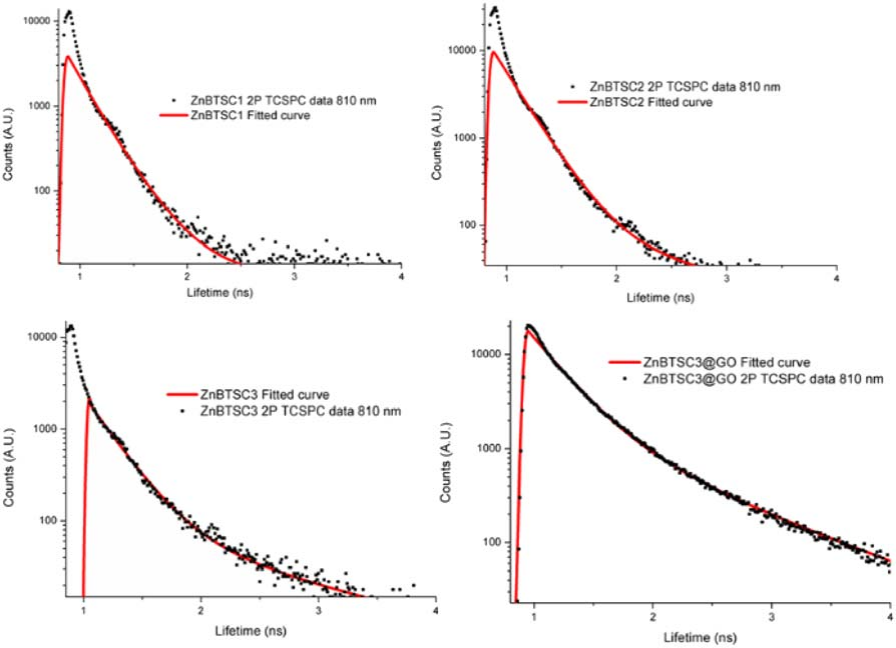
Two photon time-correlated single photon counting (TCSPC) point decays and multiexponential decay fitted data curves for compounds ZnBTSC1–3 in DMSO (10 mM), multiphoton excitation *λ*_ex_ 810 nm and for ZnBTSC3@GO (formed by a 10 mM solution of ZnBTSC3 with 1 mg mL^−1^ dispersion of GO in DMSO).

Considering the striking difference in the hybrid morphology for the morphology of compound ZnBTSC3 when supported onto GO with respect to individual components, it we postulated that the two species (ZnBTSC and GO nanoflakes) that the species remain associated in the dispersed phase. Kinetic stability evaluation of the GO nanocomposites using UV-visible absorption using a 2 mg mL^−1^ concentration of ZnBTSC3@GO hybrid in DMSO were performed. UV-visible spectra of ZnBTSC@GO solutions of DMSO : H_2_O 1 : 1 v/v were prepared and spectra were acquired at 15 min and 24 h. Whilst there were no changes observed in the UV/Vis spectra after 30 min of incubation, a *ca.* 2% change in absorption band intensity was noted at 24 h for ZnBTSC2@GO. The 2-photon fluorescence lifetime spectroscopy was performed DMSO on 10 mM solutions of ZnBTSC1–3, where R = ethyl, allyl or phenyl, and data was fitted following established protocols using multiexponential decay curves in line with previous work on Zn-derivatised bis(thiosemicarbazones)^47^ (see Fig. 6, Table 2 and ESI†). Interestingly, for the ZnBTSC3@GO hybrid, the lifetime characteristics are heavily dominated by the presence of ZnBTSC3, yet with subtle variations in the distribution of lifetime with respect to free ZnBTSC3. All point decay curves for free complexes and for the dispersion of ZnBTSC3 in DMSO strongly indicate formation of highly fluorescent emissive species yet with extremely short lifetime (within instrument response range). Such components characterised by very short lifetime were found also in the thin film lifetime mapping of ZnBTSC3@GO composite and was in line with previous observations on the behaviour of fluorescent supramolecular aggregates in solution and in thin film.^61^

**Fig. 7:**
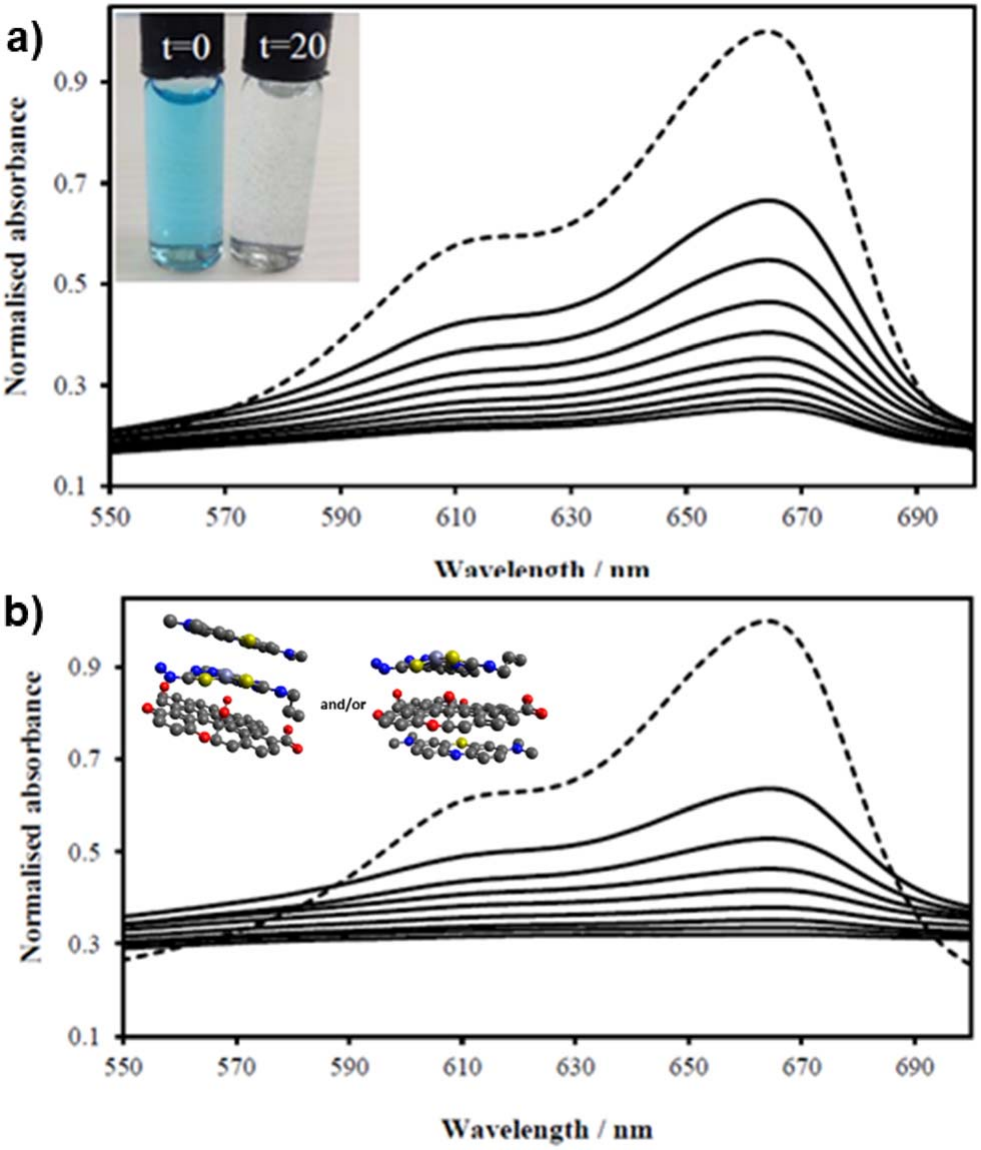
UV/Vis spectra of MB solution (2.68 mM, 200 mL, dotted line, DMSO : H_2_O 1 : 1) and upon addition of (a) GO (8 mg) and (b) compound ZnBTSC2 (8 mg) anchored upon GO (8 mg). Spectra were recorded every 2 minutes (in DMSO : H_2_O 1 : 1 v/v) showing the MB band annihilation (670 nm) without and violet light irradiation (successive solid lines) showing the rapid and progressive loss of MB absorption. Visually, in both cases MB solutions looked identical and fully discolored within 20 min (inset). Possible arrangements postulated for MB on the ZnBTSC2@GO hybrid are included (MM + model) is depicted in ESI.†

**Fig. 8:**
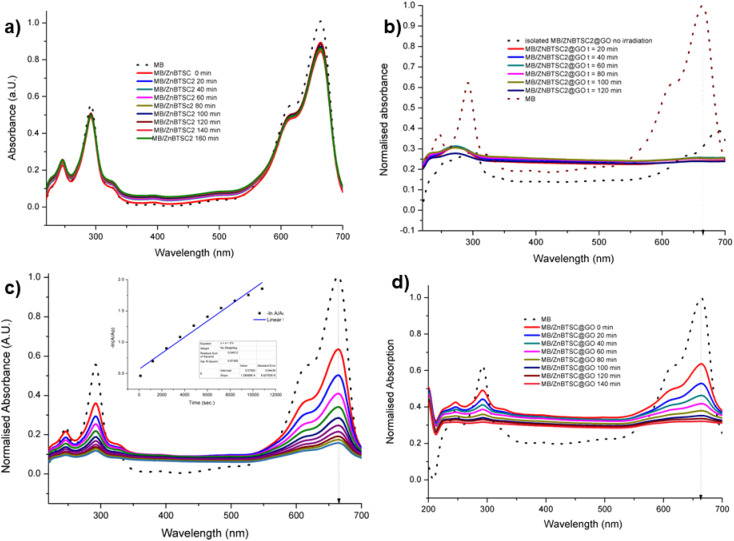
UV vis spectroscopy experiments (a) methylene blue irradiated in the presence of ZnBTSC2 over 2 h with violet light (in DMSO : H_2_O 1 : 1). (b) Methylene blue irradiated in the presence of ZnBTSc2@GO over 2 h. Blue dotted lines represent correspondingly the absorption of the original free MB solution, and of the MB onto ZnBTSC2@GO prior to irradiation. (c) Absorption of MB on GO without irradiation monitored over >3 h, in DMSO : H_2_O; the 24 h trace is given in ESI.† Black line represents free MB under the same conditions. The dispersion of MB onto GO after 24 h, expansion of the normalized absorption axis, is given in ESI;† (d) absorption of MB on ZnBTSC2@GO without irradiation monitored over 2 h. Black line represents free B under the same conditions. Additional spectroscopic details are given in ESI.†

**Table 2:** Fitted parameters for the two-photon excitation time-correlated single photon counting (TCSPC) decay curves at 810 nm excitation and laser power 2 mW (ZnBTSC1, ZnBTSC2 and ZnBTSC3 solutions were 10 mM in DMSO and the ZnBTSC3@GO hybrid was formed by anchoring a 10 mM solution of ZnBTSC3 onto 1 mg mL^−1^ dispersion of GO in DMSO)

TCSPC						
	*χ* ^2^	t_1_ (ps)	a_1_ (%)	t_2_ (ps)	a_2_ (%)	t_m_ (ps)
ZnBTSC1	1.6	185	88.5	237	11.5	201
ZnBTSC2	1.8	216	99	1235	1	227
ZnBTSC3	1.5	232	95	1179	5	279
ZnBTSC3@GO	1.5	328	86	1080	14	435

### Evaluation of the potential for the immobilisation and photochemical degradation of methylene blue by the GO nanohybrids

With the kinetically stable ZnBTSC@GO hybrids in hand, we then focused on evaluating the applicability of these nanohybrids towards addressing challenges posed by organic dyes incorporation and preliminary photocatalysis tests. Earlier work on methylene blue immobilisation considered its simultaneous adsorption onto nanoparticulate materials and conversion and established that its inherent visible light absorption shifts significantly upon methylene blue incorporation onto such supports. Several studies using liquid chromatography-mass spectrometry (LC-MS)^1,67^ suggest that the MB dye degrades photocatalytically by demethylation followed by a multiple step radical process, however these analytical methods are often difficult to deploy in industrial settings or environmentally challenging conditions.

The ZnBTSC2@GO hybrid suspensions in DMSO : H_2_O 1 : 1 mixtures were assessed for their potential to participate in photochemical energy transfer with methylene blue (MB) dye upon irradiation with a UV light source. We followed the behaviour of MB under photocatalytic activation conditions using violet light and, in the presence, and absence of the ZnBTSC2@GO hybrid which comprised the typical asymmetric ZnBTSC complex supramolecularly incorporated within GO sheets. This approach was consistent with previous work on MB decontamination in aqueous media, and, additionally, as a reliable test since MB dye was also commonly used to test photocatalytic ability of nanohybrids.^68–72^ Colorimetric observations showed that upon oxidation, this common dye and notorious pollutant, MB turns from blue to colourless, thus providing a qualitative response on its annihilation and removal from solution phase. We observed a similar feature in the presence of GO or ZnBTSC2@GO even in the absence of violet light irradiation (Fig. 7, 8 and ESI†). In this preliminary test, a dispersion of nanohybrids denoted ZnBTSC2@GO was added to a solution of MB dye and stirred over a period of 2–3 hours under violet light irradiation (*λ* = 360–400 nm). Every 20 minutes, 2 mL aliquots of the solution were removed and the UV/Vis measurements on this aliquot was performed to estimate any changes in the characteristic MB absorption profile over this observation time (up to 180 min). Overviews of the most relevant UV-Vis experiments used to monitor the MB immobilisation are given in Fig. 7 and 8 and further details are given in ESI.† A clear colour change and the associated change in absorption spectra was observed (from blue to colourless, see Fig. 7) and this has also been commonly reported upon photocatalytic degradation of the MB dye, such has been shown for TiO_2_ nano-composites under visible light^73^ as well as other recent reports.^74–76^ The UV/Vis spectrum of MB originally displayed a characteristic absorption peak at *λ*_max_ = 664 nm (in DMSO : MilliQ water 1 : 1), which almost entirely disappeared upon the addition of GO (8 mg/200 mL) even in the absence of UV irradiation. After 20 minutes the solution had qualitatively turned from blue to a colourless solution suggesting an induced change to the MB dye. Additionally, a broad peak corresponding to GO arises between 200 and 300 nm and no absorption bands are observed in the 600–700 nm range after 3 h. Interestingly, in the absence of the [ZnBTSC2]@GO hybrid, the MB alone did not degrade upon irradiation over a 3 hour duration, with no significant changes in colour/UV-Vis spectra (ESI†).

In the presence of ZnBTSC2 (used as a control experiment, in the absence of GO), the effect of up to 3 h irradiation was also limited, with less the 5% changes in the absorption spectra of 664 nm being observed, and virtually no shift in the absorption maxima throughout. Furthermore, by adding the nanohybrid ZnBTSC2@GO (16 mg, 1 : 1 ratio of ZnBTSC : GO by weight/200 mL DMSO) to MB, the solution discoloured much faster, and some precipitation occurred either with or without 3 h UV irradiation. Fig. 8 shows the UV/Vis spectra of MB solution (2.68 mM, 200 mL, dotted line) and upon addition of compound ZnBTSC2 (8 mg) anchored onto GO (8 mg). For this test, absorption spectra were recorded every 2 minutes without UV irradiation (successive solid lines), showing successive diminishing of the MB characteristic band at 664 nm. For processes under 3 h irradiation with ZnBTSC2@GO, no evidence of the original MB absorption could be detected, promoting speculations that conversion to CO_2_/H_2_O (and additional colourless degradation products) occurred over 3 h irradiation.

Possible adsorption mechanisms of MB onto the GO as well as the ZnBTSC2@GO involve electrostatic attraction, hydrogen bonding and aromatic stacking interactions, driven by hydrophobic interactions prior to any catalytic activation. It is likely that the observed enhancement of methylene blue (MB) adsorption occurs through the π–π aromatic stacking interactions between the aromatic ring of MB molecules and the ZnBTSC2@GO hybrid, resulting in non-covalent adsorption, in line with other studies that reported similar features when ZnO or WO_3_ were used in MB degradation studies.^77,78^

Recent studies highlight the unique physical and photoelectric properties of transition metal functionalised bis(thiosemicarbazones), which positioned them as novel and efficient photocatalysts for organic dyes under similar conditions.^79^ A possible reason is that the valence band of these thiol-substituted metal complexes exhibits a more negative energy level relative to the O 2p orbitals, enabling smaller band gaps and therefore increased susceptibility to visible light.

In line with previously reported systems,^79^ in the presence of violet light irradiation, the aqueous solution containing the ZnBTSC2@GO photocatalyst and MB in presence of DMSO and H_2_O undergoes photochemical processes, generating electron–hole pairs (e^−^–h^+^). It has been proposed earlier^79^ that the photothermal effect facilitates the migration of electrons from the valence band to the conduction band, leaving holes in the valence band. This mechanism could potentially also significantly diminish the likelihood of combined photo-excited electrons and holes in the ZnBTSC2@GO nanohybrid employed as a photocatalyst. Previous reports also stated that an abundance of photo-excited holes is retained^76,80,81^ on nanoparticulate supports, actively participating in the oxidation of MB, and here, the GO present in the ZnBTSC2@GO is therefore likely to amplify the photocatalytic activity of the apparently weak catalyst ZnBTSC2 when acting alone. Furthermore, the surplus photo-generated holes could react with adsorbed water within the GO nanoflake layers to produce hydroxyl radicals (OH) (as suggested by Mukhtar *et al.*),^82^ promoting the decomposition of MB. Additionally, the oxygen adsorbed on the MB surface, itself held in the proximity of the GO nanoflake surface accepts electrons, forming superoxide radical anions, and ultimately leading to OH formation upon protonation: such reactive oxygen species were shown to play pivotal roles in the mechanistic understanding of the degradation of MB under light irradiation and were extensively studied for other nanoparticulate systems.^76,80,81^ This interplay of light-induced electrons, holes, hydroxyl radicals, and superoxide free radicals engages in redox reactions with target pollutants, which could resulting in the advanced degradation of the targeted dye (here MB, in DMSO : H_2_O) into H_2_O and CO_2_ within 2–3 h irradiation with violet light. A close inspection at the UV-Vis spectroscopy and estimation of the reaction rates [ESI†] by methods in line with recent research on the immobilisation of MB on a range of nanomolecular systems^77,78^ seems to indicate that the process may not essentially need to be photocatalytically driven to effectively remove this toxic dye from aqueous environment but such sustainable processes could well be shortened in certain practical applications. Here, the need for photocatalytic conversion of MB was shown to be conveniently superseded by the rather effective adsorption equilibrium process mediated by graphene oxide.

### Evaluation of the potential for the immobilisation of glycosyl coumarin onto GO nanocomposites

With the new ZnBTSC@GO nanocomposites in hand, we also proceeded to expand upon the dye adsorbtion protocols and investigate other dye-like organic molecules in aqueous environments. The use of graphene oxide as a simultaneous carrier for glycosyl coumarin (4) and ZnBTSC3 and corresponding nano-composite formation was also investigated. To address limitations of the state-of-the-art in the supramolecular chemistry of graphene oxide, we developed and reported hereby on our new approach and underlining synthetic method, *e.g.* to isolate and characterize a water-soluble coumarin derivative, to probe the generality of the GO scaffolds in this dye-removal protocols.

**Scheme 1:**
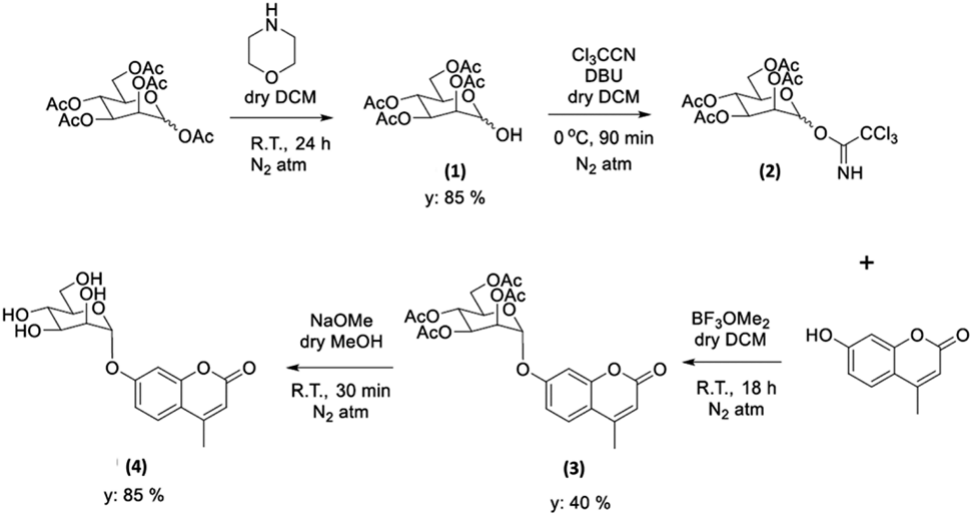
Schematic representation of the formation of the protected and deprotected GC functional coumarin dyes derivatives, compounds 3 and 4, respectively (see ESI† for synthetic details, full characterisation, and single crystals X-ray diffraction data).

**Fig. 9:**
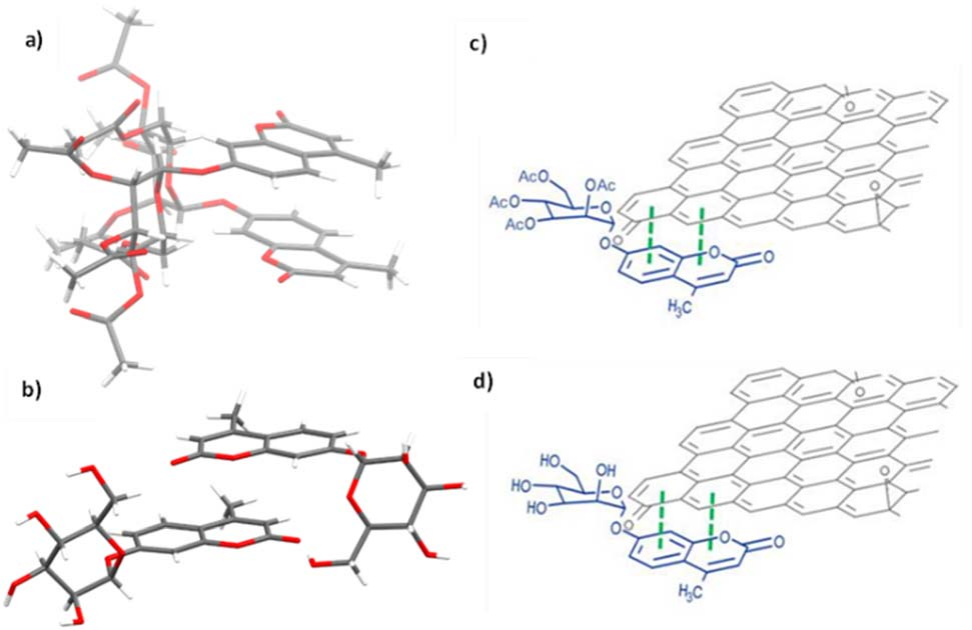
Single crystal X-ray structure analysis: (a) molecular structure of compound 3 (AcGC) showing the presence of two nearly identical molecules in the asymmetric unit, non-covalently bound to each other through an interplay of H-bonding and aromatic stacking. (b) Content of asymmetric unit showing the presence of two almost identical units of compound 4 (GC) in a head-to-tail aromatic stacked configuration and associated through further H-bonding interactions. Molecular parameters from the solid-state are given in the ESI.† Atom colours: C: grey, O: red, H: white. (c) and (d): schematic representations showing how compounds 3 and 4 (denoted AcGC and GC, respectively) may be non-covalently bound to GO nanosheets, by an analogy with the model depicted at the MB immobilisation by GO (Fig. 1, previous work) forming hybrids AcGC@GO (c) and GC@GO (d), respectively.

Glycosyl coumarin derivatives were first reported by Supuran *et al.*^83^ The study suggested that a selected deprotection of α-d-mannose pentaacetate with morpholine leads to a tetraacetyl-d-mannopyranose (1). An adapted method, applied hereby, involved the treatment of compound (1) with trichloroacetonitrile affords the corresponding tetra-*O*-acetyl-d-mannopyranose trichloroacetamidate (2) (Scheme 1 and ESI†). Then, coupling of the obtained intermediate (2) with 4-methylumbelliferone gives a methylumbelliferyltetraacetyl-d-mannopyranose (denoted AcGC, compound 3) and finally, hydrolysis of the acetyl groups results in the desired compound, methylumbelliferyl-d-mannopyranose (denoted GC, compound 4) (Scheme 1). We streamlined and optimised this process and characterized the relevant intermediates fully including by single crystals X-ray diffraction (Fig. 9(a) and (b) and ESI,† CCDC numbers 2301154 and 2301155).

We hypothesised that glycosyl coumarin (GC, compound 4) may become non-covalently immobilised to the graphene sheets by virtue of π–π stacks formation, given its flat and aromatic structure and these aromatic interactions may well be reinforced by H-bonding interactions. The molecular species GC and its acetyl-protected congener AcGC were considered as suitable models fit for the exploration of aromatic interactions between fluorogenic dyes and graphene-oxide based nanohybrids as they were fully characterised structurally hereby, by single crystals X-ray diffraction. By virtue of their flat, aromatic, and oxygen-rich functionalities, we predicted their compatibility with the extensive domains of sp^2^-bonded carbon atoms of GO in terms of the potential to form *via* π–π aromatic stacking and give rise to supramolecular nanohybrids. To explore the interactions between GC and GO, a suspension of GO nanocomposites diluted in DMSO was mixed over five minutes with a GC (4) solution in DMSO to afford the desired nanocomposite (GC@GO, Fig. 9d).

The chemical composition of GC (4) consists exclusively of light elements (C, O, and N, which are notoriously deemed weak scatterers for electron diffraction microscopies) therefore the use of techniques such as TEM and EDX, which are commonly applied for the analysis of GO nanocomposites (formed by C and O), was not sufficiently informative for the deeper analysis of this hybrid species. Therefore, spectroscopic investigation in solution was applied to demonstrate if there was a possible binding between GO and GC (4). A continuous variation method (Job's plot method, one of the most popular methods to determine the stoichiometry of a binding event using common spectroscopic methods) was applied to monitor any possible donor–acceptor exchanges in solution phase.^6,84,85^ The structural and molecular analysis of supramolecular complexes incorporating GO remain to be understood.^6^ The molecular weight of the disordered bulk material known as GO remains elusive, although several different modelling studies were performed on this functional material. Each GO nanoflake presents highly disordered structures with inhomogeneous morphologies, therefore a precise determination of binding stoichiometry to small fluorogenic molecules was not possible. However, Job's method could be used in an estimative approach, as a tool to evidence the differentiation in the emission intensity due to the quenching of the fluorescence emission upon titrations. It is known that GO and related species can be directly coated on sugars through their hydroxyl groups *via* hydrogen bonds, so by analogy, we proposed accordingly that GC and to a lesser extent AcGC would form H-bonding interactions to GO too.^86^ To explore whether π–π interactions between GC (4) and GO's surface may occur, an initial titration experiment was carried out using AcGC (3) where the hydroxyl groups are protected, in addition to GC (4).

This control experiment was chosen to determine the fluorescence emission that corresponds to coumarin unit using different fluorescence excitation maxima. In this context, it is important to take on consideration that previous studies have suggested that the presence of hydroxyl groups in a coumarin moiety could be a deciding factor of its fluorescence efficiency and total immobilisation onto GO.^87^ Multiple functional groups gave rise to a range of fluorescence maxima of glycosyl coumarin which as observed during these titration experiments. For instance, the fluorescence emission of coumarin backbone can be detected at 376 nm when excited at 310 nm, however when functionalities such as hydroxyl groups are present, the fluorescence emission was observed at 460 nm instead.^88^

**Fig. 10:**
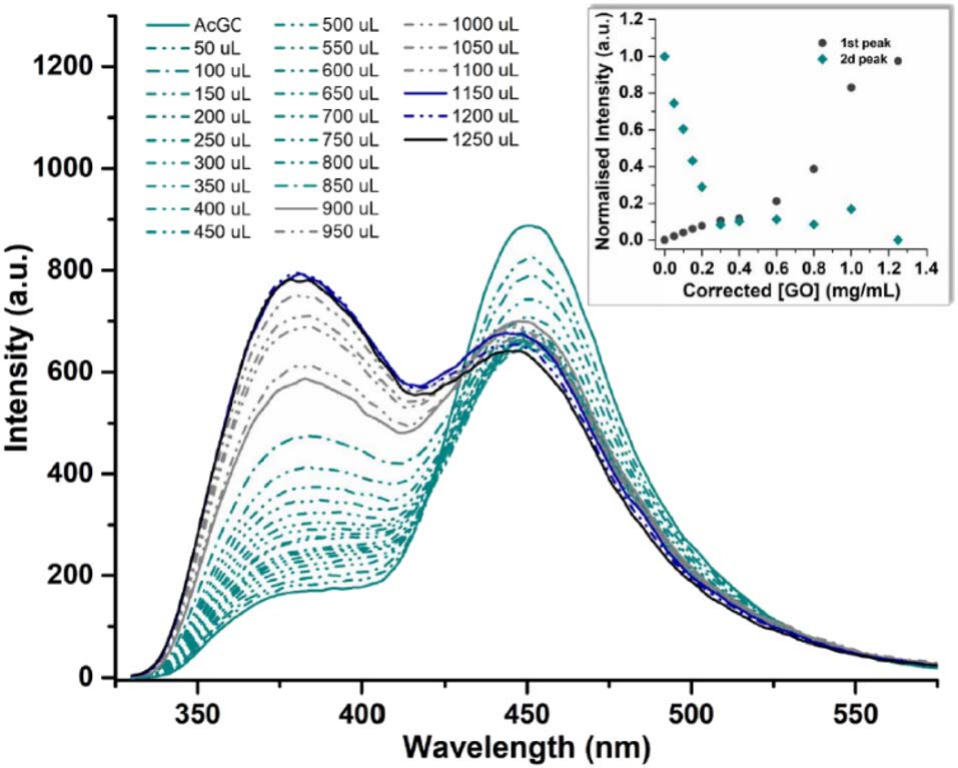
Fluorescence emission quenching in a solution of compound 3 (AcGC) (0.3 mM) during titration of graphene oxide (GO) nanocomposite (mg mL^−1^) in DMSO. The inset shows the normalised fluorescence emission intensity of the two highest peaks at the relevant GO concentration.

In a typical titration experiment exploring the fluorescence spectroscopy a GO dispersion with the acetyl glycosyl coumarin (AcGC) the standard method whereby aliquots of a specific amount of guest solution were added gradually to a host solution (keeping the overall concentration constant) was applied, and the fluorescence emission was measured by a photo-counting steady state method. Here, a solution of compound 3 (AcGC) (1 mM) and a stock dispersion of GO nanocomposite (1 mg mL^−1^) were prepared in DMSO. Then the solution of compound 3 (AcGC) was excited at 310 nm and the emission spectrum was measured in a 330–600 nm range. The dispersion of GO nanocomposite (1 mg mL^−1^, considered the ‘guest’ in the supramolecular complex formation) was added gradually (50 μL) into 1.2 mL of compound 3 (AcGC) solution (considered as the ‘host’) and the total concentration of [GO + compound 3] for each fluorescence measurement was kept constant (Fig. 10) to probe whether GO induced the quenching of the fluorescence in compound 3 (AcGC). The resulting dispersions were mixed well before each measurement and after addition of an aliquot of ‘guest’ and any excess GO was allowed to settle before each measurement for up to 5 minutes at the room temperature.

**Scheme 2:**
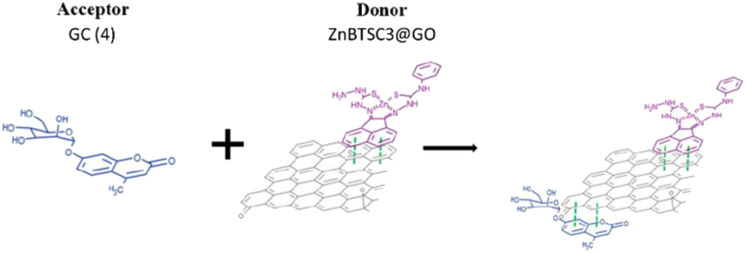
Schematic representation of the formation of complex GC@ZnBTSC3@GO; where the compound 4 (GC) and the ZnBTSC3 asymmetric complex are non-covalently attached to the planar graphitic surface of the nanocomposite.

As shown in Fig. 10, the fluorescence intensity corresponding to compound 3 (AcGC) is reduced upon addition of GO suspension. This reduction could be a result of the static and dynamic quenching due to the complex aggregation^89^ in the dispersed phase due to π–π interactions, as represented in Scheme 2.

**Fig. 11:**
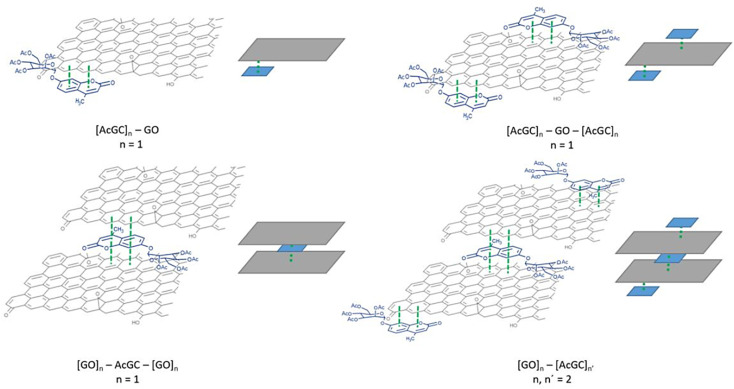
Schematic illustration of the different species postulated to result from the complexation of GO with compound 5 (AcGC). In this figure, the simplest version of these species is illustrated, and *n* = 1–2.

Titration experiments indicated that, in line with previous reports modelling binding interactions between species including on graphene oxides, a variety of arrangements may occur, *e.g.* as reported by Jabbari-Farouji *et al.*^90^ Spontaneous self-assembled systems whereby two species could “recognise” each other and bind in the dispersed phase could occur hereby, as indicated by Fig. 10 (inset graph). This titration experiment suggests that the nature of the complexation interactions between the two systems analysed hereby (AcGC and GO) in the dispersed phase is likely to be more complex than implied by the idealised molecular models depicted in Fig. 11 and Scheme 2. These complex solution equilibria dominated by aggregation interaction events may well occur in a concerted way and result in the partial increase of the intensity to the absrobtion maxima corresponding to AcGC and in a decrease of absorption bands in the spectra of the formed complex (blue and black lines from Fig. 10). It is also likely that other GO nanocomposites will form further supramolecular oligomers by stacking resulting in sandwich-like complexes with highly irregular geometries and stoichiometries in dispersed phase, and likely under thermodynamic exchange.

Fig. 11 shows a simplified view of some of the possible species that could be formed, and these are represented schematically to indicate the possibility of non-covalent binding interactions occurring between GO and AcGC (3) functional groups. However, this experimental approach showed that fluorescence spectroscopy could shed light in the nature of the assembly processes only if a further simplified model system was employed. Therefore, a better understanding of the strength of such interactions in solution has been sought by employing the conditions developed above in a model titration investigation: a fluorescence titration of coronene (instead of GO) was carried out and an equimolar mixture of compound 4 (GC) with coronene was also analysed, to estimate the possible binding interactions occurring between them. Since the binding stoichiometry could not be determined for GO nanocomposites, to examine the possible binding a theoretical model was used and the estimation of the binding stoichiometry between GC (4) and coronene was pursued, as described below.

Coronene, C_24_H_12_, a member of the polycyclic aromatic hydrocarbon (PAH) family, is a planar molecule consisting of seven fused benzene rings that has been extensively used as a model for graphene or graphene oxide systems in order to evaluate their supramolecular interactions in solution phase.^1,91^ In the solid state, this yellow crystalline material is mainly soluble in benzene or toluene, also sparingly in other organic solvents.^1,9^ This system, has also been employed as a theoretical model to study either graphene's oxidative process or internal aromatic interactions with graphene.^1,91,92^ Since both the structure and the molecular weight of coronene are known, this is facilitating kinetic and thermodynamic studies of the supramolecular association between graphene nanosheets and chromophores. In our hands, the solution of compound 4 (0.125 mM) was excited at 310 nm and the emission spectrum was measured from 330 nm to 600 nm. Gradual addition of the ‘guest’ solution (0.125 mM of coronene) to the ‘host’ solution (GC, compound 4) then followed, as described in the experimental section, till the ratio of GO to compound 4 (GC) was slightly more than 1 : 1.

The resulting saturated suspension was well-mixed before and after addition of each guest aliquot. The intrinsic fluorescence emission of compound 4 (GC) was quenched upon addition of coronene suspension and a notable shift in emission maxima was noticed, and its intensity was subsequently observed to be later quenched as the titration experiment progressed. The reduction of the fluorescence emission intensity of 4 indicated the possible complexation of the two molecules and the quenching of the new fluorescence band suggests that the coronene molecule is likely to be bound with the initially formed complex resulting in a sandwich-like complex, with either coronene or 4 (GC) being in the middle or a complex with dimer-like species. As also suggested by Farouji *et al.*,^90^ it is then very difficult to predict all these possible structural arrangements of the building blocks within the nanohybrid, for simplicity the discussion is limited to the association constant for the 1 : 1 complexation which appeared to be the most likely variant. However, we attempted to model the titration data according to a 2 : 1 binding system, as discussed below.

The decrease in the fluorescence absorption of compound 4 (GC) was monitored and a new emission maximum resulted upon the progressive increase of coronene's concentration. The data were collected, analysed by MATLAB m-files^93^ and fitted for 1 : 1, 1 : 2, 2 : 1 C_24_H_12_ : GC binding isotherms. This software resulted in *K*_a_ (eq. 1) and statistical parameters such as standard error of estimated data (SE_y_) and covariance of fitting (Cov_f_). Binding constants and statistical parameters for compound 4 (GC) are reported in Table 3.

**Table 3:** Table presenting the binding constant *K*_a_^2:1^ (M^−1^), for the interactions between GC (compound 4) and coronene, as a model complex formed by aromatic interactions. The standard error of estimated data (SE) and the covariance of fit (Cov_f_) calculated according to MATLAB m-files^93^

Compound	*K*_a_^2:1^	SE_y_	Cov_f_
GC (4)	1.18594 × 10^12^ M^−1^	0.051025667	0.028747143

Here, binding constants are described as *K*_*m*:*n*_ where *m* and *n* are integers. A complex where *m* or *n* is greater than 1 describes a system where either the host or guest possesses more than one binding site. Despite repeated attempts to adjust modelling parameters, to 1 : 1 and 1 : 2 C_24_H_12_ : 4 simulations gave physically non-sensible results suggesting that these models are not favoured systems for the C_24_H_12_ : GC complexes, and 2 : 1 is more likely to occur. The 2 : 1 C_24_H_12_ : 16 isotherm fitting reveals much lower values of *K*_a1_^2:1^ for GC in comparison to *K*_a2_^2:1^ (*K*_a2_^2:1^ = 1.18594 × 10^12^ M^−1^). These values suggest that during the second binding event more complicated species are assembled (Fig. 10, the values between 0.4 and 0.8). All the different equilibriums that may be formed due to such interactions are very difficult to predict.

Based on the previous results indicating that the asymmetric metal complexes can be anchored to the GO nanocomposites, we examined the possibility of the non-covalent binding of 4 (GC) and ZnBTSC3 on the graphitic surface. This was tested in a similar manner as the conjugation of compound 3 (AcGC) on the GO nano-composites.

More specifically the ZnBTSC3@GO hybrid (considered a ‘donor’) was incorporated and effectively bound with the solubilised 4 (GC) (‘acceptor’ molecules) (Scheme 2). A solution of 4 (GC) (1 mM in DMSO) was excited at 310 nm and the emission spectrum was measured from 330 nm to 800 nm. Then a suspension of nanocomposite ZnBTSC3@GO (1 mg mL^−1^ in DMSO, considered the ‘guest’) was added gradually into the 4 (GC) solution (considered the ‘host’) and fluorescence spectra were measured to examine the potential of complex ZnBTSC3@GO to interact with, and therefore quench the fluorescence emission of compound 4 (GC) in the dispersed phase.

**Fig. 12:**
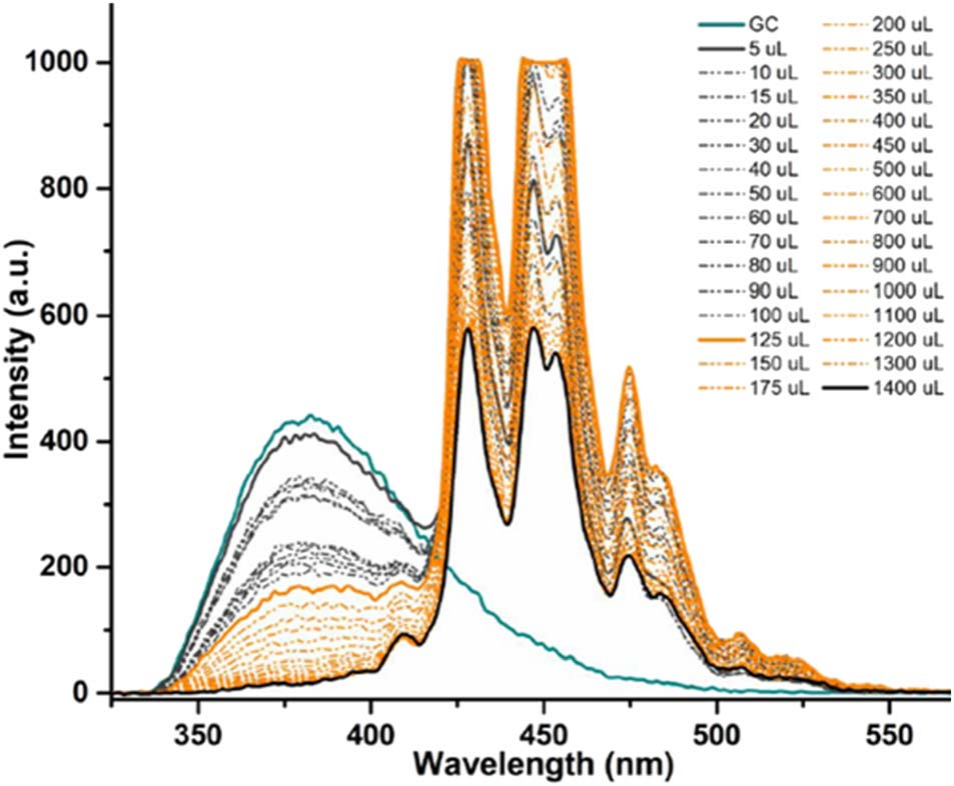
Fluorescence emission quenching in a solution of compound 4 (GC) (0.125 mM) during titration with coronene (0.125 mM) in DMSO, for model binding studies.

**Fig. 13:**
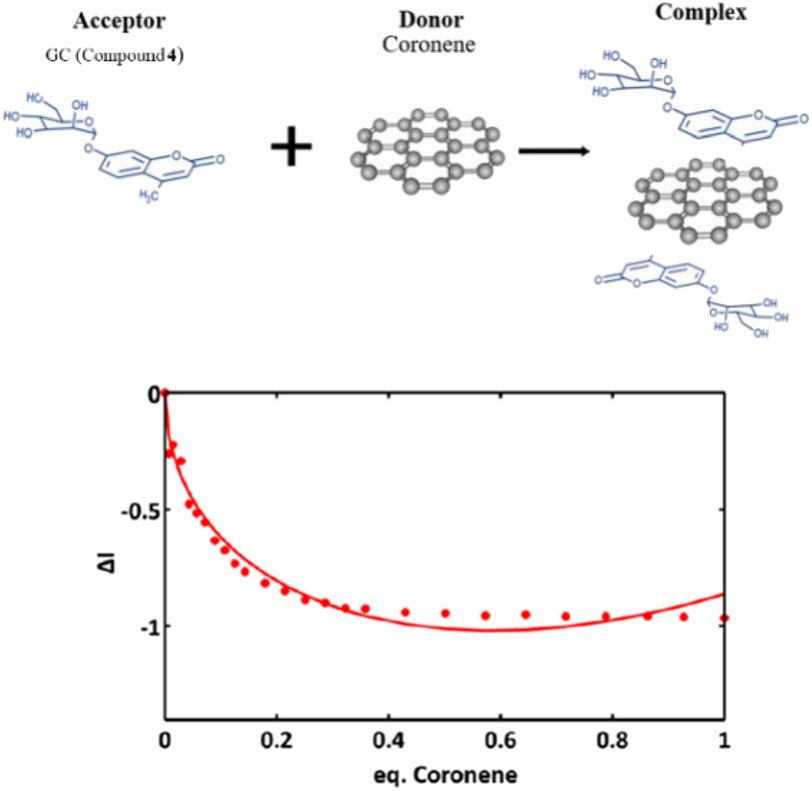
Experimental data and fitting curve (line) of a 2 : 1 isotherm binding model at the relative compound 4 : coronene ratio.

**Fig. 14:**
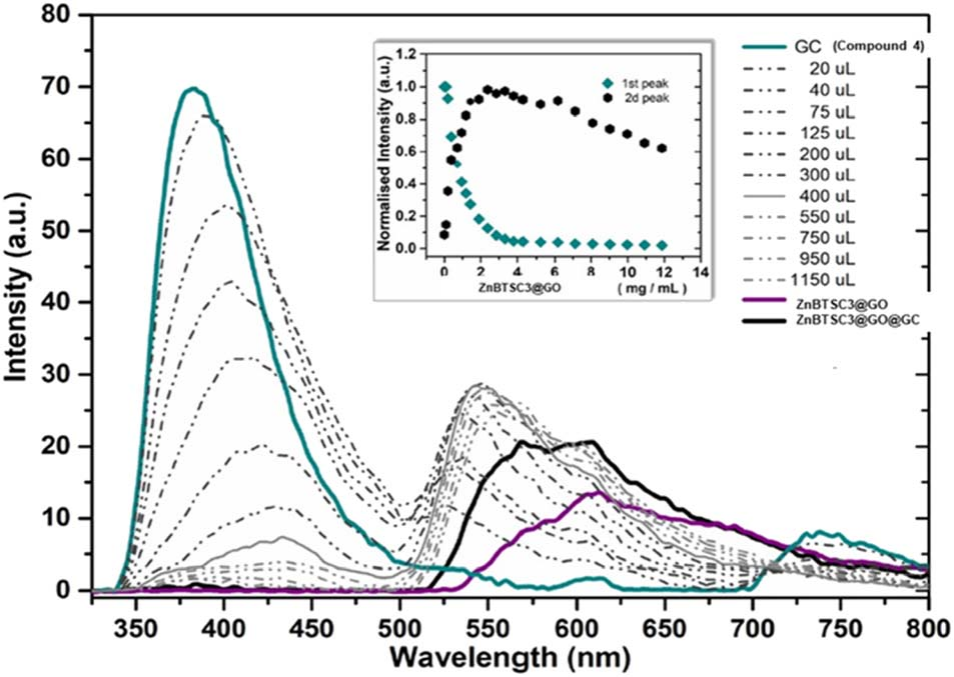
Fluorescence emission quenching in a solution of GC (compound 4 0.3 mM in DMSO) during titration of ZnBTSC3@GO nanocomposite (1 mg mL^−1^) in DMSO. The inset shows the normalised fluorescence emission intensity of the two highest peaks at the relevant concentration of ZnBTSC3@GO.

As inferred from the titration experiment with spectra given in Fig. 14, the emission intensity that corresponds to compound 4 (GC) (blue line) was reduced upon addition of the complex ZnBTSC3@GO suspension and a new fluorescence maximum was observed. Some of the proposed possible arrangements of possible species formed due to the non-covalent binding between complex ZnBTSC3@GO and compound 4 (GC) are illustrated in Fig. 9, 11, 13, Scheme 2 and ESI.† The maximum fluorescence intensity of this emission band (dark grey line) does not emit at the same wavelength as the complex ZnBTSC3@GO which could suggest the complexation of the two molecules is forming a range of different species Fig. 11, 13 and ESI.† It is important to note that as the titration proceeds, this new emission band diminished indicating fluorescence quenching, which seems to suggest a rearrangement of the formed complex GC@ZnBTSC3@GO. The inset graph of Fig. 14 depicts the normalised intensity of both emission bands at the relevant complex ZnBTSC3@GO concentration and implies a relation between the increasing fluorescence intensity of the one emission band and the decreasing fluorescence intensity of the other. Additionally, data presented in Fig. 10, 12 and 14 appear to support this hypothesis: the graphical representation of the data emerging from titration experiments presents the normalised ratio between the initial fluorescence absorption of each peak to the partial intensity of it at the relevant complex (*e.g.* ZnBTSC3@GO) concentration and suggests that the second emission band only increases its fluorescence intensity after the quenching of the first one. These results suggest that the formed hybrid has a significantly more complex supramolecular structure than suggested in Scheme 2. These experiments show that established multistep synthesis for the glucose-coumarin dyes can lead to new and rationally designed nanohybrids of these fluorogenic coumarin conjugates. Titrations with GO followed by UV and fluorescence spectroscopy compared to the findings where the coronene was used as a simple model for the aromatic domains of GO gave rise to interesting findings. We discovered evidence of strong binding interactions in obtained in aqueous organic solutions between the dyes and the carbonaceous supports.

## Conclusions

In conclusion, we recognise and report hereby that novel Zn(ii) BTSCs bind in aggregates on the GO surface, presumably at the GO defects/oxygenated functional sites. These nanohybrids provide rapid and spontaneous adsorption of organic dyes such as methylene blue or and functional coumarins and can be generalized to other water-soluble dyes. In turn these pollutants could then be recovered as GO conjugates consisting of nanohybrids held together by supramolecular interactions. This approach provides an easy route to the subsequent separation and removal of dyes and pollutants. Furthermore, a new synthetic protocol for new water-soluble dye conjugates may lead to discrete, well-understood bioconjugates and functional probes that open opportunities for new imaging chemistry and multimodality imaging applications. The findings described here are of relevance in the efforts to improve the development of robust and scalable separation methodologies for dyes and pollutants using simple graphene oxide-based supports. Due to the complexity and inhomogeneity of GO structure, these material and related hybrids represent remarkable challenges for detailed chemical investigations. We applied hereby methods specific for coordination and supramolecular chemistry to shed light onto fundamental materials properties of GO and its behaviour in donor–acceptor interactions. The observations fit well with the concept of aromatic stacking, and binding of fluorogenic molecules involved through additional H-bonding should not be discounted under these experimental conditions. We anticipate that this study will advance the state-of-the-art in understanding of GO as a molecular host, and the hybrid systems reported hereby could be further utilised by the nanomaterials community in various applications, including environmental remediation and photocatalysis.

## Experimental section

### General experimental methods

All reactions involving air- or moisture-sensitive reagents or in-termediates were carried out under anhydrous conditions and nitrogen atmosphere, using standard Schlenk techniques, unless otherwise stated. Reagents and solvents were obtained from Aldrich Chemical Co. (Gillingham, UK), Fluoro Chem (Hadfield, UK) and Fisher (Acros; Geel Belgium) and used without further purification unless otherwise stated. Solvents were reagent or HPLC grade obtained from Aldrich Chemical Co. (Gillingham, UK) or VWR (Radnor, PA, USA). Water was obtained from a Millipore Milli-Q purification system and anhydrous solvents from a PS-400-7 Innovative technologies SPS system.

Microwave reactions were conducted in a Biotage (Uppsala, Sweden) Initiator 2.5 reactor (o-450 depending on T) in stirred capped vials. The reaction mixtures were pre-stirred for 30 s and heated to the desired temperature by applying maximum power of 400 W that was reduced and kept constant once the target temperature was reached.

Thin layer chromatography (TLC) was carried out on Merck silica gel 60 F254 analytical plates (Matrix silica gel with aluminium support and fluorescent indicator 254 nm, 0.2 mm thickness) and visualized either by ultraviolet (UV) fluorescence (*λ* = 254, 366 nm), by charring with 10% KMnO_4_ in 1 M H_2_SO_4_ or by charring with 5% Na_2_SO_4_ in EtOH. The elution conditions for TLC varied and are quoted for each compound. NMR spectrometry was acquired using either a Bruker (Banner Lane, UK) Advance NMR spectrometer or a 500 MHz Agilent automated system. Bruker and Agilent 500 Spectra operated at 500 MHz for ^1^H NMR, at 125 MHz for ^13^C NMR and at 500 MHz for ^19^F. ^1^H, ^13^C and ^19^F chemical shifts are referenced to tetramethylsilane (TMS). Coupling constants (J) are reported in hertz (Hz) with a possible discrepancy ≥0.2 Hz. Chemical shifts *δ* are reported in ppm. Chemical shift of solvent residues were identified as follows: CDCl_3_: ^1^H, *δ* = 7.26, 13C, *δ* = 77.0; -DMSO-d_6_: ^1^H, *δ* = 2.50; 13C, *δ* = 39.5; D2O: ^1^H, *δ* = 4.79. Peak multiplicities are given as follows: s, singlet; d, doublet; t, triplet; q, quartet; m, multiplet; appd, apparent doublet; appt, apparent triplet; appq, apparent quartet; br, broad.

Accurate Mass Spectrometry was carried out in EPSRC national mass spectroscopy center of Swansea University, U.K, different techniques were used such as MALDI, ESI† and EI.

Analytical phase/reverse-phase was performed either on a Dionex Ultimate 3000 series HPLC system (Sunnyvale, California.) equipped with a UV-Vis diode array detector (measuring at eight wavelengths; 200–800 nm), using a Phenomenex Gemini (Macclesfield, UK) water C18 column (250 mm × 4.6 mm, 110 A) at a flow rate of 0.8 mL min^−1^. The gradient elution was 0.1% TFA Milli-Q water as solvent A and 0.1% TFA MeCN as solvent B. A reverse gradient was applied starting with A at 95%, going up to 5% A at 7.5 minutes, isocratic until 15 minutes and gradient until 95% A, then hold to 18 min (method A). Or in an Agilent 1100 series HPLC system (Agilent Technologies, Stockport, UK) equipped with a UV detector (254 nm) and a Lab-Logic Flow-count radio-detector, using a Phenomenex Gemini (Macclesfield, UK) water C18 column (250 mm × 4.6 mm, 110 A) and a Laura 3 software (LabLogic, Sheffield, UK) at a flow rate of 1 mL min^−1^. The gradient elution was 0.1% TFA Milli-Q water as solvent A and 0.1% TFA MeCN as solvent B. A reverse gradient was applied starting with A at 95%, going up to 5% A at 7.5 minutes, isocratic until 15 minutes and gradient until 95% A, then hold to 18 min (method B).

IR Spectroscopy was carried out on a PerkinElmer (Waltham, Massachusetts) Frontier FTIR machine in ATR mode.

UV-Vis spectroscopy was performed in 1 cm quartz cuvettes in a PerkinElmer (Waltham, Massachusetts) Lambda 35 UV-Vis spectrometer controlled by UV-Winlab software.

Single-photon fluorescence spectroscopy was performed in 1 cm quartz cuvettes in a PerkinElmer (Waltham, Massachusetts) LS55 luminescence spectrometer controlled by FL-Winlab 4.0 software. The two-photon investigations performed at the Rutherford Appleton Laboratory following established protocols with other bisthiosemicarbazone metal complexes using Mira titanium sapphire laser (Coherent Lasers Ltd), and the equipment and experimental setup described in previous work.^94^

The commercial dye Methylene Blue (MB, empirical formula C_16_H_18_ClN_3_S·3H_2_O, Merck) was used as received. Compound ZnBTSC3 was synthesised according to an earlier reported protocol^59^ and further details for the characterisation of this batch are given in ESI.† Full details for the synthesis and characterisation of the novel asymmetric coordination compounds denoted ZnBTSC1, ZnBTSC2 and ZnBTSC4 also for functional coumarins compounds 3 and 4 are given in ESI.†

### Single crystal X-ray diffraction crystallography

The selected crystal was mounted onto a goniometer head and cooled to 150 K with an Oxford Cryosystem. Intensity data for compounds 3 and 4 were collected on a SuperNova, Dual, Cu at zero, EosS2 using a Cu microfocus source (*λ* = 1.54184) Å. Unit cell determination, data collection, data reduction and a symmetry-related (multi-scan) absorption correction were performed using the CrysAlisPro software.^95^ The structure of 3 was solved with SUPERFLIP and of 4 was solved with SHELXT. The structures were then refined by a full-matrix least-squares procedure based on F^2^ (Shelxl-2019/2).^96,97^ All non-hydrogen atoms were refined anisotropically. Hydrogen atoms were placed onto calculated positions and refined using a riding model. Additional programmes used for analysing data and their graphical manipulation included SHELXle^98^ and Mercury.^99^

Final cif structures were deposited *via* the joint CCDC/FIZ Karlsruhe deposition service. The data have been assigned the deposition numbers 2301154 and 2301155.

### Methylene blue adsorbtion, immobilisation and degradation tests

The methylene blue adsorbtion tests were carried out using an adapted protocol from ref. 43.^43^

For the photocatalytic irradiation experiment, the solid hybrid ZnBTSC2@ GO were added to a solution of MB dye, sonicated for 2 minutes to form a dispersion then stirred over a period of 3 hours with violet light irradiation (*λ* = 360–400 nm approximately). The as-prepared nanocomposites contained 8 mg of ZnBTSC2 anchored onto 8 mg GO and were added into a dye solution (2.68 mM, 200 mL). Before the photocatalysis tests, mixtures of MB and ZnBTSC2@GO were ultrasonicated for 2 min at the room temperature. Experiments were run in duplicate. Every 20 minutes 2 mL of the solution was removed for UV/Vis measurements to determine any changes in spectra over time and irradiation experiment was carried out up to 3 h. However, after 20 minutes of irradiation the solution had already qualitatively turned from blue to colourless solution suggesting an induced change to the nature of the MB dye chromophore. UV-Vis spectroscopy showed a broad peak corresponding to GO arises between 200 and 300 nm and no absorption peaks due to MB (expected at 664 maxima) were observed in the 600–700 nm range. The concentrations of residual dye in the DMSO : H_2_O 1 : 1 solution was tested at different time intervals over 3 hours then at 24 h and compared with control experiments. A further irradiation experiment was carried out using MB alone (2.68 mM, 200 mL DMSO : H_2_O) and a MB and ZnBTSC2 solution containing 2.68 mM MB and ZnBTSC2 added as a solid (8 mg) in 200 mL DMSO : H_2_O.

Further experiments were conducted as control measures monitoring the UV-Vis spectroscopy of MB, MB in presence of GO, MB in presence of ZnBTSC2, with and without UV irradiation. As expected the MB did not show significant changes in colour/UV-Vis spectra upon irradiation with violet light over 3 h. For control experiments with MB (2.68 mM, 200 mL) and GO alone, upon addition of GO (8 mg) spectra were recorded every 2 minutes for a total of 20 minutes in DMSO : H_2_O 1 : 1 and then monitored over 24 h by UV-Vis spectroscopy. A similar experiment was performed for the absorption of MB (2.68 mM, 200 mL DSO : H_2_O) upon addition of ZnBTSC2@GO hybrid (containing 8 mg of ZnBTSC2 anchored onto 8 mg GO). Qualitatively the complete discoloration of the MB samples (in the presence of GO or ZnBTSC2@GO) was observed within 20 minutes. A similar experiment was also performed with observations over 3 h and with data recorded very 20 min, in the absence of irradiation, then the solution was left overnight, and a final UV/Vis measurement was made. For the samples containing the GO with MB monitored in the absence of UV irradiation changes from blue to colourless with disappearance of the peak of MB in UV/Vis spectra occurred rapidly. Furthermore, adding the hybrid ZnBTSC2@GO to MB solution discolours much faster, and appeared to precipitate out of solution with much ease in contrast to the dispersion of GO alone. Thus, on the timescale evaluated, these reaction is clearly dominated by the adsorption equilibria. The pseudo-first order kinetics behaviour estimated for the adsorption of dyes on the hybrid materials with or without irradiation was probed using UV-Vis spectroscopy in line with published reports.^100^

### Fluorescence titration experiments

Fluorescent titration of a GO dispersion with a Acetyl Glycosyl Coumarin solution. A 1 mM solution of AcGC (3) was prepared. To this solution was added a saturating quantity of GO (1 mg mL^−1^). The resulting saturated suspensions were then sonicated until the mixtures became homogeneous. The excess of coronene was allowed to settle. The resulting suspension supernatant was titrated against another 1 mM solution of AcGC (3) in DMSO. Excitation wavelength was set at 310 nm for all experiments. An emission range 330–600 nm was scanned at 500 nm cm^−1^ for all experiments. Titrations were conducted in a 1.2 mL cuvette. Suspension containing AcGC (3) at 1 mM concentration in DMSO was added to the cuvette and the emission spectrum scanned. Subsequent scans were conducted with 50 μL aliquots removed from cuvette and replaced with 50 μL aliquots of 1 mM AcGC (3) suspension saturated with GO in order to maintain constant AcCG (3) concentration throughout the experiments. The resulting saturated suspension was well mixed before and after the addition of the saturated suspension of GC and GO and was allowed to settle before each measurement for 5 min at r.t. before measurements.

### Estimation of binding stoichiometry between glycosyl coumarin and coronene

To a 0.125 mM solution of GC (4) coronene was added until the maximum concentration was reached in which the suspension was stable (0.125 mM). The resulting concentrated suspensions were then sonicated until the mixtures became homogeneous. The excess of coronene was allowed to settle. The resulting suspension supernatant was titrated against another 0.125 mM solution of GC (4) in DMSO. Excitation wavelength was set at 310 nm for all experiments. An emission range of 330–600 nm was scanned at 100 nm min^−1^ for all experiments. Titrations were conducted in a 1.4 mL cuvette. A suspension containing GC (4) at 0.125 mM concentration in DMSO was added to the cuvette and the emission spectrum scanned. Subsequent scans were conducted with 5 μL to 100 μL aliquots removed from the cuvette and replaced with a corresponding aliquot of 0.125 mM GC (4) suspension saturated with coronene in order to maintain constant CG (4) concentration throughout the experiments. The resulting saturated suspension was well mixed before and after the addition of the saturated suspension of GC and coronene.

### Evaluation of graphene oxide as simultaneous carrier for glycosyl coumarin and ZnBTSC3 complex

A 1 mM solution of GC (4) was prepared. To this solution was added a saturating quantity of the hybrid denoted (1 mg mL^−1^). The resulting saturated suspensions were then sonicated until the mixtures became homogeneous. The excess coronene was allowed to settle. The resulting suspension supernatant was titrated against another 1 mM solution of GC (4) in DMSO. Excitation wavelength was set at 310 nm for all experiments. An emission range of 330–800 nm was scanned at 500 nm min^−1^ for all experiments. Titrations were conducted in a 1.2 mL cuvette. A suspension containing GC (4) at 1 mM concentration in DMSO was added to the cuvette and the emission spectrum was scanned. Subsequent scans were conducted with 20 μL to 200 μL aliquots removed from cuvette and replaced with a corresponding aliquot of 1 mM GC (4) suspension saturated with hybrid complex ZnBTSC3@GO to maintain constant CG (4) concentration throughout the experiments. The resulting saturated suspension was well mixed before and after the addition of the saturated suspension of GC and complex ZnBTSC3@GO. The mixture was allowed to stand for 2–3 min at the r.t. before each measurement.

## Data availability

The processed data is given in ESI,† the raw data is available from the corresponding authors upon reasonable request.

## Conflicts of interest

There are no conflicts to declare.

## Supplementary Material

NA-006-D3NA01042B-s001

NA-006-D3NA01042B-s002
