# Supplementary material for: Shedding light on the use of graphene oxide-thiosemicarbazone hybrids towards the rapid immobilisation of methylene blue and functional coumarins
Source: Nanoscale Adv. 2024 Jan 10;6(9):2287–305. doi: 10.1039/d3na01042b (PMC11059481; doi:10.1039/d3na01042b)
Supplement: NA-006-D3NA01042B-s001 [file NA-006-D3NA01042B-s001.pdf]

Electronic Supplementary Material (ESI) for Nanoscale Advances.
This journal is © The Royal Society of Chemistry 2024

Supplementary Materials

Shedding Light on the Use of Graphene Oxide-Thiosemicarbazone Hybrids Towards the Rapid Immobilisation of Methylene Blue and Functional Coumarins

Danielle Bradley[a], Sophia Sarpaki[a], Vincenzo Mirabello[a], Simone Giuseppe Giuffrida[a], Gabriele Kociok-Köhn[a], David G. Calatayud *[a,b] and Sofia I. Pascu *[a]

[a] Department of Chemistry, University of Bath, Claverton Down Rd., BA2 7AY, Bath, UK

e-mail: S.Pascu@bath.ac.uk

[b] Department of Inorganic Chemistry, Faculty of Sciences, Universidad Autónoma de Madrid, Francisco Tomás y Valiente 7, Campus de Cantoblanco 28049 Madrid, Spain.

e-mail: david.gcalatayud@uam.es

General Experimental Methods

All reagents referred to in the experimental procedure were provided from Sigma-Aldrich or Fischer and were used as described with no means of purification before use. Microwave assisted techniques were conducted using a Biotage Initiator 2.5 Reactor with sealed and magnetically stirred glass tubes. NMR spectra were recorded using 300 MHz Bruker or 500 MHz Aligent spectrometers and referenced to the necessary residual solvent peaks. UV/Vis spectra measurements were obtained in DMSO using a Perkin Elmer Lambda 35 UV/VIS Spectrophotometer. The corresponding fluorimetry studies were recorded using a Perkin Elmer LS 55 Luminescence Spectrophotometer and further to this all quantum yield measurements were made with reference to a standard solution of $[Ru(bipy)_3](PF_6)_2$ in water (4.45 µM concentration).

$$\emptyset_S = \emptyset_R \cdot \frac{D_S}{D_R} \cdot \frac{I_S}{I_R} \cdot \frac{A_S}{A_R} \cdot \left(\frac{\eta_S}{\eta_R}\right)^2 \quad (1)$$

Quantum yields were calculated using equation (1) where S is sample, R is reference, D = integrated area of fluorescence emission, I is the peak intensity of fluorescence, A is the peak Absorbance and η is the refractive index.

Transmission electron microscopy (JEOL JEN 2100 Plus) was used to image thin films (solvent evaporated) of samples on LACEY carbon grids. A Scanning Electron Microscope (JEOL JSM 648 OLV) was used to image samples that had been deposited on glass Mica sleeves with solvent evaporated or if a solid sample placed on carbon tape. All samples for imaging were coated with a 10 nm conductive thin film of chromium or gold. Further to this Energy Dispersive X-ray spectroscopy was recorded using the SEM with reference to a silica calibration sample. All samples were uncoated for EDX analysis.

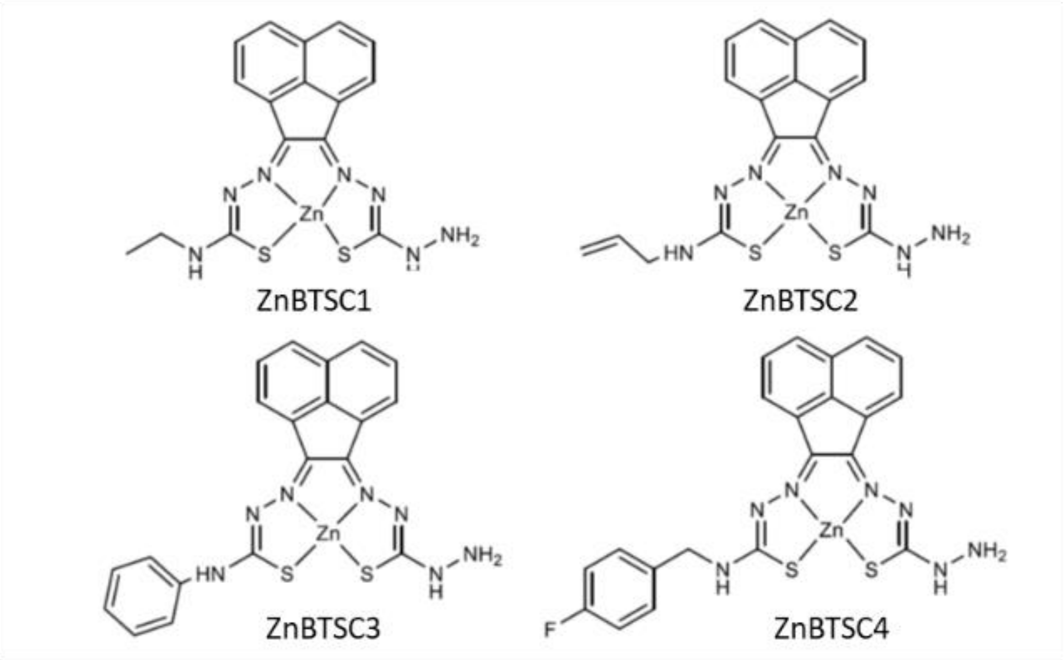

Figure S1. Schematic representation of small molecular synthetic building blocks used hereby.

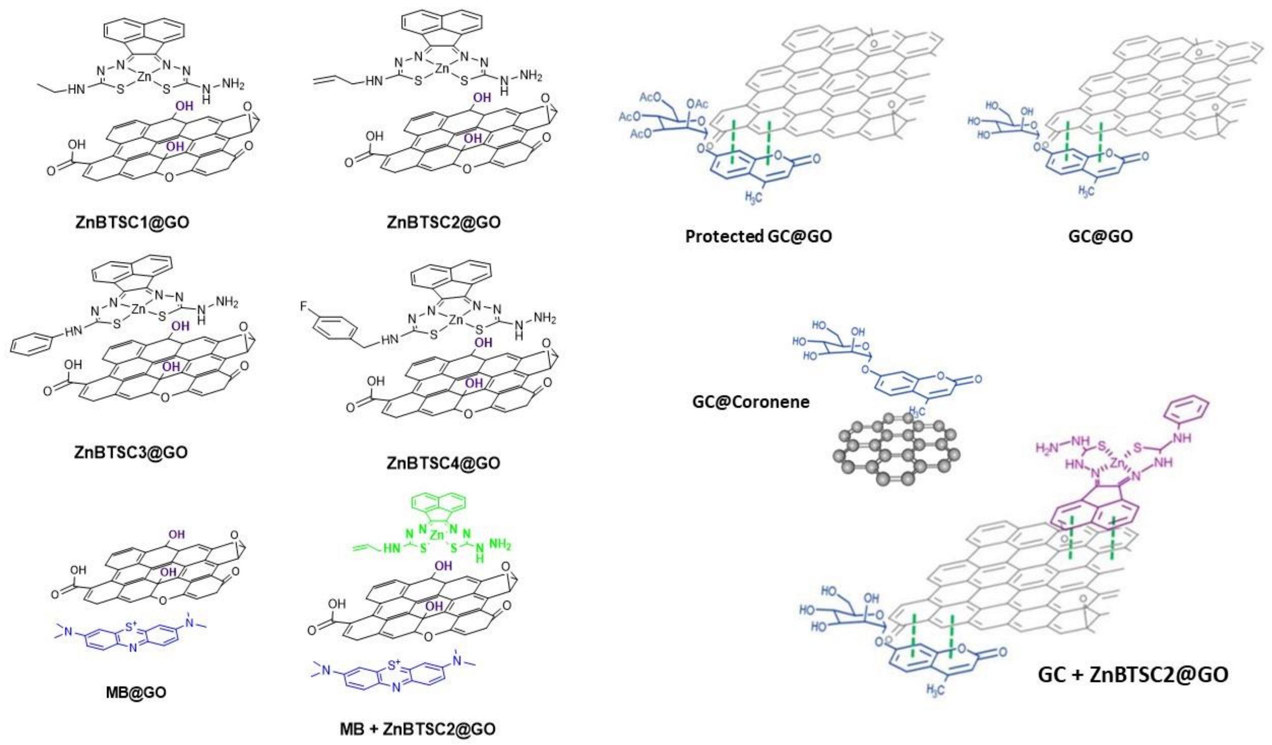

Figure S2. Schematic representation of nanohybrids formed in the dispersed phase (aqueous DMSO) investigated hereby.

Adapted synthesis of known monosubstituted ligands

Mono (substituted) 4-phenyl-3-thiosemicarbazone acenaphthenequinone:

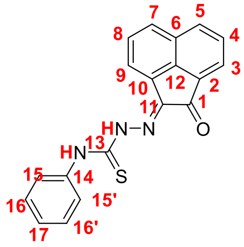

A microwave tube was filled with acenapthenoquinone (0.250g, 1.37 mmol), 4-phenylthiosemicarbazid (0.221 g, 1.37 mmol), 10 ml of Ethanol and 2 drops of concentrated HCl. The mixture was reacted at 90 °C in the microwave for 10 minutes. The slurry was then allowed to cool, filtrated, and washed with diethyl ether. The precipitate was collected to afford the desire compound (**7a**) in an orange colour with 58% yield. No further purification was necessary.

^{1}H NMR (500MHz, DMSO) δ_H : 12.85 (s, 1H, N-N***H***); 11.01(s, 1H, N***H***-Ph); 8.42(d, 1H, ^3J= 8.3Hz, $\boldsymbol{H^3}$); 8.16 (overlapping m, 3H, $\boldsymbol{H^7}$, $\boldsymbol{H^5}$, $\boldsymbol{H^9}$); 7.89 (overlapping t, 2H, $\boldsymbol{H^8}$, $\boldsymbol{H^4}$), 7.65 (obscured d, 2H, $\boldsymbol{H^{15}}$ $\boldsymbol{H^{15'}}$), 7.47 (t, m-H, ^3J= 7.6 Hz, 8.3 Hz, 2H, $\boldsymbol{H^{16}}$ $\boldsymbol{H^{16'}}$), 7.31 (t, ^3J= 7.6 Hz, 8.3 Hz, 1H, $\boldsymbol{H^{17}}$) ppm.

^{13}C NMR (125MHz, DMSO) δ_C: 189.12 (***C***=O); 177.11 (NHR-***C***S-NHPh); 139.87 (***C***=N-R); 137.34 ($\boldsymbol{C^2}$); 138.21 ($\boldsymbol{C^{10}}$); 133.33 ($\boldsymbol{C^3}$); 130.93 ($\boldsymbol{C^{14}}$); 130.44 ($\boldsymbol{C^3}$); 130.40 ($\boldsymbol{C^6}$); 129.42 ($\boldsymbol{C^8}$); 129.10 ($\boldsymbol{C^4}$); 128.91 ($\boldsymbol{C^{16}}$); 127.74 ($\boldsymbol{C^9}$); 126.64 ($\boldsymbol{C^{17}}$); 126.23 ($\boldsymbol{C^{15}}$); 123.03 ($\boldsymbol{C^7}$); 119.34 ($\boldsymbol{C^5}$).

Mass Spectrometry: ASAP for $C_{19}H_{13}N_3OS$, calcd for ($[M-H]^+$) 330.0696 amu found 330.0699 amu

IR (solid): ν (cm^{-1}) 3336, 3269, 3059, 1688, 1671, 1596, 1523, 1475.

HPLC (Method B): R_f = 11.41 min

Mono (substituted) 3-allyl-4-thiosemicarbazone acenaphthenequinone:

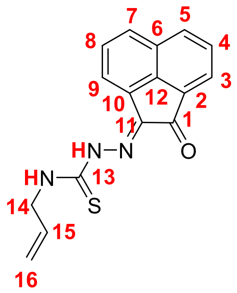

A microwave tube was filled with acenapthenoquinone (0.500 g, 2.74 mmol), 4-phenylthiosemicarbazid (0.3595 g, 2.74 mmol), 15 ml of Ethanol and 0.1ml of concentrated HCl. The mixture was reacted at 90 °C in the microwave for 10 minutes. The slurry was then allowed to cool, filtrated and washed with diethyl ether. The precipitate was collected to afford the desired compound (**7b**) in an orange colour with 58% yield, which was used in subsequent reactions without further purification.

^{1}H NMR (300MHz, DMSO-d$_6$, 298 K) δ_H: 12.63 (s, 1H, N-N*H*); 9.59(t, 1H, ^3J= 5.75Hz, 11.37Hz, N**H**-Allyl); 8.36 (d, ^3J= 8.19Hz, 1H, ***H*^{3}**); 8.11 (overlapping d, ^3J= 8.19Hz, 1H,***H*^{7}**), 8.08 (overlapping d, ^3J= 6.39 Hz, 1H,***H*^{5}**), 7.84 (overlapping m, 2H, ***H*^{4}, *H*^{8}**), 5.96 (m, 1H, NH-C***H*$_2$**-R), 5.22 (d, ^3J= 16.5Hz, 1H, RCH-C***H*$_2$**); 4.30 (t, 1H, , ^3J= 4.9 Hz, 9.9Hz, R-C***H***-CH$_2$).

^{13}C NMR (125MHz, DMSO) δ_C: 188.55 (***C***=O); 177.56 (NHR-*C*S-NHAllyl); 139.15 (***C*^{12}**); 137.34 (***C*^{2}**); 134.01 (R-***C***H-CH$_2$); 132.79 (***C*^{3}**); 130.46 (***C*^{10}**); 130.06 (***C***=N-R); 129.90(***C*^{6}**); 128.90 (***C*^{4}**); 128.61 (***C*^{11}**); 127.10 (***C*^{7}**); 122.49 (***C*^{5}**); 118.37 (***C*^{9}**); 116.31 (RCH-***C***H$_2$); 46.46 (NH-***C***H$_2$-R) ppm.

Mass Spectrometry (ASAP) for $C_{16}H_{13}N_3OS$, calcd for ([M-H]$^+$) 294.0691 amu found 294.0696 amu

IR (solid): ν (cm^{-1}) 3302, 3280, 2977, 1685, 1538, 1475, 1056, 1027.

HPLC (Method A): R$_f$ = 10.82 min

Mono (substituted) 3-ethyl-4-thiosemicarbazone acenaphthenequinone:

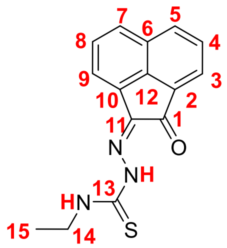

A microwave tube was filled with acenapthenoquinone (0.500 g, 2.74 mmol), 4-ethylthiosemicarbazide (0.3259 g, 2.74 mmol), 15 ml of Ethanol and 0.1ml of concentrated HCl. The mixture was reacted at 90 °C in the microwave for 10 minutes. The slurry was then allowed to cool, filtrated and washed with diethyl ether. The precipitate was collected to afford the desired compound (**7c**) in an orange colour with 84% yield. No further purification was necessary.

^{1}H NMR (300MHz, DMSO-d$_6$, 298 K) δ_H: 12.59 (s, 1H, N-N***H***); 9.44 (t, 1H, ^3J= 5.44 Hz, 10.66 Hz, N***H***-Et); 8.36 (d, ^3J= 8.27 Hz, 1H, ***H*^{3}**); 8.12 (overlapping d, ^3J= 8.49Hz, 1H,***H*^{7}**), 8.08 (overlapping d, ^3J= 6.96 Hz, 1H,***H*^{5}**), 7.99 (d, ^3J=6.96 Hz, 1H, ***H*^{9}**), 7.84 (overlapping m, 2H, ***H*^{4}, *H*^{8}**), 3.66 (q, 2H, NH-C***H*$_2$**-CH$_3$), 1.20 (t, 1H, , ^3J= 7.08 Hz, 14.09 Hz, 3H, R-C***H*$_3$**).

^{13}C NMR (125MHz, DMSO) δ_C: 188.91 (***C***=O); 177.21 (NHR-*C*S-NHEt); 139.44 (***C*^{12}**); 137.53 (***C*^{2}**); 133.14 (***C*^{3}**); 130.84 (***C*^{10}**); 130.46 (***C***=N-R); 130.26(***C*^{6}**); 129.27(***C*^{11}**); 128.98 (***C*^{4}, *C*^{8}**); 127.41 (***C*^{7}**); 122.84 (***C*^{5}**); 118.63 (***C*^{9}**); 14.45 (RCH$_2$-***C***H$_3$); ppm.

Mass Spectrometry: ASAP for $C_{15}H_{13}N_3OS$, calcd for ([M+H]$^+$) 284.0858 amu found 284.0855 amu

IR (solid): ν (cm^{-1}) 3302, 3280, 2977, 1685, 1538, 1475, 1056, 1027.

HPLC (Method A): R$_f$ = 10.71 min

Synthesis of Zn(BTSC) complexes:

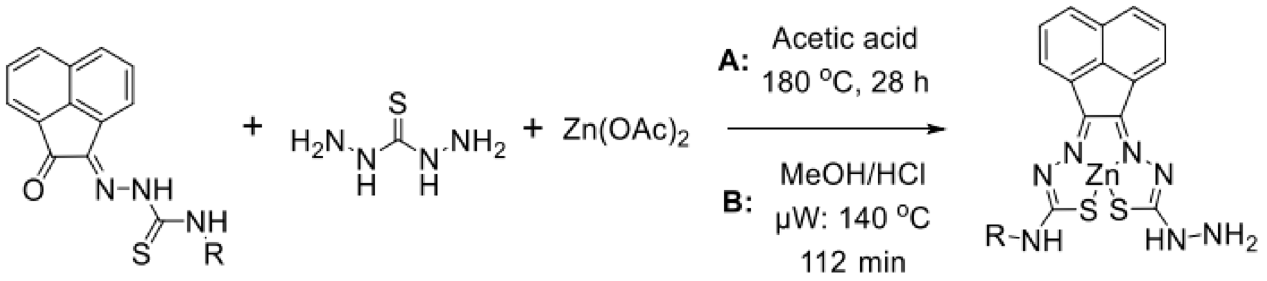

Scheme S1.

General considerations: To decrease the time of the reaction, microwave radiation was introduced as the energy source. The microwave-assisted reaction proved to be rather challenging, and optimising the working conditions was necessary. Then the reactions were successfully performed (Scheme S1, method B) with most of the compounds being obtained pure and in high yields as described below. Among the relevant points for successful optimisation are: avoid temperatures higher than 140 °C, mix the mono(substituted) ligand with the zinc acetate prior to the addition of the thiocarbohydrazide in the reaction (as for conventional heating), and lastly, replacement of acetic acid by slightly acidic methanol (0.01% v/v HCl in methanol). More specifically, the reactions for the synthesis of metal asymmetric thiosemicarbazonates were carried out as follows: a microwave vial was filled with $Zn(OAc)_2$, in methanol, and heated (conventional heating) to 60 °C. Then, the monosubstituted ligand was added and the temperature was increased to 120 °C. When the reaction reached 120 °C, the thiosemicarbohydrazide was added along with a drop of HCl (37%). Following, the reaction was allowed to react at 140 °C (under microwave radiation) for 112 minutes. The crude product was then filtered and washed with cold methanol and diethyl ether to remove any traces of the starting materials.

Zinc(II) 4-phenyl-3-thiosemicarbazo ne-thiocarbohydrazide acenapthenoquinone (ZnBTSC3):

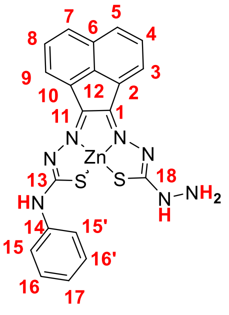

In a microwave vial 3 mL of EtOH were added and heated to 60 °C. To this, zinc acetate (0.2768 g, 1.50 mmol) was added along with 4-phenyl-3-thiosemicarbazone acenopthenoquinone (**3a**) (0.1 g, 0.31 mmol). Then the reaction was heated further to 120 °C and thiosemicarbohydrazide (0.0961 g, 0.91 mmols) was added together with 2 mL of EtOH and couple of drops of HCl. The reaction mixture was heated under µW radiation at 150 °C for 106 min. Then was filtrated hot, re-suspended in diethyl ether (100 ml), stirred for another 15min and filtrated under vacuum. Resulting deposit was further washed with diethyl ether (200 ml) and Zinc (II) 4-phenyl-3-thiosemicarbazone-thiocarbohydrazide acenapthenoquinone (**6a**) was isolated as an orange colour solid with a yield of 95 % (0.1599 g).

^{1}H NMR (500MHz, DMSO-d_6, 298 K) δ_H : 9.92 (s, 1H, N***H***-Ph); 8.64 (s, 1H, $\boldsymbol{H^3}$); 8.64 (s, 1H, $\boldsymbol{H^9}$); 7.93 (d overlapping, 2H, $\boldsymbol{H^5}$, $\boldsymbol{H^7}$), 7.68 (overlapping, 3H, $\boldsymbol{H^8}$, $\boldsymbol{H^{15}}$ $\boldsymbol{H^{15'}}$); 7.48 (t, ^3J=8.1, 16.3Hz, 1H, $\boldsymbol{H^4}$), 7.44 (t, ^3J=7.8, 15.6Hz, 2H, $\boldsymbol{H^{17}}$); 7.14 (t, ^3J=7.3, 14.6Hz, 1H, $\boldsymbol{H^{16}}$ $\boldsymbol{H^{16'}}$); 6.78 (s, 2H, N$\boldsymbol{H_2}$).
^{13}C NMR (125MHz, DMSO) δ_C: 175.98 (RN=***C***S-NHPh); 147.20 (RN=***C***S-NHNH$_2$); 140.27 ($\boldsymbol{C^{11}}$); 139.93 ($\boldsymbol{C^{1}}$); 135.00 ($\boldsymbol{C^{2}}$); 132.50 ($\boldsymbol{C^{10}}$); 130.15 ($\boldsymbol{C^{14}}$); 129.50 ($\boldsymbol{C^{12}}$); 128.56 ($\boldsymbol{C^{16}}$); 128.51 ($\boldsymbol{C^{6}}$); 128.05($\boldsymbol{C^{7}}$); 127.94 ($\boldsymbol{C^{8}}$); 127.80 ($\boldsymbol{C^{5}}$); 127.37 ($\boldsymbol{C^{4}}$); 126.13 ($\boldsymbol{C^{3}}$); 124.07 ($\boldsymbol{C^{17}}$); 123.03 ($\boldsymbol{C^{15}}$); 120.53 ($\boldsymbol{C^{9}}$).
Mass Spectrometry: MALDI-MS for $C_{20}H_{15}N_7S_2Zn$, calcd for ($[M]^+$) 481 amu found 481 amu.
HPLC (Method A): R_f = 11.55, 12.13 min

Zinc(II) 3-allyl-3-thiosemicarbazone-thiocarbohydrazide acenapthenoquinone (ZnBTSC2):

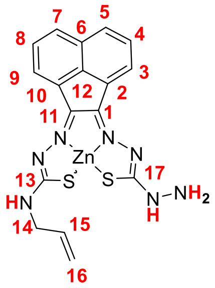

In a microwave vial 3 mL of EtOH were added and heated to 60 °C. To this, zinc acetate (0.3106 g, 1.69 mmol) was added along with 3-allyl-3-thiosemicarbazone acenopthenoquinone (**3b**) (0.1 g, 0.35 mmol). Then the reaction was heated further to 120 °C and thiosemicarbohydrazide (0.1091 g, 1.06 mmols) was added together with 2 mL of EtOH and couple of drops of HCl. The reaction mixture was heated under μW radiation at 150 °C for 106 min. Then was filtrated hot, re-suspended in diethyl ether (100 ml), stirred for another 15 min and filtrated under vacuum. Resulting deposit was further washed with diethyl ether (200 ml) and Zinc (II) 3-allyl-3-thiosemicarbazone-thiocarbohydrazide acenapthenoquinone (**6b**) was isolated as an orange colour solid with a yield of 86 % (0.135 g).

^{1}H NMR (500MHz, DMSO-d$_6$, 298 K) δ: 8.58 (bs, 1H, N***H***-Allyl); 8.33 (bs, 1H, $\boldsymbol{H^3}$); 7.94 (3H, $\boldsymbol{H^1}$**,** $\boldsymbol{H^2}$**,** $\boldsymbol{H^{2'}}$ overlapping), 7.67 (3H, $\boldsymbol{H^{1'}}$**,** $\boldsymbol{H^3}$**,** $\boldsymbol{H^{3'}}$ overlapping); 6.58 (bs, 2H,N***H***$_2$); 6.03 (bs, 1H, RC***H***=CH$_2$); 5.30 (d, 1H, ^{3}J= 1.6 Hz, RCH=C***H***$_2$); 5.15 (d, 1H, ^{3}J= 10.7Hz, RCH=C***H***$_2$); 4.19 (bs, 1H, N***H***CH$_2$R) ppm.

Mass Spectrometry: MALDI-MS for $C_{17}H_{15}N_7S_2Zn$, calcd for ([M]$^+$) 445 amu found 445 amu and for ([M+H]$^+$) 446 amu found 446 amu.

IR (solid): ν (cm^{-1}) 3305, 3254, 3136, 2935, 1627, 1578, 1392, 1242, 1137.

HPLC (Method A): R_f = 11.35 min

Zinc(II) 3-ethyl-3-thiosemicarbazone-thiocarbohydrazide acenapthenoquinone (ZnBTSC1):

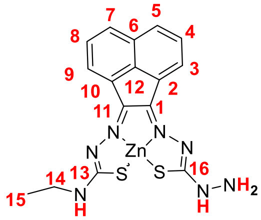

In a microwave vial 3 mL of EtOH were added and heated to 60 °C. To this, zinc acetate (0.3241 g, 1.77 mmol) was added along with 3-ethyl-3-thiosemicarbazone acenopthenoquinone (**3c**) (0.1 g, 0.35 mmol) Then the reaction was heated further to 120 °C and thiosemicarbohydrazide (0.1125 g, 1.06 mmols) was added together with 2 mL of EtOH and couple of drops of HCl. The reaction mixture was heated under μW radiation at 150 °C for 106 min. Then was filtrated hot, re-suspended in diethyl ether (100 ml), stirred for another 15min and filtrated under vacuum. Resulting deposit was further washed with diethyl ether (200 ml) and Zinc (II) 3-allyl-3-thiosemicarbazone-thiocarbohydrazide acenapthenoquinone (**6b**) was isolated as an orange colour solid with a yield of 86 % (0.135 g).

^{1}H NMR (500MHz, DMSO-d$_6$, 298 K) δ: 8.98 (bs, 1H, N***H***-Ethyl); 8.22 (bs, 1H, N***H***-NH$_2$); 8.62 (, 2H, $\boldsymbol{H^3}$, $\boldsymbol{H^9}$ overlapping); 7.96 (2H, $\boldsymbol{H^5}$**,** $\boldsymbol{H^7}$ overlapping), 7.68 (2H, $\boldsymbol{H^4}$**,** $\boldsymbol{H^{8'}}$ overlapping); 6.58 (bs, 2H,N***H***$_2$); 6.03 (bs, 1H, RC***H***=CH$_2$); 5.30 (d, 1H, ^{3}J= 1.6 Hz, RCH=C***H***$_2$); 5.15 (d, 1H, ^{3}J= 10.7Hz, RCH=C***H***$_2$); 4.19 (bs, 1H, N***H***CH$_2$R) ppm.

^{13}C NMR (125MHz, δ_C: 176.7 (RN=***C***S-NHbnzF); 176.6 (RN=***C***S-NHNH$_2$); 146.39 ($\boldsymbol{C^{11}}$); 138.1 ($\boldsymbol{C^1}$); 135.4 ($\boldsymbol{C^2}$); 135.3 ($\boldsymbol{C^{10}}$); 131.3 ($\boldsymbol{C^{12}}$); 128.7 ($\boldsymbol{C^8}$*) 128.3 (*$\boldsymbol{C^4}$); 127.7 ($\boldsymbol{C^7}$); 126.7 ($\boldsymbol{C^5}$); 126.1 ($\boldsymbol{C^9}$); 130.0 ($\boldsymbol{C^{14}}$); 38.3 ($\boldsymbol{C^{14}}$) 15.0($\boldsymbol{C^{15}}$) ppm,

Mass Spectrometry: MALDI-MS for $C_{17}H_{15}N_7S_2Zn$, calcd for ([M]$^+$) 433 amu found 433 amu

HPLC (Method A): R_f = 11.23 min

Zinc(II) 4-fluorobenzyl-3-thiosemicarbazone-thiocarbohydrazide acenapthenoquinone (ZnBTSC4):

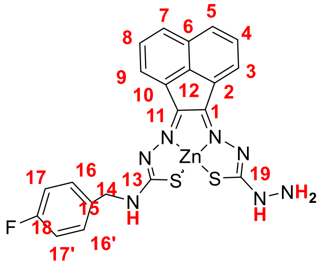

In a microwave vial 3 mL of EtOH were added and heated to 60 °C. At that moment zinc acetate (0.2527 g, 1.38 mmol) was added along with 4-fluoro-benzyl-3-thiosemicarbazone acenopthenoquinone (**3e**) (0.1 g, 0.28 mmol) Then the reaction was heated further to 120 °C and thiosemicarbohydrazide (0.0881 g, 0.83 mmols) was added together with 2 mL of EtOH and couple of drops of HCl. The reaction mixture was heated under μW radiation at 150 °C for 106 min. Then was filtrated hot, re-suspended in diethyl ether (100 ml), stirred for another 15min and filtrated under vacuum. Resulting deposit was further washed with diethyl ether (200 ml) and Zinc (II) 4-phenyl-3-thiosemicarbazone-thiocarbohydra-zide acenapthenoquinone (**6e**) was isolated as an orange colour solid with a yield of 96 % (0.143 g).

^{1}H NMR (500MHz, DMSO-d$_6$, 298 K) δ_H : 9.60 (bs, 1H, N***H***-bnzF); 8.57 (s, 1H, ***H^3***); 8.39 (s, 1H, ***H^9***); 7.91 (overlapping, 2H, ***H^5***, ***H^7***), 7.62 (overlapping, 3H, ***H^8***, ***H^4*, *H^3***); 7.44 (t, ^3J= 7.26 Hz, 13.44 Hz, 2H, ***H^{16} H$^{16'}$***);
7.16 (t, ^3J= 7.99 Hz, 16.34 Hz, 2H, ***H^{17} H$^{17'}$***); 6.42 (bs, 2H, N***H$_2$***); 4.72 (t, 2H, ^3J= 5.46 Hz, 11.93 Hz, ***H^{14}***).
^{13}C NMR (125MHz, δ_C: 177.8 (RN=***C***S-NHbnzF); 176.6 (RN=***C***S-NHNH$_2$); 162.7 (***C^{11}***); 160.6 (***C^1***); 146.98(***C^{15}***); 136.2 (***C^2***); 135.3 (***C^{10}***); 130.9 (***C^{14}***); 130.0 (***C^{12}***); 129.3 (***C^{18}***); 128.52 (***C^3***); 128.34 (***C^8*, *C^4***); 127.84 (***C^7***); 126.75 (***C^5***); 126.31 (***C^9***); 115.39 (d, *J*= 21.45 Hz ***C^{17}***); 68.7 (***C^{19}***)45.83 (***C^{14}***) ppm,
^{19}F NMR (500 MHz, DMSO) δ_C : -116.49
Mass Spectrometry: MALDI-MS for $C_{20}H_{15}N_7S_2Zn$, calcd for ($[M]^+$) 513 amu found 513 amu.
HPLC (Method A): R$_f$ = 12.34 min

Synthesis and functionalization of glycosyl coumarin derivatives

The synthetic protocols were carried out using an adapted protocol based on that previously reported[2].

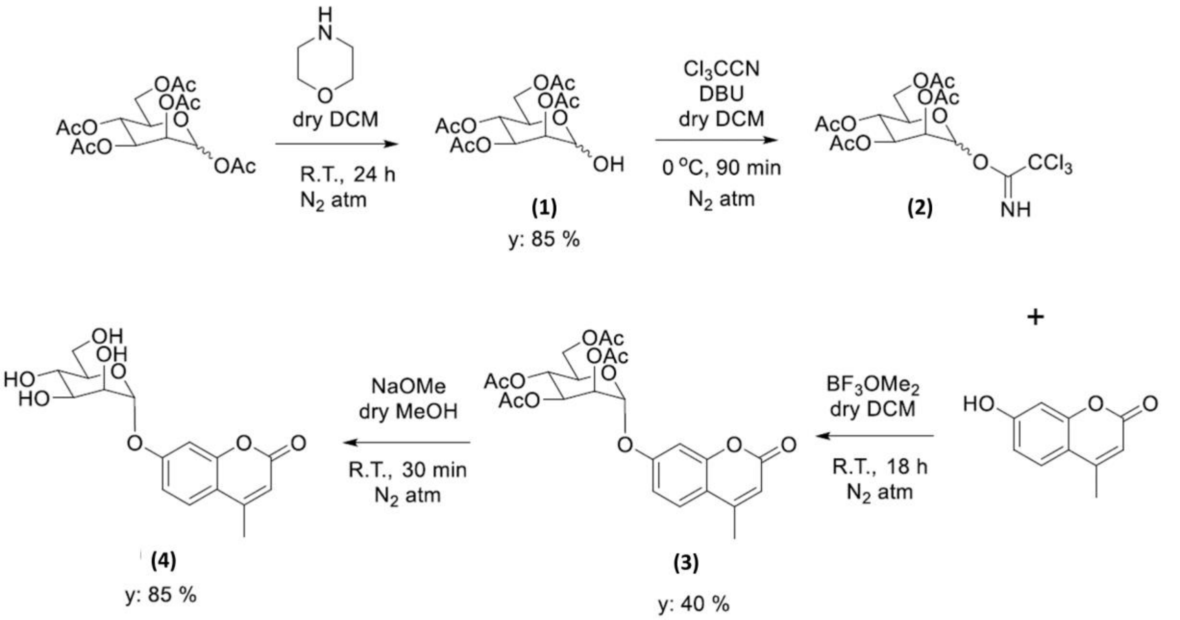

Scheme S2

Synthesis of 2,3,4,6-Tetra-O-acetyl-D-mannopyranose:

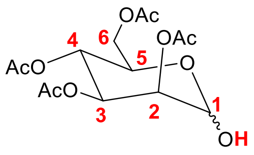

α-D-mannose pentaacetate (d) (3.0 g, 7.69 mmol) was dissolved in dry DCM (30 mL). Morpholine (2.68 ml, 30.75 mmol) was then added and the reaction mixture was let under N_2 at room temperature, overnight. The slurry was first washed with HCl solution (1 N) (2*30 ml), then with distilled water (3*30 ml) and finally dried over magnesium sulphate. The excess solvent was removed under reduced pressure to afford 2,3,4,6-Tetra-O-acetyl-D-mannopyranose (**1**) as a light yellow oil at room temperature, which was used without any further purification. (2.16 g, 71 %).

^{1}H NMR (500MHz, $CDCl_3$) δ_H: 5.26 (dd, J=3.28, 10.09Hz,1H, $\boldsymbol{H^4}$); 5.15 (t, J=9.9, 19.9Hz, 1H, $\boldsymbol{H^5}$); 5.10 (d, J=3.5, 1H, $\boldsymbol{H^3}$); 5.08 (s, 1H, $\boldsymbol{H^2}$); 4.12 (overlapping dd, 1H, $\boldsymbol{H^8}$); 4.09 (overlapping pd, 1H, $\boldsymbol{H^{6b}}$); 3.97 (pd, 1H, $\boldsymbol{H^{6a}}$); 2.04 (s, 3H, C$\boldsymbol{H_3}$Ac); 1.98 (d, J=3.3Hz, 3H, C$\boldsymbol{H_3}$Ac); 1.94 (s, 3H, C$\boldsymbol{H_3}$Ac); 1.88 (d, J=5.0Hz, 3H, C$\boldsymbol{H_3}$Ac);

^{13}C NMR (125MHz,, CDCl3) δ_C: 170.94 (**C**=O); 170.25 (**C**=O); 170.10 (**C**=O); 169.90 (**C**=O); 91.89 (**C**-OH); 70.23 ($\boldsymbol{C^2}$); 68.89 (C^3); 68.04 ($\boldsymbol{C^5}$); 66.14 ($\boldsymbol{C^4}$); 62.53 ($\boldsymbol{C^6}$); 20.76 (**C**H_3OOR); 20.60 (**C**H_3OOR); 20.57 (**C**H_3OOR); 20.56 (**C**H_3OOR) ppm.

Mass Spectrometry: ES^+ calcd for $C_{14}H_{20}O_{10}$, ($[M+Na]^+$) calcd for 371.10 amu found 371.1039 amu

Synthesis of 2,3,4,6-Tetra-O-acetyl-D-mannopyranose trichloroacetamidate:

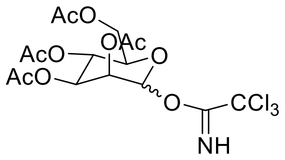

2,3,4,6-Tetra-O-acetyl-D-mannopyranose (**1**) (2.15 g, 6.17 mmol) and trichloro-acetonitrile (2.19 mL, 6.17 mmol) were dissolved in dry DCM (30 ml) and the reaction mixture was let under N_2 at O°C for 1 h. then a catalytic amount of DBU (0.19 mL, 1.23 mmols) was added and slurry was stirred for further 30 min under N_2 at O °C. The excess solvent was removed under reduced pressure to afford a crude compound of 2,3,4,6-Tetra-O-acetyl-D-mannopyranose trichloroacetamidate as a yellow oil at room temperature, which was used without any further purification.

Synthesis of 4- Methylumbelliferyl-7-yl-2,3,4,6-Tetra-O-acetyl- α -D-mannopyranose:

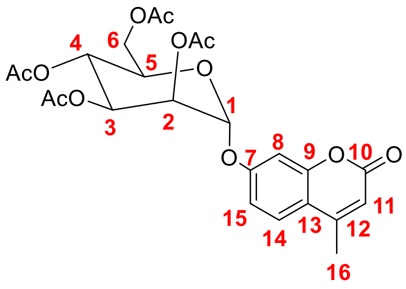

The crude 2,3,4,6-Tetra-O-acetyl-D-mannopyranose-trichloro-acetamidate) (**2**) was dissolved in dry DCM (40 mL). 4-Methylumbel-liferylone (**3**) (2.28 g, 6.17 mmols) was added to the slurry along with boron trifluoride metharate (0.11 mL, 1.2 mmol) and the reaction mix-ture was let under N_2 at 23 °C over nigh. The slurry was first diluted with 10 ml of DCM, then washed with HCl solution (1N) (2*20 ml) and distilled water (3*20 ml) and dried over magnesium sulphate. The ex-cess solvent was removed under reduced pressure. The crude product was then purified by crystallisation with methanol to afford 4-Methylumbelliferyl-7-yl-2,3,4,6-Tetra-O-acetyl- α -D-mannopyranose as a white solid (2.47 g, 40 %).

^{1}H NMR (500MHz, CDCl3) δ_H: 7.53 (d, J=9.0Hz, 1H, ***H***^{14}); 7.12 (d, J=2.4Hz, 1H, ***H***^{8}); 7.03(dd, J=2.7, 8.8Hz, 1H, ***H***^{15}); 6.20 (d, J=1.2, 1H, ***H***^{11}); 5.58 (d, J=1.8, 1H, ***H***^{1}); 5.55 (dd, J=3.5, 10.04Hz,1H, ***H***^{3}); 5.47 (pq, J=1.8, 3.5Hz, 1H, ***H***^{2}); 5.37 (t, J=10.1, 20.3Hz, 1H, ***H***^{4}); 4.29 (dd, J=5.7, 12.4Hz, 1H, ***H***^{6}); 4.05 (m, 2H, ***H***^{5}**,** ***H***^{7}); 2.41 (d, J=1.1Hz, 3H, C***H***$_3$Ac); 2.21 (s, 3H, C***H***$_3$Ac); 2.05 (d, J=4.8Hz, 9H, C***H***$_3$Ac);
^{13}C NMR (125MHz, CDCl3) δ_C: 170.51 (***C***=O); 169.97(***C***=O); 169.94(***C***=O); 169.64(***C***=O); 160.76 (***C***^{7}); 158.16 (***C***^{10}); 154.89 (***C***^{9}); 152.06 (***C***^{12}); 125.78(***C***^{14}); 115.50 (***C***^{13}); 113.44 (***C***^{15}); 113.30 (***C***^{11}); 104.38 (***C***^{16}); 95.92 (***C***^{1}); 69.56 (***C***^{2}); 69.07 (***C***^{3}); 68.66 (***C***^{5}); 65.76 (***C***^{4}); 62.03 (***C***^{6}); 20.86 (***C***H_3OOR); 20.68 (***C***H_3OOR); 20.66 (***C***H_3OOR); 20.64 (***C***H_3OOR); 18.68 (***C***H_3OOR).
Mass Spectrometry: ES$^+$ for $C_{24}H_{26}O_{12}$, calcd for ($[M+Na]^+$) 529.13 amu found 529.14 amu.

4-methylumbelliferyl-7-yl-α-D-mannopyranose:

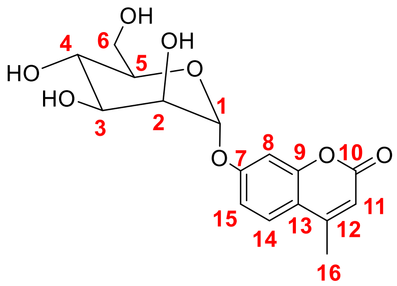

4- Methylumbelliferyl-7-yl-2,3,4,6-Tetra-O-acetyl- α -D-mannopyra-nose (4) (0.32 g, 0.64 mmol) was added in a solution of sodium meth-oxide (0.061 g, 0.96 mmol) in dry methanol (7 ml). The reaction mixture was stirred under N_2 at room temperature for 30 min. The crude product was purified by crystallisation with methanol. The solid was removed by filtration and the excess solvent of filtrate was removed by reduced pres-sure to afford the desired compound (**5**) as an off white oil (0.2 g, 93%). ^{1}H NMR (300MHz, D_2O, 298 K) δ_H : 6.85 (d, 3J = 8.2Hz, 1H, ***H***^{14}); 6.36 (d, ^{3}J = 2.3Hz, 1H, ***H***^{8}); 6.28 (dd, ^{3}J =2.3, 8.2Hz, 1H, ***H***^{15}); 5.88 (s, 1H); 5.54 (s, 1H, ***H***^{11}); 4.14 (d, ^{3}J=1.52, 1H, ***H***^{1}); 4.03 (dd, ^{3}J=3.4, 8.8Hz, 1H, ***H***^{2}); 3.79 (m, 4H, ***H***^{4}**,** ***H***^{5} **,*****H***^{6}**,** ***H***^{7}).
^{13}C NMR (125MHz, D_2O, 298 K) δ_C : 177.49 (***C***^{7}); 165.01 (***C***^{10}); 156.55 (***C***^{9}); 147.32 (***C***^{12}); 129.78 (***C***^{14}); 128.16 (***C***^{13}); 124.14 (***C***^{11}); 108.11 (***C***^{8}); 103.08 (***C***^{15}); 98.73 (***C***^{1}); 73.65 (***C***^{5}); 70.82 (***C***^{2}); 70.71 (***C***^{3}); 67.04 (***C***^{4}); 61.08 (***C***^{6}); 24.29 (***C***^{16}) ppm.
Mass Spectrometry: ES$^+$ $C_{16}H_{18}O_8$, calcd for ($[M+H]^+$) 339.1074 amu found 339.1079 amu.

Preparation of Graphene Oxide by the adapted Hummer's Method

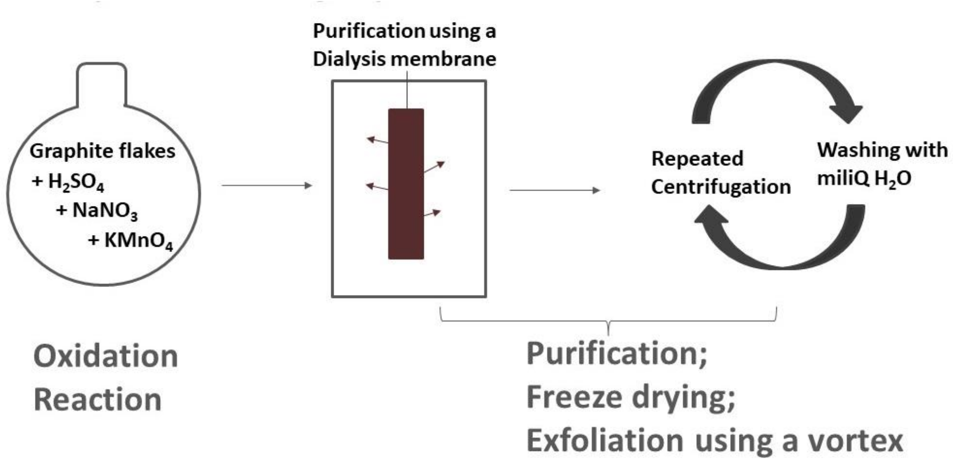

Scheme S3

Graphene flakes (0.250 g, 20.82 mmol) was placed in a round bottom flask (250 mL) with a magnetic stirrer bar and placed in a dry ice bath. Sulfuric acid (95 %, 5.75 mL) was added dropwise to the flask, followed by sodium nitrate (0.125 g, 1.47 mmol) and the reaction mixture was then stirred for 30 minutes in the ice bath. Over the duration of a one-hour period potassium permanganate (0.750 g, 4.75 mmol) was added slowly to the reaction mixture. The ice bath was then removed and the reaction mixture was heated to 45℃ and stirred for 1 hour, milli-Q water (15 mL) was added and then stirred for 30 minutes further. To end the reaction milli-Q water (30 mL) with hydrogen peroxide (3 %, 7.5 mL) was added to the mixture.

The reaction mixture was pipetted into dialysis membranes which were then sealed with clips and placed into the dialysis vessel and left for 42 hours to remove salt impurities from the graphene oxide. The membranes were then emptied into a beaker and rinsed with milli-Q water. The reaction mixture was then pipetted into Eppendorf centrifuge tubes and centrifuged (3000 rpm) for 5 minutes, the supernatant was discarded and the graphene oxide wet solid was washed with water. The samples were sonicated to mix the graphene oxide solid with the fresh water, and then centrifuged again. This process was repeated twice more, and then the solid was transferred in the minimum amount of water into one single vial, which was then freeze dried overnight.

IR (cm^{-1}): 3147 υ(O-H), 2129 υ(CO_2), 1722 υ(C=O), 1647 υ(C=C), 1387 υ(C-O), 968 υ(C-O).

Preparation of [Zn(BTSC)]-graphene oxide nanocomposites

In typical coordination experiment, the [Zn(BTSC)] complex (with R = Et, Allyl, Ph, and F-BzPh, each in 20 mg/ DMSO mL) and 20 mg of GO nanocomposites suspended in 1 mL of DMSO were mixed, and the resulted dispersion was diluted further with 2 mL of EtOH and 1 mL of $NaHCO_3$ buffer in order to adjust the pH to 5.5. The mixture was allowed to react at 100 °C for 2.5 h, when it was allowed to cool down, centrifuged, washed with EtOH and H_2O and allowed to freeze-dry overnight, prior to investigations by TEM and SEM.

Fluorescence titration experiments.

Fluorescent titration of a GO dispersion with an Acetyl Glycosyl Coumarin solution.

A 1 mM solution of Acetyl-protected Glycosyl Coumarin (AcGC), (5) was prepared. To this solution was added a saturating quantity of GO (1 mg/mL). The resulting saturated suspensions were then sonicated until the mixtures became homogeneous. The excess of coronene was allowed to settle. The resulting suspension supernatant was titrated against another 1 mM solution of Ac-protected GC (5) in DMSO. Excitation wavelength was set at 310 nm for all experiments. An emission range 330-600 nm was scanned at 500 nm cm^{-1} for all experiments. Titrations were conducted in a 1.2 mL cuvette. Suspension containing AcGC (5) at 1 mM concentration in DMSO was added to the cuvette and the emission spectrum scanned. Subsequent scans were conducted with 50 μL aliquots removed from cuvette and replaced with 50 μL aliquots of 1 mM AcGC (5) suspension saturated with GO to maintain constant AcCG (5) concentration throughout the experiments. The resulting saturated suspension was well mixed before and after the addition of the saturated suspension of GC and GO, and the dispersions were allowed to settle before each measurement for 5 min.

Evaluation of binding stoichiometry between glycosyl coumarin and coronene

To a 0.125 mM solution of glycosyl coumarin, GC (4) coronene was added until the maximum concentration was reached in which the suspension was stable (0.125 mM). The resulting concentrated suspensions were then sonicated until the mixtures became homogeneous. The excess of coronene was allowed to settle. The resulting suspension supernatant was titrated against another 0.125 mM solution of GC (4) in DMSO. Excitation wavelength was set at 310 nm for all experiments. An emission range of 330-600 nm was scanned at 100 nm min-1 for all experiments. Titrations were conducted in a 1.4 mL cuvette. A suspension containing GC (4) at 0.125 mM concentration in DMSO was added to the cuvette and the emission spectrum scanned. Subsequent scans were conducted with 5 μL to 100 μL aliquots removed from the cuvette and replaced accordingly with the corresponding aliquot of 0.125 mM GC (4) as a suspension saturated with coronene to maintain constant CG (4) concentration throughout the experiments. The resulting saturated suspension was well mixed before and after the addition of the saturated suspension of GC and coronene and prior to fluorescence and UV-vis spectroscopy.

Selected characterization data for [Zn(BTSCs)] small molecular compounds.

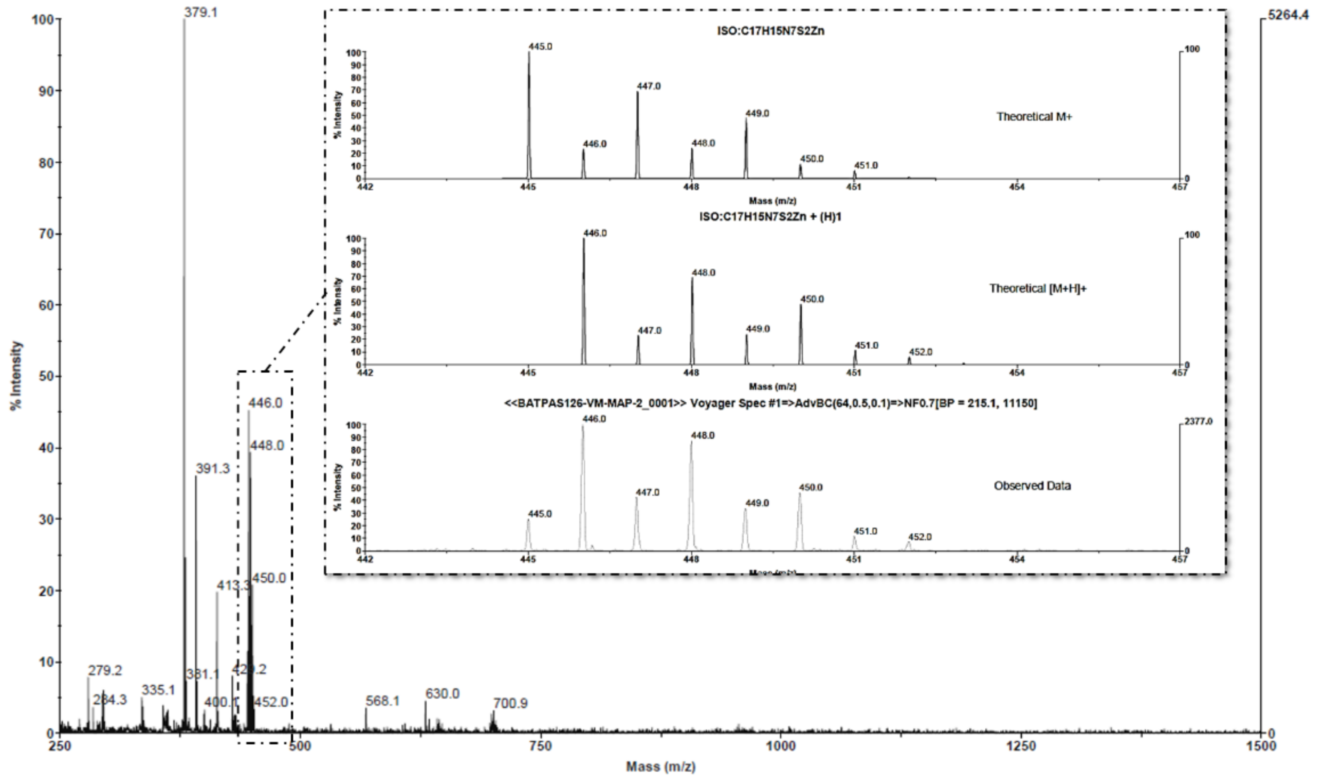

Figure S3. MALDI analysis mass spectrometry result for the Zinc(II) 3-allyl-3-thiosemicarbazone-thiocarbohydrazide acenaphthenequinone (R = allyl), denoted ZnBTSC2.

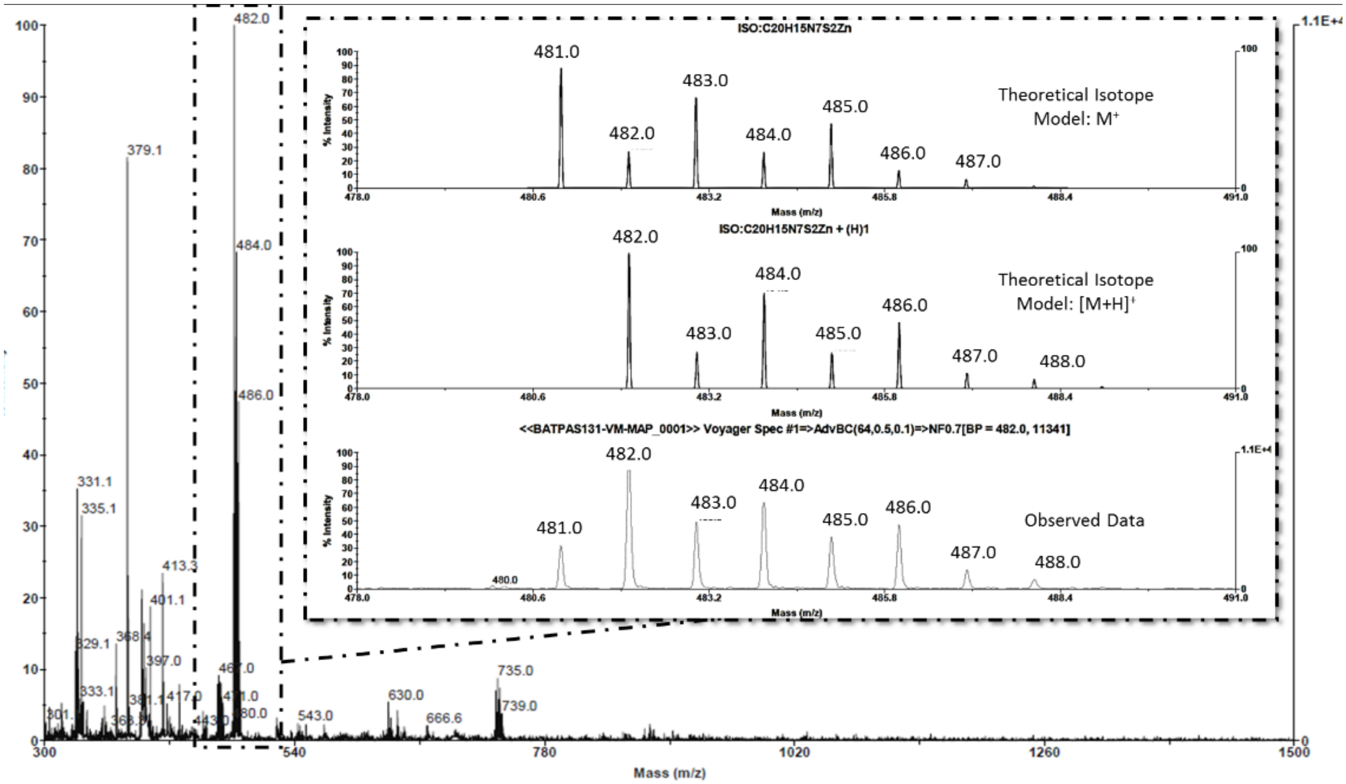

Figure S4. MALDI analysis mass spectrometry for Zinc(II) 4-phenyl-3-thiosemicarbazone-thiocarbo-hydrazide acenaphthenequinone, denoted ZnBTSC3.

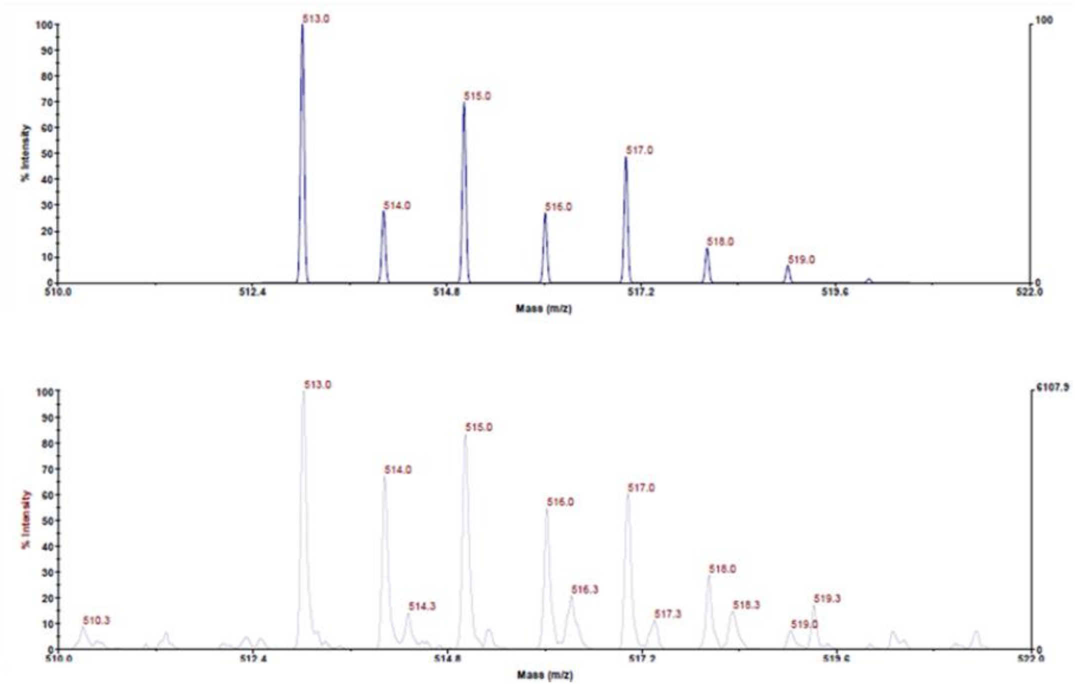

Figure S5. MALDI mass spectrometry data for Zinc(II) p-F-4-phenyl-3-thiosemicarbazone-thiocarbo-hydrazide acenaphthenequinone. a) theoretical isotope pattern [M]+ = 513.02 amu b) observed data [M]+ = 513.0 amu (denoted compound ZnBTSC4)

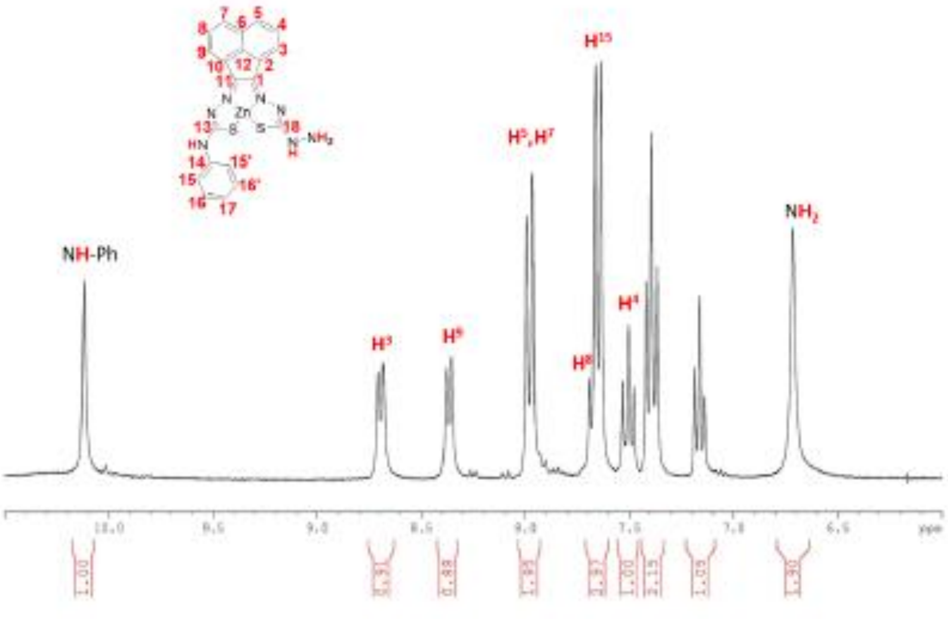

Figure S6. ^{1}H NMR spectrum (500 MHz, DMSO-d$_6$) of for the complex denoted ZnBTSC3.

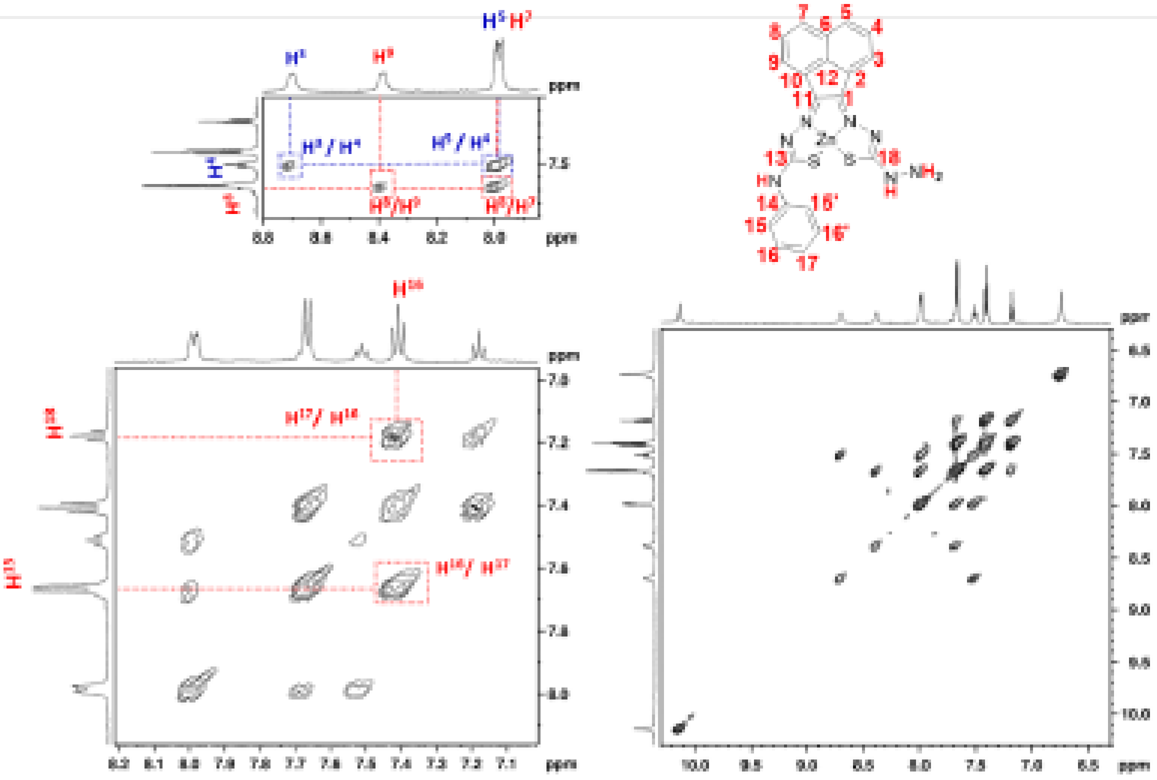

Figure S7. ^{1}H COSY NMR spectrum (500 MHz, DMSO-d$_6$) for the complex denoted ZnBTSC3

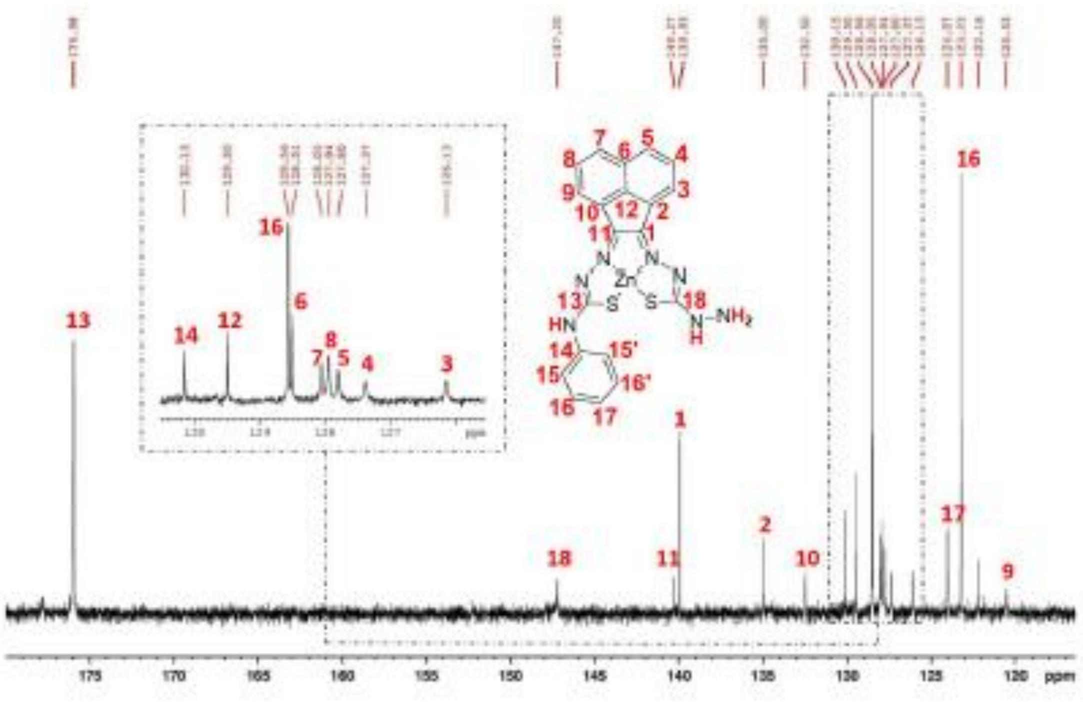

Figure S8. $^{13}C\{^{1}H\}$ NMR spectrum (500 MHz, DMSO-d_6) for the ZnBTSC3 complex.

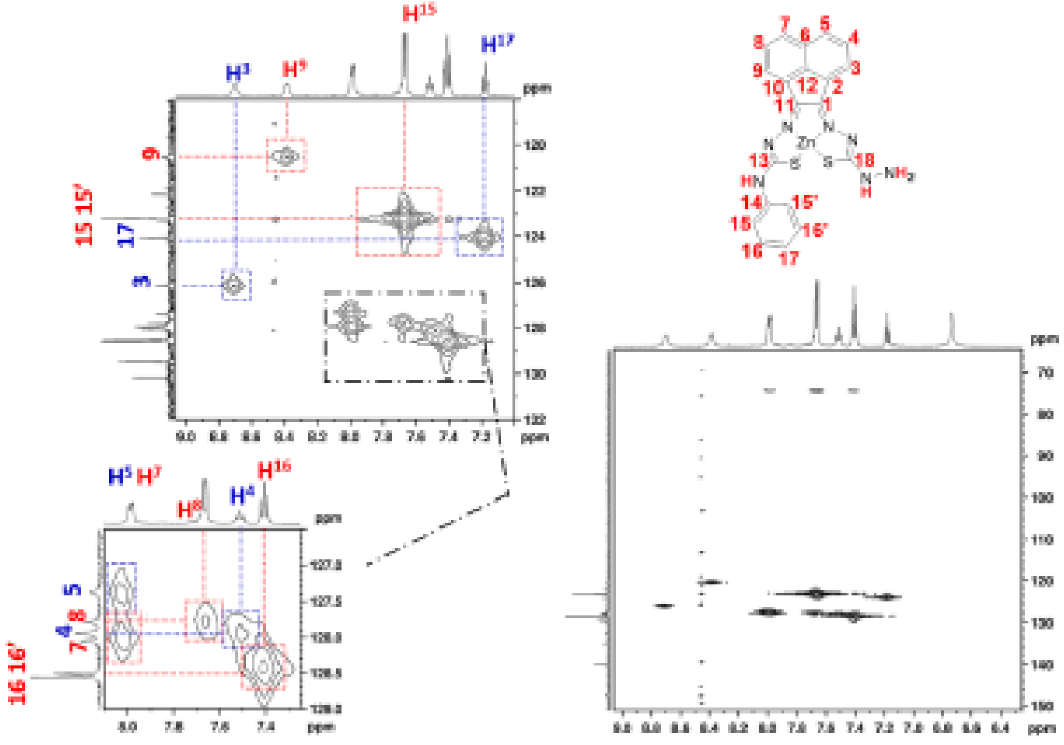

Figure S9. ^{1}H-^{13}C HSQC NMR spectrum (500MHz, DMSO-d_6) for the complex ZnBTSC3.

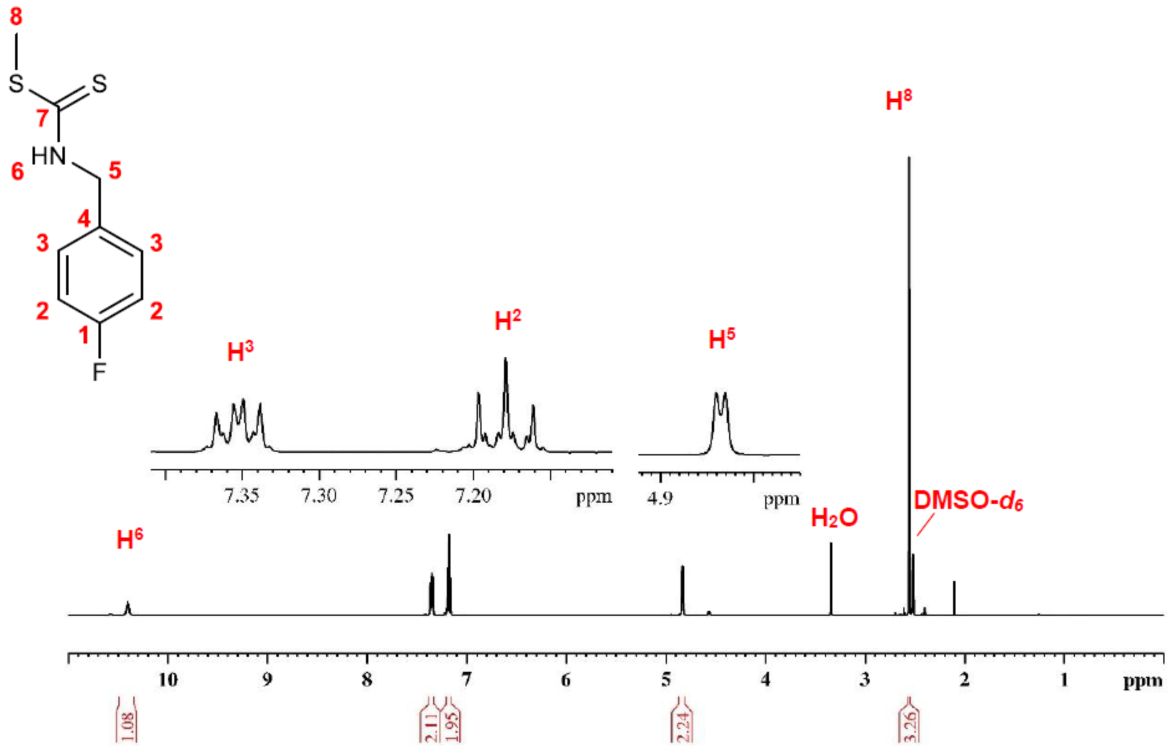

Figure S10. ^{1}H NMR spectra (500 MHz, DMSO-d$_6$, 298 K) for the precursor used in the synthesis of ZnBTSC4.

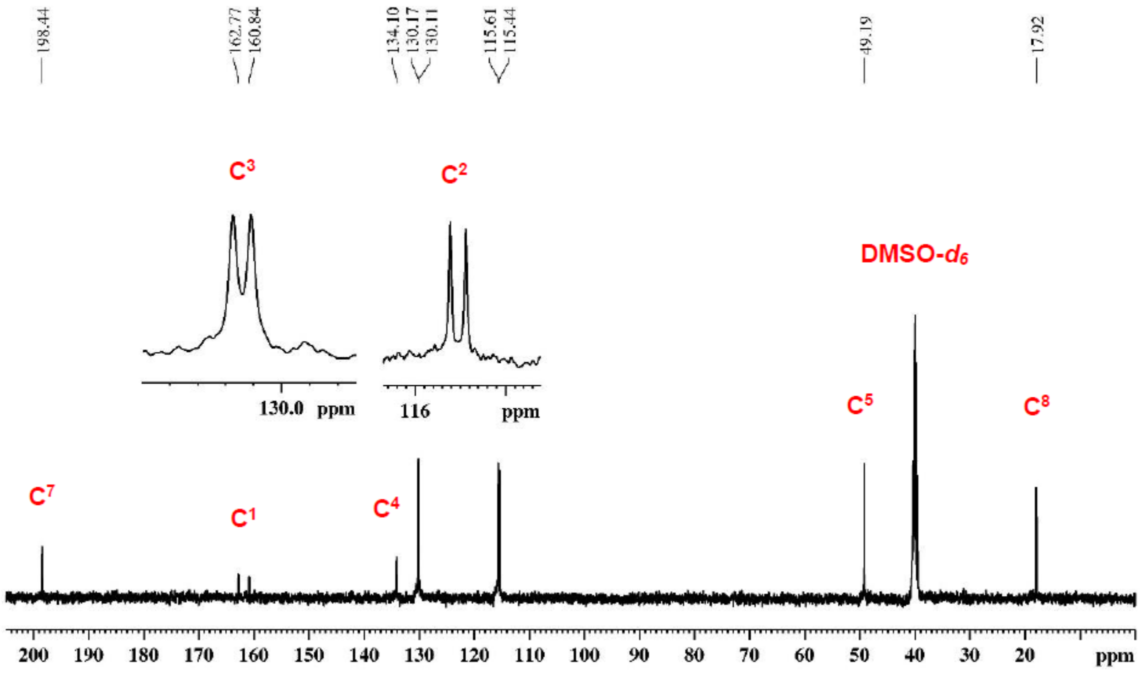

Figure S11. $^{13}C\{^{1}H\}$ NMR spectra (126 MHz, DMSO-d$_6$, 298 K) for the precursor used in the synthesis of ZnBTSC4.

.

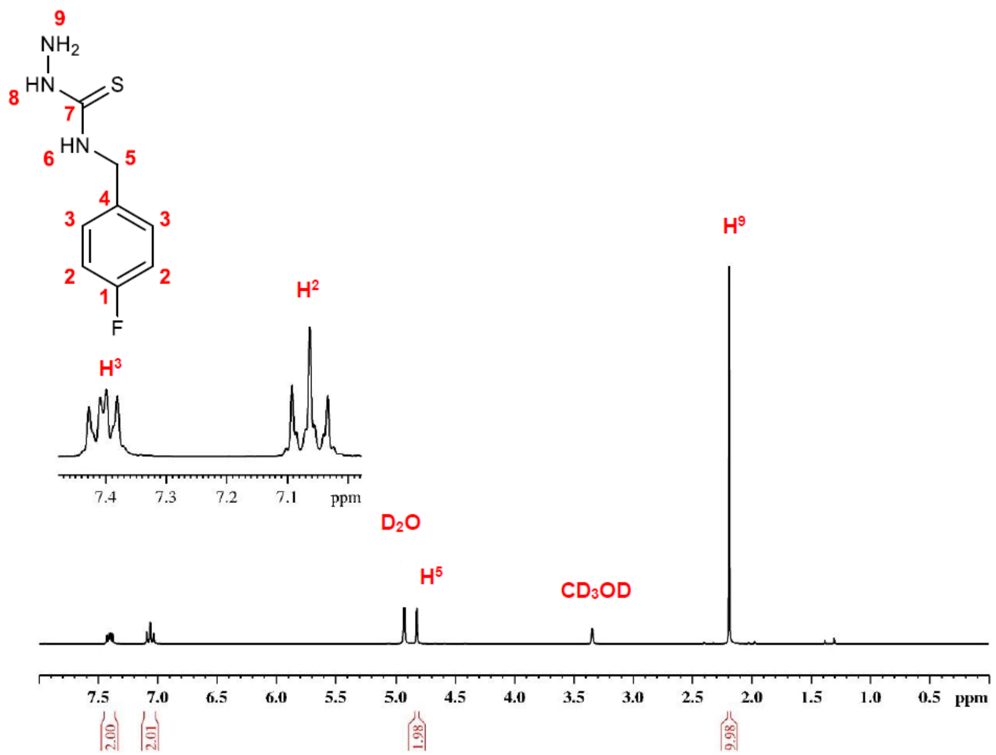

Figure S12. ¹H NMR spectra (300 MHz, Methanol-d4, 298 K) of N-(4-fluorobenzyl) hydrazinecarbothioamide, the thiosemicarbazide precursor used in the synthesis of ZnBTSC4.

.

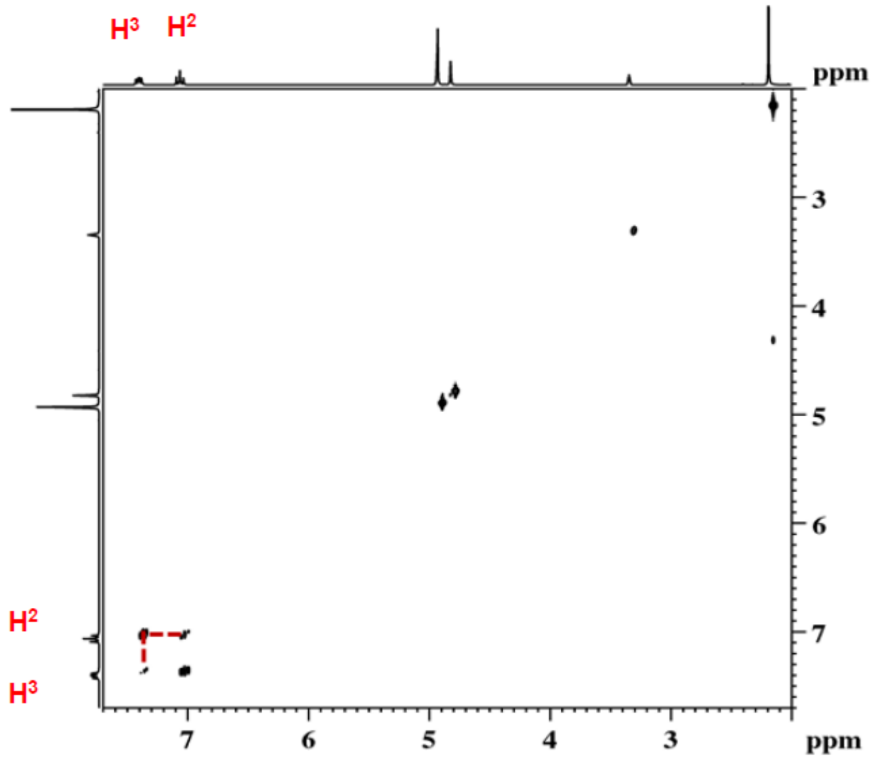

Figure S13. COSY NMR spectra (300 MHz, Methanol-d4, 298 K) of N-(4-fluorobenzyl) hydrazinecarbothioamide, the thiosemicarbazide precursor used in the synthesis of ZnBTSC4.

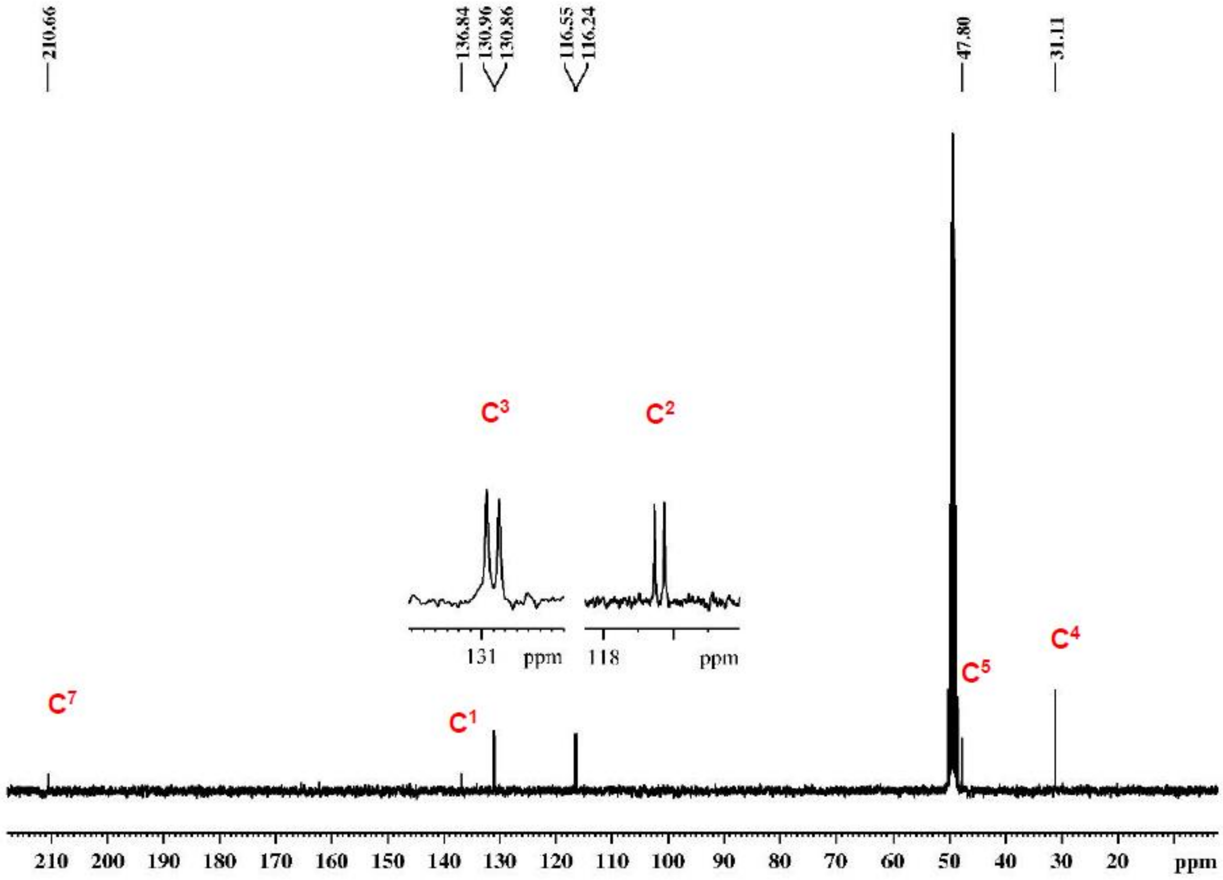

Figure S14. ^{13}C NMR spectra (75 MHz, Methanol-d$_4$, 298 K) of N-(4-fluorobenzyl) hydrazinecarbothioamide, the thiosemicarbazide precursor used in the synthesis of ZnBTSC4.

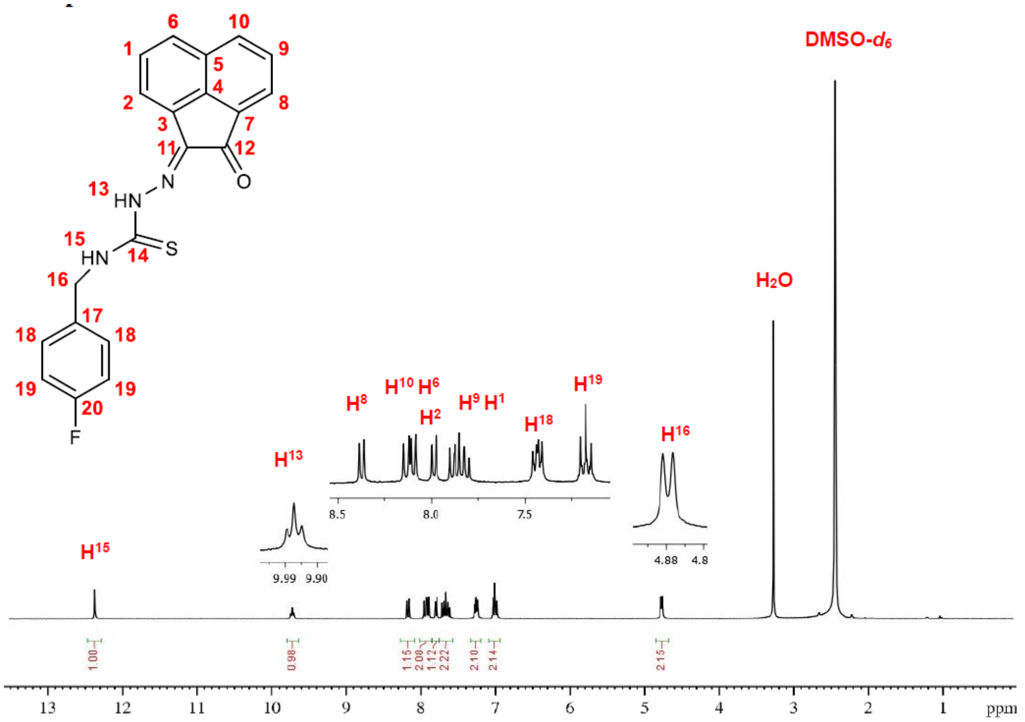

Figure S15. ^{1}H NMR spectra (300 MHz, DMSO-d$_6$, 298 K) of methyl(4-fluorobenzyl)-thiosemicarbazone acenapthenequinone.

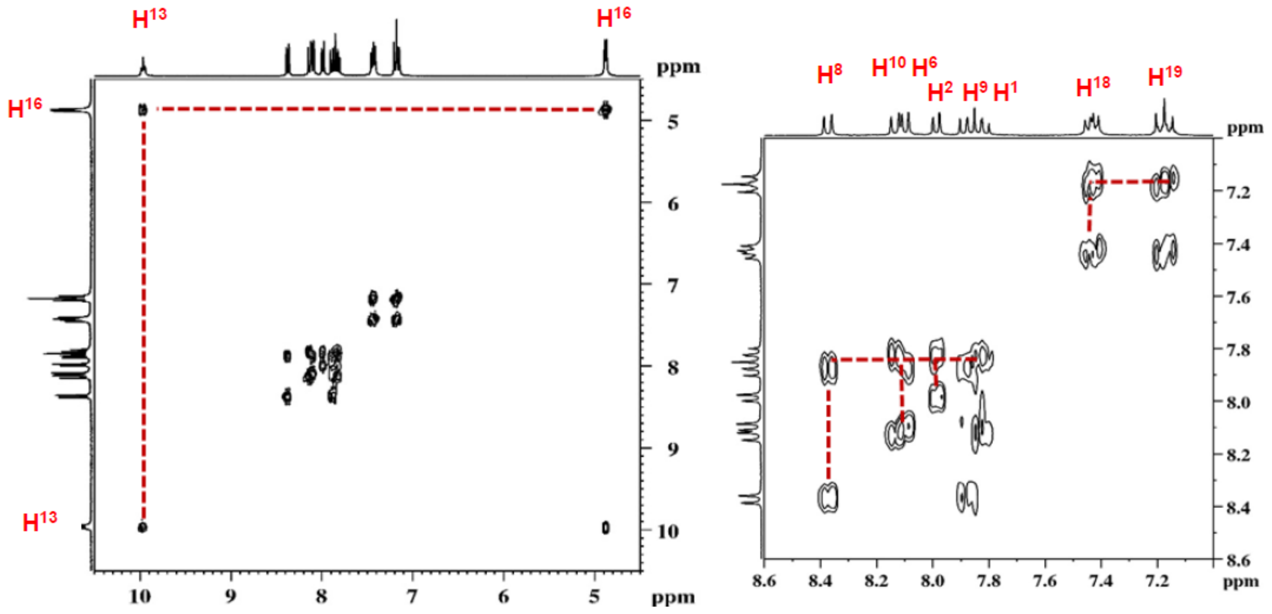

Figure S16. COSY NMR spectra (300 MHz, DMSO-d$_6$, 298 K) of of methyl(4-fluorobenzyl)-thiosemi-carbazone acenapthenequinone.

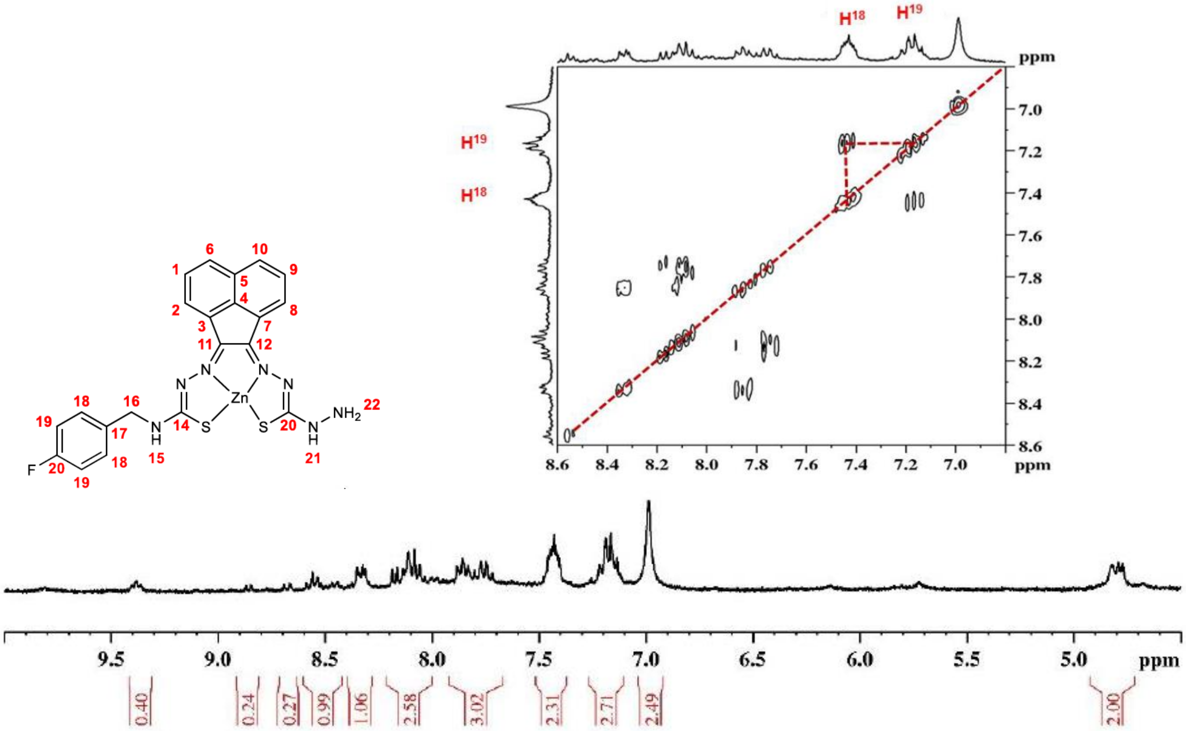

Figure S17. ^{1}H NMR spectra (300 MHz, DMSO-d$_6$, 298 K) Zinc(II) 4-fluorobenzyl-3-thiosemicarba-zone-thiocarbohydrazide acenaphthenoquinone and Inset: COSY NMR spectra (300 MHz, DMSO-d$_6$, 298 K) of **ZnBTSC4**.

Selected characterization data for glycosyl coumarin (GC) derivatives

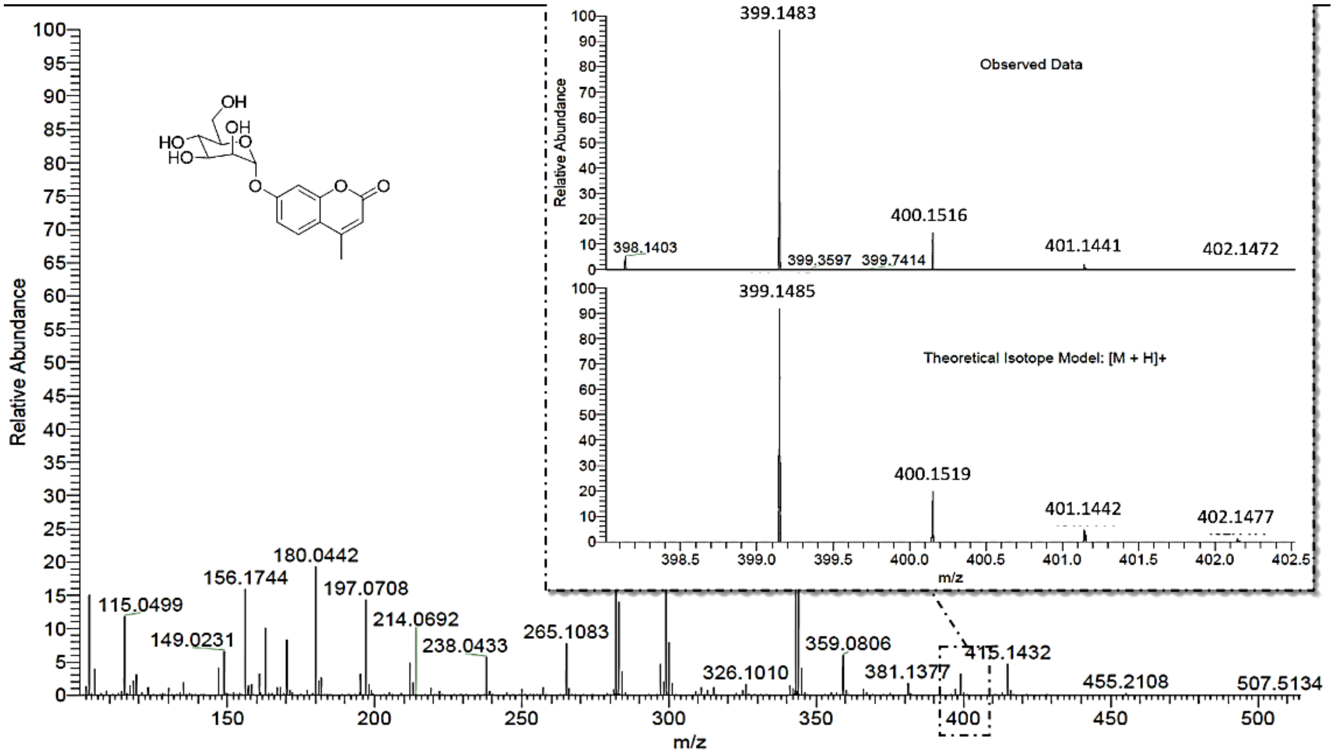

Figure S18. MALDI analysis mass spectrometry for 4-Methylumbelliferyl-7-yl-α-D-mannopyranose (GC, **compound 4**).

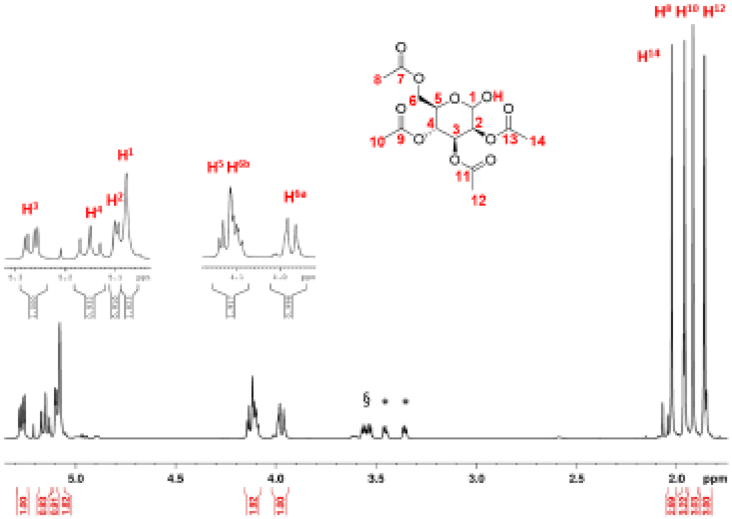

Figure S19. ^{1}H NMR spectra (500 MHz, $CDCl_3$) presenting the 2,3,4,6-Tetra-O-acetyl-D-mannopyranose precursor.

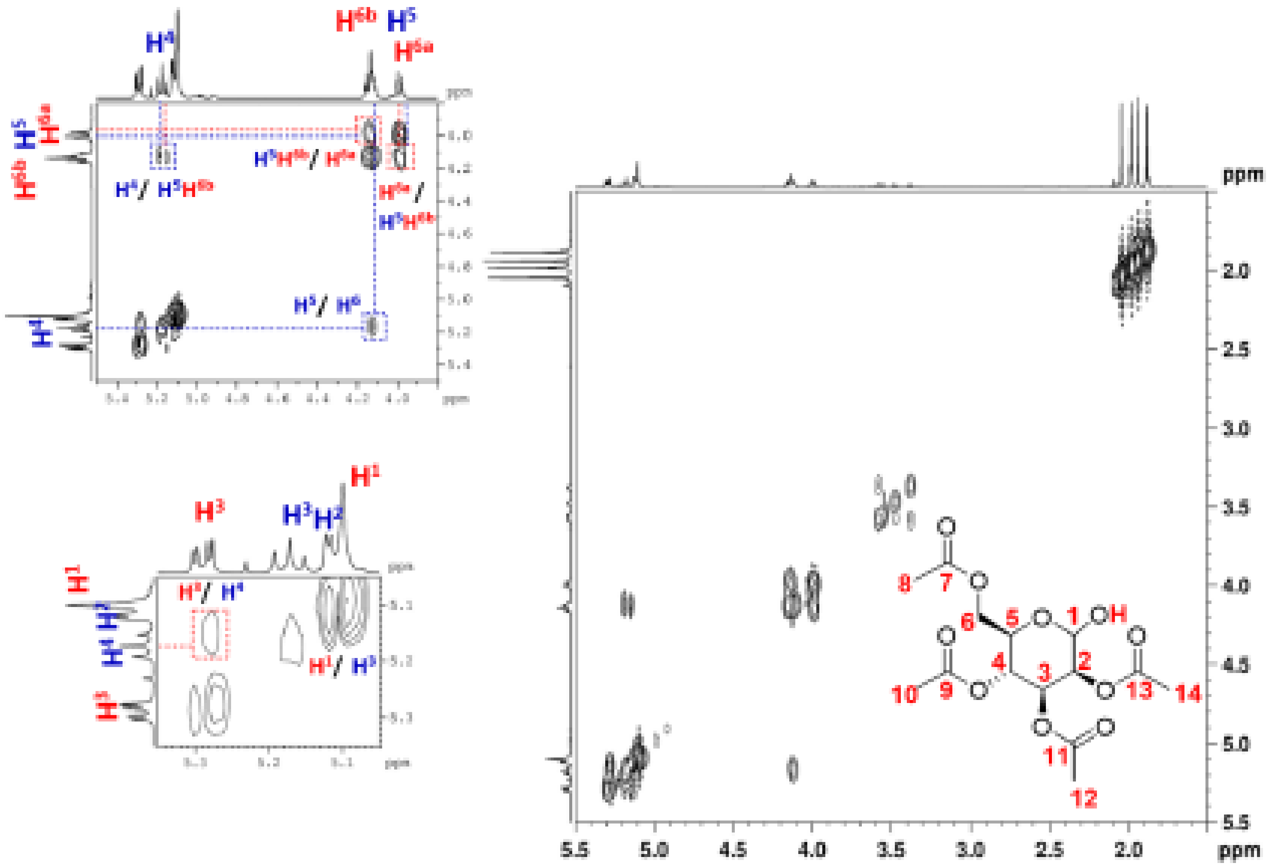

Figure S20. ^{1}H-^{1}H COSY NMR spectra (500 MHz, CDCl₃) presenting the 2,3,4,6-tetra-O-acetyl-D-mannopyranose.

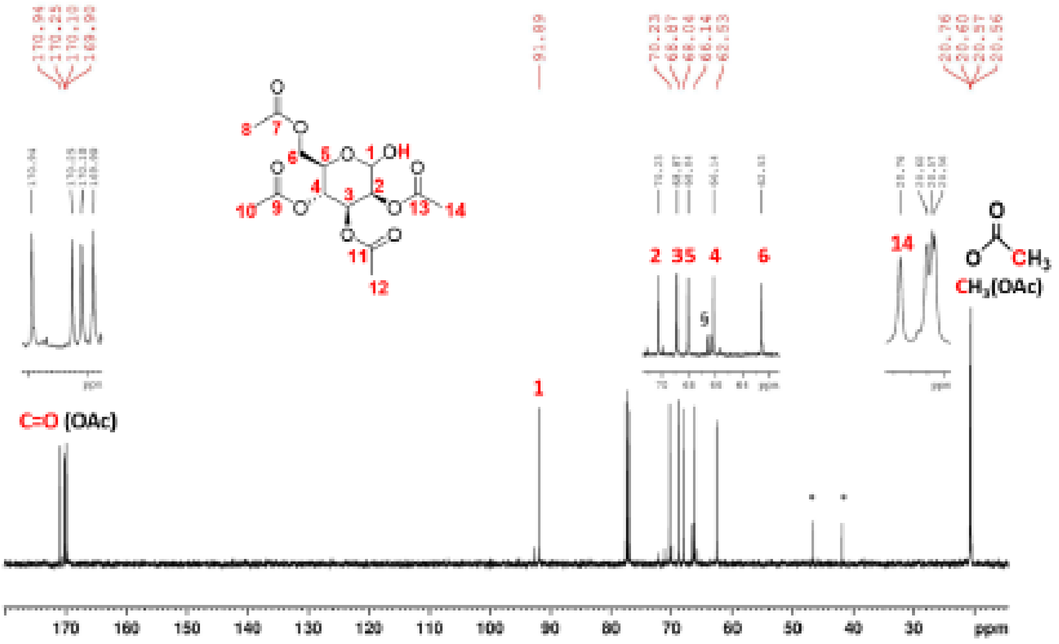

Figure S21. ^{13}C{^{1}H} NMR spectra (500 MHz, CDCl₃) presenting the 2,3,4,6-tetra-O-acetyl-D-manno-pyranose.

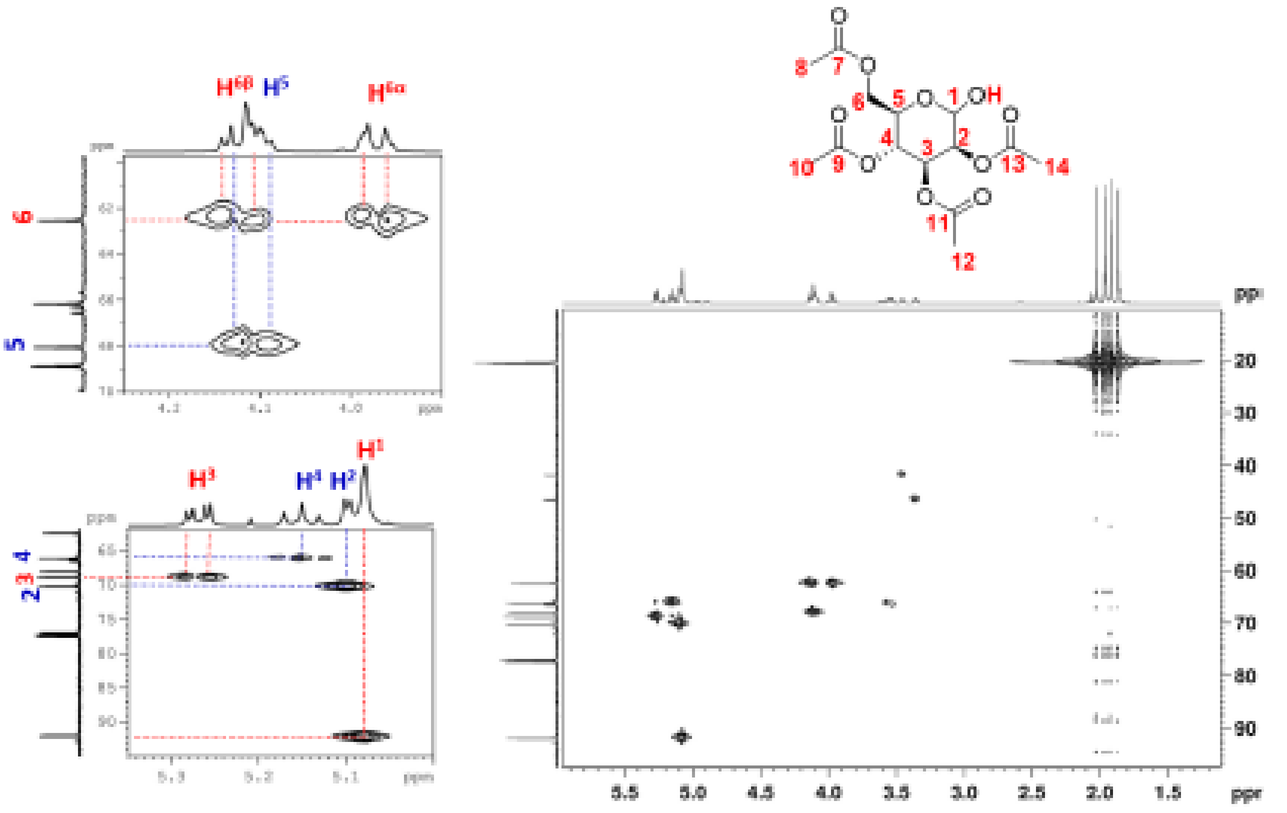

Figure S22. ^{1}H-^{13}C HSQC NMR spectra (500 MHz, $CDCl_3$) of 2,3,4,6-tetra-O-acetyl-D-mannopyranose.

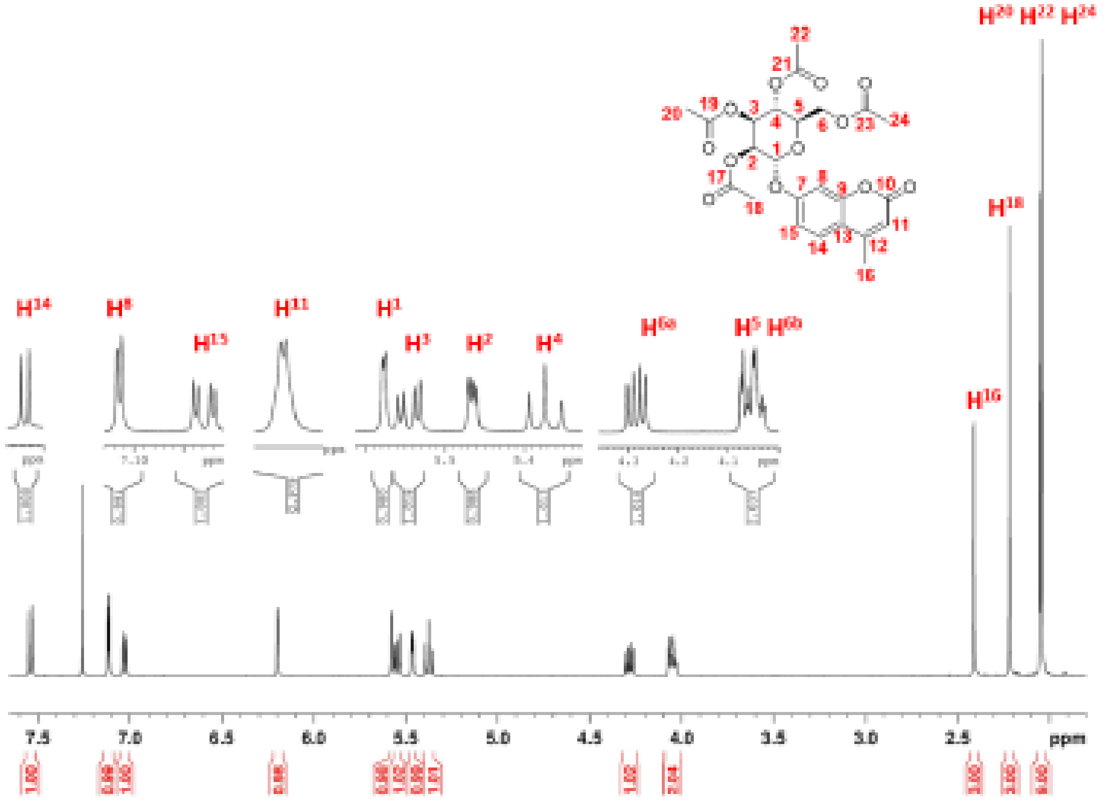

Figure S23. ^{1}H NMR spectra (500 MHz, $CDCl_3$) of 4- Methylumbelliferyl-7-yl-2,3,4,6-tetra-O-acetyl-α -D-mannopyranose (AcGC, Compound **5**).

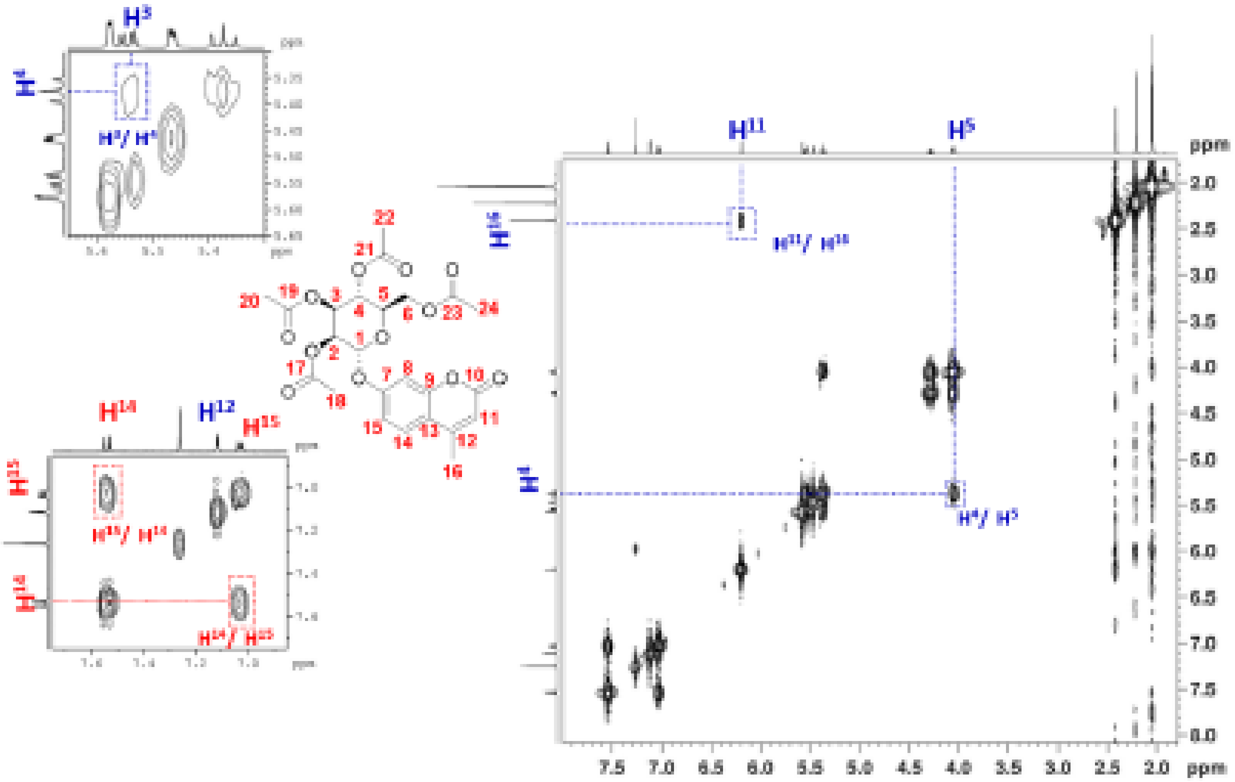

Figure S24. COSY NMR spectra (500 MHz, $CDCl_3$) of 4- Methylumbelliferyl-7-yl-2,3,4,6-Tetra-O-acetyl- α -D-mannopyranose (AcGC, **5**).

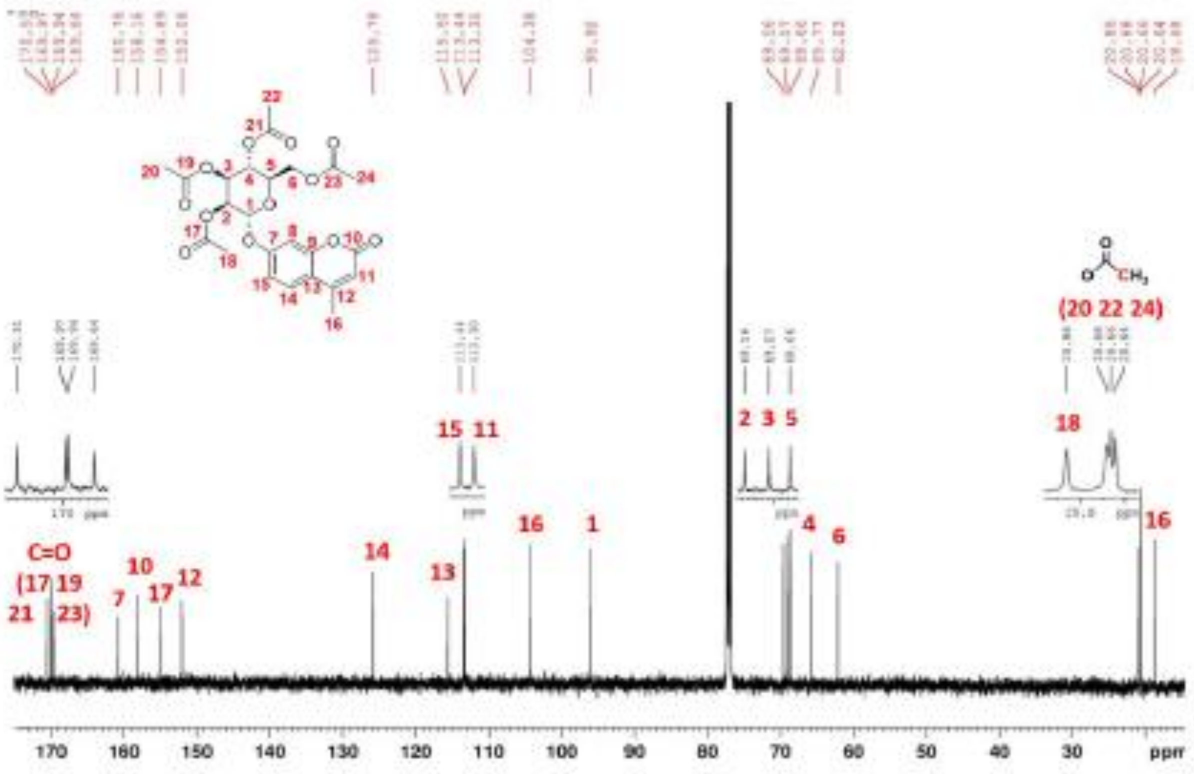

Figure S25. $^{13}C\{^{1}H\}$ NMR spectra (500 MHz, $CDCl_3$) of 4- Methylumbelliferyl-7-yl-2,3,4,6-tetra-O-acetyl- α -D-mannopyranose (AcGC, **5**).

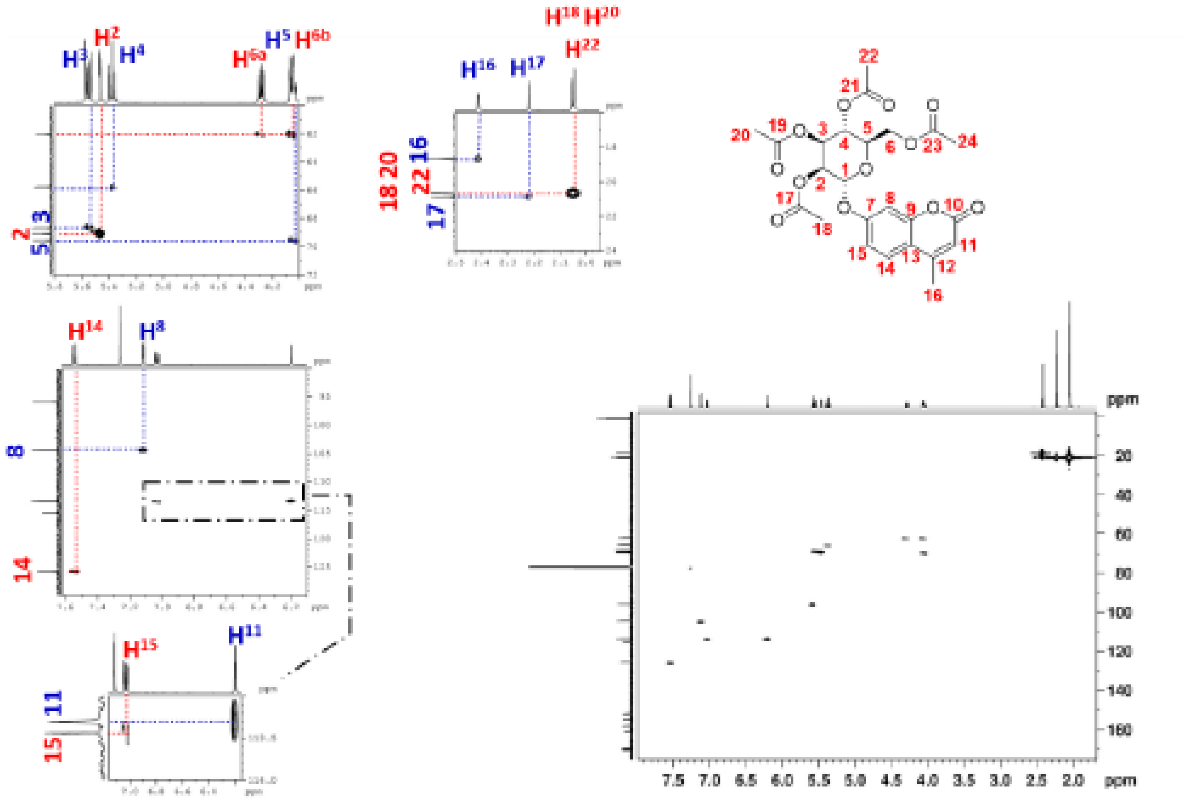

Figure S26. ^{1}H-^{13}C HSQC NMR spectra (500 MHz, $CDCl_3$) of 4- Methylumbelliferyl-7-yl-2,3,4,6-Tetra-O-acetyl- α -D-mannopyranose (AcGC, **5**).

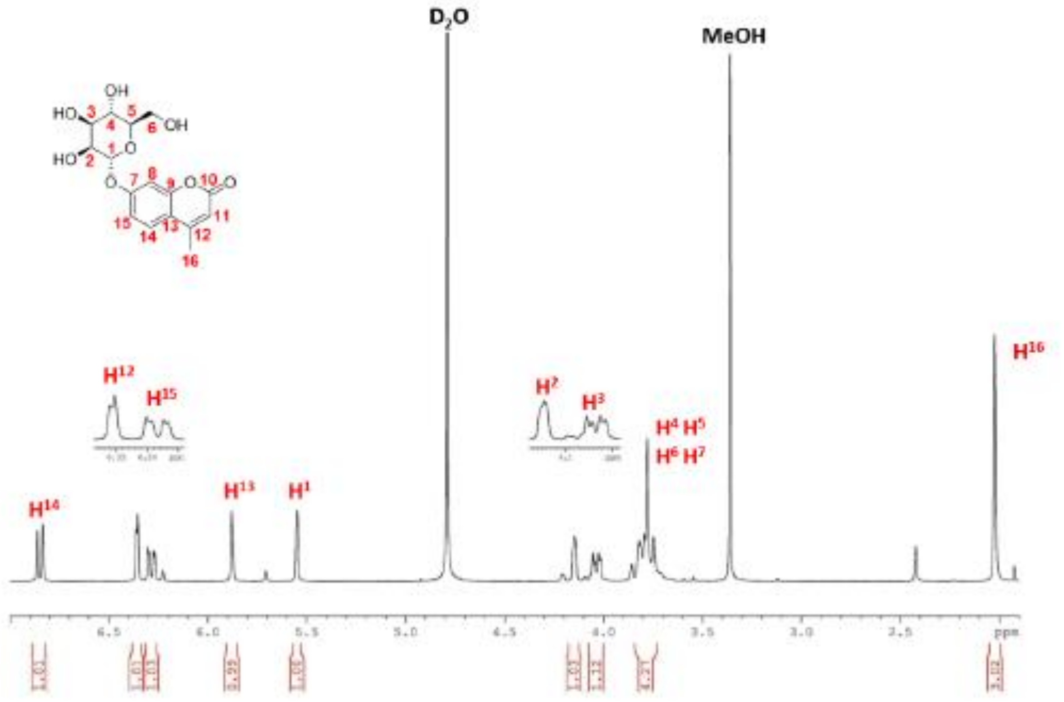

Figure S27. ^{1}H NMR spectra (500 MHz, MeOD) of 4- Methylumbelliferyl-7-yl-α-D-mannopyranose (GC, **4**).

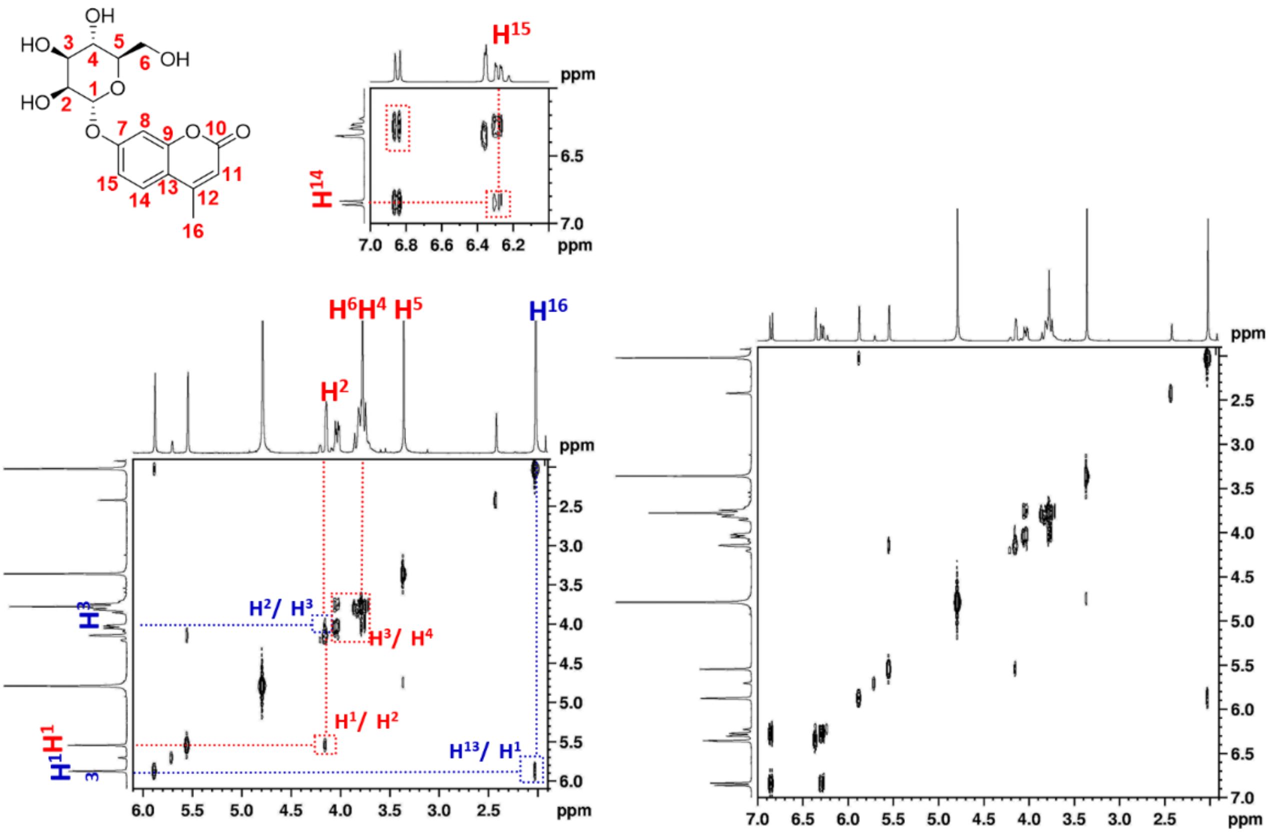

Figure S28. ^{1}H COSY NMR spectra (500 MHz, MeOD) of 4- Methylumbelliferyl-7-yl - α -D-manno-pyranose (GC, **4**).

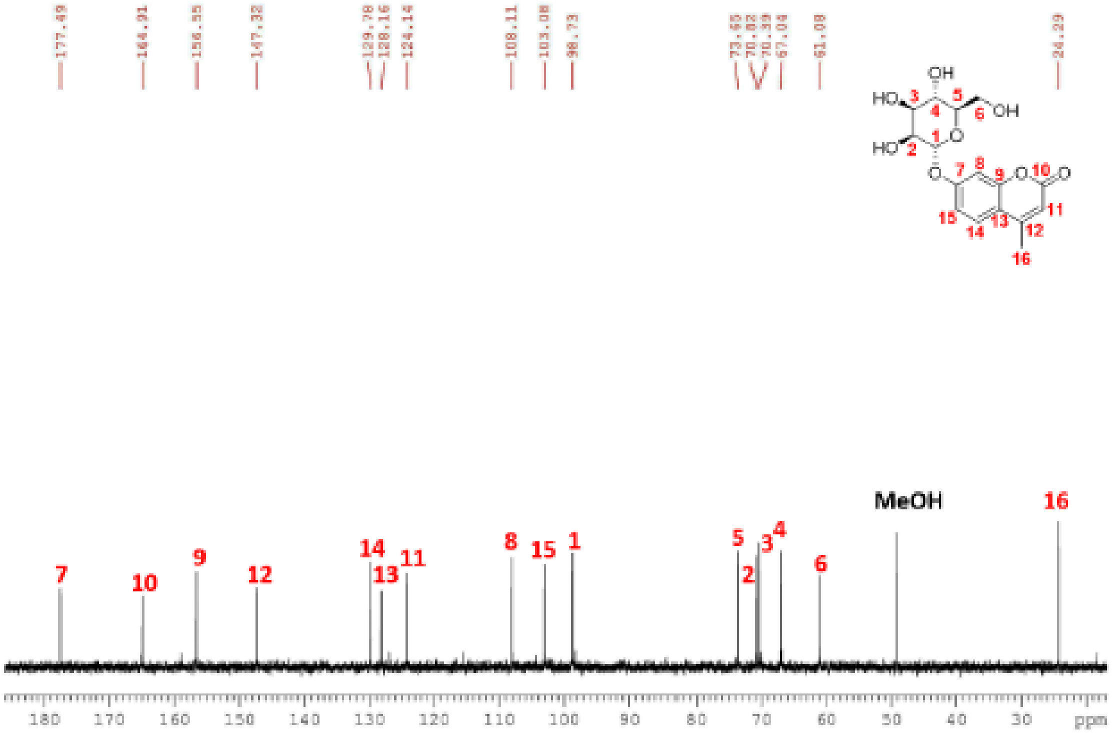

Figure S29. ^{13}C NMR spectra (500 MHz, MeOD) of 4- Methylumbelliferyl-7-yl-α-D-mannopyranose (GC, **4**).

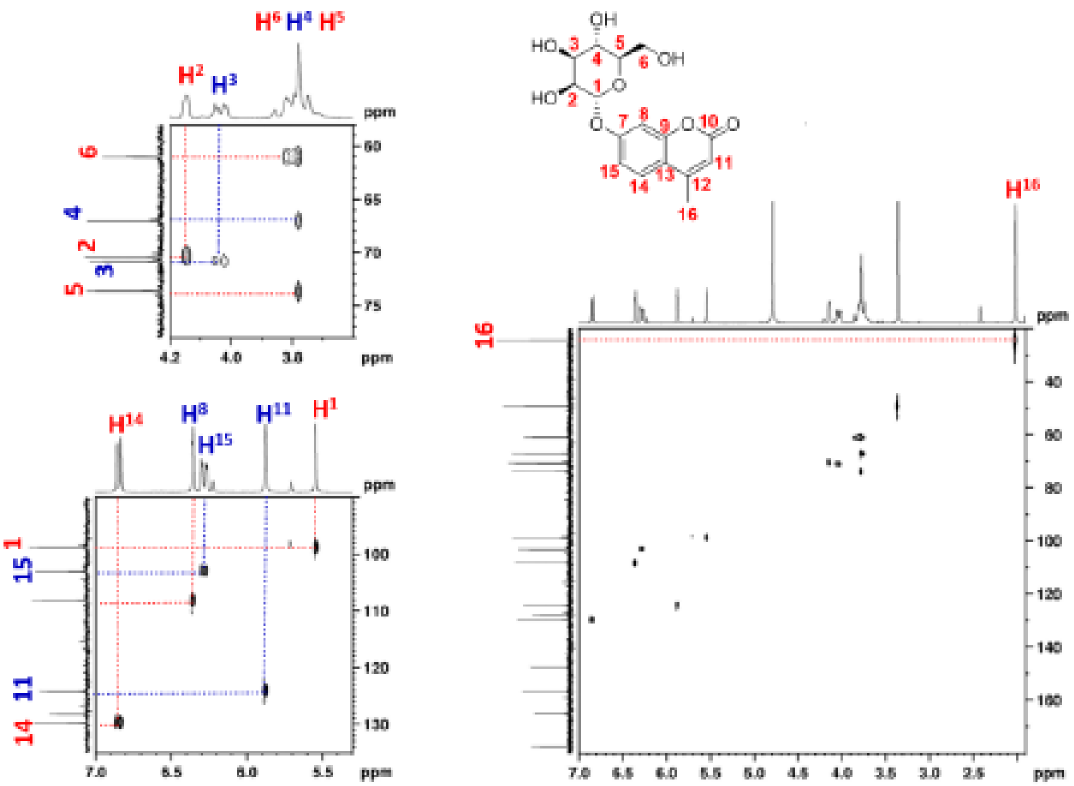

Figure S30. ^{1}H-^{13}C HSQC NMR spectra (500 MHz, MeOD) of 4- Methylumbelliferyl-7-yl -α-D-mannopyranose (GC, **4**).

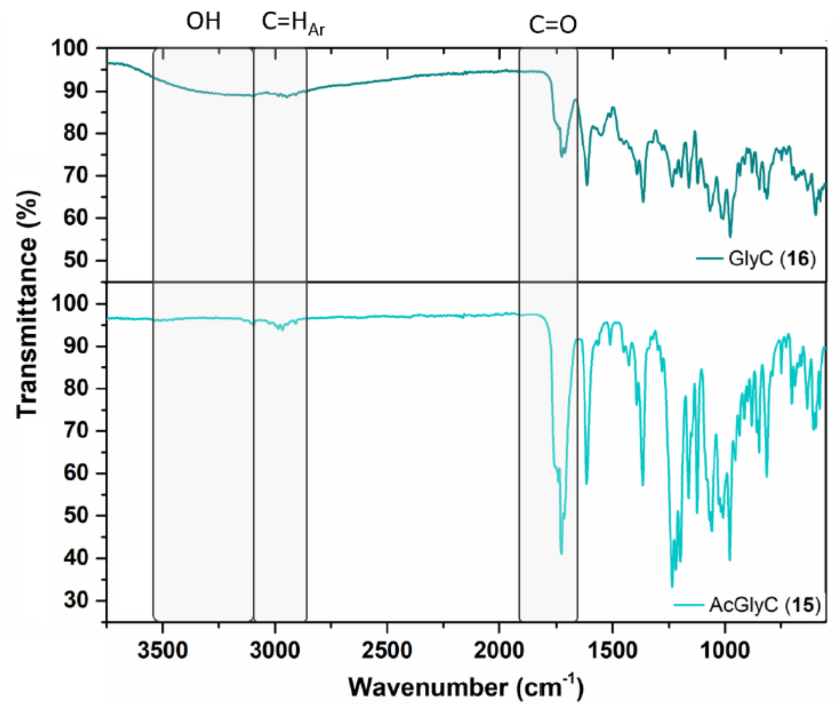

Figure S31. Comparative solid state IR spectra for the tetracetyl-glycosyl coumarin (denoted **AcGC) (5**) (bottom) and glycosyl coumarin (denoted **GC**) (**4**) (top).

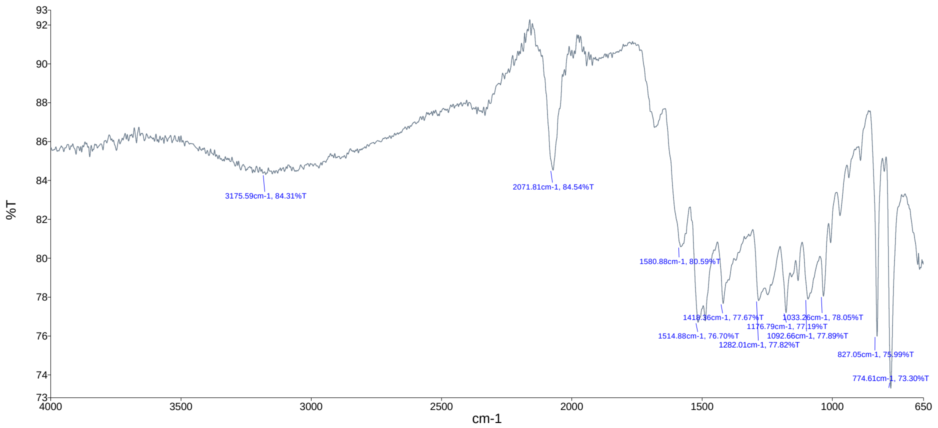

(a)

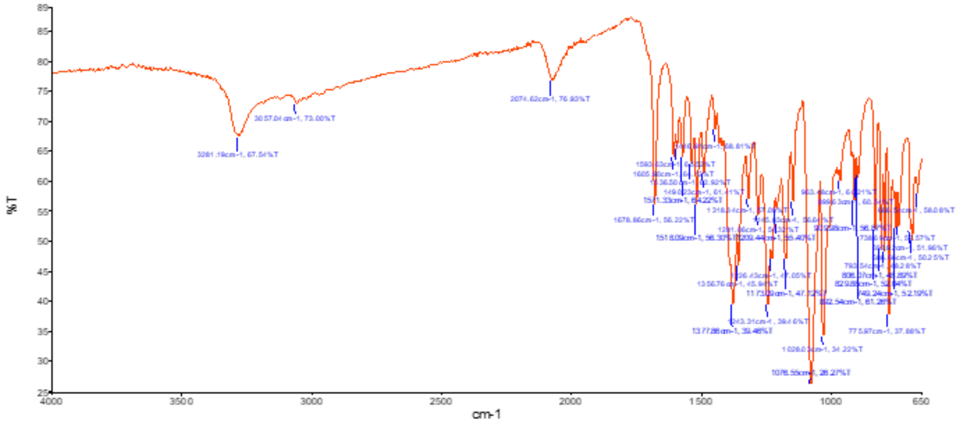

(b)

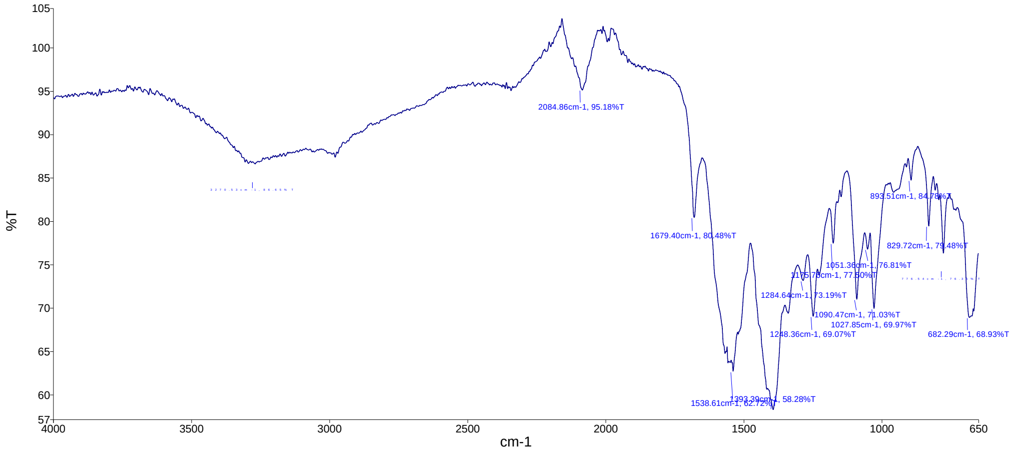

(c)

Figure S32. Comparative solid-state IR spectra of compounds ZnBTSC2 (a), ZnBTSC3 (b) and ZnBTSC4 (c).

Selected UV-Vis spectroscopy titration data.

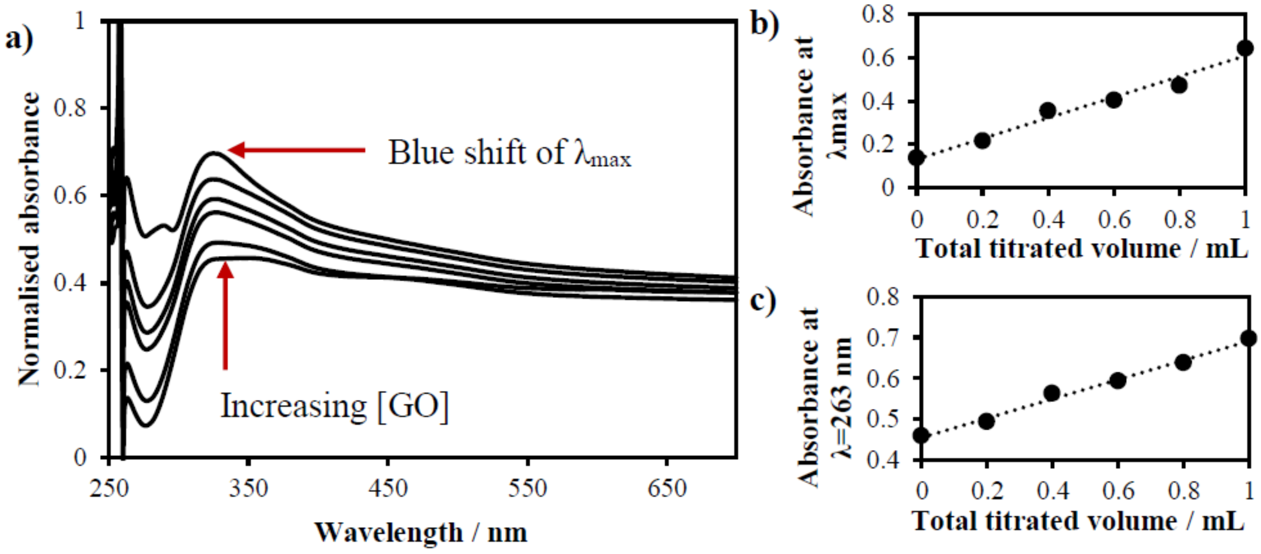

Figure S33. a) UV/Vis titrations of **ZnBTSC1** from a stock solution (1.15 mM **compound ZnBTSC1** in DMSO, solid line). The increased absorbance with consecutive titrations of a duplicate solution saturated with GO (dashed line) was observed. b) Absorbance at λmax with each 0.2 mL titre increased linearly with GO content. c) Absorbance at λ = 258 nm (local maxima for GO) with each 0.2 mL titre increases linearly with the GO content.

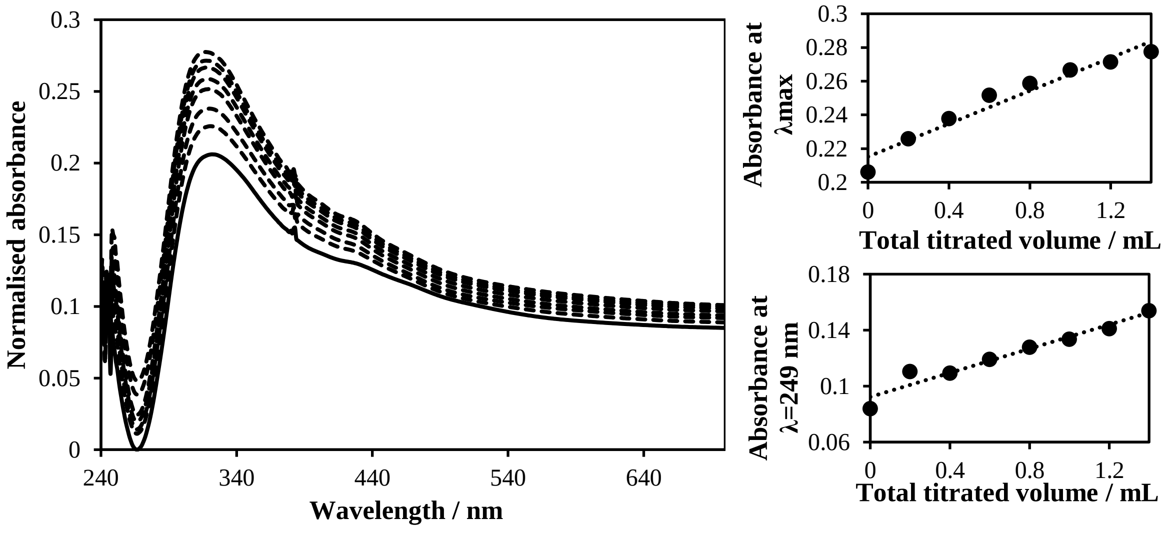

Figure S34. a) UV/Vis titration of **Compound ZnBTSC2,** (solid line, 20 uM in DMSO). Experiment showed the increased absorbance with consecutive titrations (in duplicate), where solutions were saturated with GO (dashed line). b) Absorbance at λ_{max} with each 0.2 mL titre increases linearly with GO content. c) Absorbance at λ = 249 nm (local maxima for GO) with each 0.2 mL titre increases linearly with GO content.

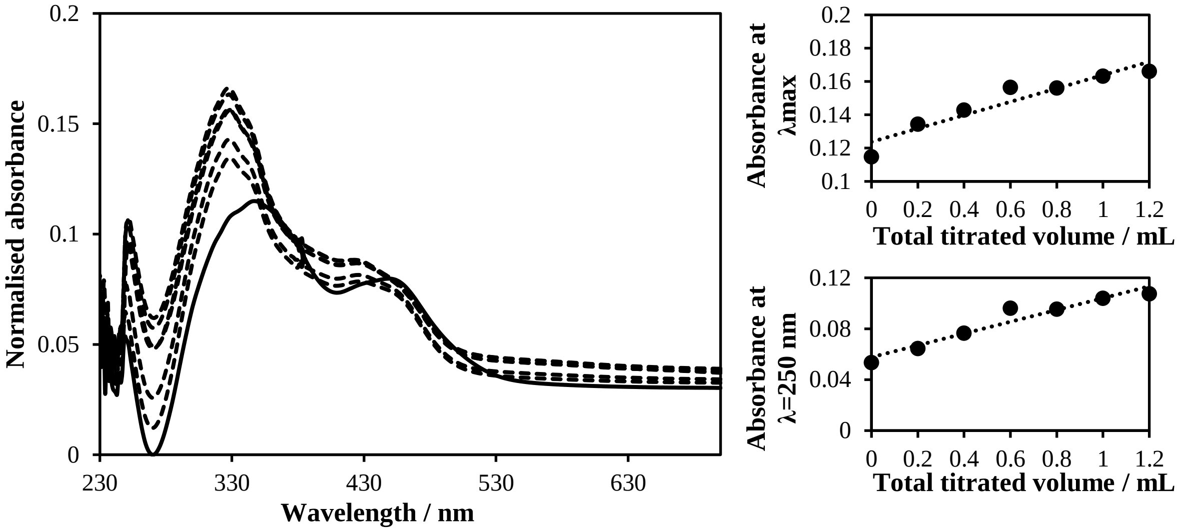

Figure S35. a) UV/Vis titrations of **ZnBTSC3** (solid line, **20 uM**) showing the increased absorbance with consecutive titrations of a duplicate solution saturated with GO (dashed line). b) Absorbance at λ_{max} with each 0.2 mL titre increases linearly with GO content. c) Absorbance at λ = 258 nm (local maxima for GO) with each 0.2 mL titre increases linearly with GO content.

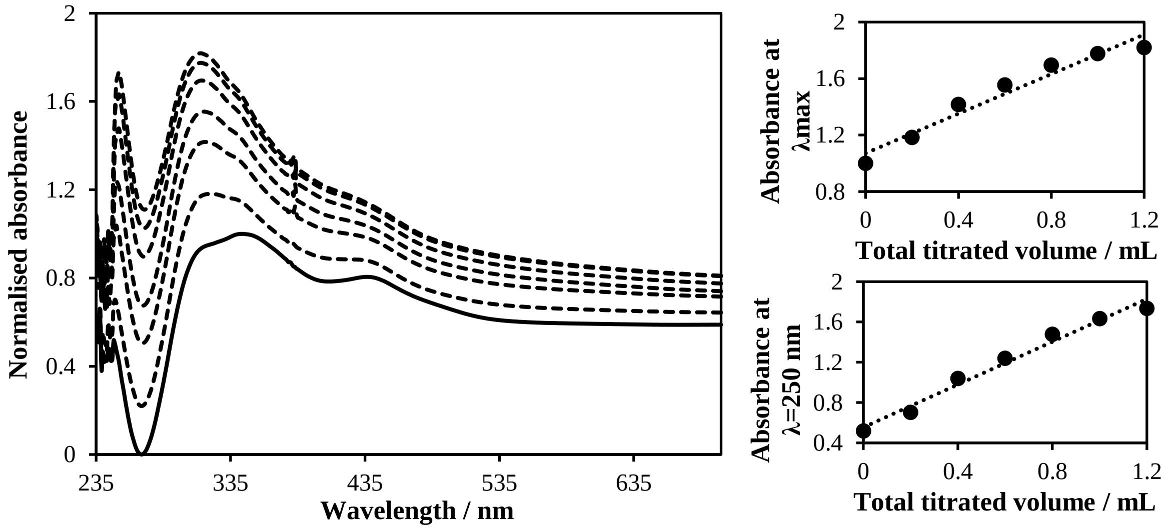

Figure S36. A) UV/Vis titrations of **ZnBTSC4** (solid line, 20 uM in DMSO) showing the increased absorbance with consecutive titrations of a duplicate solution saturated with GO (dashed line). B) Absorbance at λ_{max} with each 0.2 mL titre increases linearly with GO content. C) Absorbance at λ = 250 nm (local maxima for GO) with each 0.2 mL titre increases linearly with GO content.

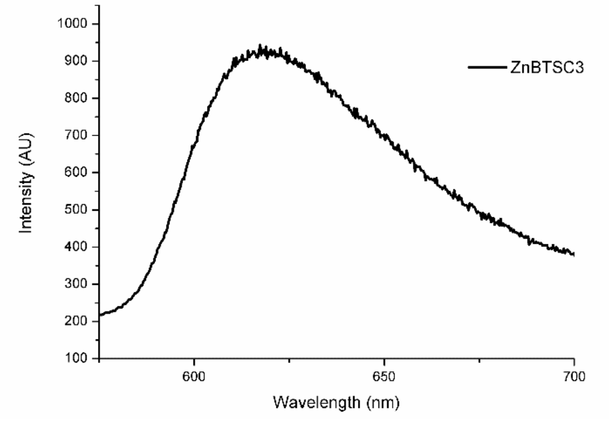

(a)

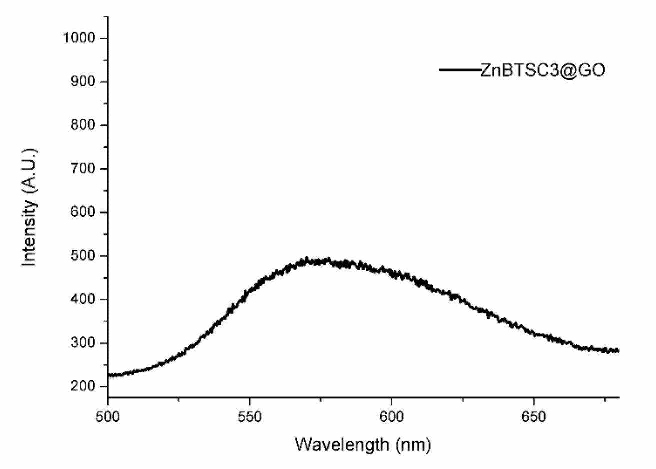

(b)

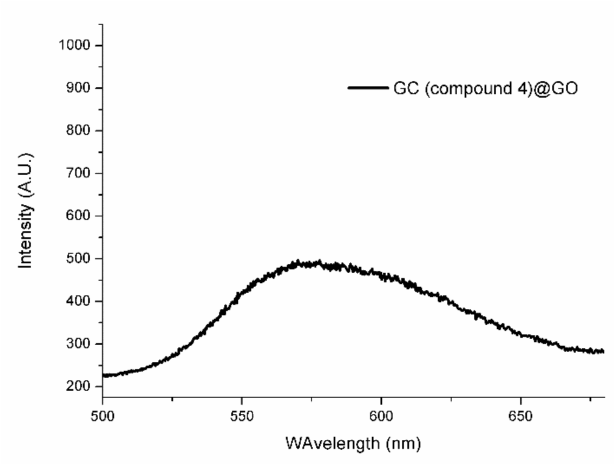

(c)

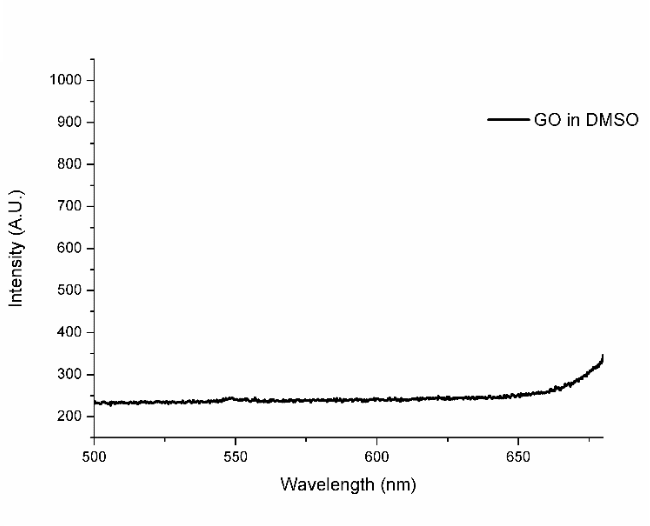

(d)

Figure S37. Two photon fluorescence spectroscopy investigations, excitation 810 nm laser power 2-3 mW: (a) 10 mM solution of ZnBTSC3 (b) ZnBTSC3@GO, 10 mM of ZnBTSC3 in 1mg/mL of GO in DMSO; (c) Coumarin Glucose (compound 4)@GO, 10 mM of ZnBTSC3 in 1mg/mL of GO in DMSO; (d) 1mg/mL of GO in DMSO.

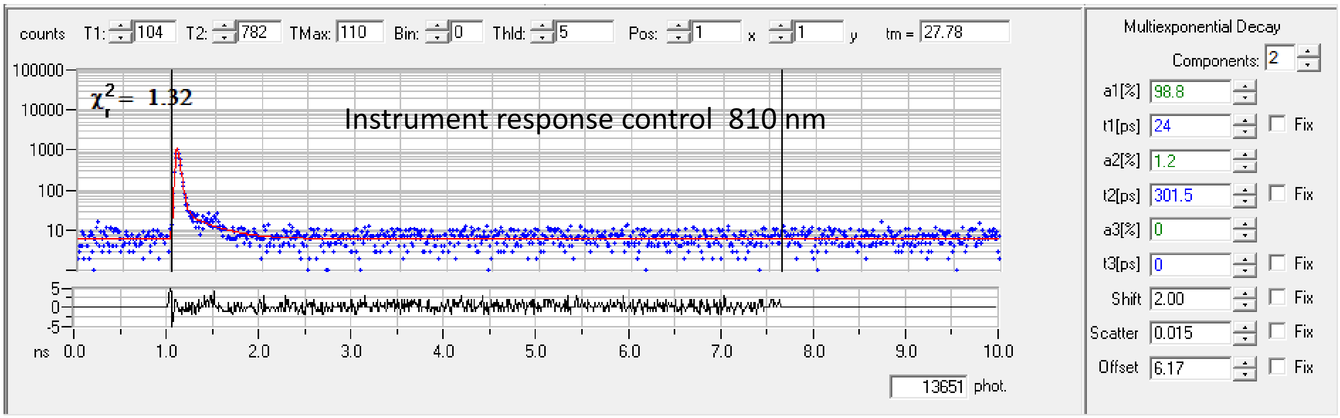

(a)

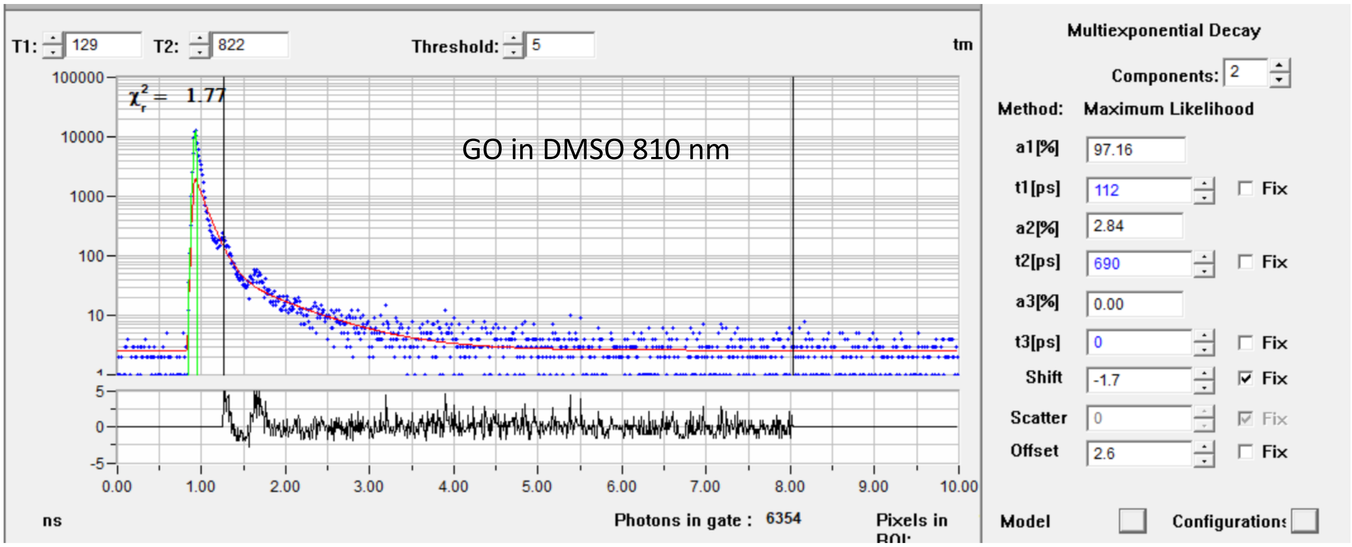

(b)

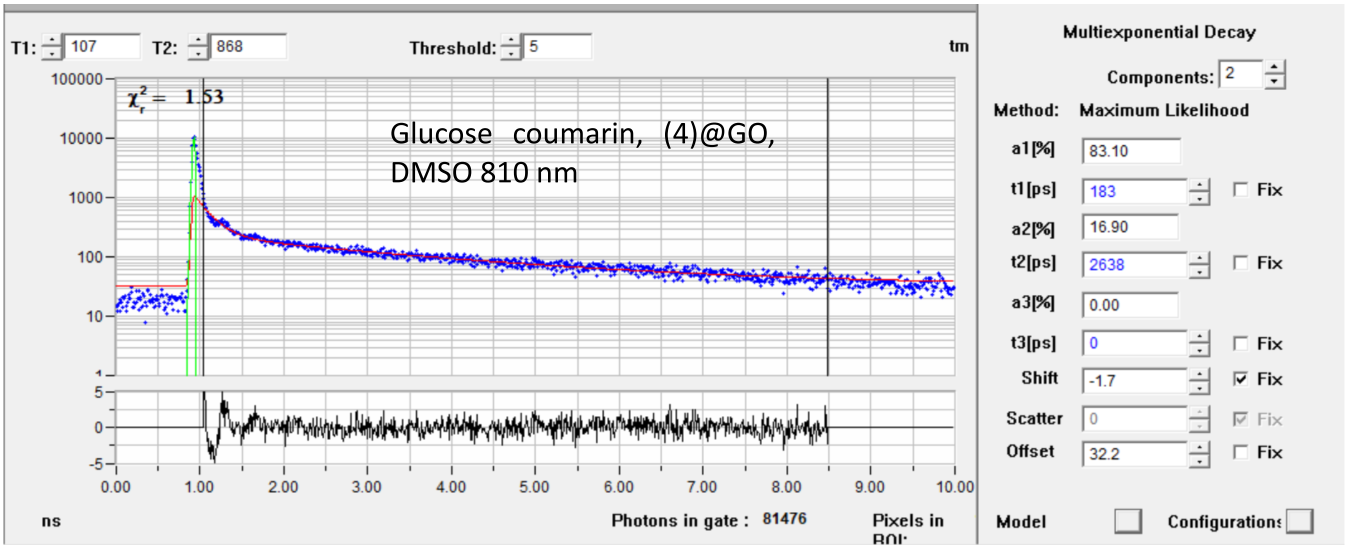

(c)

Figure S38. Two photon fluorescence spectroscopy investigations at 810 nm laser power 2-3 mW: (a) Instrument response (control); (b) 1mg/mL of GO in DMSO; (c) Coumarin Glucose (compound 4)@GO, 10 mM of 4 in 1mg/mL of GO in DMSO.

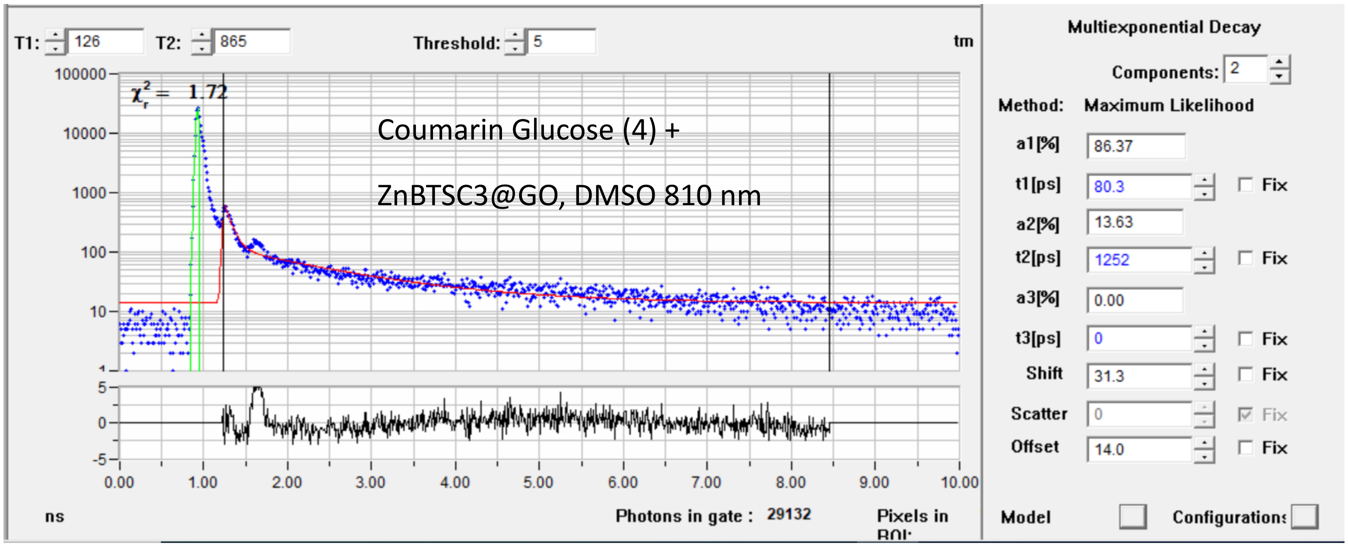

Figure S39. Two photon fluorescence spectroscopy investigations at 810 nm excitation, laser power 2-3 mW: Coumarin Glucose (compound 4)@GO, 10 mM; ZnBTSC3 10 mM in presence of 1mg/mL of GO in DMSO.

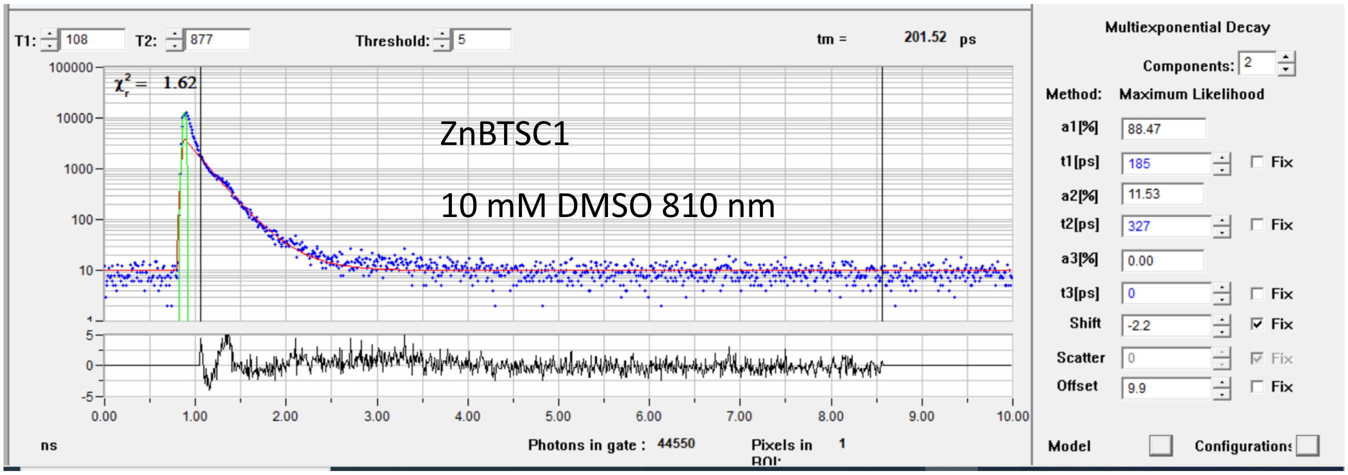

(a)

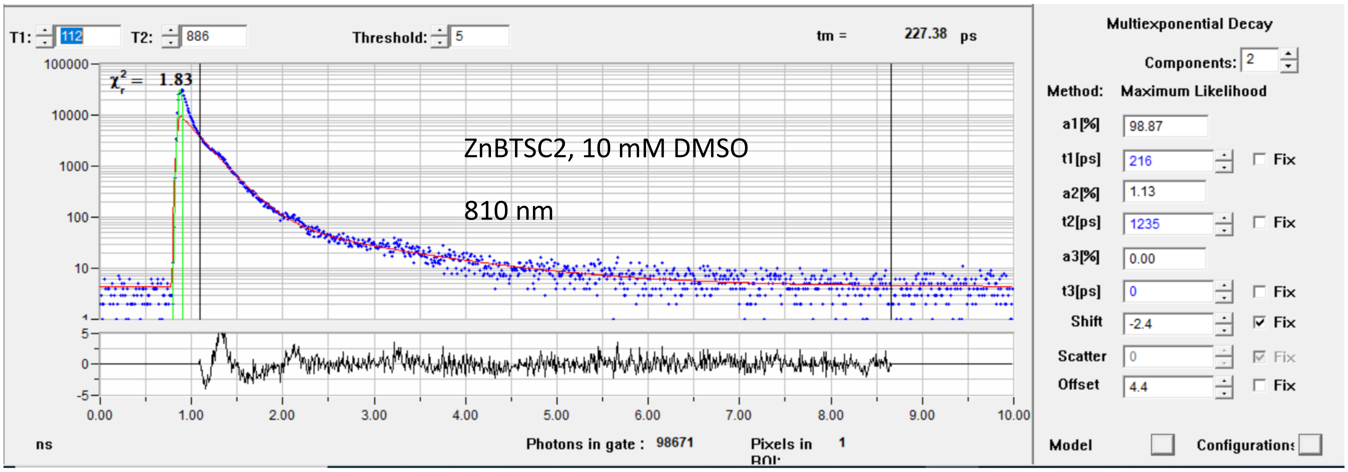

(b)

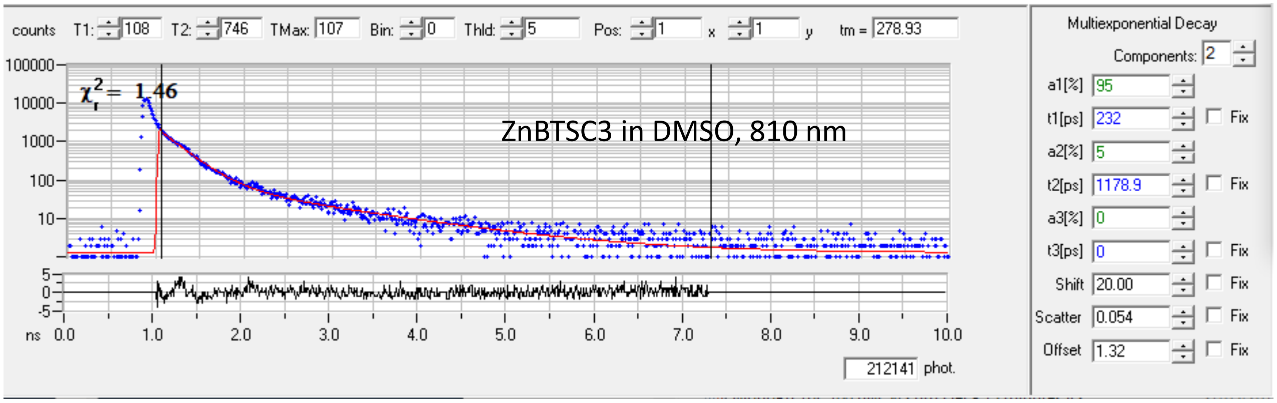

(c)

Figure S40. Two photon fluorescence spectroscopy (TCSPC) data and fitted curves for (a) ZnBTSC1; (b) ZnBTSC2 and (c) ZnBTSC3, each at 10 mM in DMSO, excitation 810 nm laser power 3 mW.

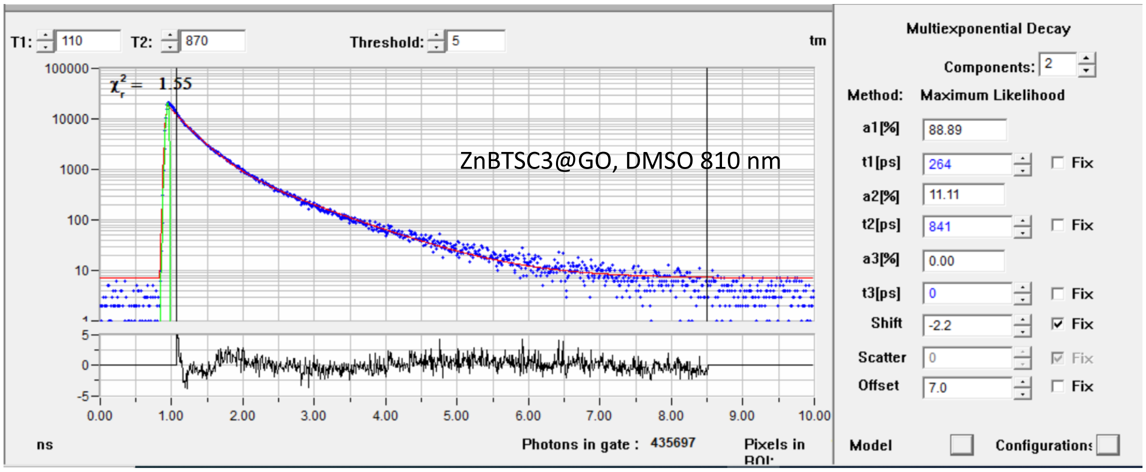

(a)

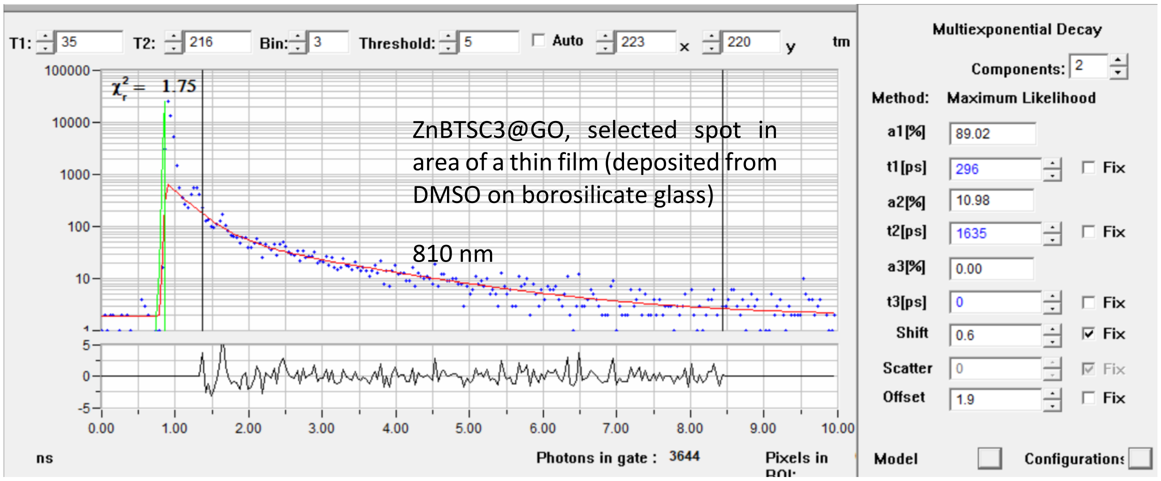

(b)

Figure S41. Two photon fluorescence spectroscopy investigations at 810 nm laser power 2-3 mW: (a) ZnBTSC3@GO, 10 mM of ZnBTSC3 in 1mg/mL of GO in DMSO; (b) TCSPC data and fitting corresponding to a selected spot - ZnBTSC3@GO, 10 mM of ZnBTSC3 in 1mg/mL of GO deposited on borosilicate glass from DMSO;

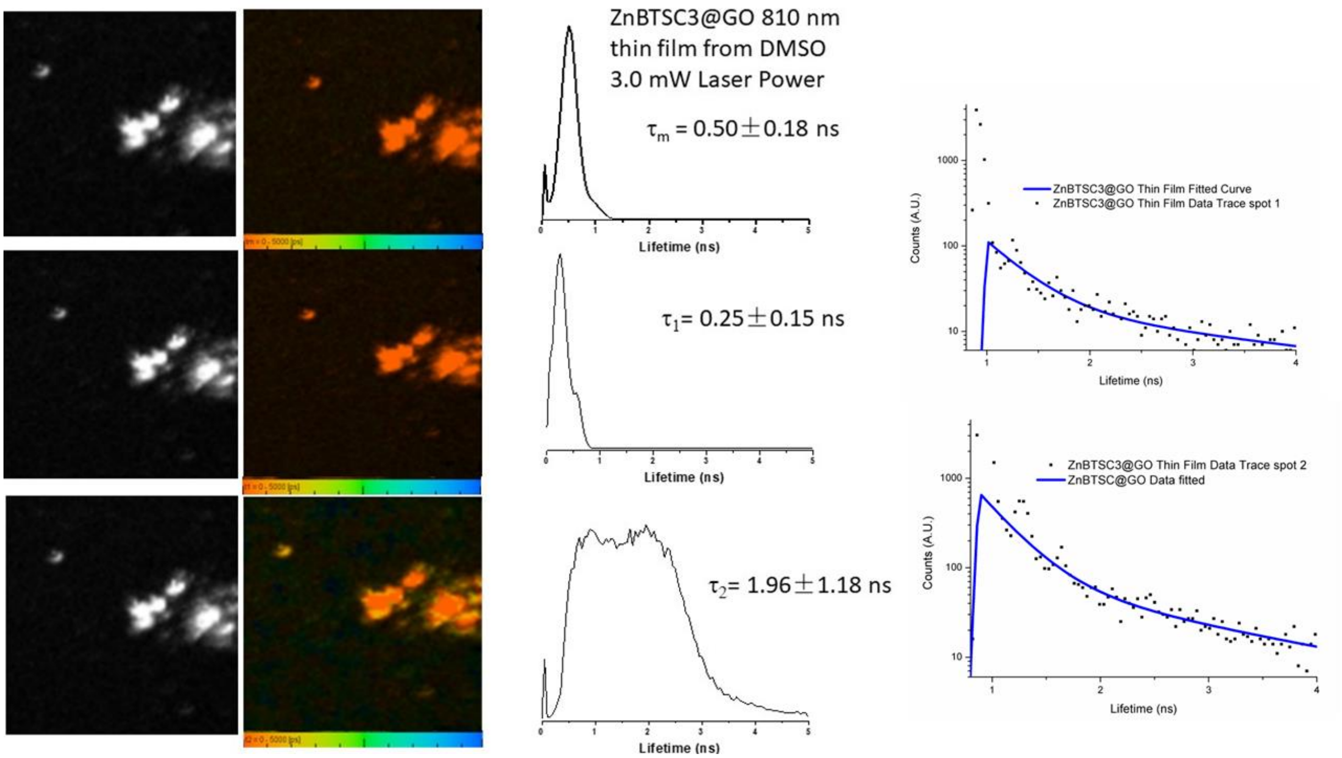

Figure S42. Two photon fluorescence spectroscopy investigations at 810 nm laser power 3 mW of ZnBTSC3@GO, 10 mM of ZnBTSC3 in 1 mg/mL of GO deposited on borosilicate glass from aqueous DMSO showing consistency in the dispersed phase and in thin film.

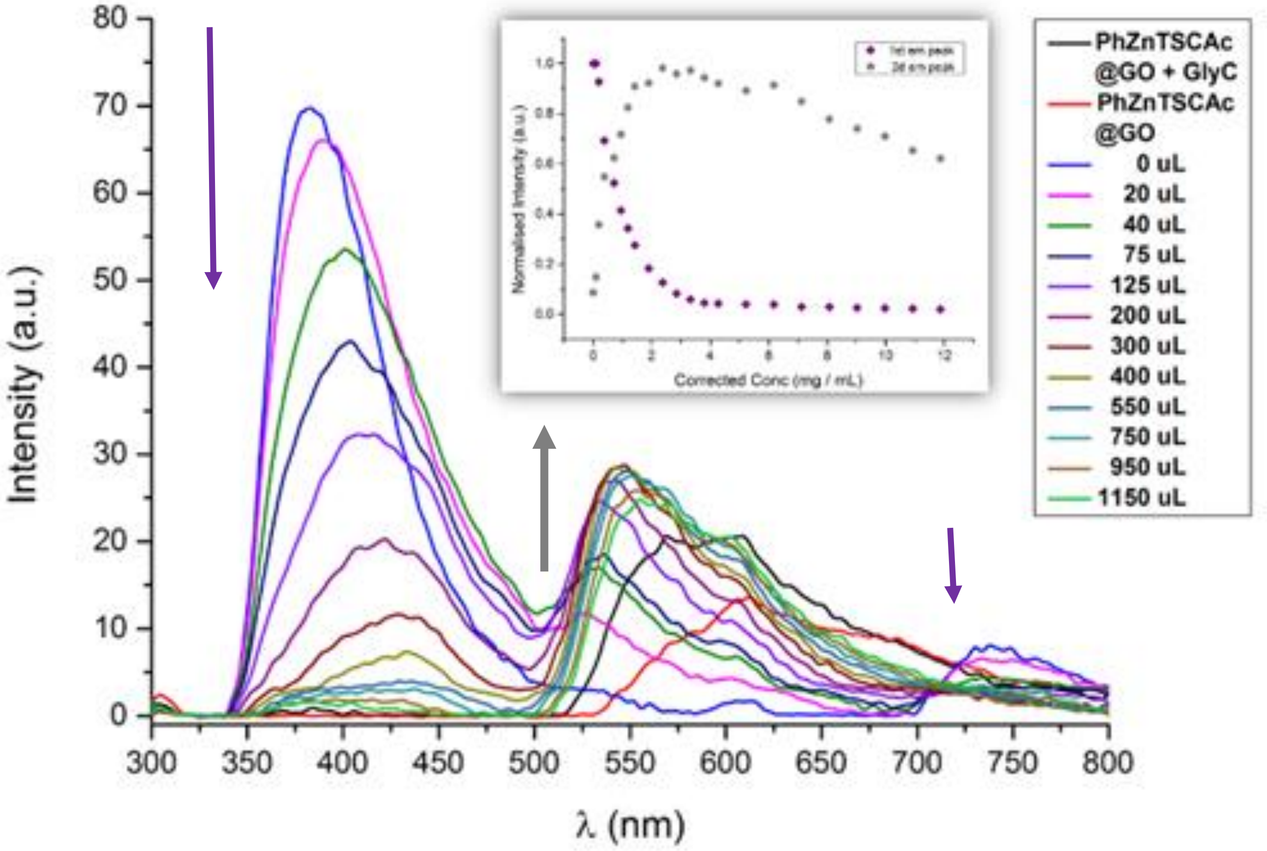

Figure S43. Fluorescence emission quenching experiment of a solution of GC (**4**) (0.3 mM) during titration experiment with **ZnBTSC3@GO** nanocomposite (mg/mL) in DMSO. The inset shows the normalised fluorescence emission intensity of the two maximum emission peaks at the relevant concentrations.

Note: The emission intensity that corresponds to compound 4 (GC) (lighter-blue line, max emission 400 nm, and also 730 nm) was reduced upon addition of the nanohybrid ZnBTSC3@GO in a DMSO suspension, and occurrence of a new fluorescence maximum (broad, ca 500-700 nm) was observed. The maximum fluorescence intensity of this emission band does not occur at the same wavelength as the free ZnBTSC3@GO (550 nm -700 nm) which suggests that complexation occurs, but also gives rise to a range of different supramolecularly aggregated species. As the titration proceeds, this new emission band is quenched, suggesting a rearrangement of the formed complex GC@ZnBTSC3@GO and significant self-aggregation. The inset graph depicts the normalised intensity of both emission bands at the relevant concentration of the complex ZnBTSC3@GO and points to a correlation between the increasing fluorescence intensity of the one emission band and the decreasing fluorescence intensity of the other.

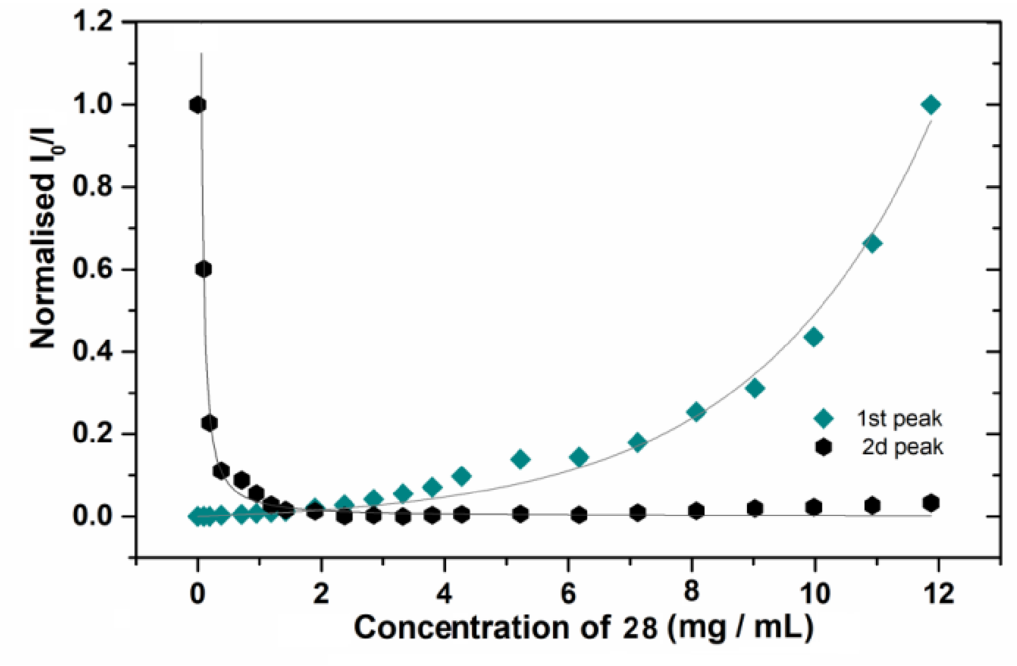

Figure S44. Normalised ratio of the initial emission intensity to the partial intensity for the two fluorescence maxima in Figure S43, at the relevant concentration for complex PhZnTSCAc @GO. The graph shows the normalised ratio between the initial fluorescence absorption of each peak to the partial intensity of it at the relevant complex PhZnTSCAc @GO concentration and it shows that the second emission band only increases its fluorescence intensity after the quenching of the first one. These results suggest that the formed hybrid has complex supramolecularly aggregated nature and a diverse variety of arrangements (Figure S44) are likely to form due to the non-covalent binding between complex PhZnTSCAc@GO and compound 4 (GC). These complex species in equilibrium may well be responsible for the rapid immobilization of such dyes in the nanohybrid.

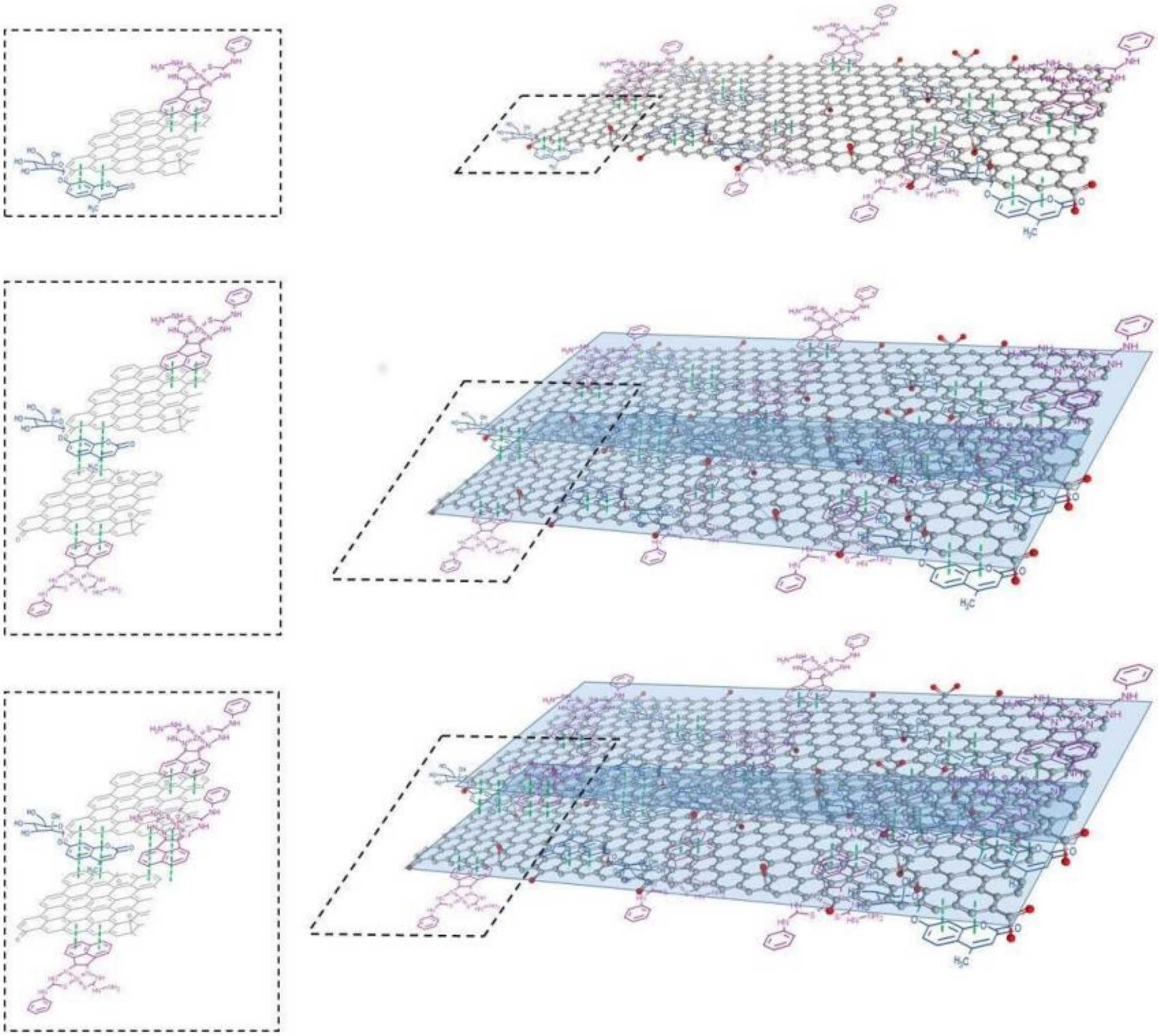

Figure S44. Schematic illustration of the postulated species of complex GC@ZnBTSC3@GO. In this figure the simplest version of those species is illustrated, thus n=1 or 2.

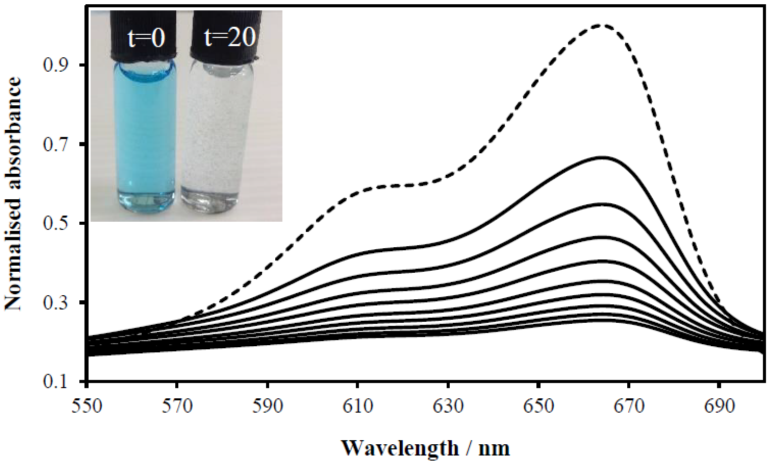

(a)

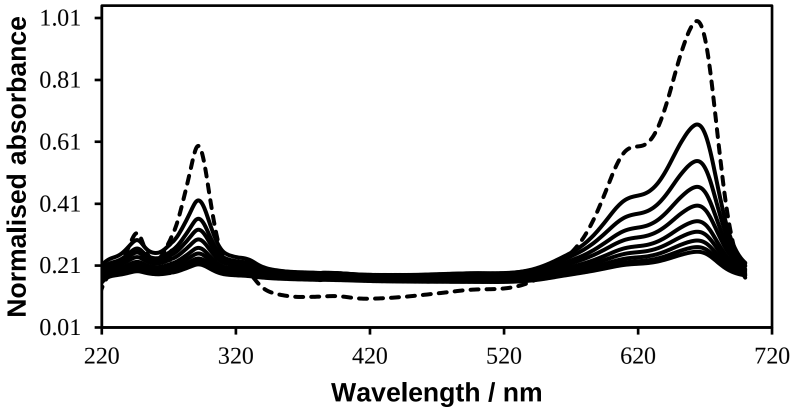

(b)

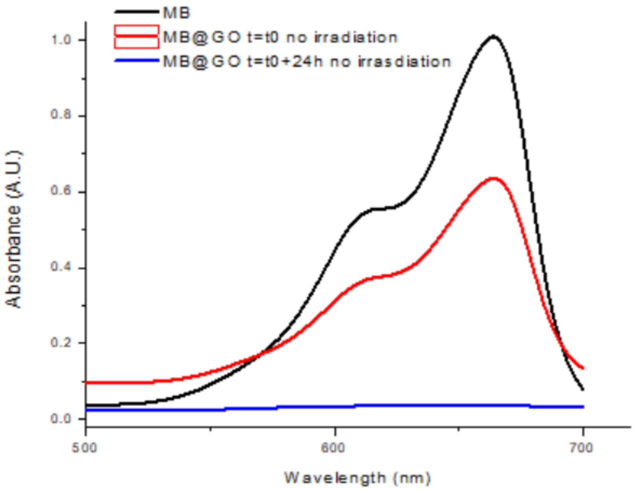

(c)

Figure S45. (a-b) UV/Vis spectroscopy of a methylene blue (MB) dye solution (2.68 mM, 200 mL, dotted line) and upon addition of GO (8 mg), whereby spectra were recorded every 2 minutes (successive solid lines, decreasing absorbance) over 20 minutes, without irradiation. 9c) **(c)** UV-Vis spectroscopy of MB solution (2.68 mM, 200 mL, dotted line) and of the MB solution (2.68 mM, 200 mL) upon addition of GO (8 mg) over time, without UV irradiation.

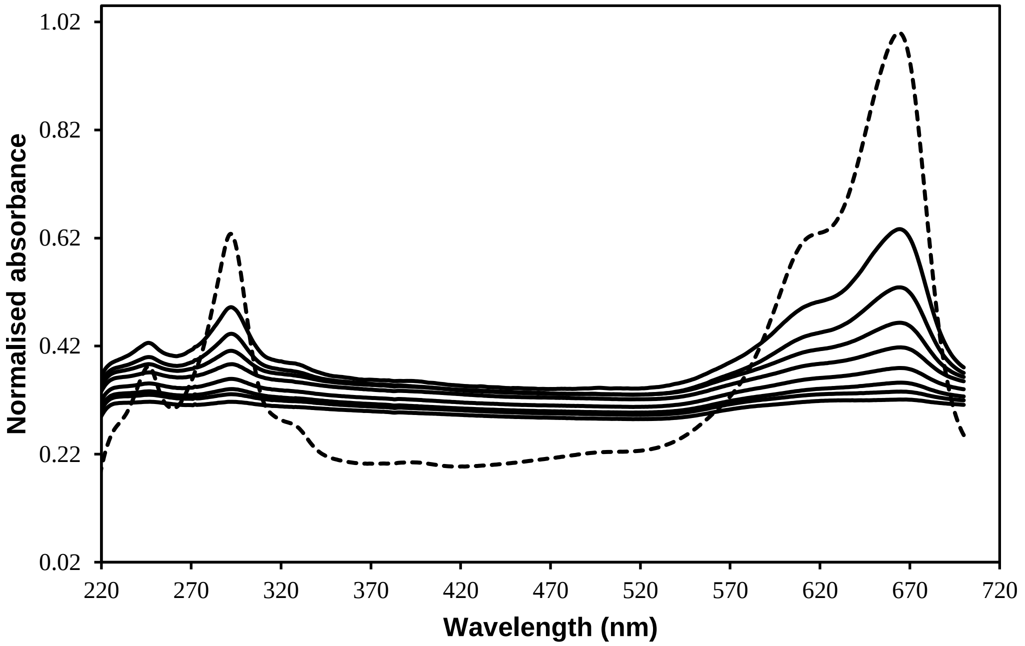

1.02
0.82
0.62
0.42
0.22
0.02
Normalised absorbance
220
270
320
370
420
470
520
570
620
670
720
Wavelength (nm)

(a)

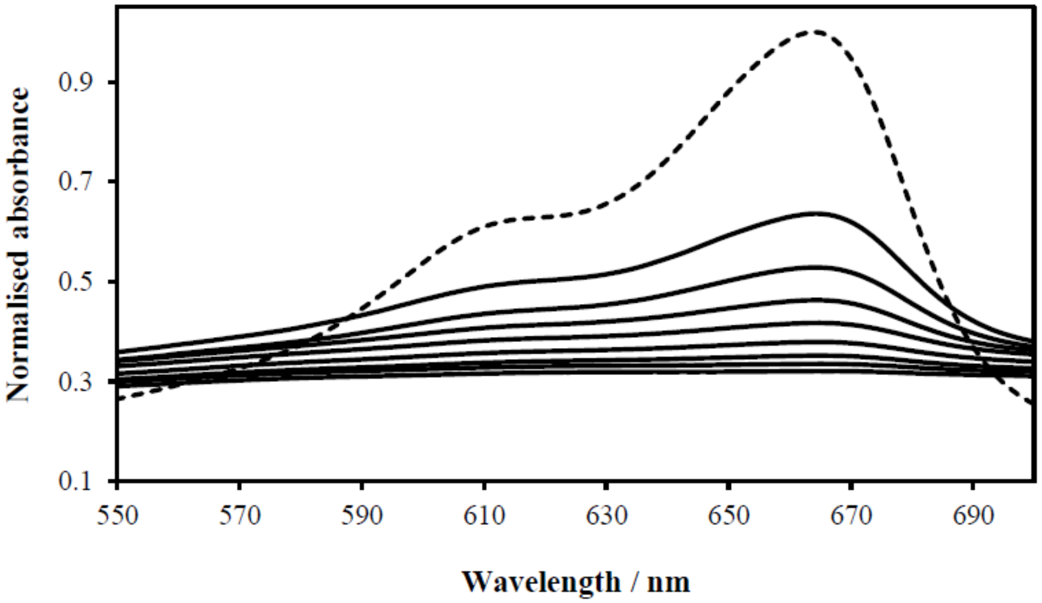

0.9
0.7
0.5
0.3
0.1
Normalised absorbance
550
570
590
610
630
650
670
690
Wavelength / nm

(b)

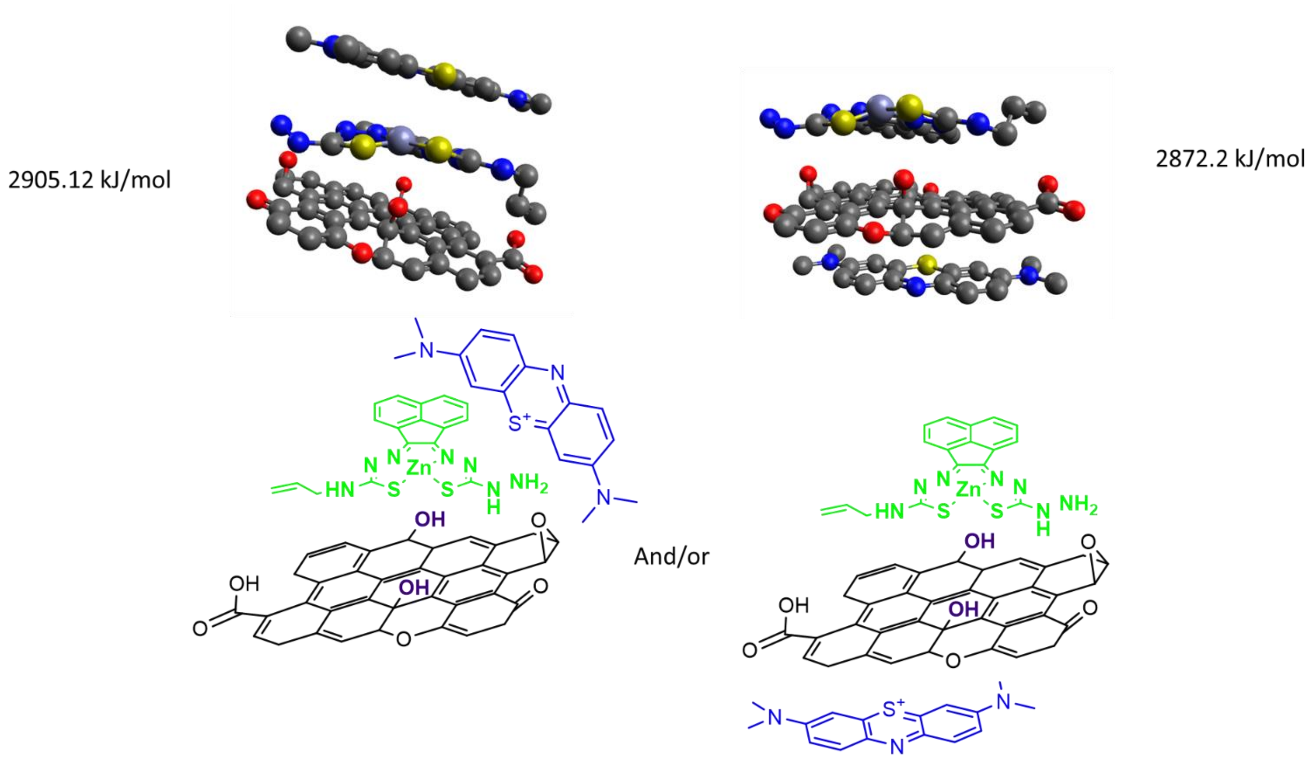

(c)

Figure S46. (a-b) UV/Vis spectroscopy of MB solution (2.68 mM, 200 mL, dotted line) and of the MB solution (2.68 mM, 200 mL) upon addition of a hybrid formed by compound **ZnBTSC2** (8 mg) anchored onto GO (8 mg). Spectra were recorded every 2 minutes without UV irradiation over 20 min (corresponding data is represented by successive solid lines, decreasing absorbance). **(c)** proposed geometries for the MB association with ZnBTSC2@GO pre-formed hybrid, from MM+ optimization.

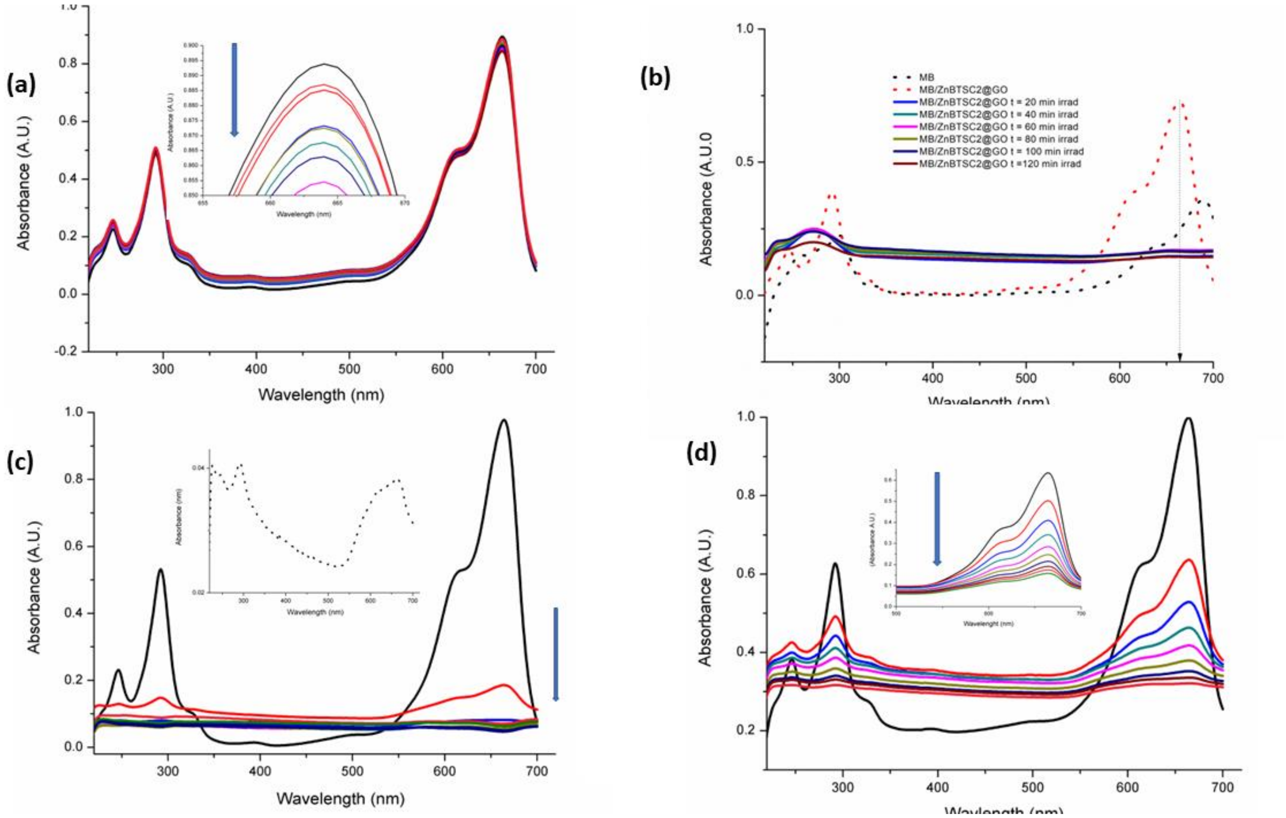
(a)
Absorbance (A.U.)
Wavelength (nm)
(b)
MB
MB/ZnBTSC2@GO
MB/ZnBTSC2@GO t = 20 min irrad
MB/ZnBTSC2@GO t = 40 min irrad
MB/ZnBTSC2@GO t = 60 min irrad
MB/ZnBTSC2@GO t = 80 min irrad
MB/ZnBTSC2@GO t = 100 min irrad
MB/ZnBTSC2@GO t =120 min irrad
Absorbance (A.U.0
(c)
Absorbance (A.U.)
Wavelength (nm)
(d)
Absorbance (A.U.)

(e)

MB on GO - No irradiation

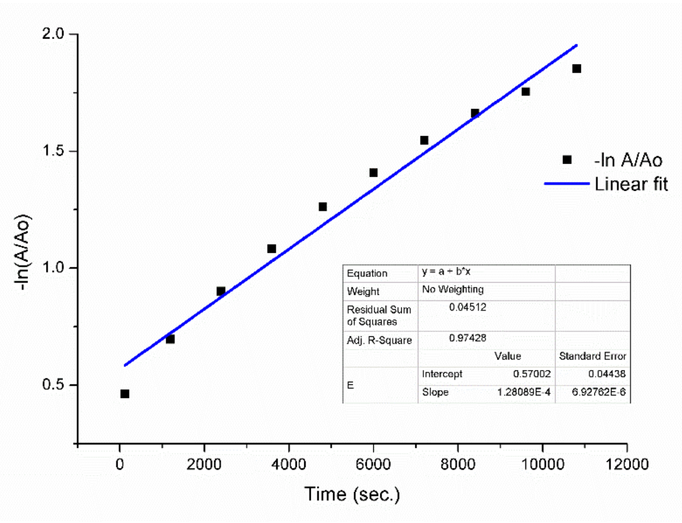

(f)

MB on ZnBTSC2@GO - No irradiation

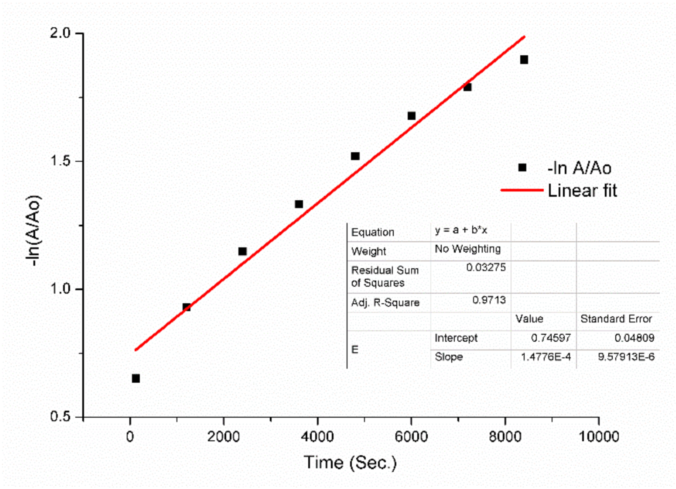

(g)

MB with ZnBTSC2 in DMSO:H2O 1:1

3h irradiation (violet light)

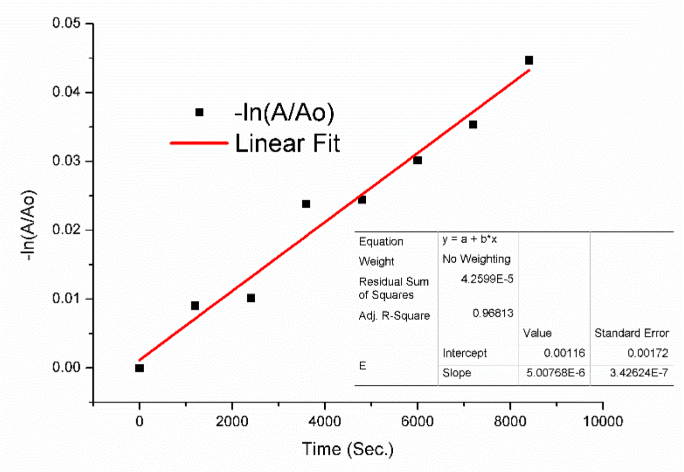

Figure S48. (a-d) Overview of the UV-Vis data collected over 2-3 hours under a series of conditions and corresponding estimation of pseudo-first order kinetics behavior: **(a)** Methylene blue irradiated in the presence of ZnBTSC2 over 2 h with violet light (in DMSO:H_2O 1:1); **(b)** Methylene blue irradiated in the presence of ZnBTSC2@GO over 2 h. Dotted lines represent correspondingly the absorption of the original free MB solution, and of the MB onto ZnBTSC2@GO prior to irradiation. **(c)** Absorption of MB on GO without irradiation monitored over 3 h, in DMSO:H_2O; Inset: the dispersion of MB onto GO after 24 h, expansion of the normalized absorption axis; **(d)** Absorption of MB on ZnBTSC2@GO without irradiation monitored over 2 h, with detailed view of first 20 min. Black line represents free B under the same conditions. **(e-g)** Pseudo-first order behaviour of MB under the various conditions tested. Whilst the behaviour of MB with ZnBTSC2@GO in DMSO:H_2O 1:1 under 3h irradiation (violet light) also displayed a similar trend (with ca 8 x 10^{-6} rate), the R^2 was ca. 0.7.

.

Selected solid state characterisation data

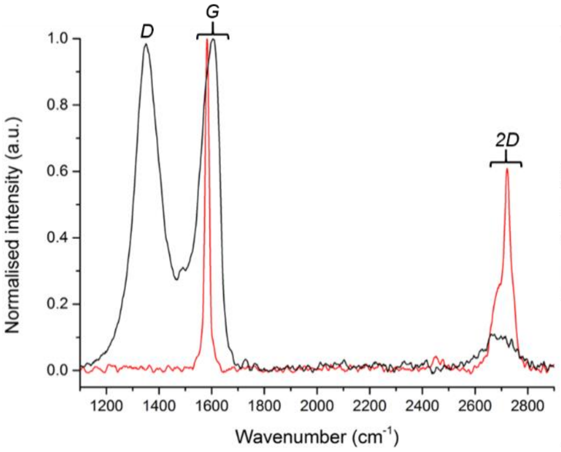

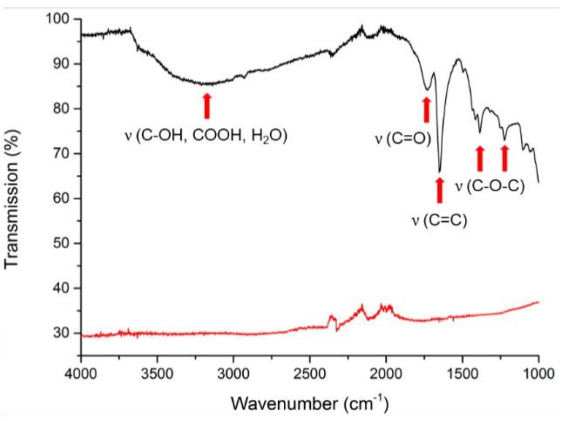

(a) **(b)**

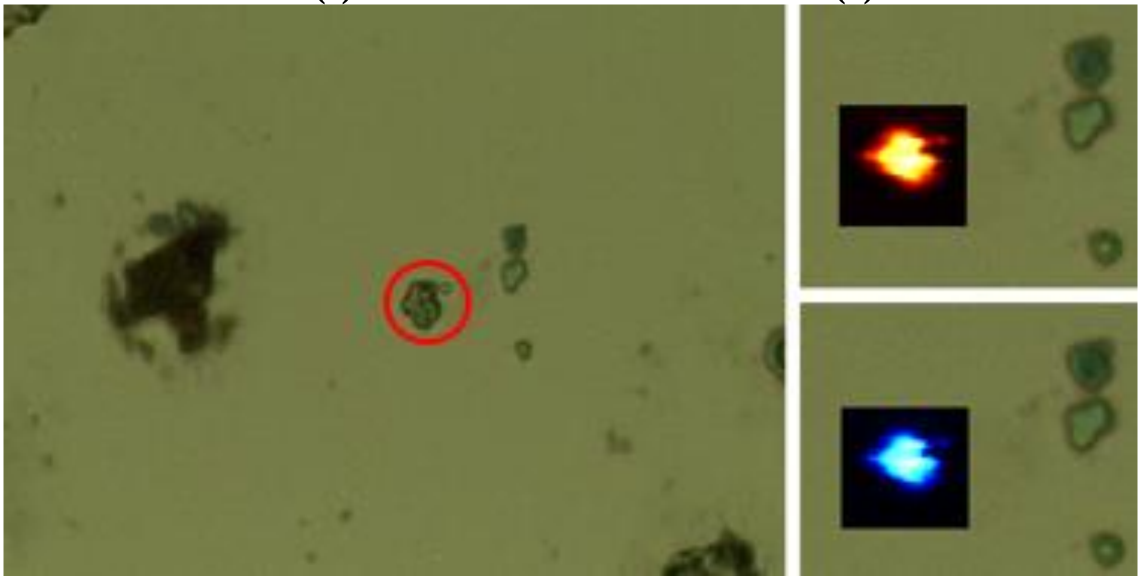

(c)

Figure S49: Raman (**a**) and solid state IR (b) spectroscopies of the as-synthesised GO nanoflakes (black lines) and pristine graphite starting material (red lines). (**c**) Raman map of a selected GO nanoflake (the 2D-mapping, left); the red-colour scale corresponds to the energy of the *G band* of GO (1601 cm^{-1}) and the colour scale represented in blue corresponds to the energy of the energy of *D band* (1367 cm^{-1}). In the Raman spectroscopy 2D-mapping, each colour scale selected was represented in 4 different colours, from the darkest to the lightest shade, corresponding to diverse depth of the flake and, for each selected depth, the corresponding Raman spectra are given in Figures S50 and S51. As expected, in the Raman spectrum of graphite, the *G band* at 1582.10 cm^{-1} is due to the symmetry of hexagonal lattice; the $2D_1$ and $2D_2$ *bands* at 2688.28 cm^{-1} and 2722.40 cm^{-1}, respectively, due to a double resonance enhancement by 2-phonons. In the GO Raman spectrum, the *D* peak is located at 1357.03 cm^{-1} due to the out-of-plane vibrations attributed to the presence of structural defects induced by the disruption of sp^2 bonds of the carbon owing to the existence of oxidised functional groups (carboxyl, alcoholic and epoxy groups); the *G band* at 1595.05 cm^{-1} is due to the in-plane vibrations of the sp^2 carbon atoms; the *2D band* at 2688.98 cm^{-1} is due to the out-of-plane vibrations attributed to the presence of structural defects.

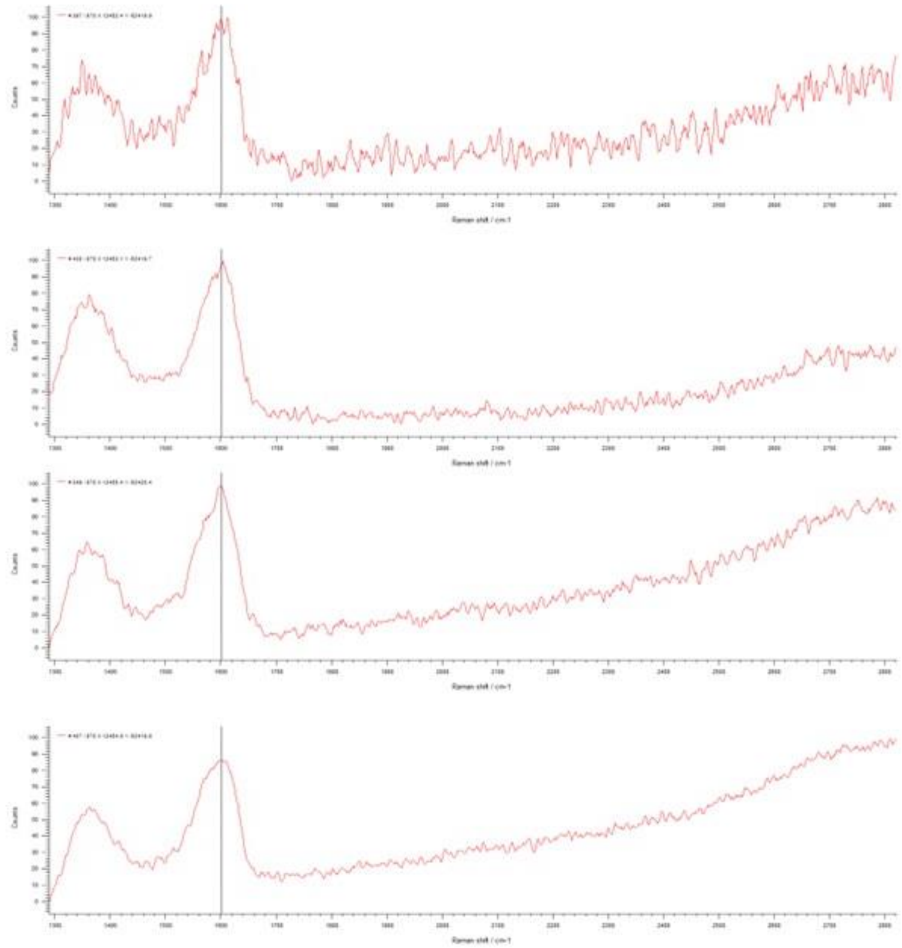

Figure S50: Raman spectra of the flake, radiated at 1601 cm^{-1}. On the top, Raman spectrum of the deepest point (corresponding to dark red in the red-colour scale) of the selected flake and, on the bottom, Raman spectrum of the highest part (corresponding to white) of the flake.

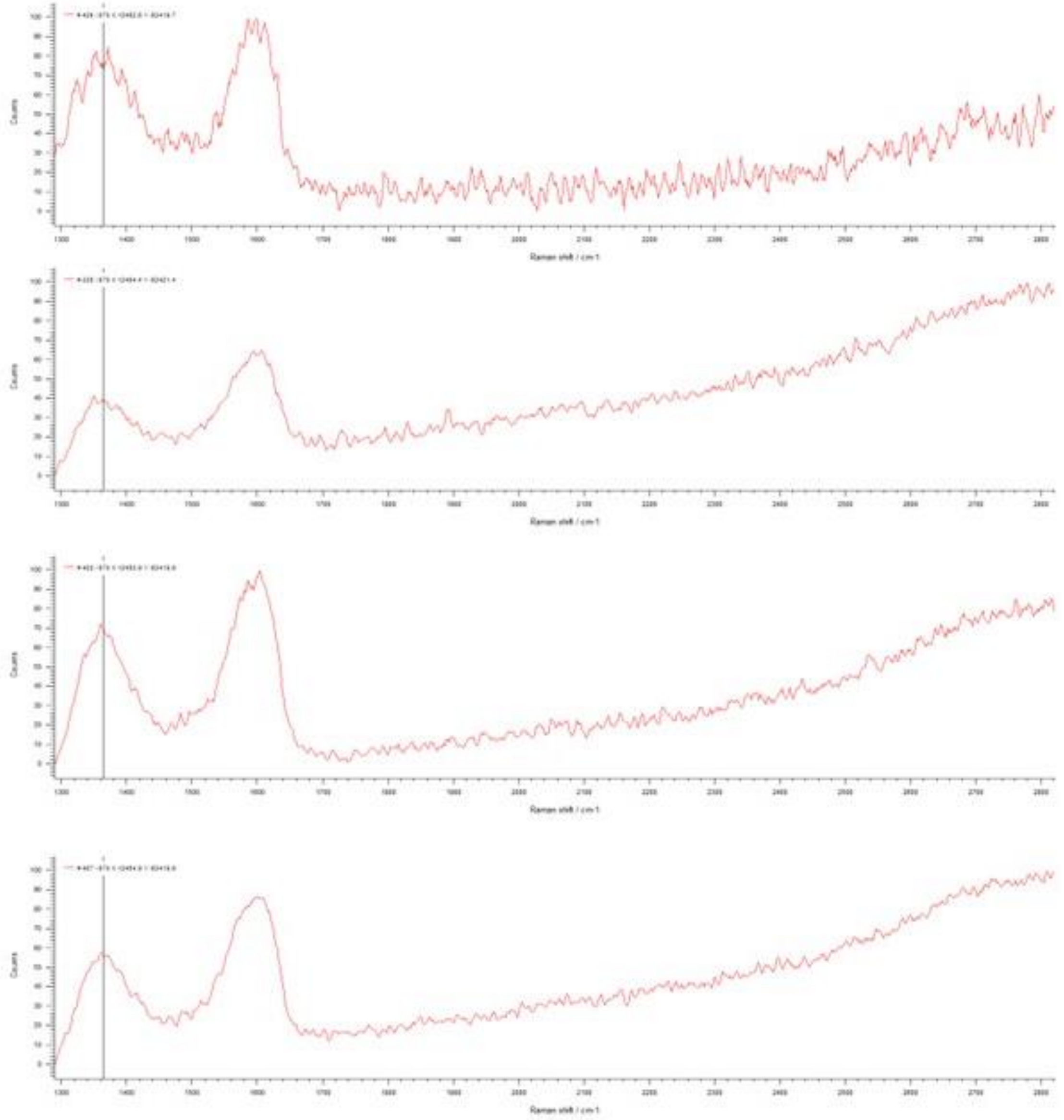

Figure S51: Raman spectra of the flake, radiated at 1357 cm^{-1}. On the top, Raman spectrum of the deepest point (corresponding to dark blue in the blue-colour scale) of the selected flake and, on the bottom, Raman spectrum of the highest part (corresponding to white) of the flake.

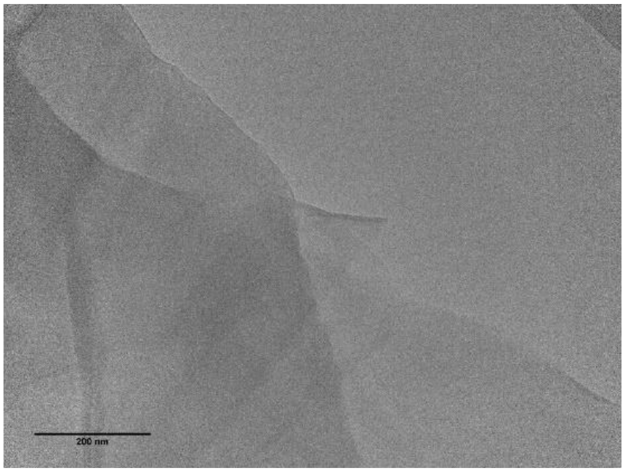

(a)

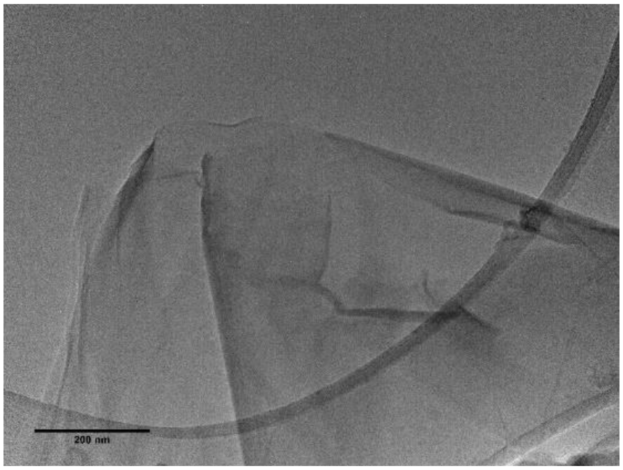

(b)

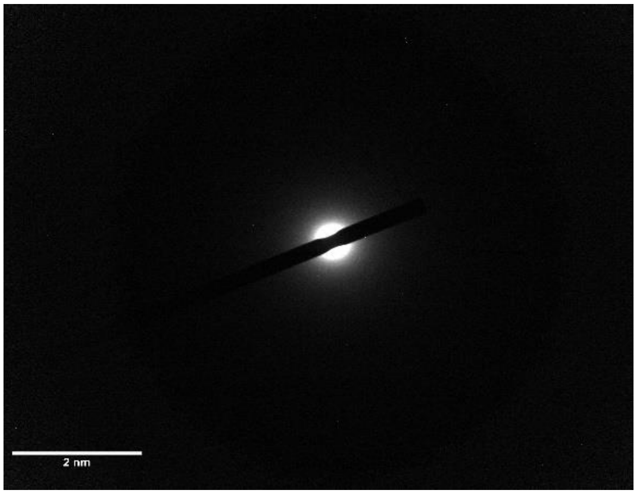

(c)

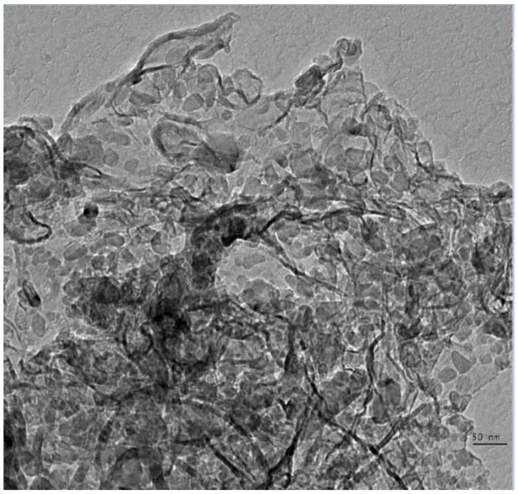

(d)

Element	Weight %	Atomic %
C	14.13	20.4
O	56.22	60.94
Al	14.19	9.12
Si	15.46	9.54
Total	100	100

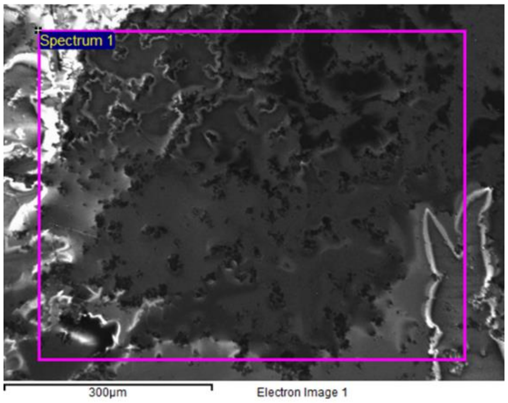

Figure S52. TEM (a-b), electron diffraction (SAED); (c), HRTEM (d) and SEM with EDX (e) and of the as-prepared exfoliated GO nanoflakes used hereby. Samples were deposited onto lacey copper grids from EtOH.

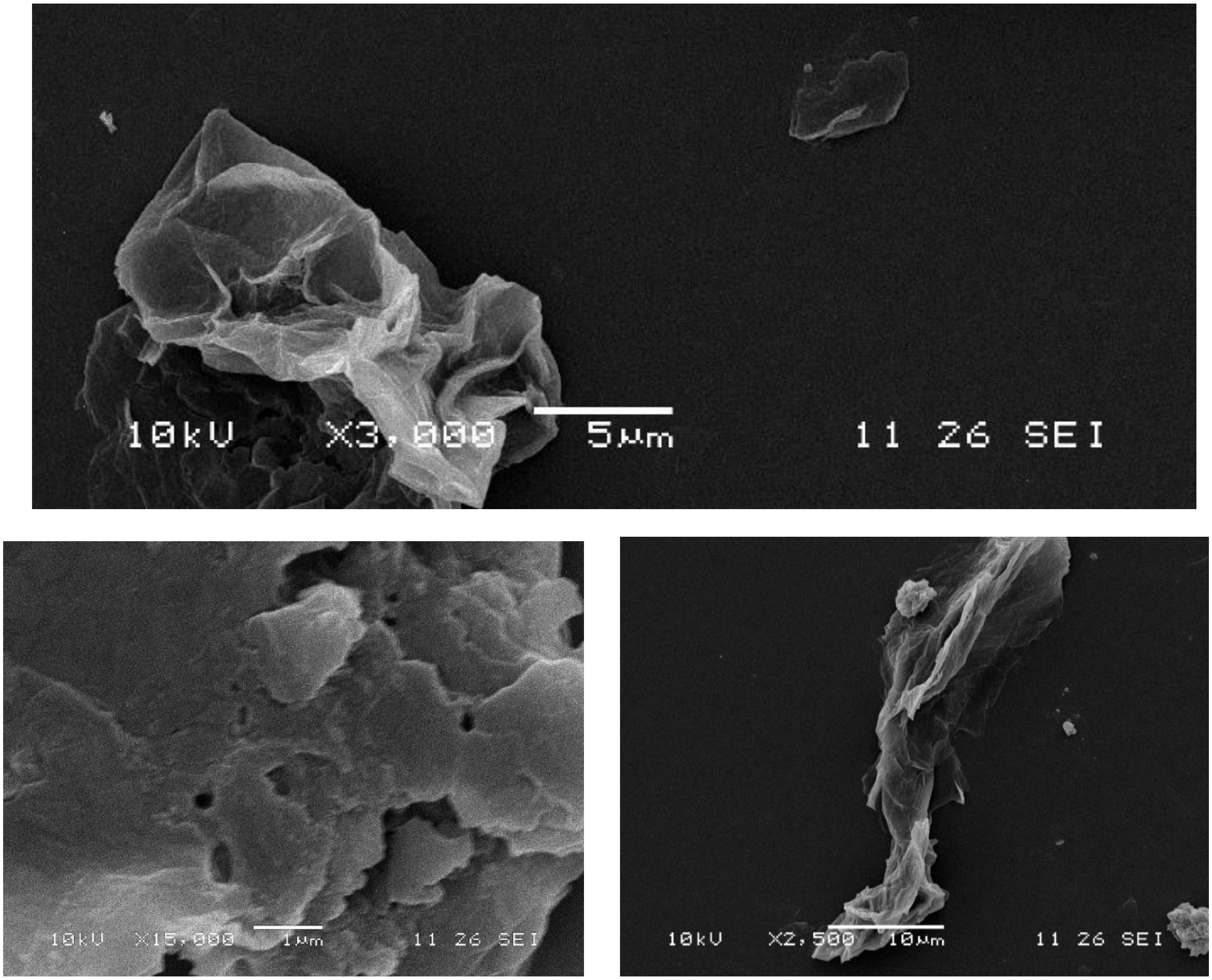

Figure S53. FESEM of the as-prepared GO at different magnifications. Graphene oxide 1 mg/ 1 mL dispersion in EtOH were imaged on HOPG after chromium coating.

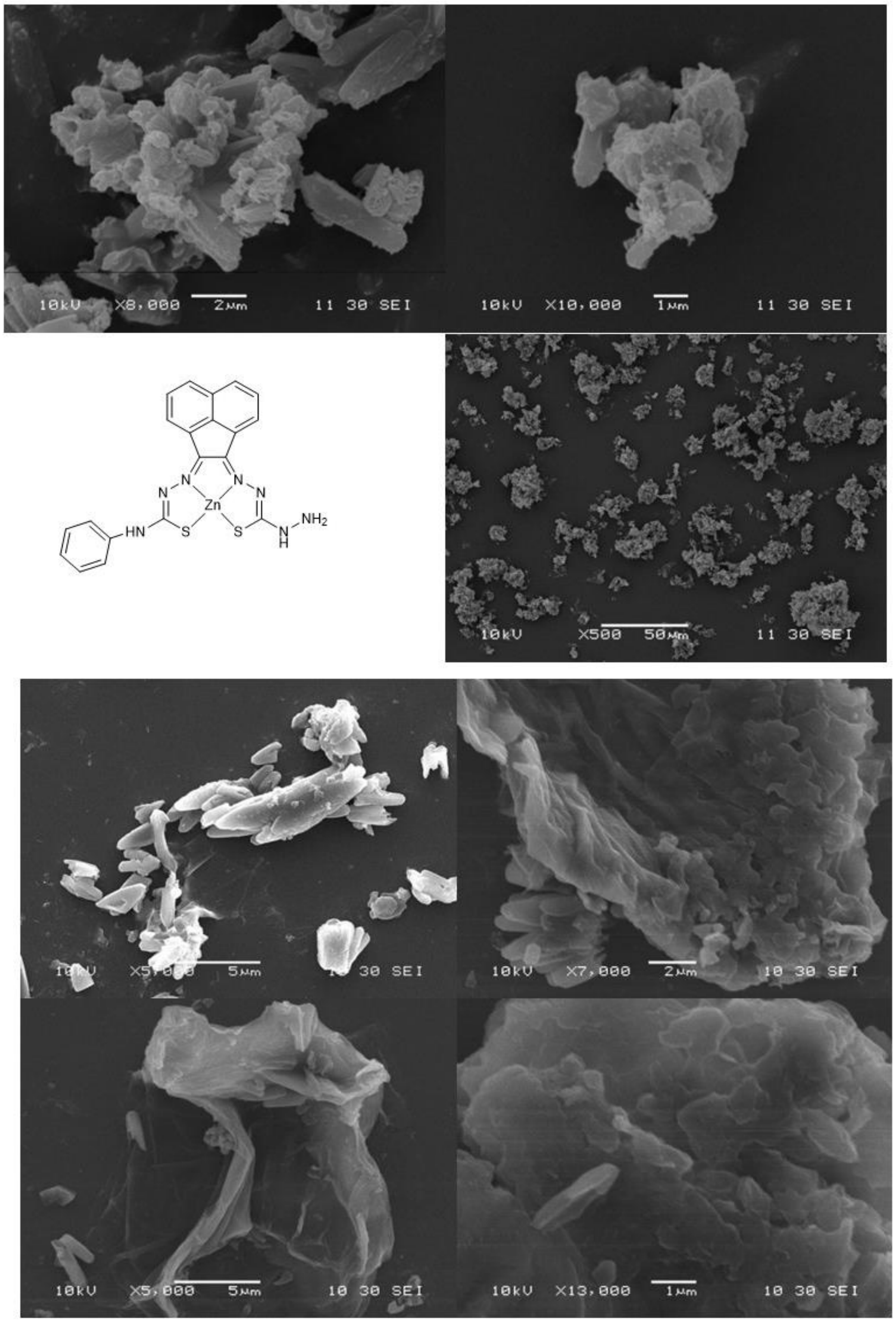

Figure S54. FESEM of microcystaline ZnBTSC3, imaged on HOPG and with gold coating.

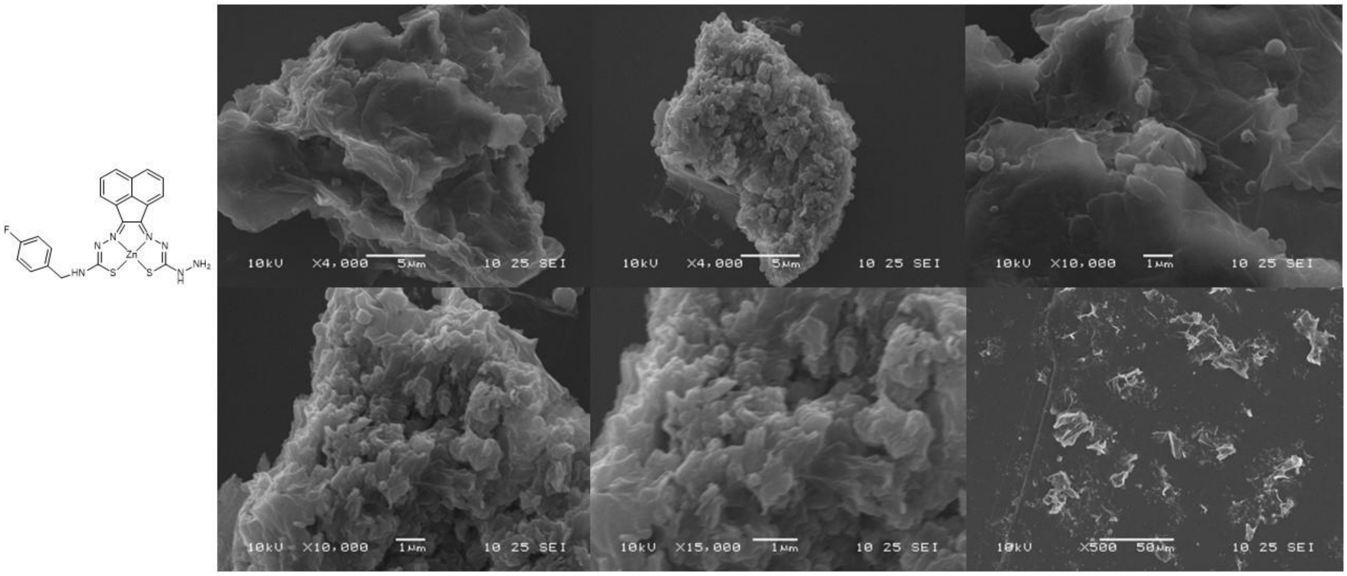

Figure S55. FESEM of microcystaline ZnBTSC4, imaged on HOPG with gold coating.

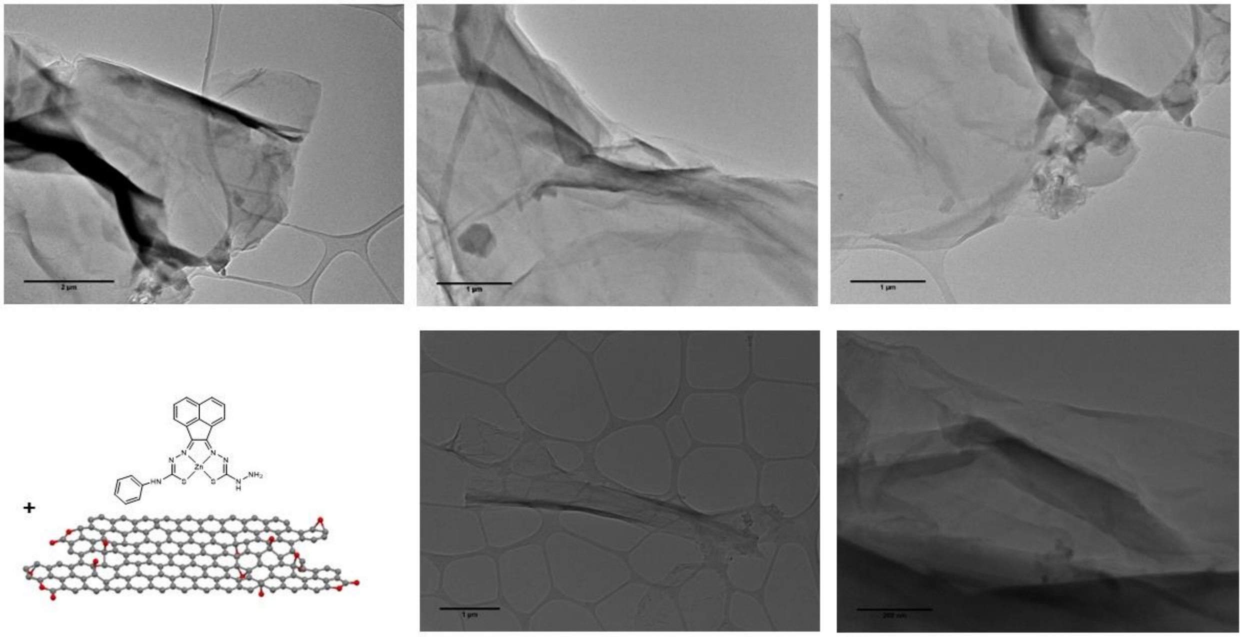

ZnBTSC3

Element	Weight %	Atomic %
C K	73.53	87.80
O K	5.02	4.50
Si K	0.43	0.22
S K	12.59	5.63
Zn L	8.43	1.85

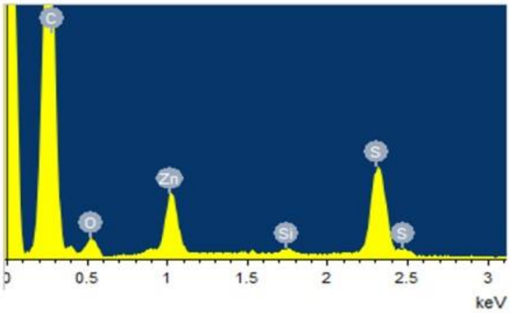

ZnBTSC3@GO

Element	Weight %	Atomic %
O K	50.97	66.34
Mg K	0.51	0.44
Al K	16.37	12.63
Si K	20.30	15.05
S K	0.29	0.19
C K	4.45	2.37
Fe L	6.49	2.42
Zn L	0.63	0.56

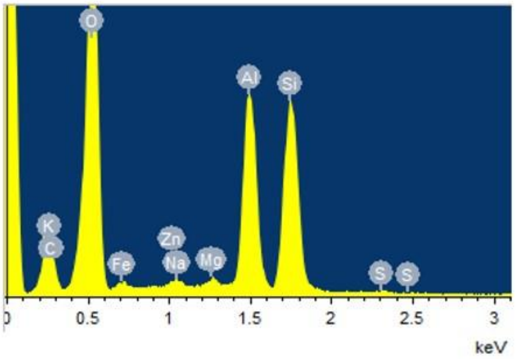

Figure S56. TEM characterization of ZnBTSC3@GO hybrid at different magnifications and corresponding EDX.

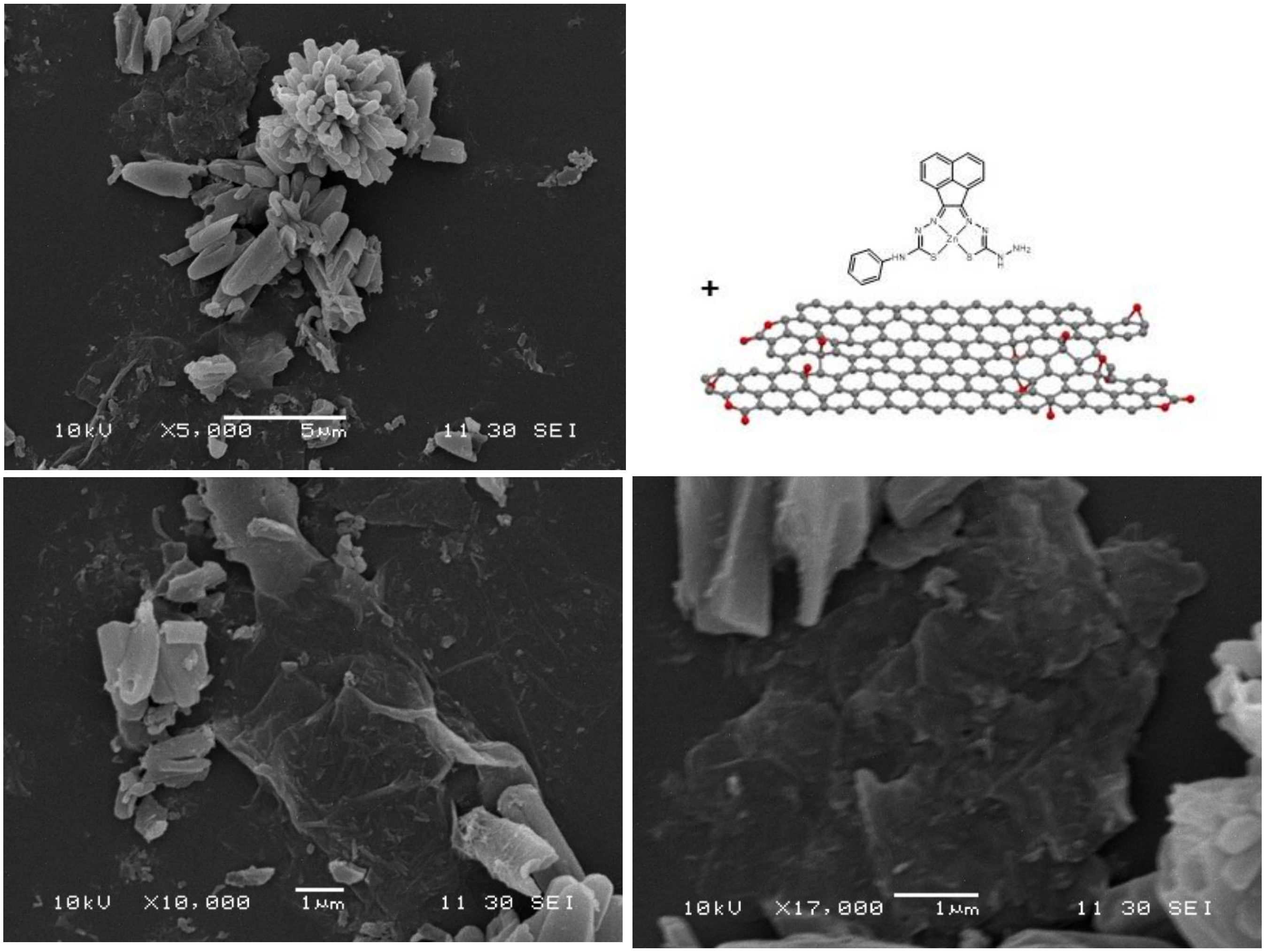

Figure S57. FESEM characterisation of ZnBTSC3@GO hybrid at different magnifications. Images were recorded after the isolated hybrid was deposited onto carbon stubs from a 9:1 EtOH:DMSO 0.25 mg/ml dispersion, vacuum dried and chromium coated.

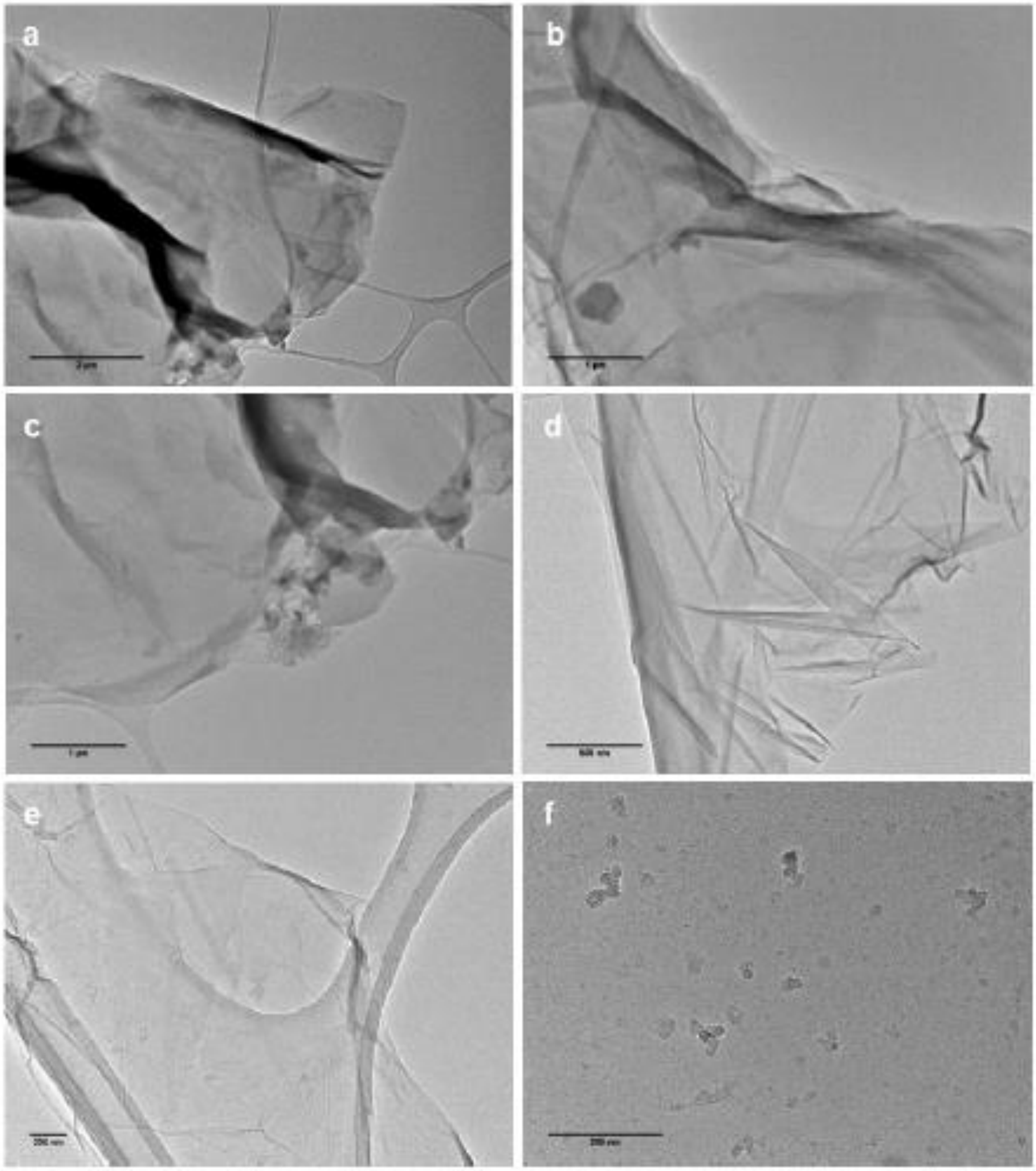

Figure S58. A comparison of the TEMs for ZnBTSC3@GO and ZnBTSC4@GO. TEMs (a-c) correspond to the as-prepared nanohybrids of ZnBTSC3 anchored onto exfoliated GO nanoflakes which were used hereby. Samples were deposited onto lacey copper grids from EtOH:DMSO (9:1 v/v ratio, 0.25 mg/ml). Images dislplay GO nanoflakes decorated with aggreagated ZnBTSC3 on the Surface. TEMs (d-f) correspond to the as-prepared nanohybrid of ZnBTSC4 anchored onto exfoliated GO nanoflakes, used hereby. Samples were deposited onto lacey copper grids from EtOH:DMSO (9:1 v/v ratio, 0.25 mg/ml). Images display GO nanoflakes decorated with surface-aggregated ZnBTSC4.

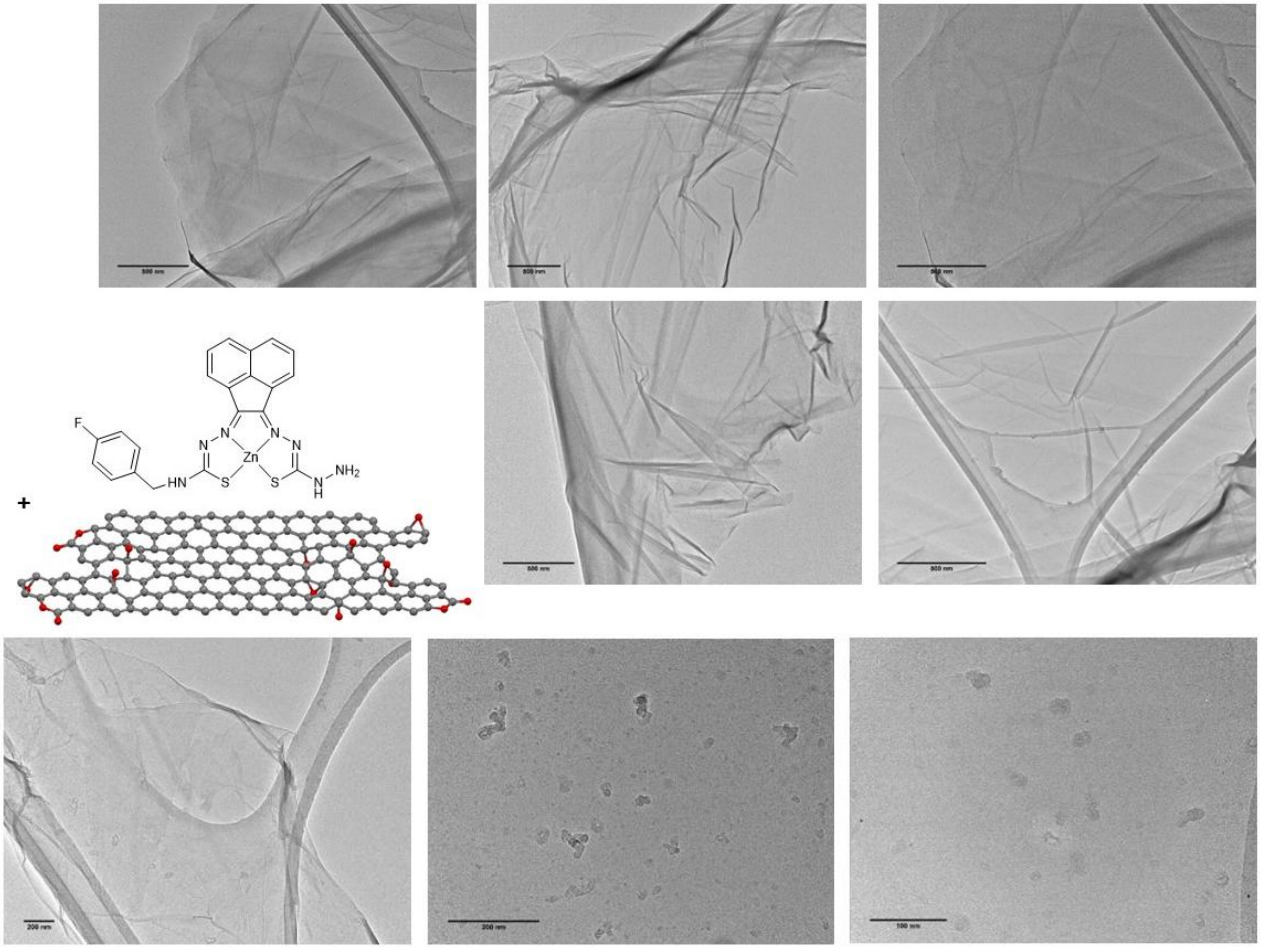

Figure S59. TEM characterization of ZnBTSC4@GO hybrid at different magnifications.

X-ray Crystallography Details

The selected crystal was mounted onto a goniometer head and cooled to 150K with an Oxford Cryosystem. Intensity data for (CG) (**4**) and (AcGC) (**5**) were collected on a SuperNova, Dual, Cu at zero, EosS2 using a Cu microfocus source (λ = 1.54184) Å. Unit cell determination, data collection, data reduction and a symmetry-related (multi-scan) absorption correction were performed using the CrysAlisPro software [1] The structure of AcGC (CCDC: 2301154, internal data code s15sip4) was solved with SUPERFLIP and that of GC (CCDC: 2301155, internal data code s17sip7) was solved with SHELXT. The structures were then refined by a full-matrix least-squares procedure based on F^2 (Shelxl-2019/2).[2] All non-hydrogen atoms were refined anisotropically. Hydrogen atoms were placed onto calculated positions and refined using a riding model. Additional programmes used for analyzing data and their graphical manipulation included SHELXle [3] and Mercury[4].The crystal structures data via the joint CCDC/FIZ Karlsruhe deposition service, deposition numbers are 2301154 (AcGC) and 2301155 (GC).

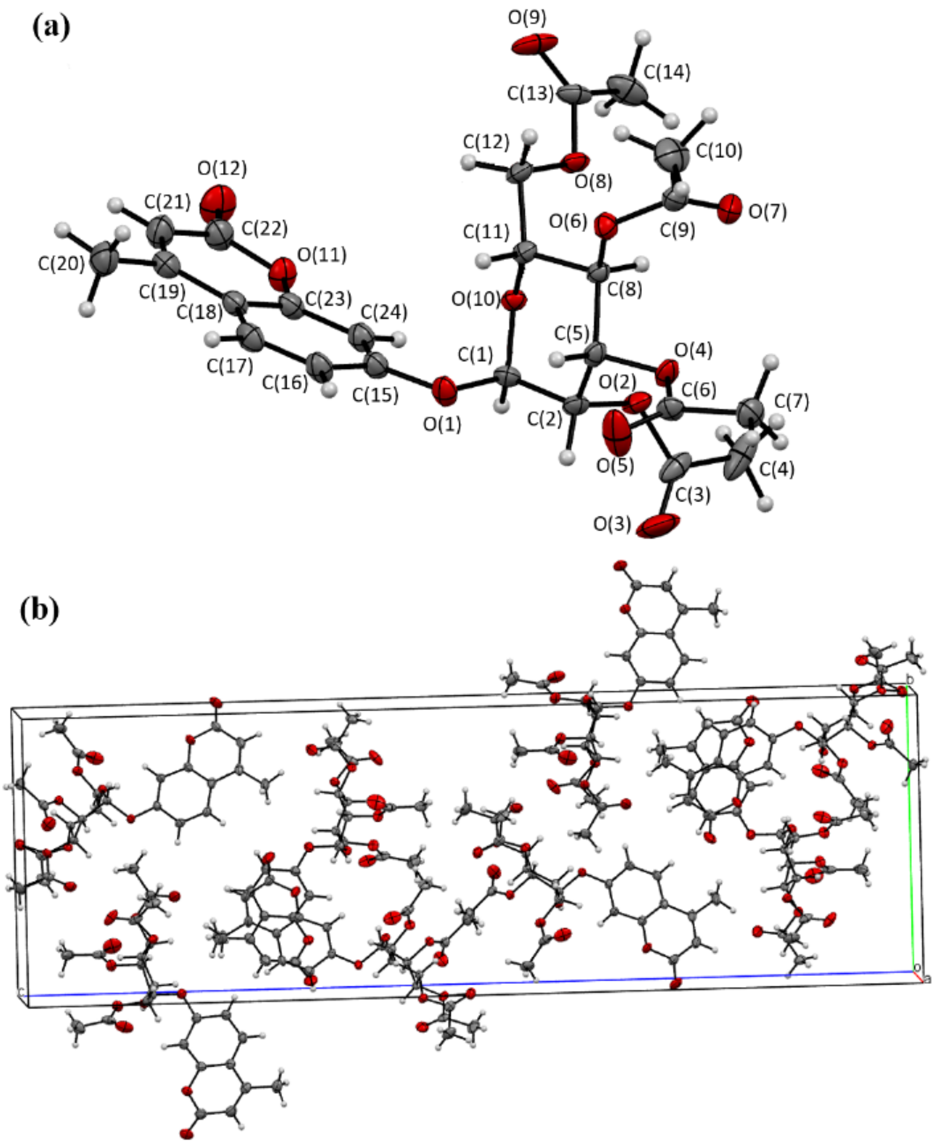

Figure S60. (a) Molecular structure of 4-methylumbelliferyl-7-yl-2,3,4,6-tetra-O-acetyl- α-D-mannopyranose (AcGC) (**5**). Only one of the molecules of the asymmetric unit is shown for clarity. **(b)** 3D X-ray packing showing the unit cell. Thermal ellipsoids at 50% probability.

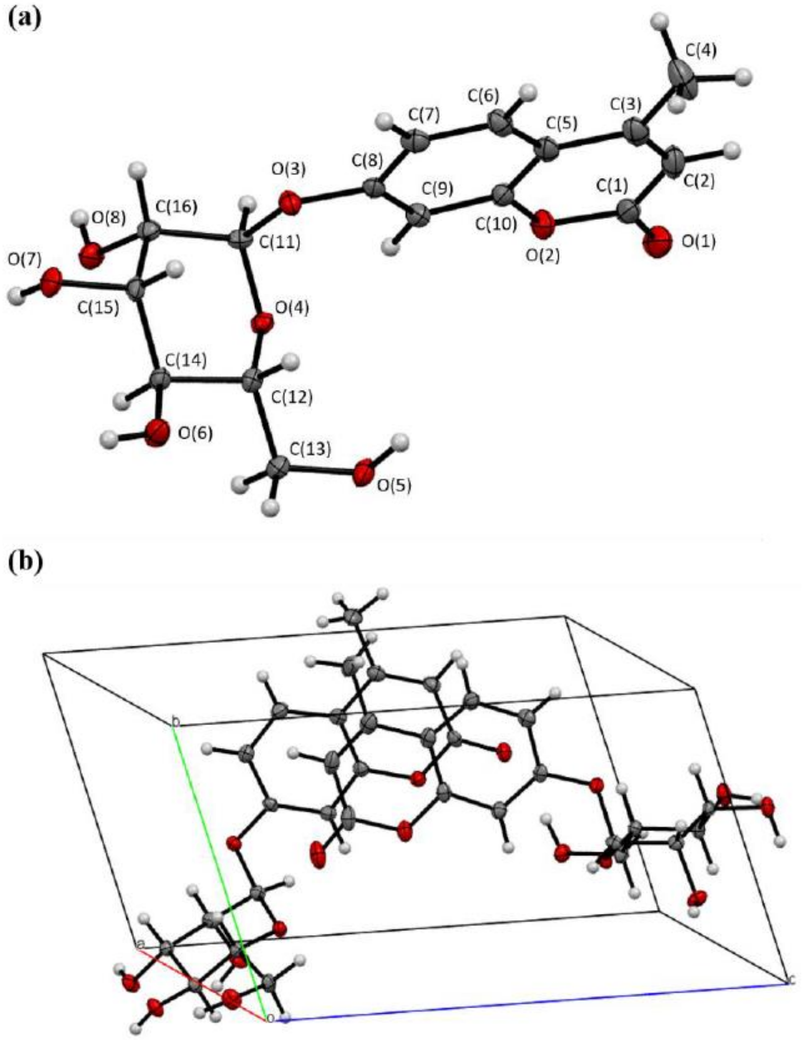

Figure S61. **(a)** Molecular structure of 4-methylumbelliferyl-7-yl-α-D-mannopyranose, **GC (4).** Only one of the molecules of the asymmetric unit is shown for clarity**. (b)** 3D X-ray packing showing the unit cell. Thermal ellipsoids at 50% probability.

Table S1. Crystal data and structure refinement for (AcGC) (**5**).

Identification code	(AcGC) (**5**)	
Empirical formula	C24 H26 O12	
Formula weight	506.45	
Temperature	150(2) K	
Wavelength	1.54184 Å	
Crystal system	Orthorhombic	
Space group	$P2_12_12_1$	
Unit cell dimensions	a = 8.45570(10) Å	a= 90°.
	b = 13.6108(2) Å	b= 90°.
	c = 42.3604(6) Å	g = 90°.
Volume	4875.21(11) $Å^3$	
Z	8	
Density (calculated)	1.380 Mg/m^3	
Absorption coefficient	0.955 mm^{-1}	
F(000)	2128	
Crystal size	0.320 x 0.150 x 0.100 mm^3	
Theta range for data collection	4.512 to 71.987°.	
Index ranges	-6<=h<=10, -16<=k<=16, -45<=l<=52	
Reflections collected	42452	
Independent reflections	9541 [R(int) = 0.0602]	
Completeness to theta = 67.684°	99.9 %	
Absorption correction	Semi-empirical from equivalents	
Max. and min. transmission	1.00000 and 0.78152	
Refinement method	Full-matrix least-squares on F^2	
Data / restraints / parameters	9541 / 0 / 660	
Goodness-of-fit on F^2	1.026	
Final R indices [I>2sigma(I)]	R1 = 0.0439, wR2 = 0.1129	
R indices (all data)	R1 = 0.0473, wR2 = 0.1163	
Absolute structure parameter	0.09(7)	
Extinction coefficient	n/a	
Largest diff. peak and hole	0.665 and -0.282 $e.Å^{-3}$	

Table S2. Atomic coordinates (x10^4) and equivalent isotropic displacement parameters (Å^{2}x 10^3) for (AcGC) (**5**). U(eq) is defined as one third of the trace of the orthogonalized U^{ij} tensor.

	x	y	z	U(eq)
C(1)	-240(3)	4882(2)	1435(1)	22(1)
C(2)	-709(3)	3841(2)	1338(1)	22(1)
C(3)	-2252(4)	3523(2)	883(1)	32(1)
O(3)	-3352(3)	3285(2)	1044(1)	54(1)
C(5)	583(3)	3104(2)	1419(1)	21(1)
C(6)	-235(4)	1426(2)	1448(1)	24(1)
C(7)	-256(4)	500(2)	1260(1)	32(1)
C(8)	2185(3)	3453(2)	1303(1)	19(1)
C(9)	3994(4)	2108(2)	1230(1)	31(1)
C(10)	5142(5)	1487(3)	1408(1)	45(1)
C(11)	2498(3)	4471(2)	1437(1)	19(1)
C(12)	4057(3)	4922(2)	1342(1)	24(1)
C(13)	5333(4)	5504(2)	889(1)	28(1)
C(14)	5176(5)	5719(3)	545(1)	44(1)
C(15)	119(4)	5726(2)	1927(1)	25(1)
C(16)	286(4)	5637(2)	2254(1)	28(1)
C(17)	879(4)	6413(2)	2425(1)	29(1)
C(18)	1340(3)	7296(2)	2278(1)	24(1)
C(19)	2102(4)	8110(2)	2434(1)	28(1)
C(20)	2439(5)	8064(3)	2782(1)	39(1)
C(21)	2543(4)	8891(3)	2261(1)	35(1)
C(22)	2263(4)	8966(3)	1924(1)	33(1)
C(23)	1075(4)	7367(2)	1953(1)	25(1)
C(24)	451(4)	6604(2)	1774(1)	26(1)
C(25)	6313(3)	8252(2)	1025(1)	18(1)
C(26)	6808(3)	8855(2)	739(1)	17(1)
C(27)	8259(3)	8267(2)	297(1)	24(1)
C(28)	8273(4)	7508(3)	44(1)	32(1)
C(29)	5565(3)	9619(2)	656(1)	18(1)
C(30)	6892(3)	10814(2)	348(1)	24(1)
C(31)	7105(5)	11172(3)	16(1)	44(1)
C(32)	3922(3)	9181(2)	641(1)	16(1)
C(33)	2324(3)	10247(2)	325(1)	24(1)
C(34)	1304(4)	11144(3)	353(1)	38(1)

C(35)	3599(3)	8659(2)	952(1)	17(1)
C(36)	1996(3)	8181(2)	977(1)	23(1)
C(37)	609(3)	6821(2)	791(1)	27(1)
C(38)	623(4)	5933(3)	584(1)	38(1)
C(39)	6498(3)	8477(2)	1582(1)	20(1)
C(40)	7028(4)	9066(2)	1830(1)	24(1)
C(41)	7053(4)	8694(2)	2133(1)	26(1)
C(42)	6538(3)	7730(2)	2196(1)	23(1)
C(43)	6515(4)	7287(3)	2507(1)	28(1)
C(44)	7192(5)	7827(3)	2784(1)	41(1)
C(45)	5881(4)	6388(3)	2539(1)	33(1)
C(46)	5253(5)	5833(3)	2277(1)	33(1)
C(47)	5983(4)	7183(2)	1944(1)	24(1)
C(48)	5967(4)	7531(2)	1637(1)	25(1)
O(1)	-359(3)	4889(2)	1772(1)	27(1)
O(2)	-864(2)	3856(2)	1000(1)	26(1)
O(4)	271(3)	2182(2)	1270(1)	26(1)
C(4)	-2203(6)	3510(3)	533(1)	49(1)
O(5)	-606(4)	1513(2)	1719(1)	51(1)
O(6)	3381(2)	2809(2)	1426(1)	23(1)
O(7)	3627(3)	2017(2)	960(1)	43(1)
O(8)	4032(2)	5070(2)	1005(1)	25(1)
O(9)	6462(3)	5701(2)	1048(1)	43(1)
O(10)	1277(2)	5122(2)	1331(1)	19(1)
O(11)	1484(3)	8189(2)	1785(1)	31(1)
O(12)	2660(4)	9638(2)	1755(1)	45(1)
O(13)	6514(2)	8896(2)	1287(1)	21(1)
O(14)	6957(2)	8170(1)	482(1)	19(1)
O(15)	9243(3)	8884(2)	336(1)	44(1)
O(16)	5904(2)	10040(2)	352(1)	22(1)
O(17)	7509(3)	11148(2)	580(1)	32(1)
O(18)	2796(2)	9964(2)	618(1)	21(1)
O(19)	2691(3)	9841(2)	86(1)	31(1)
O(20)	1921(2)	7371(2)	757(1)	22(1)
O(21)	-426(3)	7028(2)	976(1)	44(1)
O(22)	4755(2)	7907(1)	998(1)	17(1)
O(23)	5386(3)	6256(2)	1983(1)	33(1)
O(24)	4660(4)	5024(2)	2288(1)	46(1)

Table S3. Bond lengths [Å] for (AcGC) (**5**).

C(1)-O(10)	1.396(3)
C(1)-O(1)	1.431(4)
C(1)-C(2)	1.529(4)
C(1)-H(1)	1.0000
C(2)-O(2)	1.437(3)
C(2)-C(5)	1.523(4)
C(2)-H(2)	1.0000
C(3)-O(3)	1.199(5)
C(3)-O(2)	1.351(4)
C(3)-C(4)	1.485(5)
C(5)-O(4)	1.430(3)
C(5)-C(8)	1.517(4)
C(5)-H(5)	1.0000
C(6)-O(5)	1.195(4)
C(6)-O(4)	1.348(4)
C(6)-C(7)	1.492(4)
C(7)-H(7A)	0.9800
C(7)-H(7B)	0.9800
C(7)-H(7C)	0.9800
C(8)-O(6)	1.436(3)
C(8)-C(11)	1.520(4)
C(8)-H(8)	1.0000
C(9)-O(7)	1.191(5)
C(9)-O(6)	1.367(4)
C(9)-C(10)	1.491(5)
C(10)-H(10A)	0.9800
C(10)-H(10B)	0.9800
C(10)-H(10C)	0.9800
C(11)-O(10)	1.433(3)
C(11)-C(12)	1.509(4)
C(11)-H(11)	1.0000
C(12)-O(8)	1.439(4)
C(12)-H(12A)	0.9900
C(12)-H(12B)	0.9900
C(13)-O(9)	1.199(5)
C(13)-O(8)	1.343(4)
C(13)-C(14)	1.490(5)

C(14)-H(14A)	0.9800
C(14)-H(14B)	0.9800
C(14)-H(14C)	0.9800
C(15)-O(1)	1.374(4)
C(15)-C(24)	1.387(4)
C(15)-C(16)	1.397(4)
C(16)-C(17)	1.377(5)
C(16)-H(16)	0.9500
C(17)-C(18)	1.409(4)
C(17)-H(17)	0.9500
C(18)-C(23)	1.399(4)
C(18)-C(19)	1.443(4)
C(19)-C(21)	1.343(5)
C(19)-C(20)	1.502(5)
C(20)-H(20A)	0.9800
C(20)-H(20B)	0.9800
C(20)-H(20C)	0.9800
C(21)-C(22)	1.449(5)
C(21)-H(21)	0.9500
C(22)-O(12)	1.211(4)
C(22)-O(11)	1.378(4)
C(23)-O(11)	1.369(4)
C(23)-C(24)	1.388(4)
C(24)-H(24)	0.9500
C(25)-O(22)	1.403(3)
C(25)-O(13)	1.424(3)
C(25)-C(26)	1.521(4)
C(25)-H(25)	1.0000
C(26)-O(14)	1.441(3)
C(26)-C(29)	1.521(4)
C(26)-H(26)	1.0000
C(27)-O(15)	1.194(4)
C(27)-O(14)	1.357(3)
C(27)-C(28)	1.490(4)
C(28)-H(28A)	0.9800
C(28)-H(28B)	0.9800
C(28)-H(28C)	0.9800
C(29)-O(16)	1.435(3)
C(29)-C(32)	1.513(3)

C(29)-H(29)	1.0000
C(30)-O(17)	1.204(4)
C(30)-O(16)	1.345(3)
C(30)-C(31)	1.500(4)
C(31)-H(31A)	0.9800
C(31)-H(31B)	0.9800
C(31)-H(31C)	0.9800
C(32)-O(18)	1.432(3)
C(32)-C(35)	1.524(4)
C(32)-H(32)	1.0000
C(33)-O(19)	1.196(4)
C(33)-O(18)	1.358(4)
C(33)-C(34)	1.499(4)
C(34)-H(34A)	0.9800
C(34)-H(34B)	0.9800
C(34)-H(34C)	0.9800
C(35)-O(22)	1.429(3)
C(35)-C(36)	1.508(4)
C(35)-H(35)	1.0000
C(36)-O(20)	1.443(4)
C(36)-H(36A)	0.9900
C(36)-H(36B)	0.9900
C(37)-O(21)	1.208(5)
C(37)-O(20)	1.346(3)
C(37)-C(38)	1.493(5)
C(38)-H(38A)	0.9800
C(38)-H(38B)	0.9800
C(38)-H(38C)	0.9800
C(39)-O(13)	1.374(3)
C(39)-C(48)	1.383(4)
C(39)-C(40)	1.395(4)
C(40)-C(41)	1.380(4)
C(40)-H(40)	0.9500
C(41)-C(42)	1.408(4)
C(41)-H(41)	0.9500
C(42)-C(47)	1.384(4)
C(42)-C(43)	1.447(4)
C(43)-C(45)	1.343(5)
C(43)-C(44)	1.497(5)

C(44)-H(44A)	0.9800
C(44)-H(44B)	0.9800
C(44)-H(44C)	0.9800
C(45)-C(46)	1.445(5)
C(45)-H(45)	0.9500
C(46)-O(24)	1.211(5)
C(46)-O(23)	1.375(4)
C(47)-O(23)	1.370(4)
C(47)-C(48)	1.387(4)
C(48)-H(48)	0.9500
C(4)-H(4A)	0.9800
C(4)-H(4B)	0.9800
C(4)-H(4C)	0.9800

Table S4. Bond angles [°] for (AcGC) (**5**).

O(10)-C(1)-O(1)	112.3(2)
O(10)-C(1)-C(2)	111.7(2)
O(1)-C(1)-C(2)	105.0(2)
O(10)-C(1)-H(1)	109.3
O(1)-C(1)-H(1)	109.3
C(2)-C(1)-H(1)	109.3
O(2)-C(2)-C(5)	107.5(2)
O(2)-C(2)-C(1)	106.2(2)
C(5)-C(2)-C(1)	111.3(2)
O(2)-C(2)-H(2)	110.6
C(5)-C(2)-H(2)	110.6
C(1)-C(2)-H(2)	110.6
O(3)-C(3)-O(2)	123.8(3)
O(3)-C(3)-C(4)	126.0(3)
O(2)-C(3)-C(4)	110.2(3)
O(4)-C(5)-C(8)	107.2(2)
O(4)-C(5)-C(2)	110.1(2)
C(8)-C(5)-C(2)	111.2(2)
O(4)-C(5)-H(5)	109.4
C(8)-C(5)-H(5)	109.4
C(2)-C(5)-H(5)	109.4
O(5)-C(6)-O(4)	123.1(3)
O(5)-C(6)-C(7)	126.5(3)
O(4)-C(6)-C(7)	110.3(3)
C(6)-C(7)-H(7A)	109.5
C(6)-C(7)-H(7B)	109.5
H(7A)-C(7)-H(7B)	109.5
C(6)-C(7)-H(7C)	109.5
H(7A)-C(7)-H(7C)	109.5
H(7B)-C(7)-H(7C)	109.5
O(6)-C(8)-C(5)	108.7(2)
O(6)-C(8)-C(11)	107.4(2)
C(5)-C(8)-C(11)	108.7(2)
O(6)-C(8)-H(8)	110.7
C(5)-C(8)-H(8)	110.7
C(11)-C(8)-H(8)	110.7
O(7)-C(9)-O(6)	123.8(3)

O(7)-C(9)-C(10)	126.5(3)
O(6)-C(9)-C(10)	109.7(3)
C(9)-C(10)-H(10A)	109.5
C(9)-C(10)-H(10B)	109.5
H(10A)-C(10)-H(10B)	109.5
C(9)-C(10)-H(10C)	109.5
H(10A)-C(10)-H(10C)	109.5
H(10B)-C(10)-H(10C)	109.5
O(10)-C(11)-C(12)	107.1(2)
O(10)-C(11)-C(8)	108.8(2)
C(12)-C(11)-C(8)	115.1(2)
O(10)-C(11)-H(11)	108.6
C(12)-C(11)-H(11)	108.6
C(8)-C(11)-H(11)	108.6
O(8)-C(12)-C(11)	108.0(2)
O(8)-C(12)-H(12A)	110.1
C(11)-C(12)-H(12A)	110.1
O(8)-C(12)-H(12B)	110.1
C(11)-C(12)-H(12B)	110.1
H(12A)-C(12)-H(12B)	108.4
O(9)-C(13)-O(8)	123.0(3)
O(9)-C(13)-C(14)	125.1(3)
O(8)-C(13)-C(14)	111.9(3)
C(13)-C(14)-H(14A)	109.5
C(13)-C(14)-H(14B)	109.5
H(14A)-C(14)-H(14B)	109.5
C(13)-C(14)-H(14C)	109.5
H(14A)-C(14)-H(14C)	109.5
H(14B)-C(14)-H(14C)	109.5
O(1)-C(15)-C(24)	123.5(3)
O(1)-C(15)-C(16)	115.4(3)
C(24)-C(15)-C(16)	121.0(3)
C(17)-C(16)-C(15)	119.6(3)
C(17)-C(16)-H(16)	120.2
C(15)-C(16)-H(16)	120.2
C(16)-C(17)-C(18)	121.3(3)
C(16)-C(17)-H(17)	119.3
C(18)-C(17)-H(17)	119.3
C(23)-C(18)-C(17)	116.9(3)

C(23)-C(18)-C(19)	118.1(3)
C(17)-C(18)-C(19)	125.1(3)
C(21)-C(19)-C(18)	118.8(3)
C(21)-C(19)-C(20)	121.0(3)
C(18)-C(19)-C(20)	120.2(3)
C(19)-C(20)-H(20A)	109.5
C(19)-C(20)-H(20B)	109.5
H(20A)-C(20)-H(20B)	109.5
C(19)-C(20)-H(20C)	109.5
H(20A)-C(20)-H(20C)	109.5
H(20B)-C(20)-H(20C)	109.5
C(19)-C(21)-C(22)	123.2(3)
C(19)-C(21)-H(21)	118.4
C(22)-C(21)-H(21)	118.4
O(12)-C(22)-O(11)	117.2(3)
O(12)-C(22)-C(21)	126.4(3)
O(11)-C(22)-C(21)	116.4(3)
O(11)-C(23)-C(24)	115.2(3)
O(11)-C(23)-C(18)	121.7(3)
C(24)-C(23)-C(18)	123.0(3)
C(15)-C(24)-C(23)	117.9(3)
C(15)-C(24)-H(24)	121.0
C(23)-C(24)-H(24)	121.0
O(22)-C(25)-O(13)	112.4(2)
O(22)-C(25)-C(26)	112.0(2)
O(13)-C(25)-C(26)	104.8(2)
O(22)-C(25)-H(25)	109.2
O(13)-C(25)-H(25)	109.2
C(26)-C(25)-H(25)	109.2
O(14)-C(26)-C(29)	109.0(2)
O(14)-C(26)-C(25)	106.2(2)
C(29)-C(26)-C(25)	111.4(2)
O(14)-C(26)-H(26)	110.1
C(29)-C(26)-H(26)	110.1
C(25)-C(26)-H(26)	110.1
O(15)-C(27)-O(14)	123.6(3)
O(15)-C(27)-C(28)	125.6(3)
O(14)-C(27)-C(28)	110.8(3)
C(27)-C(28)-H(28A)	109.5

C(27)-C(28)-H(28B)	109.5
H(28A)-C(28)-H(28B)	109.5
C(27)-C(28)-H(28C)	109.5
H(28A)-C(28)-H(28C)	109.5
H(28B)-C(28)-H(28C)	109.5
O(16)-C(29)-C(32)	107.6(2)
O(16)-C(29)-C(26)	110.1(2)
C(32)-C(29)-C(26)	112.0(2)
O(16)-C(29)-H(29)	109.0
C(32)-C(29)-H(29)	109.0
C(26)-C(29)-H(29)	109.0
O(17)-C(30)-O(16)	123.6(3)
O(17)-C(30)-C(31)	126.4(3)
O(16)-C(30)-C(31)	110.1(3)
C(30)-C(31)-H(31A)	109.5
C(30)-C(31)-H(31B)	109.5
H(31A)-C(31)-H(31B)	109.5
C(30)-C(31)-H(31C)	109.5
H(31A)-C(31)-H(31C)	109.5
H(31B)-C(31)-H(31C)	109.5
O(18)-C(32)-C(29)	108.7(2)
O(18)-C(32)-C(35)	106.6(2)
C(29)-C(32)-C(35)	108.2(2)
O(18)-C(32)-H(32)	111.1
C(29)-C(32)-H(32)	111.1
C(35)-C(32)-H(32)	111.1
O(19)-C(33)-O(18)	124.5(3)
O(19)-C(33)-C(34)	126.4(3)
O(18)-C(33)-C(34)	109.2(3)
C(33)-C(34)-H(34A)	109.5
C(33)-C(34)-H(34B)	109.5
H(34A)-C(34)-H(34B)	109.5
C(33)-C(34)-H(34C)	109.5
H(34A)-C(34)-H(34C)	109.5
H(34B)-C(34)-H(34C)	109.5
O(22)-C(35)-C(36)	107.2(2)
O(22)-C(35)-C(32)	109.3(2)
C(36)-C(35)-C(32)	115.0(2)
O(22)-C(35)-H(35)	108.4

C(36)-C(35)-H(35)	108.4
C(32)-C(35)-H(35)	108.4
O(20)-C(36)-C(35)	108.9(2)
O(20)-C(36)-H(36A)	109.9
C(35)-C(36)-H(36A)	109.9
O(20)-C(36)-H(36B)	109.9
C(35)-C(36)-H(36B)	109.9
H(36A)-C(36)-H(36B)	108.3
O(21)-C(37)-O(20)	122.5(3)
O(21)-C(37)-C(38)	125.1(3)
O(20)-C(37)-C(38)	112.4(3)
C(37)-C(38)-H(38A)	109.5
C(37)-C(38)-H(38B)	109.5
H(38A)-C(38)-H(38B)	109.5
C(37)-C(38)-H(38C)	109.5
H(38A)-C(38)-H(38C)	109.5
H(38B)-C(38)-H(38C)	109.5
O(13)-C(39)-C(48)	122.7(3)
O(13)-C(39)-C(40)	116.3(3)
C(48)-C(39)-C(40)	121.0(3)
C(41)-C(40)-C(39)	119.5(3)
C(41)-C(40)-H(40)	120.2
C(39)-C(40)-H(40)	120.2
C(40)-C(41)-C(42)	121.0(3)
C(40)-C(41)-H(41)	119.5
C(42)-C(41)-H(41)	119.5
C(47)-C(42)-C(41)	117.3(3)
C(47)-C(42)-C(43)	118.3(3)
C(41)-C(42)-C(43)	124.4(3)
C(45)-C(43)-C(42)	118.4(3)
C(45)-C(43)-C(44)	121.5(3)
C(42)-C(43)-C(44)	120.2(3)
C(43)-C(44)-H(44A)	109.5
C(43)-C(44)-H(44B)	109.5
H(44A)-C(44)-H(44B)	109.5
C(43)-C(44)-H(44C)	109.5
H(44A)-C(44)-H(44C)	109.5
H(44B)-C(44)-H(44C)	109.5
C(43)-C(45)-C(46)	123.2(3)

C(43)-C(45)-H(45)	118.4
C(46)-C(45)-H(45)	118.4
O(24)-C(46)-O(23)	116.7(3)
O(24)-C(46)-C(45)	126.8(3)
O(23)-C(46)-C(45)	116.5(3)
O(23)-C(47)-C(42)	121.8(3)
O(23)-C(47)-C(48)	115.1(3)
C(42)-C(47)-C(48)	123.1(3)
C(39)-C(48)-C(47)	118.0(3)
C(39)-C(48)-H(48)	121.0
C(47)-C(48)-H(48)	121.0
C(15)-O(1)-C(1)	117.3(2)
C(3)-O(2)-C(2)	116.0(2)
C(6)-O(4)-C(5)	118.7(2)
C(3)-C(4)-H(4A)	109.5
C(3)-C(4)-H(4B)	109.5
H(4A)-C(4)-H(4B)	109.5
C(3)-C(4)-H(4C)	109.5
H(4A)-C(4)-H(4C)	109.5
H(4B)-C(4)-H(4C)	109.5
C(9)-O(6)-C(8)	118.2(2)
C(13)-O(8)-C(12)	114.4(2)
C(1)-O(10)-C(11)	114.7(2)
C(23)-O(11)-C(22)	121.8(3)
C(39)-O(13)-C(25)	117.0(2)
C(27)-O(14)-C(26)	116.4(2)
C(30)-O(16)-C(29)	116.8(2)
C(33)-O(18)-C(32)	118.0(2)
C(37)-O(20)-C(36)	113.1(2)
C(25)-O(22)-C(35)	114.4(2)
C(47)-O(23)-C(46)	121.7(3)

Table S5. Anisotropic displacement parameters ($Å^2 x 10^3$) for (AcGC) (**5**). The anisotropic displacement factor exponent takes the form: $-2p^2[h^2 a^{*2}U^{11} + ... + 2 h k a^* b^* U^{12}]$

	U^{11}	U^{22}	U^{33}	U^{23}	U^{13}	U^{12}
C(1)	17(1)	26(1)	24(1)	-1(1)	5(1)	-3(1)
C(2)	18(1)	27(2)	22(1)	-1(1)	3(1)	-8(1)
C(3)	28(2)	17(1)	51(2)	-2(1)	-17(2)	0(1)
O(3)	22(1)	58(2)	83(2)	-7(2)	-5(1)	-18(1)
C(5)	24(1)	21(1)	17(1)	-1(1)	1(1)	-8(1)
C(6)	23(1)	21(1)	28(1)	1(1)	7(1)	-6(1)
C(7)	35(2)	23(2)	37(2)	-4(1)	6(1)	-5(1)
C(8)	17(1)	19(1)	20(1)	0(1)	0(1)	-4(1)
C(9)	23(1)	24(2)	46(2)	-13(1)	5(1)	-5(1)
C(10)	39(2)	29(2)	67(3)	-7(2)	2(2)	10(2)
C(11)	17(1)	21(1)	20(1)	1(1)	-2(1)	-3(1)
C(12)	18(1)	28(2)	25(1)	0(1)	-4(1)	-8(1)
C(13)	16(1)	20(1)	48(2)	6(1)	8(1)	-3(1)
C(14)	40(2)	48(2)	44(2)	13(2)	19(2)	0(2)
C(15)	27(2)	23(2)	25(1)	-2(1)	7(1)	-2(1)
C(16)	35(2)	25(2)	25(2)	0(1)	9(1)	-3(1)
C(17)	32(2)	34(2)	20(1)	1(1)	6(1)	1(1)
C(18)	23(1)	25(1)	24(1)	-1(1)	6(1)	2(1)
C(19)	25(1)	31(2)	29(2)	-5(1)	5(1)	1(1)
C(20)	43(2)	46(2)	28(2)	-7(2)	1(1)	-8(2)
C(21)	41(2)	31(2)	32(2)	-6(1)	2(1)	-8(1)
C(22)	39(2)	26(2)	34(2)	-2(1)	8(1)	-5(1)
C(23)	26(1)	21(1)	28(2)	0(1)	9(1)	0(1)
C(24)	30(2)	26(2)	23(1)	0(1)	5(1)	-2(1)
C(25)	15(1)	19(1)	18(1)	0(1)	0(1)	-4(1)
C(26)	15(1)	20(1)	17(1)	-2(1)	0(1)	-6(1)
C(27)	22(1)	27(2)	24(1)	5(1)	6(1)	2(1)
C(28)	39(2)	33(2)	23(2)	0(1)	10(1)	7(1)
C(29)	18(1)	18(1)	18(1)	0(1)	2(1)	-4(1)
C(30)	18(1)	20(1)	33(2)	7(1)	3(1)	-3(1)
C(31)	54(2)	41(2)	36(2)	15(2)	3(2)	-18(2)
C(32)	14(1)	18(1)	17(1)	0(1)	1(1)	-1(1)
C(33)	14(1)	28(2)	29(2)	7(1)	-4(1)	-4(1)
C(34)	30(2)	37(2)	47(2)	13(2)	-3(2)	12(1)

C(35)	16(1)	20(1)	17(1)	1(1)	3(1)	-1(1)
C(36)	18(1)	25(1)	27(1)	-2(1)	5(1)	-4(1)
C(37)	16(1)	27(2)	39(2)	14(1)	-9(1)	-8(1)
C(38)	39(2)	26(2)	49(2)	5(2)	-17(2)	-16(1)
C(39)	19(1)	24(1)	18(1)	1(1)	-2(1)	-2(1)
C(40)	28(2)	22(1)	24(1)	-3(1)	-2(1)	-4(1)
C(41)	31(2)	29(2)	19(1)	-6(1)	-4(1)	0(1)
C(42)	24(1)	26(1)	18(1)	-2(1)	0(1)	6(1)
C(43)	33(2)	35(2)	17(1)	1(1)	-1(1)	12(1)
C(44)	61(2)	46(2)	17(2)	-3(1)	-7(2)	11(2)
C(45)	48(2)	33(2)	20(1)	7(1)	2(1)	9(1)
C(46)	45(2)	29(2)	26(2)	10(1)	3(1)	2(1)
C(47)	29(2)	22(1)	21(1)	3(1)	-2(1)	-1(1)
C(48)	33(2)	22(1)	19(1)	2(1)	-4(1)	-6(1)
O(1)	35(1)	24(1)	23(1)	-2(1)	10(1)	-7(1)
O(2)	18(1)	34(1)	25(1)	3(1)	-4(1)	-8(1)
O(4)	31(1)	24(1)	22(1)	-3(1)	3(1)	-14(1)
C(4)	75(3)	25(2)	48(2)	1(2)	-39(2)	-7(2)
O(5)	89(2)	32(1)	32(1)	-3(1)	27(1)	-21(1)
O(6)	24(1)	20(1)	25(1)	-2(1)	-1(1)	2(1)
O(7)	29(1)	54(2)	44(2)	-29(1)	-1(1)	-4(1)
O(8)	16(1)	31(1)	27(1)	2(1)	2(1)	-10(1)
O(9)	18(1)	36(1)	76(2)	13(1)	-3(1)	-11(1)
O(10)	17(1)	21(1)	21(1)	2(1)	2(1)	-4(1)
O(11)	43(1)	22(1)	27(1)	2(1)	3(1)	-5(1)
O(12)	62(2)	30(1)	44(2)	5(1)	5(1)	-15(1)
O(13)	26(1)	21(1)	17(1)	0(1)	-3(1)	-7(1)
O(14)	17(1)	21(1)	20(1)	-2(1)	4(1)	-3(1)
O(15)	34(1)	53(2)	47(2)	-10(1)	20(1)	-20(1)
O(16)	22(1)	23(1)	22(1)	6(1)	0(1)	-9(1)
O(17)	30(1)	26(1)	40(1)	0(1)	-2(1)	-11(1)
O(18)	19(1)	21(1)	23(1)	2(1)	-1(1)	3(1)
O(19)	34(1)	34(1)	24(1)	4(1)	-5(1)	-3(1)
O(20)	17(1)	24(1)	27(1)	0(1)	2(1)	-8(1)
O(21)	14(1)	44(2)	75(2)	12(1)	10(1)	-6(1)
O(22)	16(1)	17(1)	19(1)	2(1)	-1(1)	-4(1)
O(23)	50(1)	27(1)	22(1)	9(1)	-5(1)	-11(1)
O(24)	67(2)	33(1)	38(1)	16(1)	-1(1)	-11(1)

Table S6. Hydrogen coordinates (x 10^4) and isotropic displacement parameters ($Å^2$x 10^3) for (AcGC) (**5**).

	x	y	z	U(eq)
H(1)	-1016	5360	1345	27
H(2)	-1732	3648	1439	27
H(5)	621	3009	1653	25
H(7A)	-599	-46	1394	48
H(7B)	808	366	1179	48
H(7C)	-992	572	1082	48
H(8)	2218	3465	1067	23
H(10A)	4586	927	1502	68
H(10B)	5636	1878	1575	68
H(10C)	5959	1246	1263	68
H(11)	2456	4433	1673	23
H(12A)	4208	5558	1451	29
H(12B)	4939	4480	1400	29
H(14A)	4641	6351	517	66
H(14B)	4555	5199	444	66
H(14C)	6229	5748	449	66
H(16)	-9	5045	2357	34
H(17)	980	6353	2648	34
H(20A)	1443	7993	2898	59
H(20B)	2971	8669	2848	59
H(20C)	3123	7499	2826	59
H(21)	3062	9420	2365	42
H(24)	257	6681	1555	31
H(25)	7046	7680	1048	21
H(26)	7847	9182	780	21
H(28A)	8138	6856	138	48
H(28B)	9284	7534	-69	48
H(28C)	7407	7635	-105	48
H(29)	5580	10151	818	22
H(31A)	7591	11826	18	65
H(31B)	6073	11209	-89	65
H(31C)	7790	10716	-100	65
H(32)	3821	8719	458	19
H(34A)	1871	11652	472	57

H(34B)	324	10975	464	57
H(34C)	1049	11391	142	57
H(35)	3703	9148	1127	21
H(36A)	1824	7939	1195	28
H(36B)	1159	8665	927	28
H(38A)	-464	5704	552	57
H(38B)	1242	5412	686	57
H(38C)	1099	6099	380	57
H(40)	7371	9720	1791	29
H(41)	7423	9093	2301	31
H(44A)	7137	7410	2972	62
H(44B)	6584	8430	2820	62
H(44C)	8297	7996	2741	62
H(45)	5845	6104	2744	40
H(48)	5602	7131	1468	30
H(4A)	-1422	3025	462	74
H(4B)	-1906	4162	455	74
H(4C)	-3248	3333	451	74

Table S7. Torsion angles [°] for (AcGC) (**5**).

O(10)-C(1)-C(2)-O(2)	-67.7(3)
O(1)-C(1)-C(2)-O(2)	170.4(2)
O(10)-C(1)-C(2)-C(5)	49.0(3)
O(1)-C(1)-C(2)-C(5)	-72.9(3)
O(2)-C(2)-C(5)-O(4)	-52.2(3)
C(1)-C(2)-C(5)-O(4)	-168.1(2)
O(2)-C(2)-C(5)-C(8)	66.5(3)
C(1)-C(2)-C(5)-C(8)	-49.4(3)
O(4)-C(5)-C(8)-O(6)	-68.0(3)
C(2)-C(5)-C(8)-O(6)	171.6(2)
O(4)-C(5)-C(8)-C(11)	175.5(2)
C(2)-C(5)-C(8)-C(11)	55.0(3)
O(6)-C(8)-C(11)-O(10)	-177.2(2)
C(5)-C(8)-C(11)-O(10)	-59.8(3)
O(6)-C(8)-C(11)-C(12)	62.7(3)
C(5)-C(8)-C(11)-C(12)	-179.9(2)
O(10)-C(11)-C(12)-O(8)	-56.8(3)
C(8)-C(11)-C(12)-O(8)	64.2(3)
O(1)-C(15)-C(16)-C(17)	174.9(3)
C(24)-C(15)-C(16)-C(17)	-4.0(5)
C(15)-C(16)-C(17)-C(18)	-0.7(5)
C(16)-C(17)-C(18)-C(23)	3.8(5)
C(16)-C(17)-C(18)-C(19)	-174.1(3)
C(23)-C(18)-C(19)-C(21)	-0.9(4)
C(17)-C(18)-C(19)-C(21)	177.0(3)
C(23)-C(18)-C(19)-C(20)	-179.0(3)
C(17)-C(18)-C(19)-C(20)	-1.0(5)
C(18)-C(19)-C(21)-C(22)	1.2(5)
C(20)-C(19)-C(21)-C(22)	179.2(3)
C(19)-C(21)-C(22)-O(12)	-178.3(4)
C(19)-C(21)-C(22)-O(11)	1.0(5)
C(17)-C(18)-C(23)-O(11)	-179.7(3)
C(19)-C(18)-C(23)-O(11)	-1.6(4)
C(17)-C(18)-C(23)-C(24)	-2.6(4)
C(19)-C(18)-C(23)-C(24)	175.5(3)
O(1)-C(15)-C(24)-C(23)	-173.7(3)
C(16)-C(15)-C(24)-C(23)	5.1(5)

O(11)-C(23)-C(24)-C(15)	175.5(3)
C(18)-C(23)-C(24)-C(15)	-1.7(5)
O(22)-C(25)-C(26)-O(14)	-70.3(3)
O(13)-C(25)-C(26)-O(14)	167.5(2)
O(22)-C(25)-C(26)-C(29)	48.2(3)
O(13)-C(25)-C(26)-C(29)	-73.9(3)
O(14)-C(26)-C(29)-O(16)	-51.7(3)
C(25)-C(26)-C(29)-O(16)	-168.6(2)
O(14)-C(26)-C(29)-C(32)	68.0(3)
C(25)-C(26)-C(29)-C(32)	-48.9(3)
O(16)-C(29)-C(32)-O(18)	-69.1(3)
C(26)-C(29)-C(32)-O(18)	169.7(2)
O(16)-C(29)-C(32)-C(35)	175.5(2)
C(26)-C(29)-C(32)-C(35)	54.3(3)
O(18)-C(32)-C(35)-O(22)	-176.3(2)
C(29)-C(32)-C(35)-O(22)	-59.5(3)
O(18)-C(32)-C(35)-C(36)	63.1(3)
C(29)-C(32)-C(35)-C(36)	179.8(2)
O(22)-C(35)-C(36)-O(20)	-54.5(3)
C(32)-C(35)-C(36)-O(20)	67.2(3)
O(13)-C(39)-C(40)-C(41)	-179.7(3)
C(48)-C(39)-C(40)-C(41)	1.2(5)
C(39)-C(40)-C(41)-C(42)	-0.5(5)
C(40)-C(41)-C(42)-C(47)	-1.1(5)
C(40)-C(41)-C(42)-C(43)	-179.5(3)
C(47)-C(42)-C(43)-C(45)	-3.4(4)
C(41)-C(42)-C(43)-C(45)	174.9(3)
C(47)-C(42)-C(43)-C(44)	176.4(3)
C(41)-C(42)-C(43)-C(44)	-5.3(5)
C(42)-C(43)-C(45)-C(46)	1.4(5)
C(44)-C(43)-C(45)-C(46)	-178.4(3)
C(43)-C(45)-C(46)-O(24)	-179.6(4)
C(43)-C(45)-C(46)-O(23)	2.5(5)
C(41)-C(42)-C(47)-O(23)	-176.9(3)
C(43)-C(42)-C(47)-O(23)	1.6(5)
C(41)-C(42)-C(47)-C(48)	2.2(5)
C(43)-C(42)-C(47)-C(48)	-179.3(3)
O(13)-C(39)-C(48)-C(47)	-179.3(3)
C(40)-C(39)-C(48)-C(47)	-0.2(5)

O(23)-C(47)-C(48)-C(39)	177.6(3)
C(42)-C(47)-C(48)-C(39)	-1.6(5)
C(24)-C(15)-O(1)-C(1)	11.2(4)
C(16)-C(15)-O(1)-C(1)	-167.7(3)
O(10)-C(1)-O(1)-C(15)	53.6(3)
C(2)-C(1)-O(1)-C(15)	175.1(2)
O(3)-C(3)-O(2)-C(2)	4.9(5)
C(4)-C(3)-O(2)-C(2)	-175.7(3)
C(5)-C(2)-O(2)-C(3)	114.3(3)
C(1)-C(2)-O(2)-C(3)	-126.5(3)
O(5)-C(6)-O(4)-C(5)	8.7(5)
C(7)-C(6)-O(4)-C(5)	-171.4(3)
C(8)-C(5)-O(4)-C(6)	132.1(3)
C(2)-C(5)-O(4)-C(6)	-106.8(3)
O(7)-C(9)-O(6)-C(8)	2.2(4)
C(10)-C(9)-O(6)-C(8)	-176.9(3)
C(5)-C(8)-O(6)-C(9)	101.4(3)
C(11)-C(8)-O(6)-C(9)	-141.2(2)
O(9)-C(13)-O(8)-C(12)	4.2(4)
C(14)-C(13)-O(8)-C(12)	-174.8(3)
C(11)-C(12)-O(8)-C(13)	177.7(3)
O(1)-C(1)-O(10)-C(11)	60.3(3)
C(2)-C(1)-O(10)-C(11)	-57.3(3)
C(12)-C(11)-O(10)-C(1)	-172.0(2)
C(8)-C(11)-O(10)-C(1)	63.1(3)
C(24)-C(23)-O(11)-C(22)	-173.3(3)
C(18)-C(23)-O(11)-C(22)	3.9(5)
O(12)-C(22)-O(11)-C(23)	175.8(3)
C(21)-C(22)-O(11)-C(23)	-3.6(5)
C(48)-C(39)-O(13)-C(25)	-14.3(4)
C(40)-C(39)-O(13)-C(25)	166.7(2)
O(22)-C(25)-O(13)-C(39)	72.5(3)
C(26)-C(25)-O(13)-C(39)	-165.6(2)
O(15)-C(27)-O(14)-C(26)	-0.8(4)
C(28)-C(27)-O(14)-C(26)	179.3(2)
C(29)-C(26)-O(14)-C(27)	104.7(3)
C(25)-C(26)-O(14)-C(27)	-135.2(2)
O(17)-C(30)-O(16)-C(29)	1.3(4)
C(31)-C(30)-O(16)-C(29)	-179.3(3)

C(32)-C(29)-O(16)-C(30)	150.7(2)
C(26)-C(29)-O(16)-C(30)	-87.0(3)
O(19)-C(33)-O(18)-C(32)	5.9(4)
C(34)-C(33)-O(18)-C(32)	-173.4(2)
C(29)-C(32)-O(18)-C(33)	93.2(3)
C(35)-C(32)-O(18)-C(33)	-150.4(2)
O(21)-C(37)-O(20)-C(36)	3.7(4)
C(38)-C(37)-O(20)-C(36)	-174.4(3)
C(35)-C(36)-O(20)-C(37)	172.0(2)
O(13)-C(25)-O(22)-C(35)	60.9(3)
C(26)-C(25)-O(22)-C(35)	-56.8(3)
C(36)-C(35)-O(22)-C(25)	-171.8(2)
C(32)-C(35)-O(22)-C(25)	62.9(3)
C(42)-C(47)-O(23)-C(46)	2.5(5)
C(48)-C(47)-O(23)-C(46)	-176.7(3)
O(24)-C(46)-O(23)-C(47)	177.5(3)
C(45)-C(46)-O(23)-C(47)	-4.4(5)

Table S8. Crystal data and structure refinement for CG (**4**).

Identification code	CG (**4**)	
Empirical formula	C16 H18 O8	
Formula weight	338.30	
Temperature	150.01(10) K	
Wavelength	1.54184 Å	
Crystal system	Triclinic	
Space group	P1	
Unit cell dimensions	a = 7.9134(3) Å	a= 105.567(4)°.
	b = 7.9212(4) Å	b= 104.160(4)°.
	c = 12.5663(6) Å	g = 91.397(3)°.
Volume	732.32(6) $Å^3$	
Z	2	
Density (calculated)	1.534 Mg/m^3	
Absorption coefficient	1.060 mm^{-1}	
F(000)	356	
Crystal size	0.150 x 0.090 x 0.020 mm^3	
Theta range for data collection	3.782 to 73.044°.	
Index ranges	-9<=h<=8, -9<=k<=9, -15<=l<=13	
Reflections collected	4688	
Independent reflections	3355 [R(int) = 0.0226]	
Completeness to theta = 67.684°	99.8 %	
Absorption correction	Semi-empirical from equivalents	
Max. and min. transmission	1.00000 and 0.82567	
Refinement method	Full-matrix least-squares on F^2	
Data / restraints / parameters	3355 / 3 / 467	
Goodness-of-fit on F^2	1.032	
Final R indices [I>2sigma(I)]	R1 = 0.0296, wR2 = 0.0770	
R indices (all data)	R1 = 0.0307, wR2 = 0.0782	
Absolute structure parameter	-0.03(12)	
Extinction coefficient	n/a	
Largest diff. peak and hole	0.220 and -0.193 e.$Å^{-3}$	

Table S9. Atomic coordinates (x 10^4) and equivalent isotropic displacement parameters (Å^{2}x 10^3) for CG (**4**). U(eq) is defined as one third of the trace of the orthogonalized U^{ij} tensor.

	x	y	z	U(eq)
O(1)	3714(3)	4385(3)	2692(2)	30(1)
O(2)	2933(2)	5177(2)	4305(2)	22(1)
O(3)	1440(2)	6648(2)	7873(1)	16(1)
O(4)	2459(2)	3858(2)	7782(1)	14(1)
O(5)	5990(2)	3244(2)	7734(2)	19(1)
O(6)	4638(2)	5162(3)	10839(2)	22(1)
O(7)	992(2)	5642(2)	10851(2)	20(1)
O(8)	-433(2)	3157(2)	8700(2)	19(1)
C(1)	3437(3)	5607(4)	3430(2)	22(1)
C(2)	3575(3)	7453(4)	3477(2)	26(1)
C(3)	3278(3)	8732(4)	4344(2)	22(1)
C(4)	3399(4)	10647(4)	4377(3)	32(1)
C(5)	2809(3)	8241(3)	5272(2)	19(1)
C(6)	2508(3)	9442(3)	6241(2)	19(1)
C(7)	2035(3)	8877(3)	7082(2)	18(1)
C(8)	1889(3)	7069(3)	6984(2)	15(1)
C(9)	2193(3)	5841(3)	6045(2)	16(1)
C(10)	2634(3)	6459(3)	5208(2)	17(1)
C(11)	1010(3)	4834(3)	7771(2)	13(1)
C(12)	3863(3)	4466(3)	8815(2)	14(1)
C(13)	5303(3)	3266(3)	8692(2)	17(1)
C(14)	3209(3)	4449(3)	9864(2)	15(1)
C(15)	1665(3)	5554(3)	9883(2)	14(1)
C(16)	232(3)	4890(3)	8776(2)	15(1)
O(9)	6565(2)	6562(2)	7361(2)	20(1)
O(10)	7323(2)	5680(2)	5740(2)	18(1)
O(11)	8941(2)	3538(2)	2225(1)	16(1)
O(12)	7326(2)	1044(2)	2251(1)	14(1)
O(13)	3149(2)	-34(2)	98(2)	20(1)
O(14)	5845(2)	-867(2)	-839(1)	18(1)
O(15)	9539(2)	-1012(2)	-416(2)	18(1)
O(16)	10448(2)	-706(2)	1938(2)	21(1)
C(17)	7020(3)	7002(3)	6604(2)	16(1)
C(18)	7264(3)	8792(3)	6551(2)	19(1)

C(19)	7730(3)	9163(3)	5675(2)	18(1)
C(20)	7986(4)	11018(3)	5624(2)	26(1)
C(21)	8013(3)	7739(3)	4757(2)	16(1)
C(22)	8463(3)	7958(3)	3785(2)	20(1)
C(23)	8740(3)	6540(3)	2959(2)	18(1)
C(24)	8560(3)	4847(3)	3077(2)	14(1)
C(25)	8057(3)	4563(3)	4003(2)	16(1)
C(26)	7804(3)	6026(3)	4830(2)	15(1)
C(27)	9001(3)	1828(3)	2388(2)	14(1)
C(28)	6212(3)	761(3)	1112(2)	13(1)
C(29)	4420(3)	60(3)	1142(2)	17(1)
C(30)	7012(3)	-536(3)	273(2)	14(1)
C(31)	8804(3)	270(3)	320(2)	14(1)
C(32)	9968(3)	776(3)	1549(2)	16(1)

Table S10. Bond lengths [Å] for CG (**4**).

O(1)-C(1)	1.213(4)
O(2)-C(1)	1.376(3)
O(2)-C(10)	1.378(3)
O(3)-C(8)	1.368(3)
O(3)-C(11)	1.433(3)
O(4)-C(11)	1.398(3)
O(4)-C(12)	1.444(3)
O(5)-C(13)	1.433(3)
O(5)-H(5A)	0.93(4)
O(6)-C(14)	1.421(3)
O(6)-H(6A)	0.80(4)
O(7)-C(15)	1.429(3)
O(7)-H(7A)	0.87(5)
O(8)-C(16)	1.428(3)
O(8)-H(8A)	0.74(4)
C(1)-C(2)	1.448(4)
C(2)-C(3)	1.347(4)
C(2)-H(2)	0.9500
C(3)-C(5)	1.447(3)
C(3)-C(4)	1.506(4)
C(4)-H(4A)	0.9800
C(4)-H(4B)	0.9800
C(4)-H(4C)	0.9800
C(5)-C(10)	1.394(3)
C(5)-C(6)	1.405(4)
C(6)-C(7)	1.378(3)
C(6)-H(6)	0.9500
C(7)-C(8)	1.403(3)
C(7)-H(7)	0.9500
C(8)-C(9)	1.390(3)
C(9)-C(10)	1.383(3)
C(9)-H(9)	0.9500
C(11)-C(16)	1.525(3)
C(11)-H(11)	1.0000
C(12)-C(13)	1.512(3)
C(12)-C(14)	1.532(3)
C(12)-H(12)	1.0000

C(13)-H(13A)	0.9900
C(13)-H(13B)	0.9900
C(14)-C(15)	1.521(3)
C(14)-H(14)	1.0000
C(15)-C(16)	1.518(3)
C(15)-H(15)	1.0000
C(16)-H(16)	1.0000
O(9)-C(17)	1.223(3)
O(10)-C(17)	1.364(3)
O(10)-C(26)	1.379(3)
O(11)-C(24)	1.374(3)
O(11)-C(27)	1.423(3)
O(12)-C(27)	1.401(3)
O(12)-C(28)	1.441(3)
O(13)-C(29)	1.428(3)
O(13)-H(13)	0.90(5)
O(14)-C(30)	1.426(3)
O(14)-H(14A)	0.80(4)
O(15)-C(31)	1.418(3)
O(15)-H(15A)	0.88(5)
O(16)-C(32)	1.414(3)
O(16)-H(16A)	0.84(4)
C(17)-C(18)	1.447(3)
C(18)-C(19)	1.342(3)
C(18)-H(18)	0.9500
C(19)-C(21)	1.447(3)
C(19)-C(20)	1.499(3)
C(20)-H(20A)	0.9800
C(20)-H(20B)	0.9800
C(20)-H(20C)	0.9800
C(21)-C(26)	1.393(3)
C(21)-C(22)	1.404(3)
C(22)-C(23)	1.373(3)
C(22)-H(22)	0.9500
C(23)-C(24)	1.396(3)
C(23)-H(23)	0.9500
C(24)-C(25)	1.387(3)
C(25)-C(26)	1.390(3)
C(25)-H(25)	0.9500

C(27)-C(32)	1.531(3)
C(27)-H(27)	1.0000
C(28)-C(29)	1.523(3)
C(28)-C(30)	1.531(3)
C(28)-H(28)	1.0000
C(29)-H(29A)	0.9900
C(29)-H(29B)	0.9900
C(30)-C(31)	1.522(3)
C(30)-H(30)	1.0000
C(31)-C(32)	1.532(3)
C(31)-H(31)	1.0000
C(32)-H(32)	1.0000

Table S11. Bond angles [°] for CG (**4**).

C(1)-O(2)-C(10)	121.2(2)
C(8)-O(3)-C(11)	118.79(18)
C(11)-O(4)-C(12)	114.12(16)
C(13)-O(5)-H(5A)	111(2)
C(14)-O(6)-H(6A)	108(2)
C(15)-O(7)-H(7A)	108(3)
C(16)-O(8)-H(8A)	110(3)
O(1)-C(1)-O(2)	116.1(2)
O(1)-C(1)-C(2)	126.7(3)
O(2)-C(1)-C(2)	117.2(2)
C(3)-C(2)-C(1)	122.8(2)
C(3)-C(2)-H(2)	118.6
C(1)-C(2)-H(2)	118.6
C(2)-C(3)-C(5)	118.6(2)
C(2)-C(3)-C(4)	122.4(2)
C(5)-C(3)-C(4)	119.0(3)
C(3)-C(4)-H(4A)	109.5
C(3)-C(4)-H(4B)	109.5
H(4A)-C(4)-H(4B)	109.5
C(3)-C(4)-H(4C)	109.5
H(4A)-C(4)-H(4C)	109.5
H(4B)-C(4)-H(4C)	109.5
C(10)-C(5)-C(6)	117.1(2)
C(10)-C(5)-C(3)	118.4(2)
C(6)-C(5)-C(3)	124.5(2)
C(7)-C(6)-C(5)	121.3(2)
C(7)-C(6)-H(6)	119.3
C(5)-C(6)-H(6)	119.3
C(6)-C(7)-C(8)	119.4(2)
C(6)-C(7)-H(7)	120.3
C(8)-C(7)-H(7)	120.3
O(3)-C(8)-C(9)	124.2(2)
O(3)-C(8)-C(7)	114.9(2)
C(9)-C(8)-C(7)	121.0(2)
C(10)-C(9)-C(8)	117.8(2)
C(10)-C(9)-H(9)	121.1
C(8)-C(9)-H(9)	121.1

O(2)-C(10)-C(9)	115.0(2)
O(2)-C(10)-C(5)	121.7(2)
C(9)-C(10)-C(5)	123.3(2)
O(4)-C(11)-O(3)	112.52(18)
O(4)-C(11)-C(16)	112.85(18)
O(3)-C(11)-C(16)	104.25(17)
O(4)-C(11)-H(11)	109.0
O(3)-C(11)-H(11)	109.0
C(16)-C(11)-H(11)	109.0
O(4)-C(12)-C(13)	107.32(17)
O(4)-C(12)-C(14)	110.58(16)
C(13)-C(12)-C(14)	111.36(18)
O(4)-C(12)-H(12)	109.2
C(13)-C(12)-H(12)	109.2
C(14)-C(12)-H(12)	109.2
O(5)-C(13)-C(12)	114.07(18)
O(5)-C(13)-H(13A)	108.7
C(12)-C(13)-H(13A)	108.7
O(5)-C(13)-H(13B)	108.7
C(12)-C(13)-H(13B)	108.7
H(13A)-C(13)-H(13B)	107.6
O(6)-C(14)-C(15)	111.62(18)
O(6)-C(14)-C(12)	106.71(17)
C(15)-C(14)-C(12)	108.79(17)
O(6)-C(14)-H(14)	109.9
C(15)-C(14)-H(14)	109.9
C(12)-C(14)-H(14)	109.9
O(7)-C(15)-C(16)	110.97(17)
O(7)-C(15)-C(14)	112.62(18)
C(16)-C(15)-C(14)	110.82(18)
O(7)-C(15)-H(15)	107.4
C(16)-C(15)-H(15)	107.4
C(14)-C(15)-H(15)	107.4
O(8)-C(16)-C(15)	108.14(18)
O(8)-C(16)-C(11)	109.74(18)
C(15)-C(16)-C(11)	108.98(17)
O(8)-C(16)-H(16)	110.0
C(15)-C(16)-H(16)	110.0
C(11)-C(16)-H(16)	110.0

C(17)-O(10)-C(26)	121.38(18)
C(24)-O(11)-C(27)	116.60(17)
C(27)-O(12)-C(28)	112.83(16)
C(29)-O(13)-H(13)	109(3)
C(30)-O(14)-H(14A)	107(3)
C(31)-O(15)-H(15A)	115(3)
C(32)-O(16)-H(16A)	108(2)
O(9)-C(17)-O(10)	116.6(2)
O(9)-C(17)-C(18)	125.7(2)
O(10)-C(17)-C(18)	117.8(2)
C(19)-C(18)-C(17)	122.0(2)
C(19)-C(18)-H(18)	119.0
C(17)-C(18)-H(18)	119.0
C(18)-C(19)-C(21)	119.4(2)
C(18)-C(19)-C(20)	121.9(2)
C(21)-C(19)-C(20)	118.8(2)
C(19)-C(20)-H(20A)	109.5
C(19)-C(20)-H(20B)	109.5
H(20A)-C(20)-H(20B)	109.5
C(19)-C(20)-H(20C)	109.5
H(20A)-C(20)-H(20C)	109.5
H(20B)-C(20)-H(20C)	109.5
C(26)-C(21)-C(22)	117.4(2)
C(26)-C(21)-C(19)	118.0(2)
C(22)-C(21)-C(19)	124.6(2)
C(23)-C(22)-C(21)	121.1(2)
C(23)-C(22)-H(22)	119.4
C(21)-C(22)-H(22)	119.4
C(22)-C(23)-C(24)	119.7(2)
C(22)-C(23)-H(23)	120.1
C(24)-C(23)-H(23)	120.1
O(11)-C(24)-C(25)	124.2(2)
O(11)-C(24)-C(23)	114.7(2)
C(25)-C(24)-C(23)	121.1(2)
C(24)-C(25)-C(26)	117.8(2)
C(24)-C(25)-H(25)	121.1
C(26)-C(25)-H(25)	121.1
O(10)-C(26)-C(25)	115.7(2)
O(10)-C(26)-C(21)	121.5(2)

C(25)-C(26)-C(21)	122.8(2)
O(12)-C(27)-O(11)	111.81(17)
O(12)-C(27)-C(32)	113.44(18)
O(11)-C(27)-C(32)	106.33(17)
O(12)-C(27)-H(27)	108.4
O(11)-C(27)-H(27)	108.4
C(32)-C(27)-H(27)	108.4
O(12)-C(28)-C(29)	105.89(17)
O(12)-C(28)-C(30)	108.45(17)
C(29)-C(28)-C(30)	113.01(17)
O(12)-C(28)-H(28)	109.8
C(29)-C(28)-H(28)	109.8
C(30)-C(28)-H(28)	109.8
O(13)-C(29)-C(28)	110.51(18)
O(13)-C(29)-H(29A)	109.5
C(28)-C(29)-H(29A)	109.5
O(13)-C(29)-H(29B)	109.5
C(28)-C(29)-H(29B)	109.5
H(29A)-C(29)-H(29B)	108.1
O(14)-C(30)-C(31)	111.47(17)
O(14)-C(30)-C(28)	107.50(17)
C(31)-C(30)-C(28)	108.98(17)
O(14)-C(30)-H(30)	109.6
C(31)-C(30)-H(30)	109.6
C(28)-C(30)-H(30)	109.6
O(15)-C(31)-C(30)	107.10(17)
O(15)-C(31)-C(32)	110.86(18)
C(30)-C(31)-C(32)	110.20(18)
O(15)-C(31)-H(31)	109.5
C(30)-C(31)-H(31)	109.5
C(32)-C(31)-H(31)	109.5
O(16)-C(32)-C(27)	105.23(19)
O(16)-C(32)-C(31)	112.70(18)
C(27)-C(32)-C(31)	110.93(17)
O(16)-C(32)-H(32)	109.3
C(27)-C(32)-H(32)	109.3
C(31)-C(32)-H(32)	109.3

Table S12. Anisotropic displacement parameters ($Å^2 x 10^3$) for CG (**4**). The anisotropic displacement factor exponent takes the form: $-2p^2[h^2 a^{*2}U^{11} + ... + 2 h k a^* b^* U^{12}]$

	U^{11}	U^{22}	U^{33}	U^{23}	U^{13}	U^{12}
O(1)	32(1)	44(1)	18(1)	11(1)	12(1)	13(1)
O(2)	26(1)	27(1)	16(1)	8(1)	9(1)	6(1)
O(3)	21(1)	15(1)	15(1)	6(1)	7(1)	3(1)
O(4)	14(1)	16(1)	13(1)	3(1)	5(1)	3(1)
O(5)	18(1)	20(1)	23(1)	6(1)	12(1)	2(1)
O(6)	16(1)	35(1)	13(1)	6(1)	3(1)	2(1)
O(7)	22(1)	25(1)	16(1)	5(1)	11(1)	5(1)
O(8)	14(1)	18(1)	26(1)	9(1)	5(1)	-1(1)
C(1)	18(1)	38(1)	15(1)	12(1)	5(1)	7(1)
C(2)	22(1)	38(2)	20(1)	13(1)	7(1)	-2(1)
C(3)	18(1)	30(1)	18(1)	11(1)	-1(1)	-3(1)
C(4)	43(2)	32(2)	23(1)	15(1)	3(1)	-9(1)
C(5)	14(1)	26(1)	16(1)	9(1)	1(1)	0(1)
C(6)	19(1)	20(1)	17(1)	7(1)	1(1)	-1(1)
C(7)	16(1)	20(1)	17(1)	5(1)	1(1)	3(1)
C(8)	11(1)	21(1)	12(1)	7(1)	1(1)	2(1)
C(9)	17(1)	17(1)	16(1)	6(1)	4(1)	3(1)
C(10)	14(1)	24(1)	14(1)	6(1)	4(1)	3(1)
C(11)	12(1)	12(1)	14(1)	3(1)	2(1)	3(1)
C(12)	14(1)	15(1)	15(1)	4(1)	6(1)	1(1)
C(13)	16(1)	20(1)	18(1)	7(1)	8(1)	5(1)
C(14)	13(1)	20(1)	13(1)	6(1)	5(1)	2(1)
C(15)	16(1)	15(1)	14(1)	5(1)	9(1)	3(1)
C(16)	14(1)	14(1)	18(1)	5(1)	6(1)	4(1)
O(9)	23(1)	22(1)	17(1)	4(1)	10(1)	2(1)
O(10)	23(1)	15(1)	17(1)	4(1)	9(1)	2(1)
O(11)	19(1)	13(1)	15(1)	2(1)	7(1)	1(1)
O(12)	14(1)	18(1)	12(1)	4(1)	4(1)	1(1)
O(13)	11(1)	27(1)	20(1)	3(1)	3(1)	2(1)
O(14)	14(1)	22(1)	14(1)	1(1)	4(1)	3(1)
O(15)	14(1)	20(1)	20(1)	-1(1)	9(1)	2(1)
O(16)	24(1)	18(1)	19(1)	5(1)	3(1)	8(1)
C(17)	13(1)	19(1)	12(1)	0(1)	2(1)	0(1)
C(18)	20(1)	17(1)	18(1)	1(1)	7(1)	3(1)

C(19)	16(1)	17(1)	18(1)	3(1)	3(1)	1(1)
C(20)	39(2)	15(1)	28(1)	4(1)	18(1)	5(1)
C(21)	13(1)	16(1)	17(1)	4(1)	3(1)	2(1)
C(22)	23(1)	16(1)	24(1)	7(1)	9(1)	2(1)
C(23)	20(1)	19(1)	18(1)	6(1)	7(1)	0(1)
C(24)	11(1)	15(1)	13(1)	1(1)	3(1)	0(1)
C(25)	17(1)	13(1)	16(1)	3(1)	4(1)	1(1)
C(26)	13(1)	16(1)	14(1)	4(1)	2(1)	0(1)
C(27)	12(1)	14(1)	15(1)	3(1)	4(1)	2(1)
C(28)	12(1)	14(1)	14(1)	4(1)	4(1)	2(1)
C(29)	13(1)	23(1)	15(1)	4(1)	6(1)	0(1)
C(30)	12(1)	15(1)	14(1)	3(1)	5(1)	2(1)
C(31)	13(1)	14(1)	16(1)	3(1)	7(1)	3(1)
C(32)	12(1)	15(1)	18(1)	3(1)	4(1)	2(1)

Table S13. Hydrogen coordinates (x 10^4) and isotropic displacement parameters ($Å^2$x 10^3) for CG (**4**).

	x	y	z	U(eq)
H(5A)	6130(50)	4370(50)	7660(30)	34(10)
H(6A)	4380(40)	5010(40)	11380(30)	21(8)
H(7A)	560(60)	4590(60)	10790(40)	50(12)
H(8A)	-1370(60)	2990(60)	8370(40)	41(12)
H(2)	3889	7776	2871	31
H(4A)	4206	11331	5099	49
H(4B)	2237	11073	4319	49
H(4C)	3831	10782	3734	49
H(6)	2634	10668	6317	23
H(7)	1809	9704	7723	22
H(9)	2100	4617	5979	20
H(11)	90	4308	7040	16
H(12)	4318	5695	8894	17
H(13A)	6271	3643	9400	20
H(13B)	4844	2053	8614	20
H(14)	2838	3213	9824	18
H(15)	2095	6780	9938	17
H(16)	-728	5688	8772	18
H(13)	3590(60)	-530(60)	-490(40)	46(11)
H(14A)	6210(50)	-1620(60)	-1260(40)	40(11)
H(15A)	10670(60)	-820(60)	-320(40)	52(12)
H(16A)	10440(50)	-1550(50)	1360(30)	28(9)
H(18)	7088	9732	7154	22
H(20A)	7288	11121	4888	39
H(20B)	9227	11326	5703	39
H(20C)	7612	11820	6247	39
H(22)	8578	9107	3698	24
H(23)	9052	6711	2309	22
H(25)	7891	3407	4069	19
H(27)	9710	1941	3184	16
H(28)	6125	1903	914	16
H(29A)	4486	-1126	1259	20
H(29B)	4064	845	1791	20
H(30)	7138	-1661	490	16

H(31)	8667	1341	41	16
H(32)	11049	1502	1596	19

Table S14. Torsion angles [°] for CG (**4**).

C(10)-O(2)-C(1)-O(1)	178.0(2)
C(10)-O(2)-C(1)-C(2)	-2.2(3)
O(1)-C(1)-C(2)-C(3)	-178.5(3)
O(2)-C(1)-C(2)-C(3)	1.7(4)
C(1)-C(2)-C(3)-C(5)	0.3(4)
C(1)-C(2)-C(3)-C(4)	-179.0(2)
C(2)-C(3)-C(5)-C(10)	-1.9(4)
C(4)-C(3)-C(5)-C(10)	177.5(2)
C(2)-C(3)-C(5)-C(6)	178.3(2)
C(4)-C(3)-C(5)-C(6)	-2.3(4)
C(10)-C(5)-C(6)-C(7)	-1.0(3)
C(3)-C(5)-C(6)-C(7)	178.8(2)
C(5)-C(6)-C(7)-C(8)	1.5(3)
C(11)-O(3)-C(8)-C(9)	-9.2(3)
C(11)-O(3)-C(8)-C(7)	170.92(19)
C(6)-C(7)-C(8)-O(3)	179.0(2)
C(6)-C(7)-C(8)-C(9)	-0.9(3)
O(3)-C(8)-C(9)-C(10)	179.87(19)
C(7)-C(8)-C(9)-C(10)	-0.3(3)
C(1)-O(2)-C(10)-C(9)	-177.9(2)
C(1)-O(2)-C(10)-C(5)	0.7(3)
C(8)-C(9)-C(10)-O(2)	179.47(19)
C(8)-C(9)-C(10)-C(5)	0.8(3)
C(6)-C(5)-C(10)-O(2)	-178.7(2)
C(3)-C(5)-C(10)-O(2)	1.4(3)
C(6)-C(5)-C(10)-C(9)	-0.2(3)
C(3)-C(5)-C(10)-C(9)	179.9(2)
C(12)-O(4)-C(11)-O(3)	60.6(2)
C(12)-O(4)-C(11)-C(16)	-57.1(2)
C(8)-O(3)-C(11)-O(4)	68.6(2)
C(8)-O(3)-C(11)-C(16)	-168.78(17)
C(11)-O(4)-C(12)-C(13)	179.35(17)
C(11)-O(4)-C(12)-C(14)	57.7(2)

O(4)-C(12)-C(13)-O(5)	59.7(2)
C(14)-C(12)-C(13)-O(5)	-179.12(19)
O(4)-C(12)-C(14)-O(6)	-176.60(18)
C(13)-C(12)-C(14)-O(6)	64.2(2)
O(4)-C(12)-C(14)-C(15)	-56.0(2)
C(13)-C(12)-C(14)-C(15)	-175.26(19)
O(6)-C(14)-C(15)-O(7)	-61.3(2)
C(12)-C(14)-C(15)-O(7)	-178.76(17)
O(6)-C(14)-C(15)-C(16)	173.76(18)
C(12)-C(14)-C(15)-C(16)	56.3(2)
O(7)-C(15)-C(16)-O(8)	-61.2(2)
C(14)-C(15)-C(16)-O(8)	64.7(2)
O(7)-C(15)-C(16)-C(11)	179.55(17)
C(14)-C(15)-C(16)-C(11)	-54.5(2)
O(4)-C(11)-C(16)-O(8)	-64.0(2)
O(3)-C(11)-C(16)-O(8)	173.63(17)
O(4)-C(11)-C(16)-C(15)	54.3(2)
O(3)-C(11)-C(16)-C(15)	-68.1(2)
C(26)-O(10)-C(17)-O(9)	178.55(19)
C(26)-O(10)-C(17)-C(18)	-1.6(3)
O(9)-C(17)-C(18)-C(19)	-178.5(2)
O(10)-C(17)-C(18)-C(19)	1.6(3)
C(17)-C(18)-C(19)-C(21)	-0.5(4)
C(17)-C(18)-C(19)-C(20)	-179.8(2)
C(18)-C(19)-C(21)-C(26)	-0.7(3)
C(20)-C(19)-C(21)-C(26)	178.7(2)
C(18)-C(19)-C(21)-C(22)	178.4(2)
C(20)-C(19)-C(21)-C(22)	-2.2(4)
C(26)-C(21)-C(22)-C(23)	-2.0(4)
C(19)-C(21)-C(22)-C(23)	178.9(2)
C(21)-C(22)-C(23)-C(24)	0.5(4)
C(27)-O(11)-C(24)-C(25)	-7.2(3)
C(27)-O(11)-C(24)-C(23)	171.97(19)
C(22)-C(23)-C(24)-O(11)	-177.3(2)
C(22)-C(23)-C(24)-C(25)	1.8(3)
O(11)-C(24)-C(25)-C(26)	176.65(19)
C(23)-C(24)-C(25)-C(26)	-2.4(3)
C(17)-O(10)-C(26)-C(25)	-179.42(19)
C(17)-O(10)-C(26)-C(21)	0.4(3)

C(24)-C(25)-C(26)-O(10)	-179.33(19)
C(24)-C(25)-C(26)-C(21)	0.8(3)
C(22)-C(21)-C(26)-O(10)	-178.5(2)
C(19)-C(21)-C(26)-O(10)	0.7(3)
C(22)-C(21)-C(26)-C(25)	1.4(3)
C(19)-C(21)-C(26)-C(25)	-179.4(2)
C(28)-O(12)-C(27)-O(11)	62.8(2)
C(28)-O(12)-C(27)-C(32)	-57.5(2)
C(24)-O(11)-C(27)-O(12)	72.5(2)
C(24)-O(11)-C(27)-C(32)	-163.18(17)
C(27)-O(12)-C(28)-C(29)	-175.15(17)
C(27)-O(12)-C(28)-C(30)	63.3(2)
O(12)-C(28)-C(29)-O(13)	171.89(17)
C(30)-C(28)-C(29)-O(13)	-69.5(2)
O(12)-C(28)-C(30)-O(14)	177.06(16)
C(29)-C(28)-C(30)-O(14)	60.0(2)
O(12)-C(28)-C(30)-C(31)	-62.0(2)
C(29)-C(28)-C(30)-C(31)	-179.07(18)
O(14)-C(30)-C(31)-O(15)	-64.9(2)
C(28)-C(30)-C(31)-O(15)	176.62(17)
O(14)-C(30)-C(31)-C(32)	174.44(17)
C(28)-C(30)-C(31)-C(32)	55.9(2)
O(12)-C(27)-C(32)-O(16)	-72.8(2)
O(11)-C(27)-C(32)-O(16)	163.89(17)
O(12)-C(27)-C(32)-C(31)	49.3(2)
O(11)-C(27)-C(32)-C(31)	-74.0(2)
O(15)-C(31)-C(32)-O(16)	-49.6(2)
C(30)-C(31)-C(32)-O(16)	68.8(2)
O(15)-C(31)-C(32)-C(27)	-167.28(17)
C(30)-C(31)-C(32)-C(27)	-48.9(2)

Table S15. Hydrogen bonds for CG (**4**) [Å and °].

D-H...A	d(D-H)	d(H...A)	d(D...A)	<(DHA)
O(5)-H(5A)...O(9)	0.93(4)	1.92(4)	2.836(3)	172(4)
O(6)-H(6A)...O(1)#1	0.80(4)	2.02(4)	2.813(3)	175(3)
O(8)-H(8A)...O(5)#2	0.74(4)	2.09(4)	2.805(3)	163(5)

O(13)-H(13)...O(14)	0.90(5)	1.95(5)	2.693(2)	140(4)
O(14)-H(14A)...O(9)#3	0.80(4)	2.02(4)	2.794(2)	164(4)
O(15)-H(15A)...O(13)#4	0.88(5)	1.94(5)	2.810(2)	170(5)
O(16)-H(16A)...O(7)#5	0.84(4)	2.23(4)	2.940(3)	143(3)
O(16)-H(16A)...O(15)	0.84(4)	2.33(4)	2.809(3)	116(3)

Symmetry transformations used to generate equivalent atoms:

#1 x,y,z+1   #2 x-1,y,z   #3 x,y-1,z-1   #4 x+1,y,z

#5 x+1,y-1,z-1

References

1. CrysAlisPro Version 1.171.37.35 (2014) and 1.171.38.46 (Rigaku Oxford Diffraction, 2015)

2. SUPERFLIP: L. Palatinus and G. Chapuis, J. Appl. Crystallogr., 2007, 40, 786-790; SHELXL: G. M. Sheldrick, ActaCryst, 2015, C71, 3-8.

3. ShelXle: a Qt graphical user interface for SHELXL C. B. Hübschle, G. M. Sheldrick and B. Dittrich

ShelXle: a Qt graphical user interface for SHELXL. J. Appl. Cryst., 44, (2011) 1281-1284.

4. Mercury: C. F. Macrae, P. R. Edgington, P. McCabe, E. Pidcock, G. P. Shields, R. Taylor, Towler and van der Streek, J. Appl. Crystallogr., 2006, 39, 453-457.
